# Splice-Junction-Based Mapping of Alternative Isoforms in the Human Proteome

**DOI:** 10.1016/j.celrep.2019.11.026

**Published:** 2019-12-10

**Authors:** Edward Lau, Yu Han, Damon R. Williams, Cody T. Thomas, Rajani Shrestha, Joseph C. Wu, Maggie P.Y. Lam

**Affiliations:** 1 Stanford Cardiovascular Institute, Department of Medicine, Stanford University, Palo Alto, CA, USA; 2 Department of Radiology, School of Medicine, Stanford University, Palo Alto, CA, USA; 3 Consortium for Fibrosis Research and Translation, Anschutz Medical Campus, University of Colorado, Aurora, CO, USA; 4 Departments of Medicine-Cardiology and Biochemistry and Molecular Genetics, Anschutz Medical Campus, University of Colorado, Aurora, CO, USA; 5 Lead Contact

## Abstract

The protein-level translational status and function of many alternative splicing events remain poorly understood. We use an RNA sequencing (RNA-seq)-guided proteomics method to identify protein alternative splicing isoforms in the human proteome by constructing tissue-specific protein databases that prioritize transcript splice junction pairs with high translational potential. Using the custom databases to reanalyze ~80 million mass spectra in public proteomics datasets, we identify more than 1,500 noncanonical protein isoforms across 12 human tissues, including ~400 sequences undocumented on TrEMBL and RefSeq databases. We apply the method to original quantitative mass spectrometry experiments and observe widespread isoform regulation during human induced pluripotent stem cell cardiomyocyte differentiation. On a proteome scale, alternative isoform regions overlap frequently with disordered sequences and post-translational modification sites, suggesting that alternative splicing may regulate protein function through modulating intrinsically disordered regions. The described approach may help elucidate functional consequences of alternative splicing and expand the scope of proteomics investigations in various systems.

## In Brief

The translation and function of many alternative splicing events await confirmation at the protein level. Lau et al. use an integrated proteotranscriptomics approach to identify non-canonical and undocumented isoforms from 12 organs in the human proteome. Alternative isoforms interfere with functional sequence features and are differentially regulated during iPSC cardiomyocyte differentiation.

## Graphical Abstract

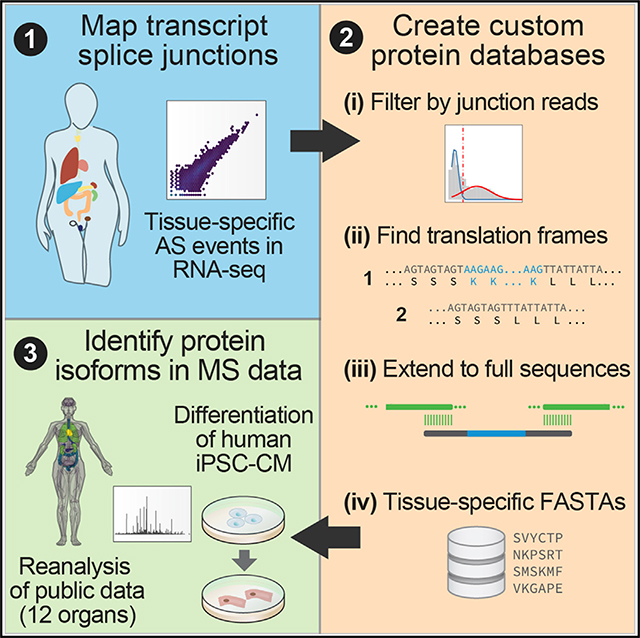

## INTRODUCTION

Protein species outnumber coding genes in eukaryotes, in part, because one gene can encode multiple transcripts through alternative splicing (AS) (Aebersold et al., 2018; Smith and Kelleher, 2018). RNA-seq experiments have discovered over 100,000 AS transcripts in the human genome (Pan et al., 2008; Wang et al., 2008), but identifying which AS isoforms are functionally important is a major unmet goal, and critically, most have never been detected at the protein level. Although computational approaches can predict isoform conservation and function (Li et al., 2017; Rodriguez et al., 2013) and Ribo-seq can survey alternative transcripts engaged to ribosomes (Weatheritt et al., 2016; van Heesch et al., 2019), these techniques stop short of assessing AS protein products empirically.

Mass spectrometry (MS)-based proteomics is the standard tool for unbiased protein identification, but it faces technical challenges in identifying AS isoforms. Chief among them, MS-based shotgun proteomics typically identifies proteins by searching mass spectra against peptide sequences in a protein database; hence, an isoform sequence not found in common databases is precluded from identification by search algorithms in typical experiments. The commonly used protein database SwissProt catalogs on average ~1.1 alternative isoforms per human gene and much fewer in other organisms. Larger sequence databases (e.g., TrEMBL and RefSeq) exist, but it is unclear whether the majority of deposited sequences are bona fide isoforms or gene fragments, polymorphisms, and redundant entries. Partly due to these limitations, the protein molecular functions of most AS events remain severely under-characterized, and a systematic picture is lacking on how AS rewires proteome functions (Tress et al., 2017a, 2017b).

Several approaches have been proposed to improve MS identification of AS isoforms, including the curation of splice variant databases (Tavares et al., 2014; Mo et al., 2008) and *de novo* 6-frame translation of genome sequences (Power et al., 2009; Fermin et al., 2006). More recently, RNA-seq has been leveraged with some success to identify variant sequences not found in standard protein databases (Ning and Nesvizhskii, 2010; Zickmann and Renard, 2015; Verbruggen et al., 2019; Cifani et al., 2018), corroborating the potential utility of an RNA-guided approach for discovering protein AS isoforms. Thus far, however, studies of this type have largely been performed in transformed cell lines or tumors known to have aberrant splicing (Ning and Nesvizhskii, 2010; Koch et al., 2014; Sheynkman et al., 2013; Evans et al., 2012; Liu et al., 2017). Moreover, many custom RNA-guided databases remain imprecise and contain large numbers of low-quality sequences that likely cannot be detected in the biological sample (e.g., from translation of multiple reading frames), suggesting there is a need for continued refinement of *in silico* translation and evaluation methods.

We describe a method that translates splice junction pairs from RNA-seq data to guide protein isoform discovery. We prioritize *in silico* translation of AS events with appreciable read counts and enforce one-frame translation to limit database size inflation and the associated false positives in database search (Alfaro et al., 2014; Ning and Nesvizhskii, 2010). The custom databases were used to recover AS protein isoforms from public MS data on 12 primary human tissues as well as original MS data on human induced pluripotent stem cell (iPSC)-directed cardiac differentiation, the latter providing a model to assess protein isoform changes during cellular differentiation. The results support identification of noncanonical protein isoforms as well as uncharacterized junction peptides from MS experiments.

## RESULTS

### Generation of Junction-Centric Protein Sequence Databases

**Figure 1.**
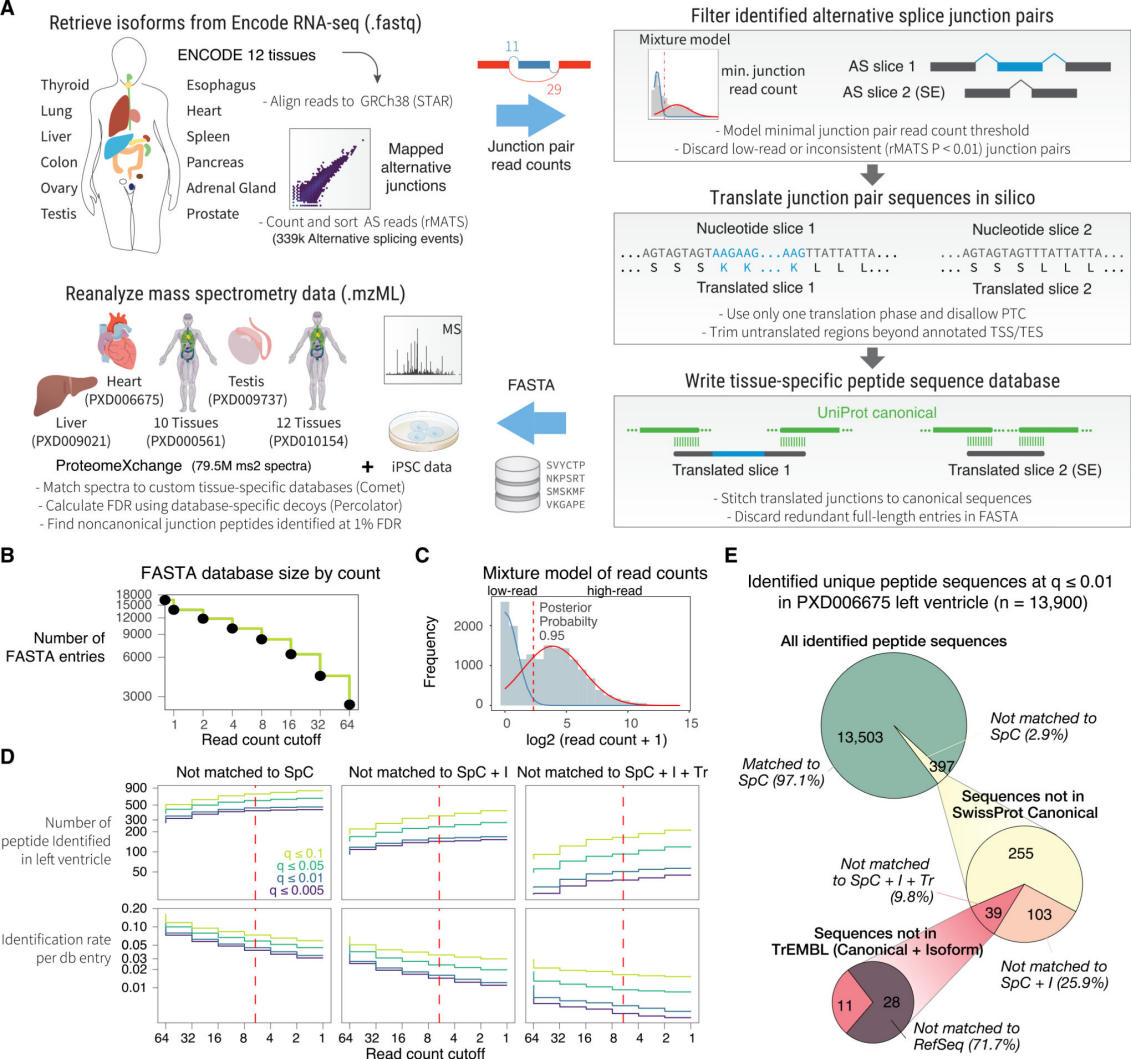
Splice-Junction-Centric Approach to Identify Protein Isoforms (A) Schematic of the method. ENCODE RNA-seq data from 12 human tissues are mapped to GRCh38. AS pairs are extracted then filtered by junction read counts and consistency. Candidate junctions are trimmed using Ensembl GTF-annotated translation start site (TSS) and translation end site (TES) and then translated in-frame by using either GTF-annotated reading frames or by choosing a frame that does not lead to PTC. The translated junction pairs are extended to encompass the full protein sequence. The created custom tissue-specific databases are used to identify noncanonical protein isoforms in public and original MS data. (B) Number of translated sequences versus minimal skipped junction read count threshold following *in silico* translation in ENCODE human heart data. Inclusion of low-read junctions increases database size. (C) Gaussian mixture fitting overlaid on skipped junction read counts of all AS events in the heart database. Dotted line: chosen threshold. (D) Number of identified noncanonical isoform sequences in the reanalyzed human heart left ventricle MS data versus junction read count thresholds. Color: Percolator FDR cutoff calculated with database-specific decoys. (E) Proportion of identified distinct peptide sequences in the left ventricle dataset (13,900 total) not matchable to SwissProt canonical (SpC), SwissProt canonical + isoform (SpC + I), TrEMBL (Tr), or RefSeq.

We assembled a computational workflow to translate AS junctions to protein sequences *in silico* (Figure 1A). Differential exon usage analysis is a common transcriptomics tool to assess the percent spliced in (PSI) index of splice events and exon inclusion across samples. We reasoned that by focusing our analysis on alternative junction pairs rather than all assembled transcripts, we can target more relevant splicing events within a tissue and create precise sequence databases. We retrieved ENCODE RNA-seq data on the GTEx tissue collection of human heart, lungs, liver, pancreas, transverse colon, ovary, testis, prostate, spleen, thyroid, esophagus, and adrenal gland, each containing 101 nt paired-end (PE) total RNA-seq data with 2 biological replicates passing ENCODE consortium-wide quality control. Sequence reads are mapped to GRCh38 to identify splice junctions from GTF annotations or *de novo.* To gather AS events, we use rMATS (Shen et al., 2014) to count the exon-included and exon-excluded junction-spanning reads for each AS event, including alternative 3’ splice site (A3SS), alternative 5′ splice site (A5SS), mutually exclusive exon (MXE), skipped exon (SE), and retained intron (RI).

We use four criteria to select splice junction pairs that are more likely detectable in MS experiments (Figure 1A). (1) The skipped junction read counts of an AS event must pass a sample-specific threshold. (2) We use the statistical model in rMATS to remove splice events with significantly different exon usage across technical and biological replicates in the same tissue (p ≤ 0.01). (3) We prioritize transcripts with known annotated translation start sites and frame that can be translated in-frame without premature termination codons (PTCs). Where an unambiguous translation frame is not available, we use one frame that results in the longest translatable sequence with no PTCs. (4) To ensure reliable junction peptides can be identified that span constitutive and alternative exons, we stitch both translated slices in a splice pair (each containing one upstream exon, the alternative exons, and one downstream exon) back-to-back to the full-length canonical sequences from SwissProt through a 10-amino-acid (aa) overhang. In cases of SE and RI, one alternative exon would be empty (skipped). Orphan slices that are not extensible back to canonical sequences are discarded and redundant sequences combined. The translated junction pairs passing these criteria are written to a FASTA file for a database search.

From the ENCODE RNA-seq data, we mapped a median of 72,194 AS events per tissue, with the adrenal gland having the fewest (66,160) and testis the most events (91,895). The most common AS type was SE, accounting for 65.1% of all identified events, followed by A3SS (10.8%), then RI (10.1), MXE (7.3%), and A5SS (6.7%). The mapped splice junctions show a broad distribution of skipped junction read counts (Figure S1). As cellular transcription is noisy and MS-based proteomics typically omits products of lowly expressed genes (Ramakrishnan et al., 2009), we sought a junction read count threshold to minimize the inclusion of non-translatable junctions in the custom database. Indeed, we observed that database entry counts scale with read count filter in a log-linear relationship in the analyzed RNA sequencing data (Figure 1B). We, thus, removed low-abundance junctions based on the excluded junction read count in the two alternative junctions created by a splice event, such that only AS events expressed at appreciable levels were retained. To identify optimal read count cutoffs, a Gaussian mixture model was used to separate splice junctions into lowly and highly expressed groups. A read count cutoff was applied at 0.95 posterior probability of a junction being in the highly expressed group (Figure 1C), which in the ENCODE heart RNA-seq sample corresponds to ≥4 mapped junction reads.

We first evaluated how this read count filter influenced the number of identifiable splice junction peptides in human heart left ventricle MS data under fixed false discovery rates (FDRs), focusing on junctions that correspond to noncanonical isoforms not found in the SwissProt *Homo sapiens* canonical database. FDR is estimated using score distributions of custom decoy sequences generated from the translated tissue-specific (canonical and noncanonical) sequences. We saw that the number of identifiable junction peptides gradually plateaued at 4-count cutoff under stringent significance threshold (Percolator q-value ≤ 0.01), whereas low-confidence matches (q ≥ 0.05) continued to accrue at lower cutoffs, reflecting an inflation of false positives when low-read junctions were included. In parallel, the proportion of sequences identified per FASTA entry fell as databases grew in size; hence, the cutoff chosen (i.e., 4) balanced identification rate with FDR (Figure 1D). Compared to the indiscriminate 3-frame translation of assembled transcripts or FASTA databases translated from RNA-seq of mismatched tissues (e.g., liver), the junction-based approach supported a greater proportion of undocumented sequence identification at low FDR, indicating the database preferentially excludes low-quality sequences (Figure S1). In total, the search identified 13,900 distinct peptides sequences at 1% FDR by using a reverse decoy database followed by Percolator. Out of these peptides, 397 (2.9%) were not found in the manually curated SwissProt canonical database (Figure 1E), 142 (35.7%) were not found in the SwissProt canonical + isoform database, and 39 (9.8%) were not found in the automatically annotated sequence collection TrEMBL. Taken together, these results show that our approach can identify noncanonical and undocumented isoforms at low FDR.

### Identification of Noncanonical Splice Junctions across Tissues

**Figure 2.**
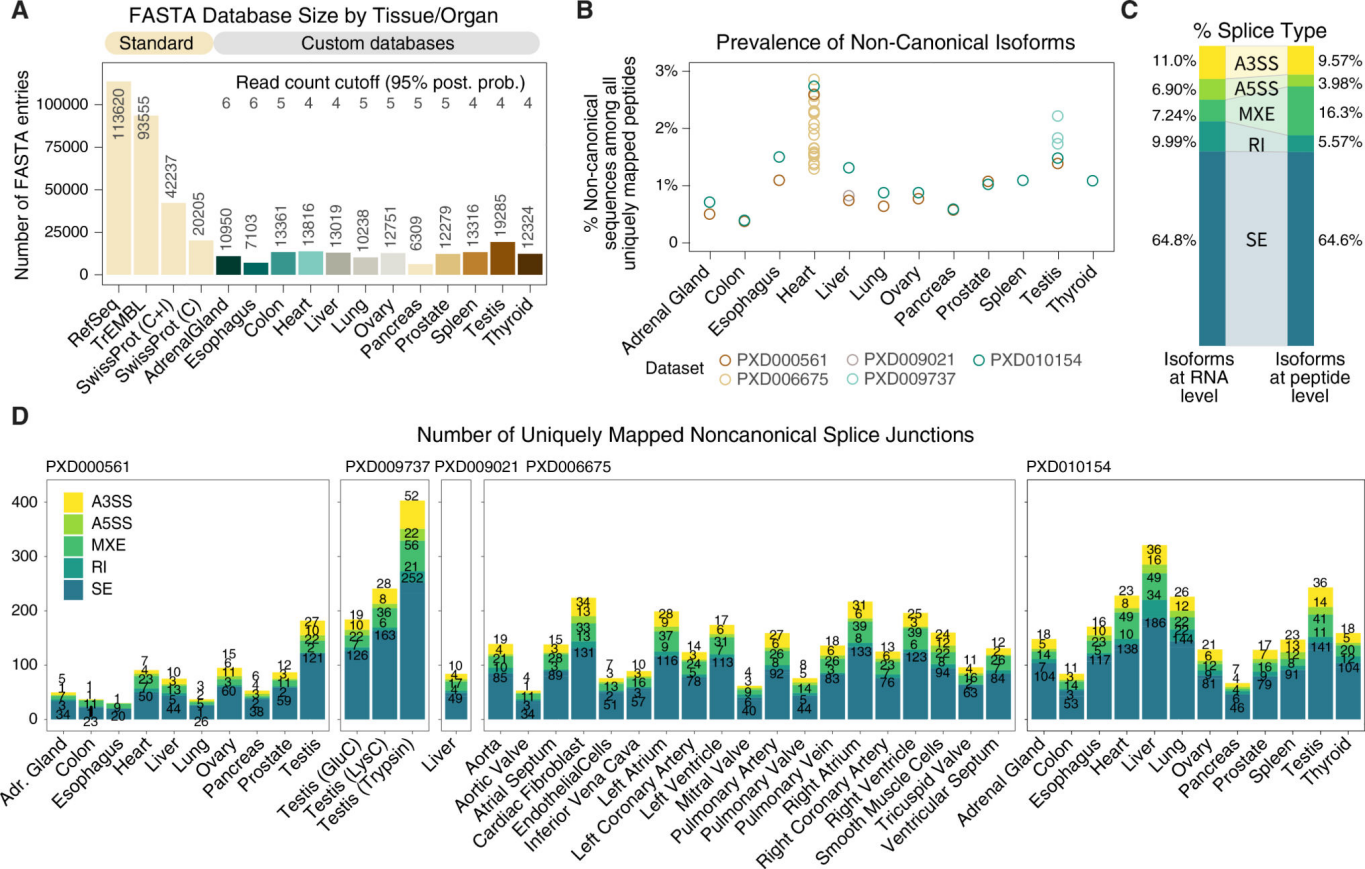
Identification of Noncanonical Isoforms in the Human Proteome (A) Comparison on the number of sequences in standard databases (RefSeq TrEMBL, SwissProt canonical + isoform, and SwissProt canonical) versus the custom tissue-specific databases. The custom databases have fewer sequences than SwissProt (B) The proportion of distinct peptides uniquely mappable to noncanonical isoforms per tissue, with the heart and testis particularly enriched in noncanonical isoforms. Color of data points corresponds to each of 5 reanalyzed human proteome datasets. (C) Proportion of AS types in RNA-seq data (left) compared to identified noncanonical peptides (right), showing higher translatable rate for MXE. (D) The number of uniquely identified noncanonical junction peptides at 1% FDR across tissues in 5 reanalyzed human proteome datasets (ProteomeXchange: PXD000561, PXD009737, PXD009021, PXD006675, and PXD010154), including noncanonical sequences from known isoforms and undocumented sequences. Color: AS type (A3SS, A5SS, MXE, RI, and SE).

We next built custom isoform databases for all 12 analyzed tissues. The filtering strategy drastically reduced the number of entries in the custom databases (Figure 2A), e.g., the heart-specific database contains 13,816 entries, versus 42,237 in the Swissprot human reference proteome (20,225 canonical + 22,012 isoform sequences), 93,555 sequences in TrEMBL, and 113,620 in RefSeq. Across 12 tissues, the custom databases contain on average 11,911 entries, with the pancreas database having the fewest sequences (6,309) and the testis the most (19,285). All generated databases are markedly sparser than SwissProt, TrEMBL, RefSeq, or 3-frame translated databases. This is expected as RNA-seq data capture only transcripts from genes expressed in a particular tissue due to tissue-specific gene regulation.

We used the custom databases to perform a secondary analysis on 4 MS datasets containing high-resolution Orbitrap FT/FT spectra on human tissues, comprising a dataset on 10 tissues (Kim et al., 2014), 1 on testis by using 3 proteases (Sun et al., 2018), 1 on liver with extensive fractionation, 1 on the heart dissected into 16 anatomical regions plus 3 isolated cell types (Doll et al., 2017), and 1 on all 12 matching tissues (Wang et al., 2019). In total, we reanalyzed 1,927 MS data files with 79.6 million MS2 fragmentation spectra. In the heart, which is the most comprehensively reanalyzed tissue here, the translated isoform sequences belonged to 6,351 genes, of which 5,731 (90.4%) were identifiable by at least 1 isoform in the reanalyzed MS data. Of all translated isoform entries (whether canonical or alternative) in the heart, 23% were uniquely identifiable by a peptide that mapped to exactly one FASTA entry in the database.

Because all splice junctions were translated pairs, uniquely mappable peptides (i.e., peptide mapped to only one FASTA entry) represent isoform-specific peptides that are not shared between the two translated isoforms within the same gene in the tissue. In most tissues, ~1% of uniquely mapped peptides correspond to a noncanonical isoform, whereas this proportion is higher in the testis and the heart, suggesting AS may preferentially influence the proteomes of these two tissues (Figure 2B). Most identified noncanonical peptides (64.6%) arose from SE, comparable to the proportion of SE in RNA-seq AS events (64.8%); MXE appeared to have higher translational potential (16.3% peptides versus 7.2% RNA), whereas RI produced relatively few protein products (5.6% peptides versus 10.0% RNA) (Figure 2C). In total, we identified 3,418 distinct and uniquely mapped peptides at 1% FDR that were not found in canonical SwissProt, corresponding to up to 1,555 noncanonical isoforms in 1,189 genes (Figure 2D).

**Figure 3.**
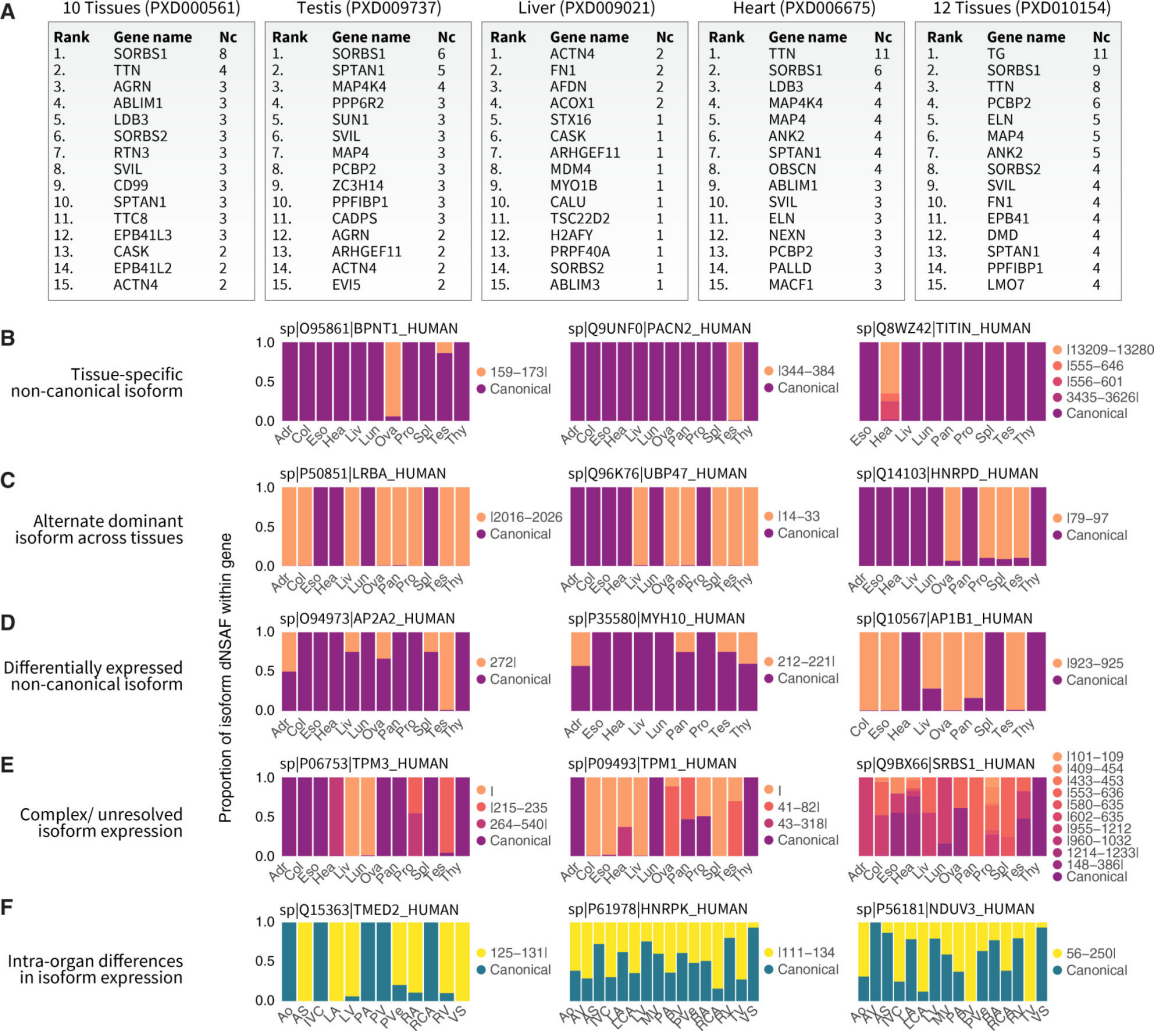
Protein Isoform Diversity and Tissue-Specific Expression (A) Top 15 genes associated with the most identified noncanonical isoform (Nc) sequences across reanalyzed human proteome datasets. (B-E) Distributed normalized spectral abundance factor (dNSAF)-based assessment of relative isoform prevalence for each gene across tissues in cases where unique peptide junctions are resolvable. Isoforms across databases are harmonized by junction position and sequence alignment (insertion | deletion on legends) against the canonical sequence. Examples show 4 classes of tissue distributions in the data. (B) Tissue-specific isoforms confined to only one assessed tissue, frequently the testis and ovary but also the heart. (C) Two isoforms of a gene with alternate expression in different tissues. (D) Quantitative differences in the expression levels of alternative versus canonical isoforms. (E) Complex patterns of multiple junctions, including instances where the relative abundance of the canonical isoform is indeterminable by dNSAF in some tissues due to the absence of unique sequences. (F) Tissue-specific expression is also evident in anatomical regions within the heart, including isoforms preferentially found in the myocardium over the vasculature. Adr, adrenal gland; Col, colon; Eso, esophagus; Hea, heart; Liv, liver; Lun, lung; Ova, ovary; Pan, pancreas; Pro, prostate; Spl, spleen; Tes, testis; Thy, thyroid; Ao, aorta; AV, aortic valve; AS, atrial septum; IVC, inferior vena cava; LA, left atrium; LCA, left coronary artery; LV, left ventricle; MV, mitral valve; PA, pulmonary artery; PV, pulmonary valve; PVe, pulmonary vein; RA, right atrium; RCA, right coronary artery; RV, right ventricle; TV, tricuspid valve; VS, ventricular septum.

Proteins with multiple noncanonical isoforms are found in diverse pathways, including muscle contraction, metabolism, and signaling. A number of proteins including SORBS1 and MAP4K4 had multiple noncanonical isoforms that were detectable across multiple tissues, whereas the protein with the most isoforms identified was titin (Figure 3A), which is also the largest protein in the human genome with the most exons and whose splicing has been widely implicated in congenital heart diseases (Guo et al., 2012; Herman et al., 2012). Several categories of tissue specificity in protein isoform expression are recognizable. First, a number of noncanonical isoforms were found only in one analyzed tissue, frequently the testis or ovary but in some cases also the heart; e.g., a 3′(2′),5′-bisphosphate nucleotidase 1 (BPNT1) isoform was identified primarily in the ovary, whereas noncanonical forms of titin (TTN) were identified only in the heart (Figure 3B). Second, a number of proteins alternate in dominant isoforms across tissues in the body, e.g., the noncanonical form of ubiquitin carboxyl-terminal hydrolase 47 (UBP47), a ubiquitin-specific protease involved in base-excision repair, is present in the liver, ovary, pancreas, spleen, testis, and thyroid but not the other 6 analyzed tissues; whereas an alternative isoform for heterogeneous nuclear ribonucleoprotein D0 (HNRPD) was found only in the ovary, prostate, spleen, and testis (Figure 3C). A third group of proteins including myosin-10 (MYH10) showed broad tissue expression of both canonical and noncanonical forms but with different relative abundance across tissues (Figure 3D). Because of difficulties in accurately measuring quantity from label-free methods with singular junction peptides, some of the alternative dominant isoforms we nominated in Figure 3C may also, in fact, be differentially expressed across tissues quantitatively. A fourth group of proteins show complex isoform patterns of multiple sequences that are difficult to unravel due to degenerate junction combinations (Figure 3E).

Likewise, we observed differential isoform expression across different anatomical regions of one organ (the heart); e.g., the vesicular trafficking protein transmembrane emp24 domain-containing 2 (TMED2) expresses a noncanonical form primarily in myocardial (atrial and ventricular) tissues but not vascular or valvular tissues; whereas a noncanonical isoform of NADH dehydrogenase (ubiquinone) flavoprotein 3, mitochondrial (NDUFV3) showed the opposite pattern of preferential expression in the vasculature (Figure 3F). Overall, the results reflect complex tissue-dependent regulations of AS at the protein level.

### Undocumented Peptide Sequences in Existing Mass Spectrometry Data

**Figure 4.**
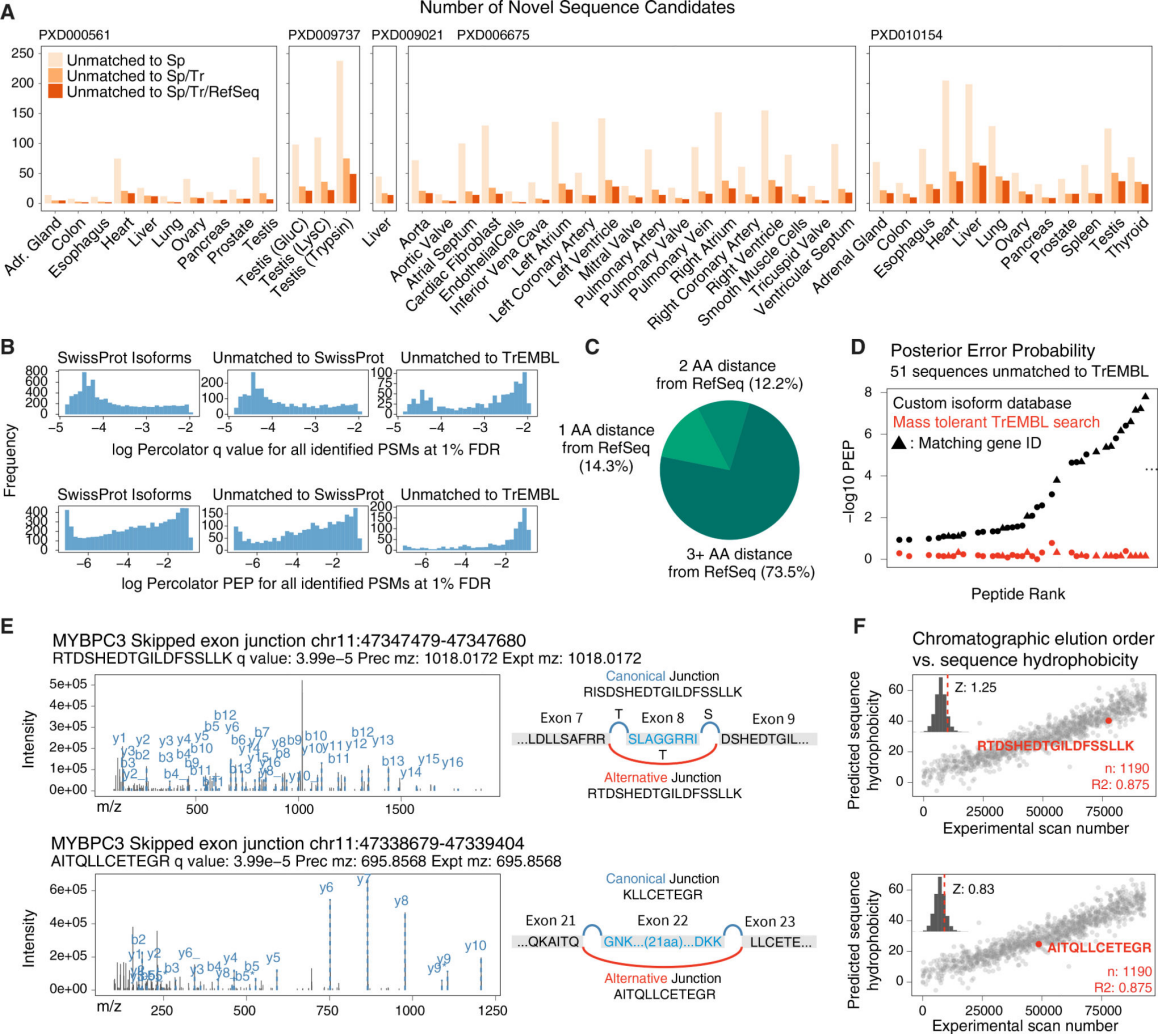
Splice Junctions Include Peptides Undocumented in Common Databases (A) Number of undocumented sequence candidates in each reanalyzed tissue across 5 public human proteome datasets. (B) Distribution of Percolator FDR and posterior error probability (PEP) of noncanonical sequences that are matched to SwissProt isoforms (left) against those not in SwissProt (middle) or TrEMBL (right). (C) Proportion of peptide sequences that are not mappable to RefSeq, allowing 1, 2, or 3 mismatches. (D) Comparison of −log10 Percolator PEP for 51 left ventricle peptide spectrum matches to sequences not in TrEMBL versus the results from the corresponding spectra in a mass tolerant open search against TrEMBL. (E) Tandem mass spectra of two identified splice junction peptides (RTDSHEDTGILDFSSLLK and AITQLLCETEGR for MYPBC3) not found in SwissProt, TrEMBL, or RefSeq. (F) The predicted hydrophobicity of the two undocumented sequences shows the sequence eluted at the expected retention time when the spectrum was acquired. Inset: *Z* score of residuals from best-fit line.

We identified isoform sequences that are undocumented in common databases. At 1% FDR, we identified 1,385 peptides in 681 genes that were not in SwissProt (canonical + isoform). Among them, 566 peptides were also not in TrEMBL, which encompasses all SwissProt entries plus computationally annotated and unreviewed sequences, and 453 peptides in 366 genes were not matched to the larger RefSeq database (Figure 4A). Undocumented peptides are particularly enriched in the testis, which is known to differ markedly from other tissues in splicing pattern (Yeo et al., 2004). On average, the undocumented sequences had higher adjusted p values and posterior error probability than noncanonical peptides in TrEMBL (Figure 4B); hence, some may be false-positive identification. However, a lower score distribution for these sequences could also be due to the lower abundance of alternative isoforms (Blencowe, 2017) and the known enrichment of lysine at splice junctions producing miscleavages (Wang et al., 2018b) whose scoring is penalized by Percolator (The et al., 2016). Regardless of search engine scores, the assignment of variant peptides demands caution and alternative explanation including mass shifts due to post-translational modifications (PTMs) or single-amino-acid variant polymorphisms (SAAVs). Hence, to further evaluate the undocumented peptide matches, we considered several additional lines of evidence.

First, we used a sequence alignment algorithm to assess whether the identified undocumented peptides may be matchable to RefSeq when allowing one or more mutations. We found that the majority (70.8%) of these sequences cannot be matched to RefSeq even with 2 mismatches; hence, the absolute majority of identified spectra are unlikely to arise from SAAV differing from the reference proteome or other unaccounted for mass shifts at a single residue (Figure 4C). Second, we evaluated whether the spectra may be better matched to a mass-tolerant open search for PTM (Figure 4D). We subjected the left ventricle dataset to a comprehensive MSFragger open search against TrEMBL, allowing a −200 to +400 Da mass shift, followed by Percolator filtering, which identified 13,880 peptides from 8,702 protein groups at 1% FDR. Among the spectra identified to 51 undocumented peptides in the left ventricle, 38 were matched to a peptide using MSFragger and 14 were matched to the same gene ID, but only one spectrum was confidently identified at 1% FDR. The other spectra did not pass FDR cutoff (median Percolator q, 0.15), and all spectra had considerably higher adjusted p values than in the custom database search. By contrast, 267 out of the 394 (67%) noncanonical isoform sequences found in TrEMBL in the same sample were matched to identical sequences in the open search; hence, the AS database search has additional identification power for a subset of spectra over open search.

Third, we manually inspected the fragmentation spectra of undocumented isoform peptides. Among the undocumented peptides are two SE junctions for myosin-binding protein C3 (MYBPC3) (RTDSHEDTGILDFSSLLK and AITQLLCETEGR), corresponding from skipping of exon 8 and exon 22, respectively (Figure 4E). Both sequences were identified from high-quality spectra with large proportions of matched fragment ions at Percolator q ≤ 0.01. Using the SSRC algorithm to determine the hydrophobicity coefficient of peptides *de novo* (Krokhin et al., 2004), we found that the peptides also eluted at the expected retention time based on their assigned sequences (Figure 4F). However, there are also undocumented sequence matches with unexpected retention time that are more likely to be false positives (Data S1). We suggest that improving sequence elution time prediction algorithms may be a useful determinant to adjudicate the validity of sequence variants.

Finally, we used targeted MS to experimentally verify a subset of undocumented sequence matches (Deutsch et al., 2016; Nesvizhskii et al., 2007). Using parallel reaction monitoring (PRM) (Peterson et al., 2012), we co-analyzed an independent biological replicate human heart lysate sample with synthetic isotope-labeled peptide standards for a subset of isoform sequences (Table S1). PRM assays target the junction sequences by acquiring tandem mass spectra using specified accurate masses and retention time of the endogenous and synthetic peptides, which should share elution time and fragmentation spectra patterns. To validate the method, we first set up PRM assays for two known pyruvate kinase isozymes, namely, M1/2 (PKM1 and PKM2), which arise from MXE of PKM exons 9 and 10 and whose alternate expression regulates energy metabolism in cardiac failure (Rees et al., 2015)(Figure S2). We successfully detected the endogenous junction peptides and their synthetic standards, the *a priori* known sequences of the standards providing verification for the identity of the protein isoforms in the endogenous sample. We next assessed 12 undocumented isoform sequences by this method, identifying all 12 synthetic heavy peptides and 6 endogenous peptides in the whole heart lysate (Figure S2). Aside from them being false positives, the remaining 6 sequences may be undetected due to a lack of extensive biological fractionation and potential biological and technical differences between the validation sample and the original dataset (ProteomeXchange: PXD006675). Although further validation of each sequence will require extensive follow-up experiments, overall the targeted MS data corroborate that our method can discover bona fide undocumented peptides in the human proteome.

### Alternative Protein Isoforms Overlap with Disordered Regions

We next asked how noncanonical isoforms may affect protein features. Among the TrEMBL-undocumented peptides we identified was a splice variant of MYBPC3 (Figure 4E). MYBPC3 is a 140-kDa protein that forms an important sarcomeric component to maintain cardiomyocyte structure and is commonly mutated in human congenital hypertrophic cardiomyopathy. We found an SE splice junction peptide, RTDSHEDTGILDFSSLLK, and its sister peptide TDSHEDT-GILDFSSLLK, both of which are repeatedly identified in multiple tissues, including whole heart, left atrium, and left ventricle in our reanalysis. SwissProt catalogs 2 isoform entries for MYBPC3, including the canonical sequence with 1,274 residues and an isoform with 1,273 residues, in which canonical ser408 and lys409 are replaced by a single arginine. Neither entry matches the isoform sequence we identified, which omits the segment SLAGGGRRIS from aa 275–284 encoded by exon 8, and corresponds instead to aa 273–274 (RT-) of the canonical sequence joined to aa 285–300 (-DSHEDTGILDFSSLLK). The noncanonical peptide had not been observed in the peptide repositories PeptideAtlas (housing 1.4M peptides) (Deutsch et al., 2015) or MassIVE-KB (2.3 M peptides) (Wang et al., 2018a) and had no identical match to any sequences of any taxonomy in RefSeq by BLASTP (Boratyn et al., 2013).

**Figure 5.**
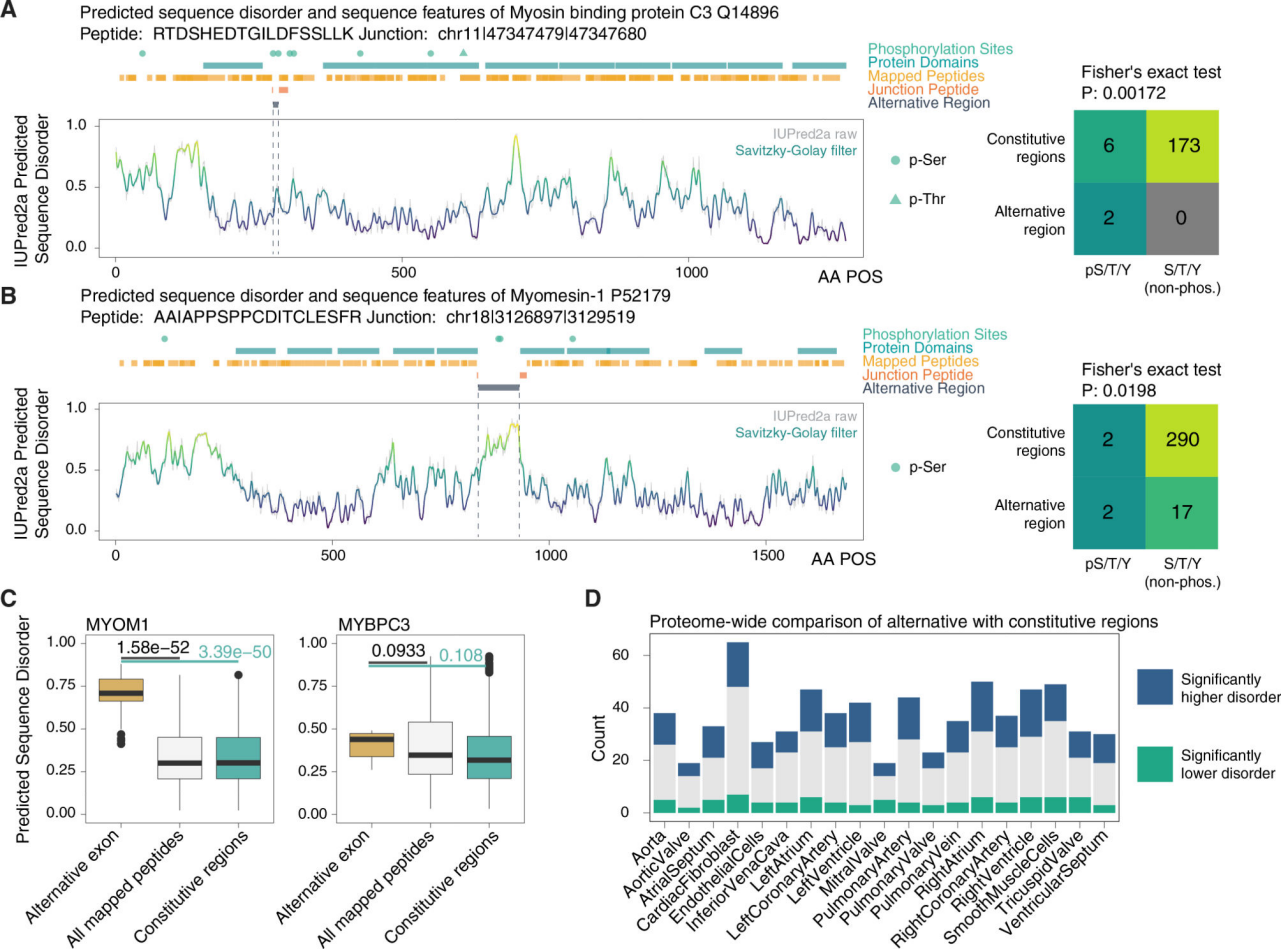
Splice Isoforms Preferentially Overlap with Disordered Protein Regions (A) Sequence features of MYBPC3 highlighting PKA regulatory sites overlapping with the alternative region (residues skipped in the noncanonical isoform) of the protein, and the identified junction peptide spanning the excluded region. Sequence disorder was predicted using IUPred2a and aligned with annotated protein domains and PTM sites on UniProt. (Right) Contingency table on the number of annotated phosphorylation sites and serine/threonine/tyrosine that are not annotated to be phosphorylated in the excluded region versus the rest of the protein sequence. (B) As above, for an MYOM1 SE isoform. (C) Boxplots showing the distribution of sequence disorder in the alternative region (gold) of MYOM1 and MYBPC3 versus all residues uniquely identified by peptide in the database search (white) and the full-length protein sequence excluding the alternative region (green). p value: Mann-Whitney test. Box: 25th–75th percentile; whiskers: 5th-95th percentile. (D) On a proteome scale, alternative regions are significantly associated with higher sequence disorder (blue) over the rest of the protein.

Intriguingly, this SE falls within an unusual region of local disorder nested between two well-defined immunoglobulin (Ig)- like protein domains. We asked whether the excised region overlapped with structural features of interest and found that it is statistically enriched in known phosphorylation site over the entire protein (Fisher’s exact test p value, 0.02). Moreover, the region spans 2 of 3 clustered phosphorylatable serines (S275, S284, and S304) that are key regulatory sites in MYBPC3 targeted by protein kinase A (PKA) (Figure 5A). The phosphorylation of S275, S284, and S304 in MYBPC3 by PKA and other kinases causes the MYBPC3 N-terminal domain to dissociate from myosin heavy chain and, hence, increase cardiac crossbridge formation (Rosas et al., 2015). Mutagenesis replacement of these serines with phosphonegative mimetic alanines in animal models led to hearts with abnormal relaxation velocity but not ejection fraction (Rosas et al., 2015), suggesting the sites may function in diastolic regulation.

Another example of alternative isoforms overlapping with important protein features is found in in myomesin-1 (MYOM1), where an SE spans a region between two fibronectin type III domains with significantly higher sequence disorder than the rest of the protein (Mann-Whitney p value, 3.3e–50) (Figure 5B). Other identified noncanonical sequences also showed a preferential location in disordered regions (Data S2), such that on a proteome scale there is a clear preference for noncanonical isoforms to alter protein regions with heightened sequence disorder (Figure 5D). Taken together, the result provides evidence for one instance where protein alternative isoform overlaps with known regulatory PTM sites and proteome-wide enrichment in disordered protein regions, presenting two potential mechanisms through which AS may regulate proteome function.

### Noncanonical Protein Isoforms Change during Cardiomyocyte Differentiation

**Figure 6.**
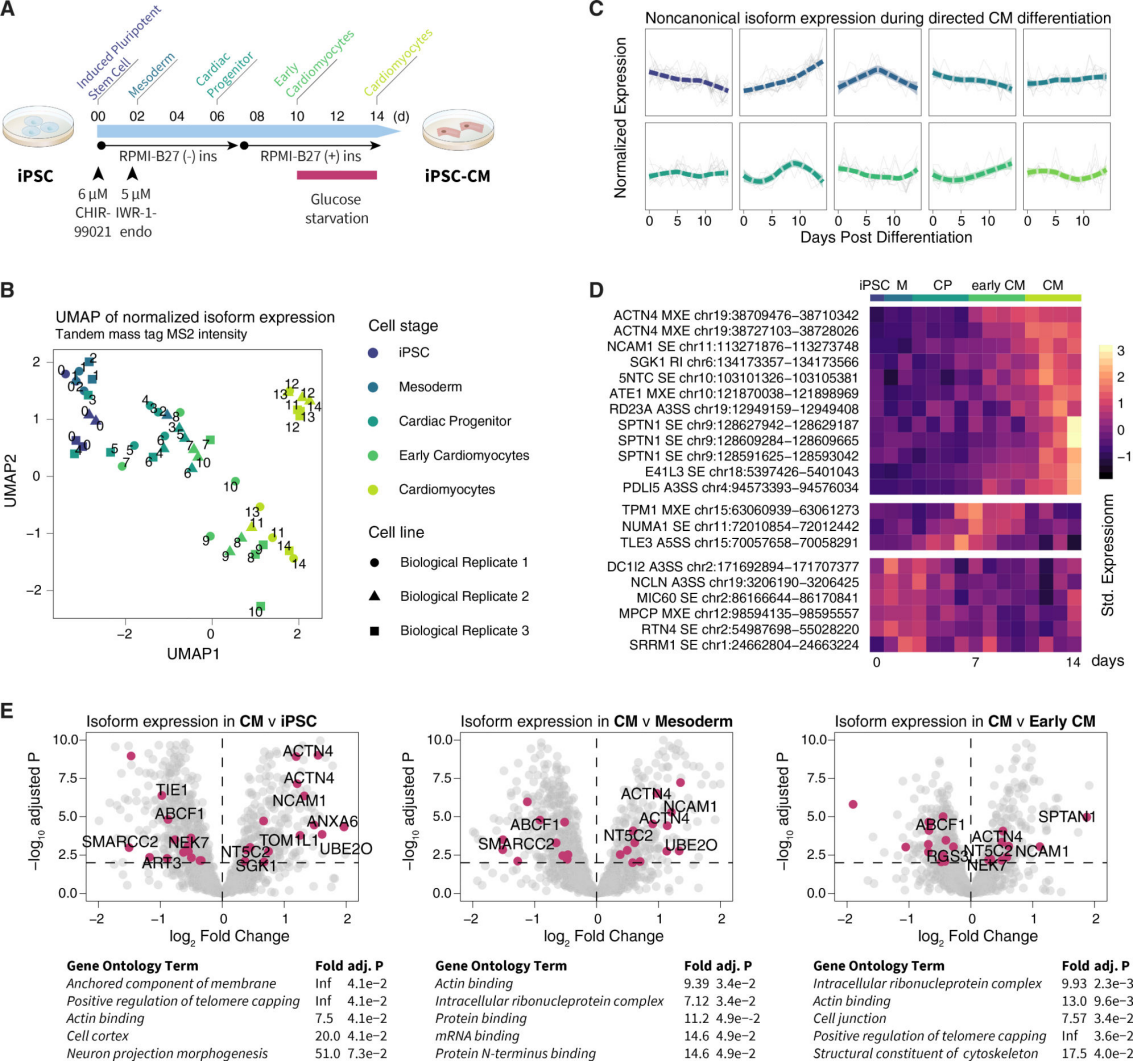
Expression of Protein Isoforms during iPSC Cardiac Differentiation (A) Schematic for human-iPSC-directed cardiac differentiation protocol with annotated stages (iPSC, day 0; mesoderm, day 1–2; cardiac progenitor, day 3–6; early CM, day 7–10; CM, day 11–14). (B) UMAP projection of tandem mass tag intensity shows that total protein expression reflects differentiation stages (n = 3 biological replicates). (C) Hierarchical clustering of noncanonical peptide expression during iPSC-CM differentiation shows diverse temporal behaviors of noncanonical isoforms in each cluster. (D) Heatmap of row-standardized expression of noncanonical isoforms with cell-specific expression during differentiation (n = 3 biological replicates). (E) Volcano plot of logFC versus −log10-adjusted p values comparing protein expression between CM with (left) iPSC, (center) mesoderm, and (right) early CM. Data points, isoforms; magenta, differentially expressed noncanonical isoforms (limmaadj. p ≤ 0.01); differentially expressed isoforms not found in SwissProt are labeled.

We next applied the workflow toward an original MS dataset we generated to examine isoform regulation during human iPSC differentiation into cardiomyocytes (CMs) (Figure 6A). Three human iPSC lines underwent directed cardiac differentiation over 14 days through an established small-molecule-based protocol (Burridge et al., 2014; Lee et al., 2019; Kitani et al., 2019). During the differentiation time course, we harvested cells daily for quantitative shotgun proteomics using 10-plex stable isotope-labeled tandem mass tags (Figure S3). The MS data files are processed as above by using the heart-specific database to identify cardiac-specific protein isoforms. As expected, the iPSC differentiation protocol led to a decrease in cyclins and an increase in cardiac-specific proteins (Figure S3), consistent with bona fide iPSC-CM formation (Kang et al., 1997), whereas the cardiac protein expression profile corresponded to the course of cellular differentiation and different stages of cardiac differentiation (Figure 6B). From 87 quantified noncanonical protein isoforms, including 14 not in the SwissProt canonical/isoform, we observed diverse cell-stage-specific expression patterns for noncanonical protein isoforms (Figure 6C), with some isoforms preferentially expressed in iPSCs, iPSC-CM, or intermediary cell stages (Figure 6D), and an overall enrichment of differentially regulated isoforms in actin binding and ribosomal processes at the pathway level (Figure 6E).

We were particularly interested in protein isoforms with differential expression between day 7 (early CM) and day 14 (CM) stages of iPSC differentiation, as they may be implicated in cardiogenesis and pluripotent-stem-cell-derived CM maturation (Figure 6D; Data S3). For instance, alpha-actinin-4 (ACTN4) is thought to link actin to various subcellular structures. We identified an ACTN4 isoform that is significantly elevated in day 14 CM (log FC, 0.50; adj.P, 4.6e–4), that differs from the canonical isoform in residues 780 to 801, and is not documented on SwissProt. Tropomyosin alpha-1 chain (TPM1) is an actin- binding protein that regulates cardiac muscle contraction. In the data, we found two significantly regulated TPM1 isoforms. The first isoform was significantly downregulated in day 14 iPSC-CM versus day-7 early iPSC-CM (logFC, −0.69; adj.P, 2.0e-3) and differed from the canonical TPM1 sequence by residues 189–212 by MXE, corresponding to an uncharacterized isoform 4 (P09493–4) on SwissProt. The second isoform was significantly upregulated (logFC, 0.73; adj.P, 3.9e–2) and differed from the canonical TPM1 sequence by residues 41–80 by MXE, corresponding to the TPM1 kappa isoform (P09493–6) on SwissProt that was previously found in single-target immunobiological studies to be increased in dilated cardiomyopathy patients (Rajan et al., 2010).

Two isoforms of neural cell adhesion molecule 1 (NCAM1) were upregulated (logFC, 0.58 and 0.52; adj.P, 4.2e–3 and 7.0e–3). NCAM1 is involved in cell adhesion, ventricular wall thickness, and cardiomyopathy. The first quantified isoform is missing residues 354–363 compared to the canonical sequence and, hence, corresponds to isoform 2 on SwissProt (P13591–1). This, in turn, corresponds to N-CAM 140 isoform in the biomedical literature that is known to be expressed in developing hearts (Gordon et al., 1990). At the same time, we quantified a second, unannotated isoform that is longer than the canonical sequence through an insertion of aa 820–1091 of the noncanonical sequence. The inserted sequence is homologous with the mouse full-length N-CAM 180 isoform (P13595; 91.6% identity by ClustalO). A human N-CAM 180 is characterized in single-target cancer studies (Blaheta et al., 2004) but not documented on human SwissProt, which likely excludes it from a number of proteomics studies. Intriguingly, both isoforms share similar expression profiles in iPSC-CM differentiation; thus, it is possible they originate from the same full-length protein with insertion at aa 820–1091 and deletion at 354–363.

We quantified one noncanonical isoform for PDZ and LIM domain protein 5 (PDLIM5) that was upregulated in day 14 CM over day 7 early CM (logFC, 1.11; adj.P, 8.9e–4). PDLIM5 belongs to the PDZ- and LIM-domain-containing protein family and is a Z-disc component of the heart, which has been previously shown by polyclonal antibodies and qPCR to be upregulated and to undergo isoform switches during embryonicstem-cell-CM differentiation (Konze et al., 2017). Our data corroborate the isoform-specific upregulation of PDLIM5 in pluripotent-stem-cell-cardiac differentiation and quantified a differentially regulated isoform that was missing aa 98–206 from the canonical sequence. This is a shared missing region in multiple SwissProt isoforms (Q96HC4–2, −4, −6, and −7) and overlaps multiple PTM sites (Figure S4A). Other identified isoforms in iPSC-CM differentiation also include alternative regions that overlap with disordered regions and known phosphorylation sites, e.g., HNRPD pS80/82/83 and pT87 have been implicated in HNRPD activity (Tolnay et al., 2002) as well as GSPM1 pS445/469/471, indicating that the alteration of PTM site availability may be one functional consequence of AS during cardiac differentiation (Figures S4B and S4C).

**Figure 7.**
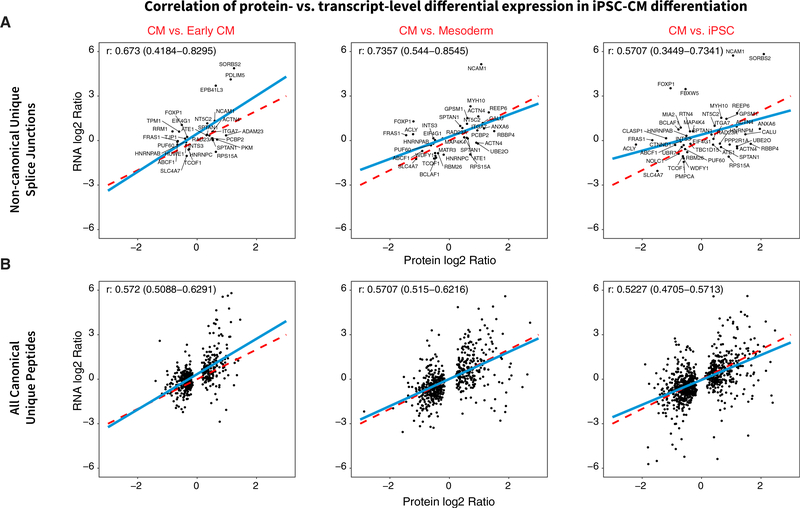
Correlation of Isoform Differential Regulation at Transcript and Protein Levels Scatterplots showing differential expression (logFC) of isoforms at transcript (y axis) versus protein (x axis) levels during iPSC-CM differentiation for noncanonical junction sequences only (A) and all canonical SwissProt unique sequences (B) that were quantified in both RNA-seq and MS and found to be differentially regulated. Protein and transcript isoform logFC show robust positive correlation (Pearson’s r, 0.57–0.74 noncanonical isoforms; 0.52–0.57 canonical). Blue line, best-fit linear regression; red dashed line, unity.

Finally, we explored the creation of cell-type-specific databases directly from the iPSC samples by acquiring deep RNA-seq data (~100 M short reads) from iPSC-CM differentiation. RNA-seq data at day 0 (pluripotent), 2 (mesoderm), 5 (cardiac progenitor/early CM), and 14 (CM) of differentiation show expected decreases in pluripotent markers and increases in CM markers (Figure S5) concomitant with the differential regulation of genes in cardiac development and splicing (Figure S6). In line with previous work (Liu et al., 2017), we observed a robust correlation (Pearson’s correlation coefficient, 0.57–0.74) between transcript and protein level changes among the quantified exon junctions (Figure 7). From the RNA-seq data, we created an iPSC/iPSC-CM sample-specific protein database, which overlaps only partially with human heart database and contains cell-type-specific translated junctions (Figure S7). Among genes with multiple quantified isoforms from the iPSC-specific database, the majority show concordant expression patterns during differentiation, but we also observed isoform-specific changes (Figure S7). For example, an SE in the respiratory complex I protein NDUFV3 corresponded to 2 recently described isoforms (short and long NDUFV3) (Bridges et al., 2017), for which we saw different expression levels in iPSC that converged during differentiation. Taken together, the results support the applicability of the method to extending isoform quantification studies into other dynamic processes, including cellular differentiation and development.

## DISCUSSION

AS is widely implicated in development, aging, and diseases (van den Hoogenhof et al., 2016; Lee and Rio, 2015), but a fuller understanding requires knowing how isoforms alter protein structure and functions (Li et al., 2017). Only a minority of expressed transcripts have the potential to be translated (Hao et al., 2015), whereas the rest may be removed by nonsense-mediated decay (NMD) or co-translational proteolysis (Weatheritt et al., 2016). The ability to empirically detect AS protein isoforms in a tissue is, thus, a critical step toward elucidating their molecular and cellular functions.

We present here a splice-junction-centric approach to create size-restricted databases to guide protein isoform identifications. The generation of accurate protein sequence databases is an important step in avoiding inflation of false positives during database search and entails finding the set of isoform peptides that exists in a particular sample and is detectable by the MS experimental design. Recent studies have used high-throughput sequencing reads as a template to identify variant protein sequences (Cifani et al., 2018; Carlyle et al., 2018; Zickmann and Renard, 2015; Verbruggen et al., 2019; Mertins et al., 2016; Wang et al., 2019). Our approach builds on prior work and is distinguished by the selection for splice junction pairs in AS events with appreciable RNA-seq read counts. We also enforce one translatable frame for each junction by picking the canonical annotated frame or a frame that does not lead to PTC during *in silico* translation, avoiding redundant entries from 3- or 6-frame translation approaches (Sheynkman et al., 2013; Zickmann and Renard, 2015; Wang and Zhang, 2013). The custom databases here contain only 6.3%−17.0% as many sequences as RefSeq but, nevertheless, enable the recovery of noncanonical isoforms across tissues, including sequences not found in TrEMBL or RefSeq.

Empirical evidence on how AS rewires proteomes has emerged slowly, with recent reports emphasizing interactomes (Yang et al., 2016) and overall protein abundance (Liu et al., 2017). We found that many identified isoforms differ from canonical sequences by excluding residues that overlap with disordered regions and phosphorylation sites. A discovered MYBPC3 isoform differs from the canonical sequence by only 10 of 1,274 residues but is located at a crucial phosphorylation region known to modulate diastolic functions of the heart, suggesting a potential manner through which it can impact protein function. The observation that most alternative exons do not alter stable protein domains (Barbosa-Morais et al., 2012; Buljan et al., 2012) has been cited as evidence against their functionality (Tress et al., 2017a, 2017b). However, unstructured regions can also regulate protein function such as by forming protein-protein interaction surfaces (Ellis et al., 2012), governing phase separation and membraneless organelles (Uversky, 2017; Harmon et al., 2017), and allosterically modulating remote catalytic domains (Keul et al., 2018; Ferreon et al., 2013). Taken together, our results support that on a proteome scale AS may influence protein function by (1) rewiring flexible regions linking stable protein domains and (2) provide a separate PTM control mechanism by toggling the binary presence/absence of modifiable residues. An intersection between AS and sequence disorder or PTMs has been hypothesized (Zhou et al., 2018) and is consistent with the notion that AS rewires protein interactomes (Yang et al., 2016; Ellis et al., 2012).

Among uniquely mapped distinct peptide sequences, we found that 1 %–3% mapped to noncanonical isoforms per tissue (Figure 2B). This proportion is consistent with most genes having one dominant principal form but does not rule out spatially and temporally regulated alternative forms with biological function. MXE is overrepresented among detectable isoforms in our workflow, which may be due to a bias in reading frame conservation. Additional translated splice junctions likely remain to be discovered as technologies continue to develop. Some AS junction peptides now appear in multiple custom-translated forms due to the combinatorial redundancy in exon junctions in short-read RNA-seq, rendering them ambiguous in protein assignment. The inability of bottom-up proteomics to identify full-length proteins also impedes accurate isoform quantification. The adoption of long-read RNA-seq and middle-down/top-down proteomics will likely mitigate these limitations. Finally, continued refinements in computational prediction of translated transcripts can improve isoform identification; e.g., some PTCs near the end of the transcript may not cause NMD, calling for better NMD prediction from frameshift sequences.

In summary, we describe an approach to create concise AS variant databases for protein isoform analysis. The method is implemented in an open source software tool (https://github.com/ed-lau/jcast) that can be applied to other RNA-seq and MS data. Although discovered isoform peptides will need to be validated by orthogonal approaches, the method here may avail understanding of the biological role of AS both in the human proteome and the proteomes of non-human organisms where splicing remains substantially less documented.

## STAR★METHODS

### LEAD CONTACT AND MATERIALS AVAILABILITY

This study did not generate new unique reagents. Further information and requests for resources and reagents should be directed to and will be fulfilled by the Lead Contact, Maggie P. Y. Lam (maggie.lam@cuanschutz.edu).

### EXPERIMENTAL MODEL AND SUBJECT DETAILS

Human iPSC lines were acquired from publicly available cryopreserved stocks in the Stanford Cardiovascular Institute Biobank. Human iPSCs (2 male and 1 female lines) were expanded in monolayer in GIBCO Essential 8 medium (Thermo) on a Matrigel matrix (Corning). Human iPSC differentiation into CM was performed on three individual donor lines using an established small-molecule Wnt-activation/inhibition protocol yielding 95% pure TNNT2+ CM (Burridge et al., 2014; Lee et al., 2019; Kitani et al., 2019). Briefly, iPSC cultures at ~90% confluence in 6-well-plates were treated with 6 μM CHIR-99021 (SelleckChem) in RPM11640 medium supplemented with B27 supplements (Thermo Fisher Scientific) for 2 days to induce mesoderm specification, allowed to recover 1 day, then treated with 5 μM IWR-1-endo (SelleckChem) for 2 days for cardiac specification. On day 7, the culture medium was changed to RPMI-B27 + insulin, and the cells were glucose-starved on day 10 to day 14. Cells were harvested daily at day 0 to day 14 post-differentiation by dissociation using TrypLE select 10x (Thermo Fisher Scientific) and pelleted by centrifugation (200 × g, ambient temperature, 5 min).

### METHOD DETAILS

#### Public RNA sequencing and mass spectrometry datasets

RNA sequencing datasets were retrieved from ENCODE at the following accessions: heart (ENCSR436QDU, ENCSR391VGU), liver (ENCSR226KML, ENCSR504QMK), lung (ENCSR425RGZ, ENCSR406SAW), pancreas (ENCSR671IYC, ENCSR586SYA), adrenal gland (ENCSR801MKV, ENCSR754WLW), transverse colon (ENCSR800WIY, ENCSR403SZN), ovary (ENCSR841ADZ, ENCSR042-GYH), esophagus (ENCSR098BUF, ENCSR750ETS), testis (ENCSR029KNZ, ENCSR344MQK), prostate (ENCSR495HDM, ENCSR701TST), spleen (ENCSR194HVU, ENCSR900SGE), and thyroid (ENCSR113HQM, ENCSR017ZLM). RNA sequencing data from at least two biological replicates from each tissue were used. All data were 101nt paired-end total RNA sequencing generated on an Illumina Hi-Seq 2500 sequencer and passed ENCODE quality control (ENCODE Project Consortium, 2012). RNA sequencing read [.fastq] files were manually retrieved on 2017–11-12. Proteomic datasets in Thermo [.raw] format were retrieved from ProteomeXchange/PRIDE (Deutsch et al., 2017) at the following accessions: “A draft map of the human proteome” (ProteoeX-change: PXD000561) (Kim et al., 2014) generated on Thermo Orbitrap Velos and Orbitrap Elite mass spectrometers with FT/FT; “Region and cell-type resolved quantitative proteomic map of the human heart and its application to atrial fibrillation” (PXD006675) (Doll et al., 2017) generated on a Thermo Q-Exactive HF mass spectrometer; “Human testis off-line LC-MS/MS” (PXD009737) (Sun et al., 2018) generated on a Thermo Q-Exactive HF-X mass spectrometer; “Proteomic analysis of human liver reference material” (PXD009021) generated on a Thermo Fusion Lumos mass spectrometer in FT/FT mode; and “A deep proteome and transcriptome abundance atlas of 29 healthy human tissues” (PXD10151) generated on a Thermo Q-Exactive Plus mass spectrometry (Wang et al., 2019).

#### RNA data processing and database generation

To align the retrieved RNA sequencing data, we used STAR v.2.5.0a (Ballouz et al., 2018; Dobin and Gingeras, 2016) on a Linux 4.10.0–32-generic Ubuntu x86_64 workstation. We mapped .fastq sequences to Ensembl GRCh38.89 STAR indexed genomes with Ensembl .gtf annotations (–sjdbGTFfile GRCh38.89.gtf–sjdbOverhang 100). To extract splice junctions from mapped reads and compare splice levels across biological replicates, we used rMATS-Turbo v.0.1 (Shen et al., 2014) on the mapped bam files with the following options (–readLength 101–anchorLength 1). We implemented a custom script written in-house in Python v.3.6.1, which tabulates the rMATS results on AS events from each tissue and filters out ineligible splice pairs by virtue of read count threshold or significant inter-sample differences. Junctions are filtered by the minimal excluded junction read count of all biological replicates (rMATS *SJC)* for a particular junction *j* for a tissue *t* such that transcript level *SJCj, t* is above a detectability threshold *SJC*_*j,t*_ ≥ *θ*_*t*_, which is estimated by a mixture Gaussian model of excluded junction read counts based on the specific RNA sequencing dataset for the tissue. In addition, we assume that the isoform is reliably observed across multiple runs, employing the statistical model implemented in rMATS to exclude significantly differential splice junctions at p ≤ 0.01 (Shen et al., 2014).

The script next retrieves nucleotide sequences from each splice pair based on the recorded genomic coordinates using the Ensembl REST web application programming interface, and attempts to identify the appropriate translation frames, transcription start sites, and transcription end sites of each splice pair from the Ensembl GRCh38.89 annotation GTF file based on the upstream exon. The retrieved qualifying nucleotide sequences are further translated into amino acid sequences using the annotated phase and frame. Peptides are selected for inclusion if they fulfill one of the following sequential considerations: (i) they are translated in-frame by the GTF-annotated translation frame in Ensembl GRCh38.89 GTF successfully without encountering a frameshift or PTC; or (ii) one of the spliced pair junctions encountered a frameshift event using the GTF-annotated frame but both are translated without PTC; (iii) they are translated without PTC using a single translation frame different from the GTF-annotated frame; (iv) in rare occasions, one of the two junctions but not the other encountered a PTC. Finally, all translated peptides are required to be stitchable back to the SwissProt canonical sequences retrieved via the gene name using a 10-amino-acid overhang. Orphan peptides that are translated but not stitchable back to SwissProt are discarded from the analysis. The translated databases used for analysis are available in Data S4.

As the conventional method to generate *de novo* databases, we performed three-frame translation of assembled transcripts using prior published workflows in the R package customProDB (Wang and Zhang, 2013). Briefly a bowtie2 index was generated for GRCh38 as specified by the customProDB package instructions. Tophat2 (v.2.1.1) (Kim et al., 2013) was then used to analyze identical human heart ENCODE RNA-seq data as above and stringtie (v.1.3.5) (Pertea et al., 2015) was used on the topcoat output. The stringtie output was piped to customProDB to build a custom database, which was then used for database search as described below.

#### Mass spectrometry database search and analysis

Mass spectrometry raw spectrum files were converted to open-source [.mzML] formats using ProteoWizard msconvert v.3.0.11392 (Adusumilli and Mallick, 2017) with the following options (–filter “peakPicking vendor”). Database search against custom databases were performed using the SEQUEST algorithm implemented in Comet v.2017.01 rev.0 (Eng et al., 2015) with the following options (–peptide_mass_tolerance 10-peptide_mass_unit 2-isotope_error 2-allowed_missed_cleavage 2-num_enzyme_termini 1-fragment_bin_tol 0.02). Conventional settings for other Comet parameters were used and a reverse decoy database was generated from the custom database for each search for FDR estimation. Static cysteine carboxyamidomethylation (C +57.021464 Da; Unimod accession #4) modification was specified. Tryptic and semi-tryptic peptides within a 10-ppm parent mass window surrounding the candidate precursor mass were searched, allowing up to 2 miscleavage events.

Peptide spectrum match data were filtered and target and decoy sequence matches were re-ranked using the semi-supervised learning method implemented in Percolator (The et al., 2016) in the Crux v.3.0 Macintosh binary distribution (McIlwain et al., 2014) with the following options (–protein T–fido-empirical-protein-q T–decoy-prefix DECOY_). Peptides with Percolator q value ≤ 0.01 are considered to be confidently identified. Mass tolerant open search comparison was performed using MSFragger (Kong et al., 2017) using standard parameters with lower mass tolerance −200 Da and upper mass tolerance was +400 Da against the UniProt TrEMBL human database (accessed 2019–02-08), followed by Percolator filtering as above.

#### Human iPSC RNA-seq and labeled shotgun proteomics

For RNA-seq, total cellular RNA from day 0, 2, 7, and 14 post-differentiation were extracted by 300 μL TRIzol/chloroform per ~1e6 cells, followed by solid-phase extraction using RNeasy mini columns (QIAgen) according to the manufacturer’s protocol. Purified RNA was eluted in 50 μL of RNase-free water and the yield quantity and quality were assessed by fragment electrophoresis on an Agilent Bioanalyzer with the RNA Integrity Number (RIN) of all samples used for sequencing above 9.0. RNA sequencing was performed on an Illumina Hi-Seq 4000 instrument to acquire paired-end 150-nt reads up to a read-depth of 31.1G to 41.7G clean bases (Novogene). The RNA sequencing data were processed identically to the public datasets above to create a custom FASTA database containing the combined human alternative splice junctions from both day 0 and day 14 time points.

Cell lysate proteins from each daily iPSC time point (n = 3 biological replicates) were extracted by commercial RIPA or M-Per tissue lysis buffer (Thermo Fisher Scientific) with 1x Thermo Halt protease/phosphatase inhibitor followed by brief pulses of sonication with typically 6 pulses at 20% amplitude followed by 5 s cooldown on ice. Total protein extracts for each sample were quantified by bicinchoninic acid assays and 150 μg proteins were digested on 10-kDa MWCO polyethersulfone filters (Thermo Fisher Scientific). Samples were washed with 8 M urea, buffer-exchanged with triethylammonium bicarbonate (100 mM, 100 μL), reduced with tris(2-carboxyethyl)phosphine (3 μL of 0.5 M, mM, 55 °C, 30 min) and alkylated with iodoacetamide (7.5 μL of 375 mM, ambient temperature, 30 min). Proteins were digested on-filter (16 hr, 37°C) with sequencing-grade modified trypsin (50:1, Pierce Trypsin Protease, MS Grade). Proteolytic digests were labeled with 10-plex tandem mass tags (Thermo Fisher Scientific) at ambient temperature with 600 rpm shaking for 2 hr. Label assignment was randomized using a random number generator. Labeling was quenched with 5% hydroxylamine following manufacturer’s protocol.

Liquid chromatography-tandem mass spectrometry was performed on peptides fractionated into 6 fractions using pH-10 reversed-phase spin columns (Thermo Pierce). Second-dimension liquid chromatography was performed using a single Easy-nLC 1000 nanoflow ultrahigh-pressure liquid chromatography (UPLC) system on an EasySpray C18 column (PepMap, 3-μm particle, 100-Å pore; 75 μm × 150 mm; Thermo Fisher Scientific) in 120-min in a pH-2 reversed-phase gradient. The nano-UPLC was run at 300 nL/min with the gradient of 0 to 105 min, 0 to 40%B, 105 to 110 min, 40 to 70%B, 110 to 115 min, 70 to 100%B, hold for 5 min, with solvent B being 80% v/v acetonitrile and 0.1% v/v formic acid. Mass spectrometry was performed using a Q-Exactive HF high-resolution Orbitrap mass spectrometer (Thermo Fisher Scientific) coupled to the nano-UPLC by an EasySpray interface. Typical MS1 survey scan was acquired at 60,000 resolving power in positive polarity in profile mode from 300 to 1650 m/z, lock mass, dynamic exclusion of 30 s, maximum injection time of 20 msec, and automatic gain control target of 3e6. MS2 scans were acquired on the top 15 ions with monoisotopic peak selection at 60,000 resolution, automatic gain control target of 2e5, maximum injection time of 110 ms, and isolation window of 1.4 m/z, with typical normalized collision-induced dissociation energy of 32 or stepped normalized collision-induced dissociation energy (NCE) of 27, 30, and 32.

#### Parallel reaction monitoring targeted mass spectrometry

For targeted mass spectrometry, 200 μg of adult whole normal human heart tissue lysate (Novus Biologicals NB820–59217) was digested with 5 μg trypsin as described above and pre-fractionated into 10 fractions using pH-10 reversed-phase spin columns (Thermo Pierce). A total of ~3 μg pre-digest equivalent/~1.5 μg estimated actual heart lysate endogenous peptides from each fraction was co-injected with ~1 pmol total of crude unmodified synthetic peptides labeled with heavy N terminus lysine or arginine (Thermo Fisher Scientific) (Figure S5). Targeted mass spectrometry data were acquired on a Q-Exactive HF high-resolution Orbitrap mass spectrometer (Thermo Fisher Scientific) in parallel reaction monitoring data acquisition mode with the following instrument settings: AGC target 2.0e+5 for PRM, NCE 24 and 27; maximum IT 110 msec; loop count of 10; isolation window 1.2 to 1.4 m/z, isolation window offset 0.5 to 0.6 m/z; resolution (MS1 and PRM) 60,000. The LC gradient used was 0 to 75 min: 0% to 40%B; 75 to 80 min: 40% to 70% B; 80 to 85 min: 70 to 100% B; 85 to 90 min: 100% B hold; at 300 nL/min. Target ion accurate mass and retention time acquisition table is in Figure S5.

### QUANTIFICATION AND STATISTICAL ANALYSIS

To quantify peptide intensity in the iPSC data, tandem mass tag intensity was corrected by the isotope contamination matrix supplied by the manufacturer, tag intensity in each 10-plex experiment was column normalized, row-normalized by two pooled reference tags per experimental block, then normalized by trimmed means of m values in edgeR (Robinson and Oshlack, 2010) and log-transformed for across-sample comparison. Non-unique peptides as well as peptides confidently identified at fewer than three independent tandem mass tag experiment blocks were discarded. Statistical analysis of differential expression was performed using the moderated t test and empirical Bayes model in limma (v.3.34.3) in R/Bioconductor (v.3.6) (Ritchie et al., 2015) using discrete developmental stages as factors. Proteins with limma adjusted P value (FDR) ≤ 0.01 in each comparison are considered to show evidence for statistically significant differential regulation.

Data statistical analysis and visualization were performed in R v.3.4.4 (2018–03-15 release) or above on x86_64-apple-darwin15.6.0 (64-bit) with the aid of Bioconductor v.3.6 (Huber et al., 2015), and MSnbase v.2.4.2 (Gatto and Lilley, 2012). Gene Ontology terms were used for protein functional annotations (The Gene Ontology Consortium, 2017). Protein sequence features were retrieved from UniProt (The UniProt Consortium, 2018). Protein sequence disorder prediction was performed using IUPred2A (Mészáros et al., 2018). Fisher’s exact test was used to assess enrichment in phosphorylation sites in isoform excluded regions and in the enrichment of Gene Ontology terms in quantified proteins. Sequence occurrence of identified peptide sequences in UniProt SwissProt or TrEMBL human (9606) sequences (retrieved 2019–02-08) (The UniProt Consortium, 2018) or RefSeq (retrieved 2019–02-07) (Pruitt et al., 2014) with 0 or more mismatch tolerance were assessed using the BioStrings v.3.7.0 package. Dimension reduction of human iPSC tandem mass tag data was performed using the uniform manifold approximation and projection (UMAP) method as described (Becht et al., 2019).

### DATA AND CODE AVAILABILITY

Mass spectrometry data on human iPSCs and human whole heart lysate have been deposited to the ProteomeXchange Consortium via the PRIDE partner repository with the dataset identifiers ProteomeXchange: PXD013426 (human iPSC shotgun proteomics) and PXD015544 (human heart targeted mass spectrometry). RNA sequencing data have been deposited to NCBI GEO (GEO: GSE137920). Public RNA-seq and mass spectrometry data used in this study are available on ENCODE and ProteomeXchange. The Python software and source code for translation of AS sequences and generation of custom databases is available at https://github.com/ed-lau/jcast.

## Supplementary Material

1

2

3

4

5

6

## Figures and Tables

**KEY RESOURCES TABLE:** 

REAGENT or RESOURCE	SOURCE	IDENTIFIER
Biological Samples		
Human whole heart tissue lysate	Novus Biologicals	NB820–59217
Chemicals, Peptides, and Recombinant Proteins		
Synthetic Peptides LAPITSDPTEATAVGAVEASFK[13C(6)15N(2)]	Thermo Fisher Scientific Standard Peptides	https://www.thermofisher.com/us/en/home/life-science/protein-biology/peptides-proteins/custom-peptide-synthesis-services.html
CLAAALIVLTESGR[13C(6)15N(4)]		
AAIAPPSPPCDITCLESFR[13C(6)15N(4)]		
APHVEFLRPLTDLQVR[13C(6)15N(4)]		
QCQGQAAQEAAGGGR[13C(6)15N(4)]		
DSGLVGLAVCNTPHER[13C(6)15N(4)]		
VGPVSAVGVTAPGK[13C(6)15N(2)]		
DSEGDTPSLINWPSSK[13C(6)15N(2)]		
LLGADSATVFNIQEPEEETANQIYWFK		
VLDIANVLFHLEQVEHPQR[13C(6)15N(4)]		
YSTGSDSASFPHTTPSMCLNPDLEGPPLELTK[13C(6)15N(2)]		
AITQLLCETEGR[13C(6)15N(4)]		
RTDSHEDTGILDFSSLLK[13C(6)15N(2)]		
ANLSSSTGNVEDSFEGFR[13C(6)15N(4)		
Deposited Data		
Quantitative shotgun proteomics data on human iPSC-cardiomyocyte differentiation	This Study	ProteomeXchange PXD013426
Targeted mass spectrometry data on isoform peptide verification	This Study	ProteomeXchange PXD015544
RNA sequencing data on human iPSC-cardiomyocyte differentiation	This Study	NCBI GEO GSE137920
Experimental Models: Cell Lines		
Human induced pluripotent stem cells	Stanford Cardiovascular Institute Biobank (Burridge et al., 2014; Lee et al., 2019; Kitani et al., 2019).	N/A
Software and Algorithms		
GRCh38.89	Ensembl	http://www.ensembl.org/
STAR v.2.5.0a	Ballouz et al., 2018	https://github.com/alexdobin/STAR
rMATS-Turbo v.0.1	Shen et al., 2014	rnaseq-mats.sourceforge.net
limma v.3.34.3	Ritchie et al., 2015	http://bioconductor.org
Comet v.2017.01 rev.0	Eng et al., 2015	http://comet-ms.sourceforge.net
ProteoWizard msconvert v.3.0.11392	Adusumilli and Mallick, 2017	http://proteowizard.sourceforge.net
Percolator v.3.0	The et al., 2016	http://crux.ms
MSFragger v.20171106	Kong et al., 2017	https://msfragger.nesvilab.org
R v.3.4.4	The R Foundation	https://www.r-project.org/
JCAST v.0.1.0	This study	https://github.com/ed-lau/jcast
Other		
RNA sequencing data on human heart	ENCODE (ENCODE Project Consortium, 2012)	ENCSR436QDU ENCSR391VGU
RNA sequencing data on human liver	ENCODE (ENCODE Project Consortium, 2012)	ENCSR226KML ENCSR504QMK
RNA sequencing data on human lung	ENCODE (ENCODE Project Consortium, 2012)	ENCSR425RGZ ENCSR406SAW
RNA sequencing data on human pancreas	ENCODE (ENCODE Project Consortium, 2012)	ENCSR671IYC
		ENCSR586SYA
RNA sequencing data on human adrenal gland	ENCODE (ENCODE Project Consortium, 2012)	ENCSR801MKV ENCSR754WLW
RNA sequencing data on human transverse colon	ENCODE (ENCODE Project Consortium, 2012)	ENCSR800WIY ENCSR403SZN
RNA sequencing data on human ovary	ENCODE (ENCODE Project Consortium, 2012)	ENCSR841ADZ ENCSR042GYH
RNA sequencing data on human esophagus	ENCODE (ENCODE Project Consortium, 2012)	ENCSR098BUF ENCSR750ETS
RNA sequencing data on human testis	ENCODE (ENCODE Project Consortium, 2012)	ENCSR029KNZ
		ENCSR344MQK
RNA sequencing data on human prostate	ENCODE (ENCODE Project Consortium, 2012)	ENCSR495HDM ENCSR701TST
RNA sequencing data on human spleen	ENCODE (ENCODE Project Consortium, 2012)	ENCSR194HVU ENCSR900SGE
RNA sequencing data on human thyroid	ENCODE (ENCODE Project Consortium, 2012)	ENCSR113HQM ENCSR017ZLM
Mass spectrometry data on human heart, liver, lung, pancreas, adrenal gland, colon, ovary, esophagus, testis, prostate	ProteomeXchange (Kim et al., 2014)	PXD000561
Mass spectrometry data on human liver	ProteomeXchange	PXD009021
Mass spectrometry data on human testis	ProteomeXchange (Sun et al., 2018)	PXD009737
Mass spectrometry data on human heart	ProteomeXchange (Doll et al., 2017)	PXD006675
Mass spectrometry data on human heart, liver, lung, pancreas, adrenal gland, colon, ovary, esophagus, testis, prostate, spleen, thyroid	ProteomeXchange (Wang et al., 2019)	PXD010154

## Highlights

- Integrated omics method identifies non-canonical and undocumented protein isoforms
- Widespread isoform shifts during human iPSC cardiomyocyte differentiation
- Unidentified protein isoforms recoverable from ~80 million public mass spectra
- Isoform sequences overlap with intrinsically disordered regions and PTM sites
