# Supplementary material for: Splice-Junction-Based Mapping of Alternative Isoforms in the Human Proteome
Source: Cell Rep. Author manuscript; Available in PMC 2020 Jan 15. (PMC6961840; doi:10.1016/j.celrep.2019.11.026)
Supplement: 1 [file NIHMS1546469-supplement-1.pdf]

Cell Reports, Volume *29*

Supplemental Information

Splice-Junction-Based Mapping of Alternative Isoforms in the Human Proteome

Edward Lau, Yu Han, Damon R. Williams, Cody T. Thomas, Rajani Shrestha, Joseph C. Wu, and Maggie P.Y. Lam

Supplemental Information

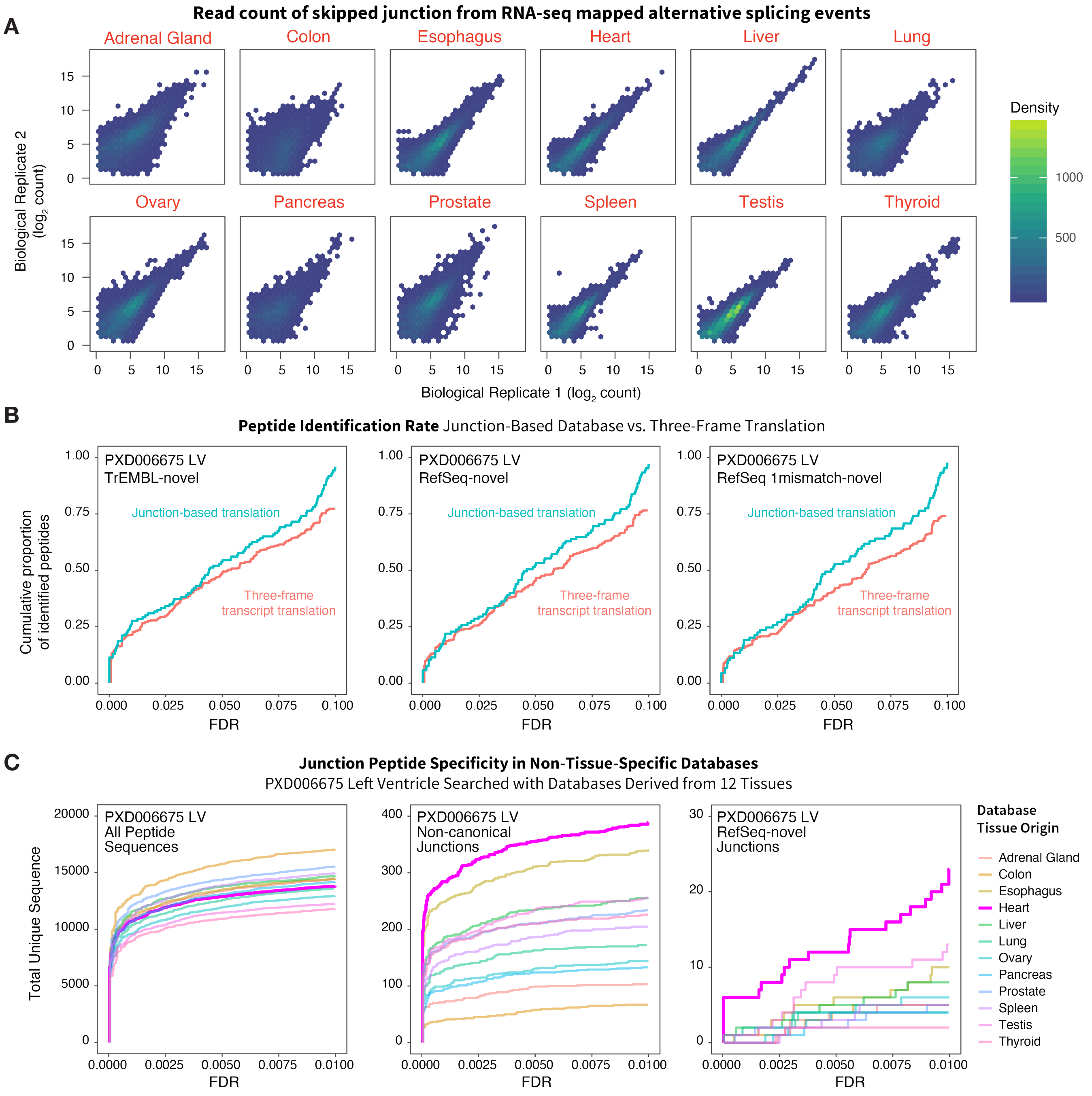

Figure S1. Construction and benchmarking of custom protein sequence databases. Related to Figure 1 and Figure 2.

A. Density scatter plot showing the distribution of log2 read counts in the skipped junction read counts of all identified alternative splicing events in replicate RNA-seq data. **B.** Line chart showing cumulative proportion of identified peptides for junction-based (blue) methods vs. three-frame translation of transcripts (red), both methods analyzing identical RNA-seq and proteomics data. Panels show identification of peptide sequences not found in (left) TrEMBL, (middle) RefSeq, or (right) RefSeq following 1 mismatch allowance left ventricle proteomics data (PXD006675). Compared to conventional three-frame translation of transcripts identified in RNA-seq data, junction-based translation of tissue-specific databases contain more precise peptides as reflected by consistently greater proportions of identifiable peptides at 0 to 10% false discovery rate (FDR). FDR is determined by Percolator q-value in decoy database search. **C.** Specificity of peptide identification in a left ventricle tissue dataset (PXD006675) when searched against custom databases generated using RNA-seq data derived from the heart or

11 other mismatched human tissues. The heart databases supported the identification of average number of total peptide sequences (left) but greater number of non-canonical isoform junction peptides (middle) and peptide sequence candidates (right) at 1% FDR, corroborating the translated junctions are specific to the tissue investigated.

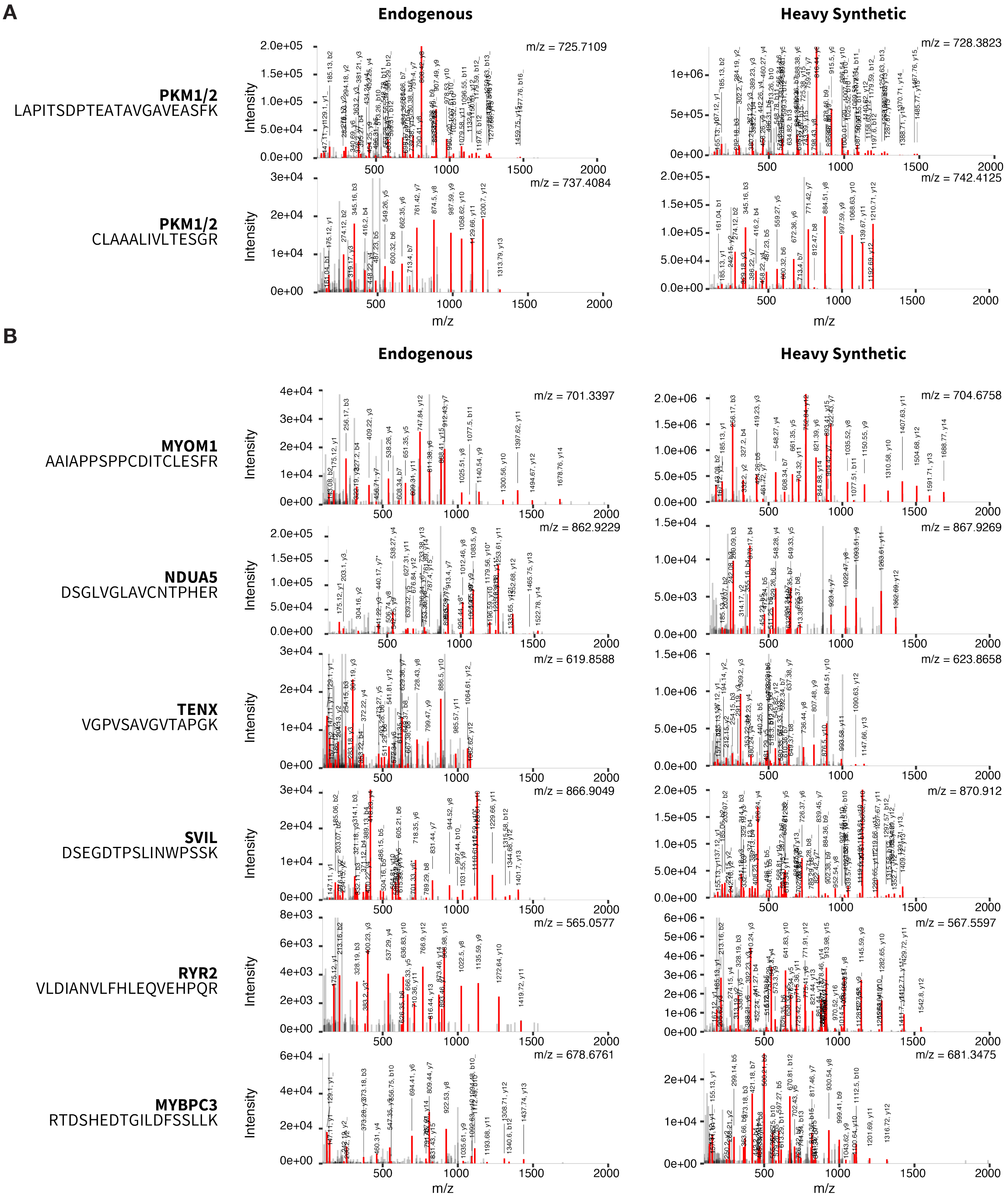

Figure S2. Targeted mass spectrometry validation of junction peptides. Related to Figure 4, Table S1, and STAR Methods.

A. Two junction peptides from the pyruvate kinase M1/M2 (PKM) isozymes known to be detectable in the heart at the protein level were used as positive controls to optimize the targeted validation experiments. The endogenous (light) peptides were readily re-identified in human whole heart tissue lysate in the PRM validation experiment alongside their heavy-labeled synthetic peptide counterpart with comparable fragmentation patterns in FT/FT MS2. **B.** MS/MS fragmentation spectra

acquired in parallel reaction monitoring (PRM) mass spectrometry of selected endogenous (left) and co-injected heavy-labeled synthetic (right) peptides in the validation experiment in an independent biological sample (see Table S1). In addition to being confidently re-identified under identical data analysis workflows, four of the endogenous peptides (MYOM1, TENX, SVIL, RYR2) showed excellent agreement with heavy synthetic standards upon manual inspection whereas two (NDUA5, MYBPC3) showed possible matches. On the mass spectra, red peaks denote matches to peptide ion fragments of the assigned peptide. Peak labels: fragment m/z, fragment b- or y- ion match at +1 or +2 charge; _: water loss; *: ammonium loss; upper right: parent ion m/z. Peptide fragment mass values were calculated from peptide sequences with cysteine carbamidomethyl modification. Spectrum peak labels in the figure were generated automatically using the MSnbase package on R/Bioconductor.

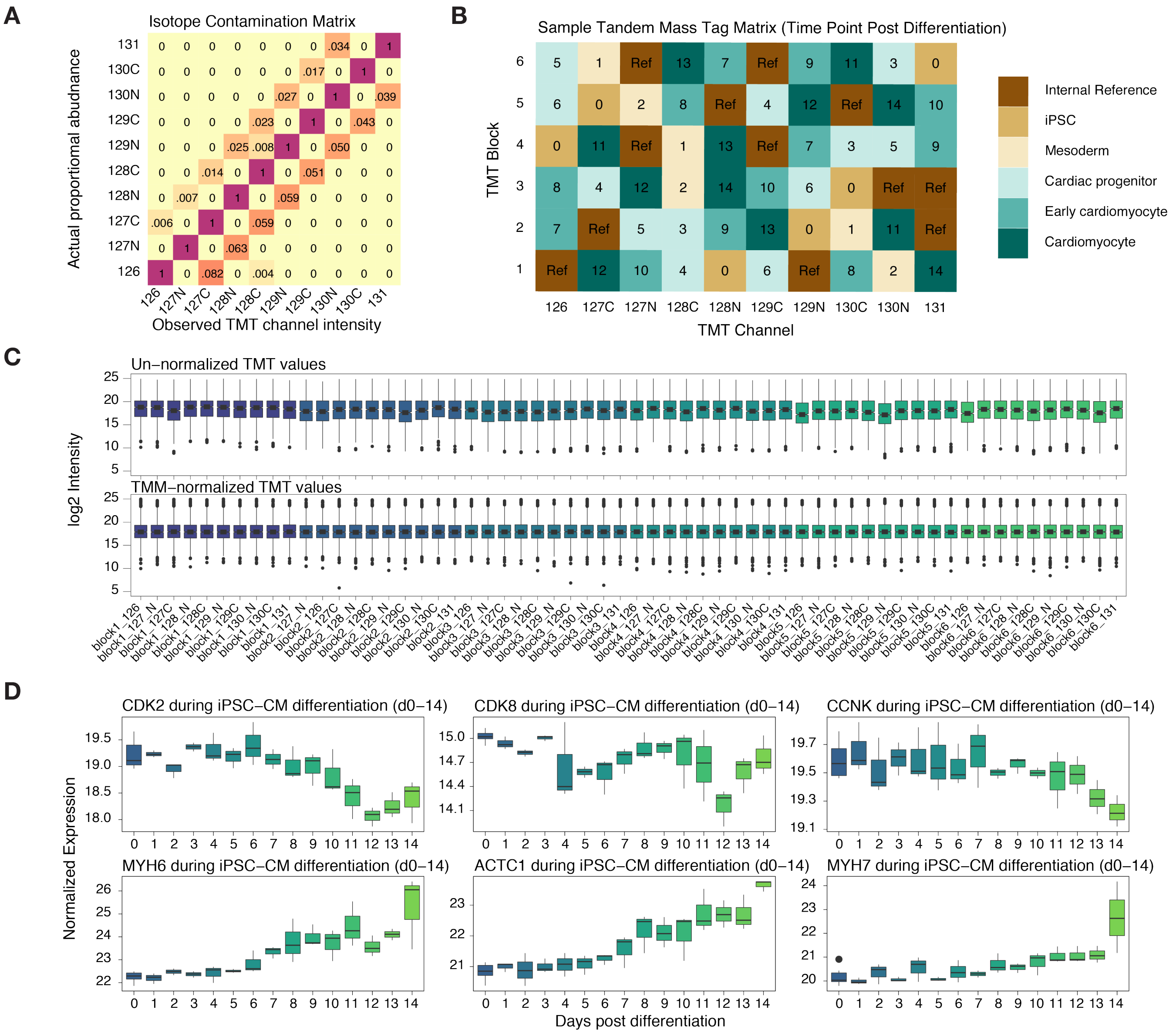

Figure S3. Tandem mass tag labeling of iPSC proteins. Related to Figure 6.

A. Isotope cross-contamination matrix for calculating actual channel intensity from observed tag intensity in tandem mass tag (TMT) data. **B.** Randomized sample assignment and internal references for TMT channels. Labels denote time point (days) post differentiation or pooled internal reference (Ref). Fill color denotes discretized differentiation stages. **C.** Distribution of unnormalized (upper) and column- and trimmed mean of M value (TMM)- normalized (lower) TMT intensity for each channel in each experimental block used for differential expression analysis. **D.** (Top) Directed differentiation of human iPSCs into cardiomyocytes from day 0 to day 14 is associated with a decrease in cell cycle proteins including cyclin-dependent kinases 2 and 8 and cyclin K. (Bottom) Increases in cardiomyocyte-specific proteins including myosin heavy chain 6 and 7 as well as cardiac muscle alpha actin, consistent with cardiomyocyte differentiation. Taken together, the proteomic changes in cell proliferation and cardiomyocyte markers are consistent with transcript-level changes and known processes during iPSC-CM differentiation.

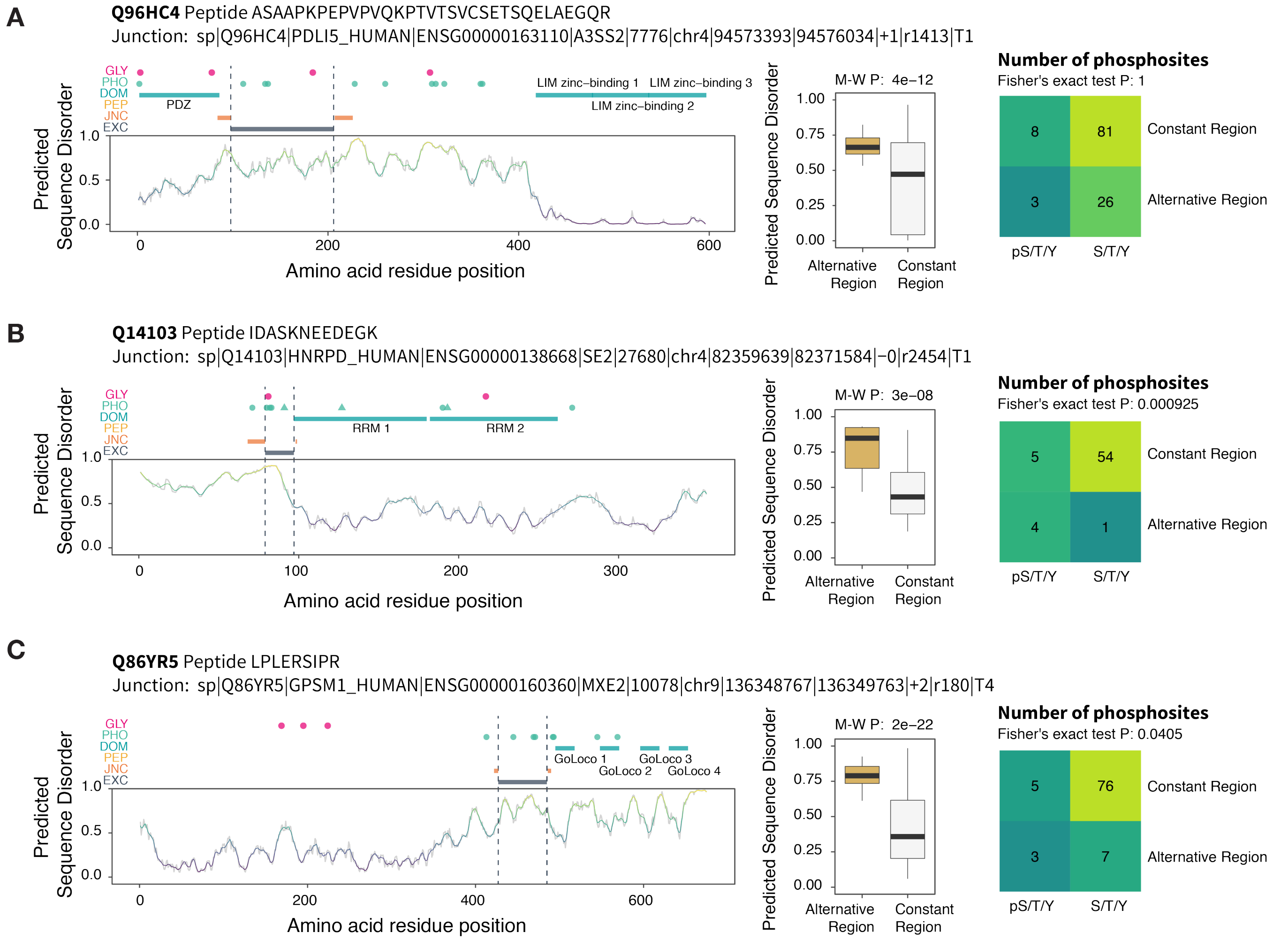

Figure S4. Sequence features of iPSC-CM protein isoforms. Related to Figure 5 and Figure 6.

Sequence features of **A** PDZ and LIM domain protein 5 (PDLIM5), **B** heterogeneous nuclear ribonucleoprotein D (HNRPD) and **C** G-protein-signaling modulator 1 (GPSM1) showing known phosphorylation sites overlapping with the alternative region (residues skipped in the non-canonical isoform) of the protein, and the identified junction peptide spanning the excluded region. Sequence disorder, annotated protein domains and phosphorylation sites were derived as in Figure 5. (Right) Contingency table on the number of annotated phosphorylation sites and serine/threonine/tyrosine (pS/T/Y) that are not annotated to be phosphorylated in the alternative region vs. the rest of the protein sequence (constant region).

A

Sample	Raw Bases (G)	Raw Reads	Clean Reads	Error Rate (%)	GC (%)
iPSC_d0_A	36.6	122043676	120178462	0.02	51.86
iPSC_d0_B	42.3	140865311	138893731	0.02	52.11
iPSC_d0_C	35.0	116526340	114789461	0.02	51.91
iPSC_d2_A	43.3	144289965	142171952	0.02	51.47
iPSC_d2_B	39.8	132606452	130554596	0.02	52.63
iPSC_d2_C	32.9	109584118	107833947	0.02	51.73
iPSC_d7_A	33.3	111123173	103700968	0.03	53.77
iPSC_d7_B	35.4	118161496	115352678	0.02	51.56
iPSC_d7_C	33.6	112012462	110271824	0.02	51.36
iPSC_d14_A	38.2	127357168	125708933	0.02	52.08
iPSC_d14_B	31.7	105753287	104168897	0.02	51.34
iPSC_d14_C	39.8	132664399	130591085	0.02	51.55

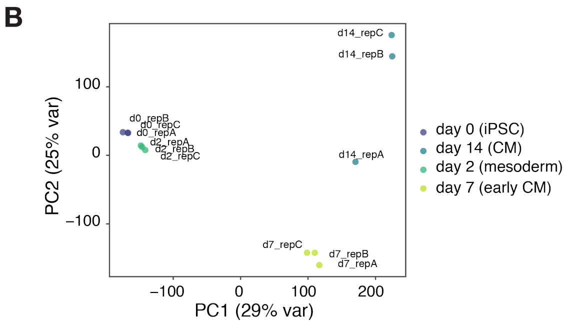

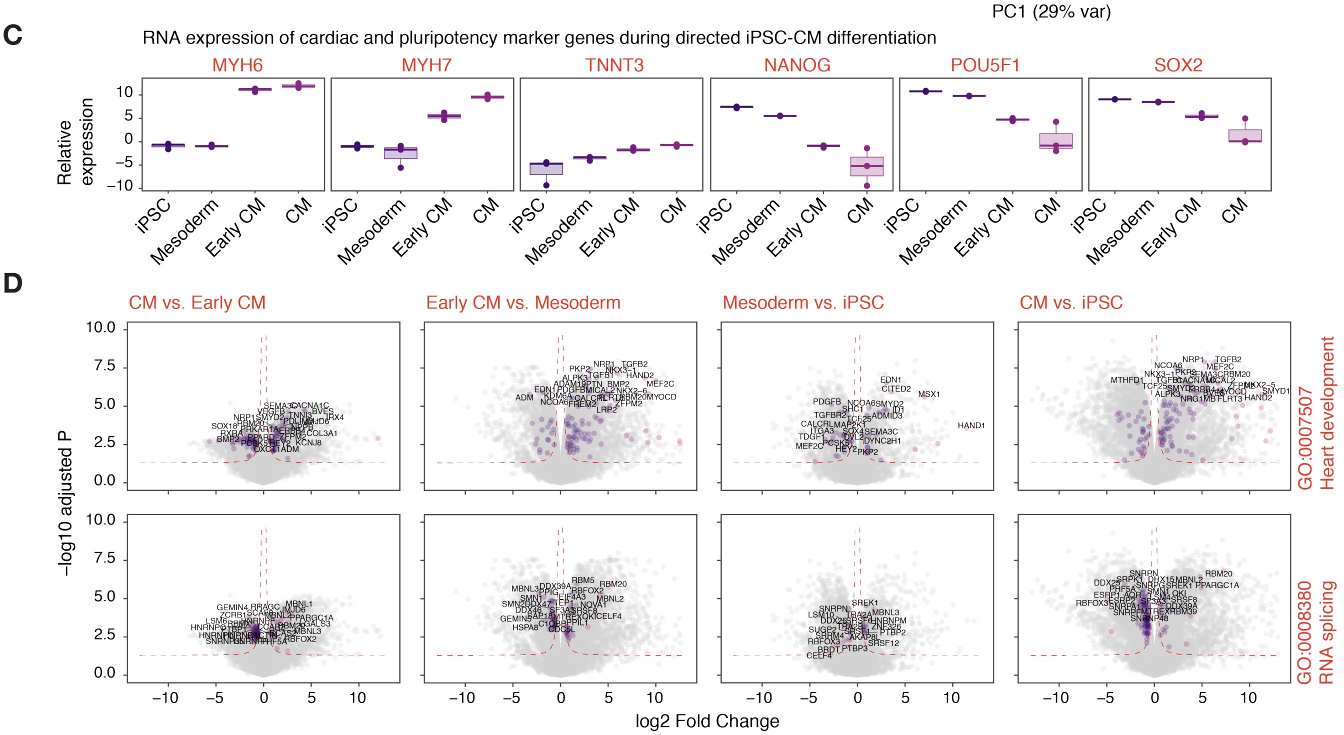

Figure S5. Transcriptome profiles of iPSC-CM differentiation. Related to Figure 6 and Figure 7.

RNA-seq data during iPSC-CM differentiation show expected changes in gene expression profiles including genes involved in cardiac development and splicing. **A.** Deep short-read RNA sequencing data were acquired (105M to 144M reads) in three technical replicates each at four specific developmental time points during iPSC-CM differentiation, corresponding to pluripotent (d0), mesoderm (d2), early cardiomyocyte (d7), and cardiomyocyte (d14) cell stages. **B.** Principal component analysis showing expected clustering of sample transcriptomic profiles by differentiation stages. X-axis: PC1 (29% explained variance); Y-axis: PC2 (25% explained variance). **C.** RNA-seq data showing an expected increase in the expression of cardiomyocyte markers (MYH6, MYH7, TNNT3) concomitant with decreased expression of pluripotency markers (NANOG, POU5F1, SOX2) during each stage of iPSC-CM differentiation. **D.** Volcano plots (log2 fold change vs. –log10 adjusted P) showing differential expression of genes involved in heart development (top; GO:0007507) and RNA splicing (bottom; GO:0008380) during each stage of iPSC-CM differentiation. Genes belonging to highlighted biological processes are in purple, other genes not in the highlighted categories are colored in grey.

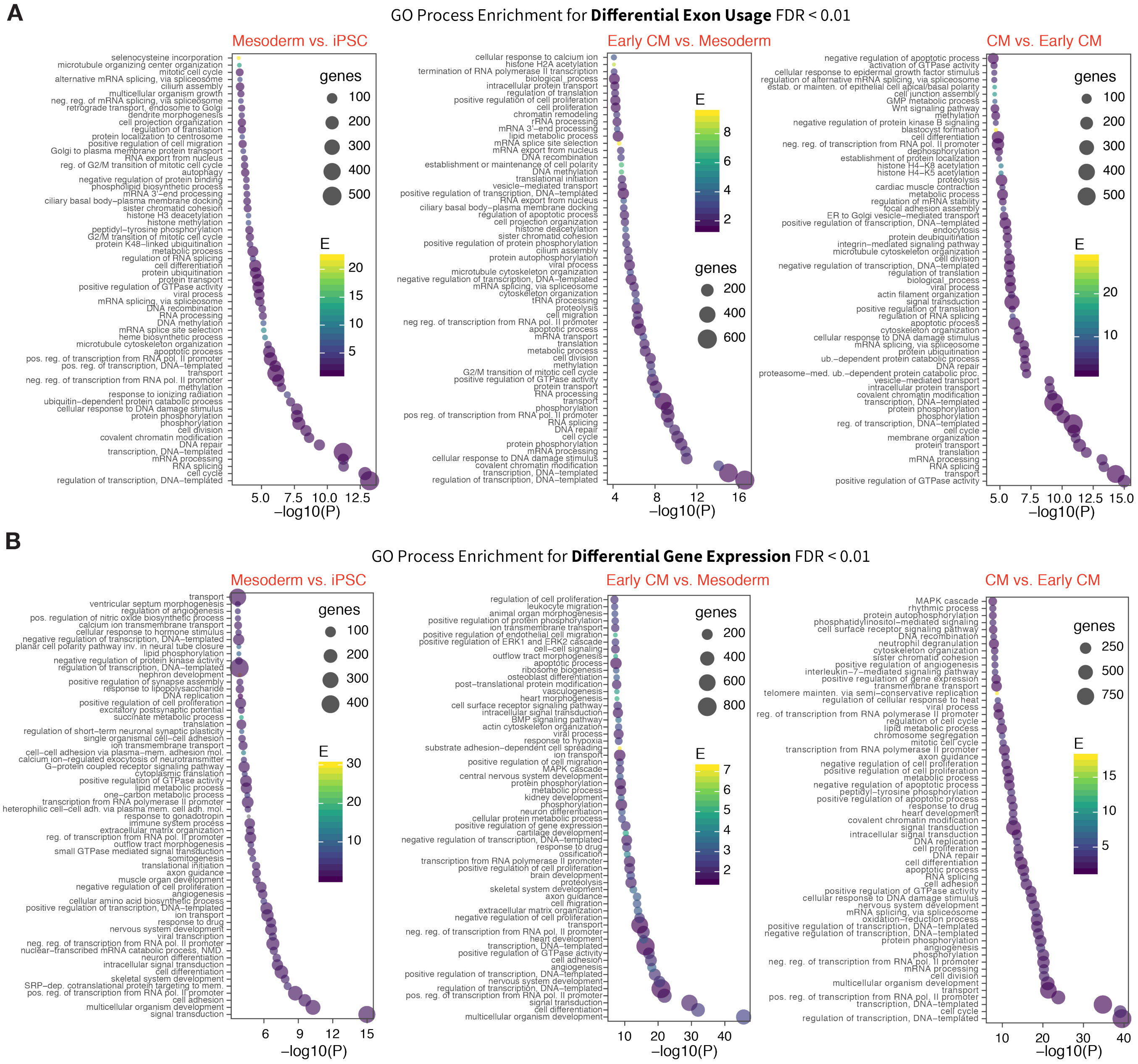

Figure S6. Differential transcript exon usages in iPSC-CM differentiation. Related to Figure 6 and Figure 7.

Top enriched Gene Ontology (GO) biological processes among genes with **A.** significant differential exon usage (rMATS false discovery rate (FDR) < 0.01) and **B.** significant differential gene expression (limma FDR adjusted P < 0.01) when comparing multiple stages of iPSC-CM differentiation. X-axis: –log10 P value of GO term enrichment (Fisher's exact test); y-axis: GO biological process (BP) terms enriched in differentially regulated gene in each analysis; color: fold-enrichment over background (all quantified genes or exons); size of data points: number of genes in the foreground annotated with each GO BP term.

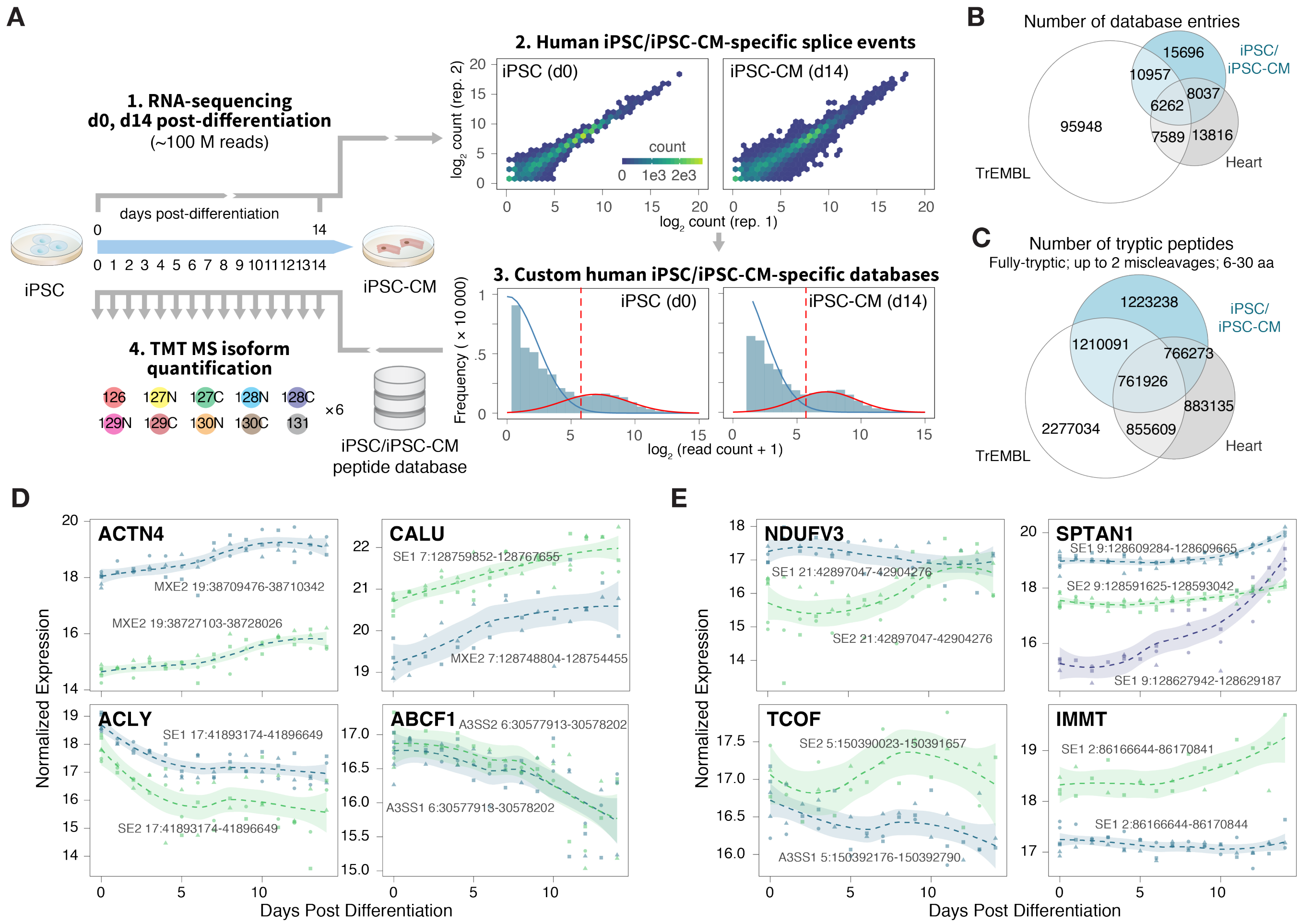

Figure S7. Human iPSC specific isoform sequence database. Related to Figure 7 and STAR Methods.

A. Experimental schema. Deep RNA sequencing data were generated from day 0 and day 14 iPSC and iPSC-cardiomyocytes, respectively. Cell specific databases are used to re-process the time-course tandem mass tag data. **B-C.** The cell-specific databases show partial overlaps with the human heart-specific databases in database entries (**B**) and tryptic peptides (**C**) (6 to 30 amino acids, allowing one miscleavages). **D-E.** Isoforms from the same gene may show concordant (**D**) or discordant (**E**) expression patterns during cardiomyocyte differentiation. Trendline and shaded areas show local regression (loess) and bootstrap uncertainty regions.

Gene Name	Junction Peptide Sequence (Heavy Modification)	Novel Sequence in this Study	Targeted Light Endogenous (m/z, z)	Targeted Heavy Synthetic (m/z, z)	PRM Data Acquisition RT Window (min)	Heavy Peptide ID in PRM (q < 0.01)	Light Peptide ID in PRM (q < 0.01)	MS2 Spectrum Manual Check
PKM1/2	LAPITSDPTEATAVGAVEASFK[13C(6)15N(2)]	✕	725.7109, 3+	728.3823, 3+	52.5—59.5	●	●	●
PKM1/2	CLAAALIVLTESGR[13C(6)15N(4)]	✕	737.4084, 2+	742.4125, 2+	55.0—55.0	●	●	●
MYOM1	AAIAPPSPPCDITCLESFR[13C(6)15N(4)]	●	701.3397, 3+	704.6758, 3+	42.0—55.0	●	●	●
TTN	APHVEFLRPLTDLQVR[13C(6)15N(4)]	●	473.5192, 4+	476.0213, 4+	40.0—48.0	●	●	✕
MYH11	QCQGQAAQEAAGGGR[13C(6)15N(4)]	●	744.8341, 2+	749.8382, 2+	19.5—25.5	●	●	✕
NDUA5	DSGLVGLAVCNTPHER[13C(6)15N(4)]	●	862.9229, 2+	867.9270, 2+	34.5—42.5	●	●	● (yellow)
TENX	VGPVSAVGVTAPGK[13C(6)15N(2)]	●	619.8588, 2+	623.8659, 2+	31.5—38.0	●	●	●
SVIL	DSEGDTPSLINWPSSK[13C(6)15N(2)]	●	866.9049, 2+	870.9120, 2+	44.5—51.5	●	●	●
PALLD	LLGADSATVFNIQEPEEETANQIYWFK	●	1038.5083, 3+	1041.1797, 3+	60.0—69.0	●	●	✕
RYR2	VLDIANVLFHLEQVEHPQR[13C(6)15N(4)]	●	565.0577, 4+	567.5597, 4+	57.0—65.0	●	●	●
PCBP2	YSTGSDSASFPHTTPSMCLNPDLEGPPLELTK[13C(6)15N(2)]	●	1150.5341, 3+	1153.2055, 3+	50.0—58.0	●	●	✕
MYBPC3	AITQLLCETEGR[13C(6)15N(4)]	●	695.8534, 2+	700.8576, 2+	36.0—44.0	●	✕	✕
MYBPC3	RTDSHEDTGILDFSSLLK[13C(6)15N(2)]	●	678.6761, 3+	681.3475, 3+	48.0—55.0	●	●	● (yellow)
PDLI5	ANLSSSTGNVEDSFEGFR[13C(6)15N(4)]	●	958.4347, 2+	963.9388, 2+	40.0—47.0	●	✕	✕

Table S1. Methods table for targeted parallel reaction monitoring. Related to Figure 4, Figure S2, and STAR Methods.

Targeted parallel reaction monitoring (PRM) mass spectrometry data were acquired for 14 selected isoform junction peptides from 2 known and 12 candidate undocumented peptide sequences. Out of the 12 undocumented sequences, 10 were tentatively found in the independent human whole heart tissue lysate by PRM using a Crux/percolator search (q < 0.01 threshold) that allows for optional heavy isotope labels ([13C(6)15N(2)] for peptides ending with lysine; [13C(6)15N(4)] for peptides ending with arginine) in the synthetic peptides. Out of the 10 sequences, 6 showed excellent (4; green) to tentative (2; yellow) fragmentation spectrum matches to the synthetic peptide upon manual inspection (see Figure S2).
