# Supplementary material for: Splice-Junction-Based Mapping of Alternative Isoforms in the Human Proteome
Source: Cell Rep. Author manuscript; Available in PMC 2020 Jan 15. (PMC6961840; doi:10.1016/j.celrep.2019.11.026)
Supplement: 2 [file NIHMS1546469-supplement-2.zip › DF1/PXD000561/Prostate/Prostate_10_ARHGAP32_AVASGQTQTGDSK.pdf]

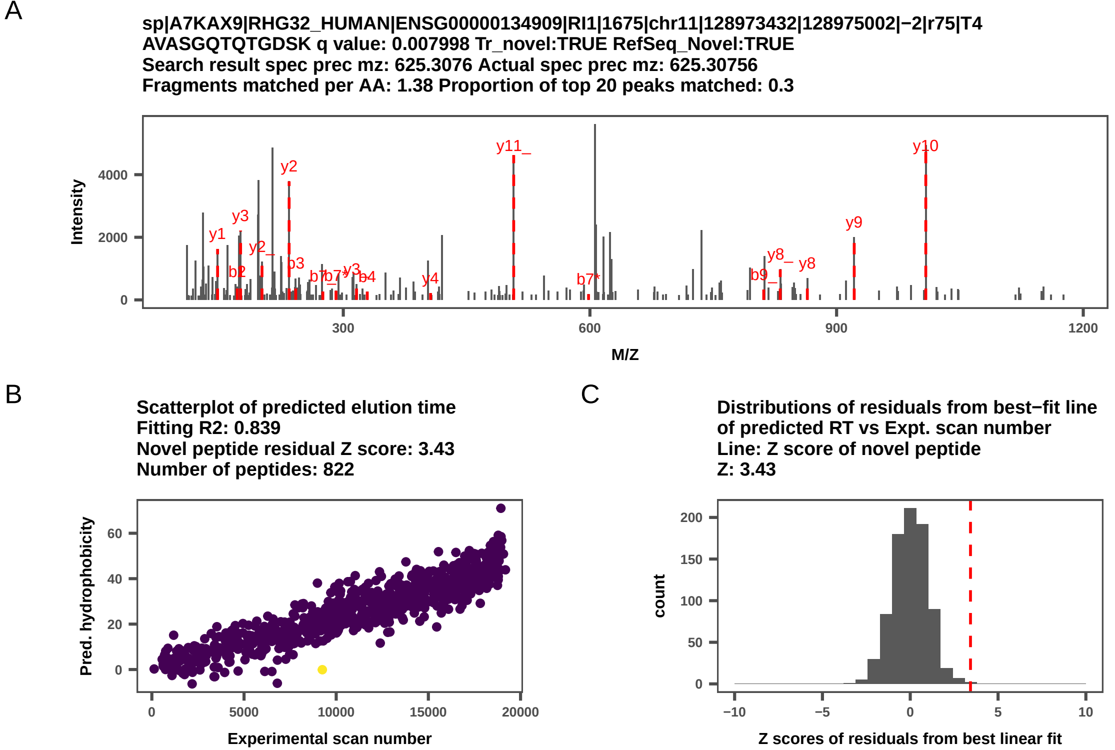
A
sp|A7KAX9|RHG32_HUMAN|ENSG00000134909|RI1|1675|chr11|128973432|128975002|−2|r75|T4
AVASGQTQTGDSK q value: 0.007998 Tr_novel:TRUE RefSeq_Novel:TRUE
Search result spec prec mz: 625.3076 Actual spec prec mz: 625.30756
Fragments matched per AA: 1.38 Proportion of top 20 peaks matched: 0.3
Intensity
4000
2000
0
300
600
900
1200
M/Z
y1
y3
y2
y11_
y10
y9
y8
y4
B
Scatterplot of predicted elution time
Fitting R2: 0.839
Novel peptide residual Z score: 3.43
Number of peptides: 822
Pred. hydrophobicity
60
40
20
0
0
5000
10000
15000
20000
Experimental scan number
C
Distributions of residuals from best−fit line
of predicted RT vs Expt. scan number
Line: Z score of novel peptide
Z: 3.43
count
200
150
100
50
0
−10
−5
0
5
10
Z scores of residuals from best linear fit
