# Supplementary material for: Splice-Junction-Based Mapping of Alternative Isoforms in the Human Proteome
Source: Cell Rep. Author manuscript; Available in PMC 2020 Jan 15. (PMC6961840; doi:10.1016/j.celrep.2019.11.026)
Supplement: 2 [file NIHMS1546469-supplement-2.zip › DF1/PXD000561/Prostate/Prostate_7_INTS6_KPFFLGASNNYHNYTSASMNQR.pdf]

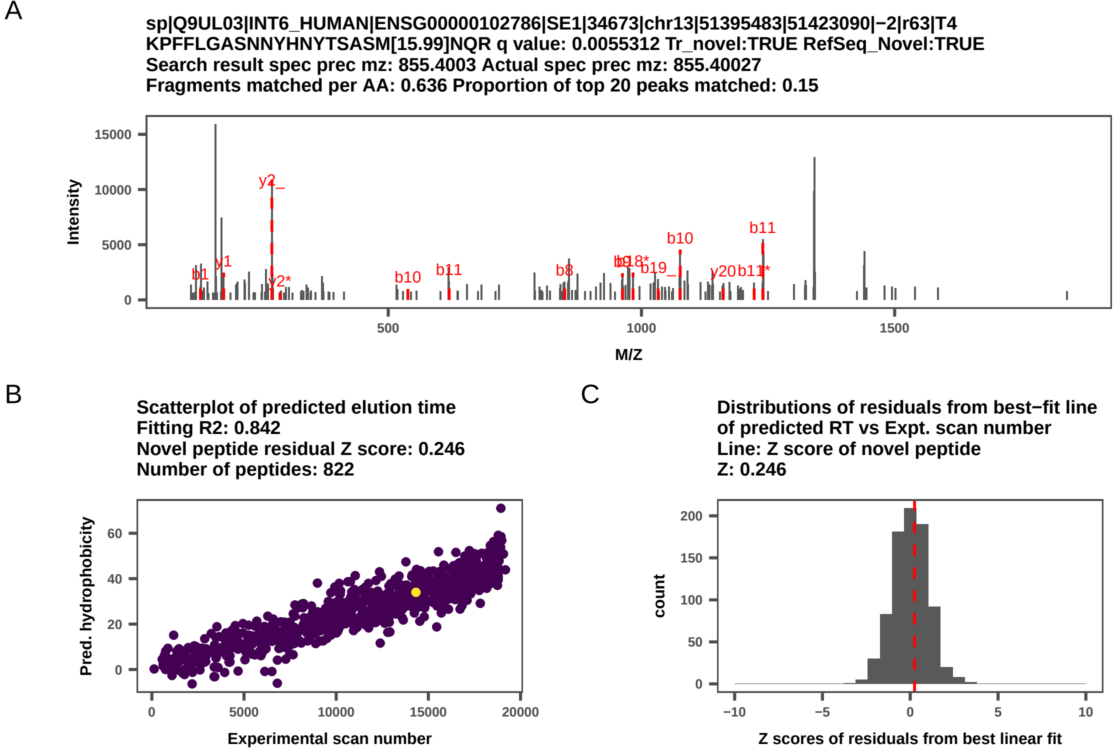
A
sp|Q9UL03|INT6_HUMAN|ENSG00000102786|SE1|34673|chr13|51395483|51423090|−2|r63|T4
KPFFLGASNNYHNYTSASM[15.99]NQR q value: 0.0055312 Tr_novel:TRUE RefSeq_Novel:TRUE
Search result spec prec mz: 855.4003 Actual spec prec mz: 855.40027
Fragments matched per AA: 0.636 Proportion of top 20 peaks matched: 0.15
Intensity
15000
10000
5000
0
M/Z
500
1000
1500
b1
y1
y2*
b10
b11
b8
b19_
b10
y20
b11*
b11
B
Scatterplot of predicted elution time
Fitting R2: 0.842
Novel peptide residual Z score: 0.246
Number of peptides: 822
Pred. hydrophobicity
60
40
20
0
Experimental scan number
0
5000
10000
15000
20000
C
Distributions of residuals from best−fit line
of predicted RT vs Expt. scan number
Line: Z score of novel peptide
Z: 0.246
count
200
150
100
50
0
Z scores of residuals from best linear fit
−10
−5
0
5
10
