# Supplementary material for: Splice-Junction-Based Mapping of Alternative Isoforms in the Human Proteome
Source: Cell Rep. Author manuscript; Available in PMC 2020 Jan 15. (PMC6961840; doi:10.1016/j.celrep.2019.11.026)
Supplement: 2 [file NIHMS1546469-supplement-2.zip › DF1/PXD000561/Prostate/Prostate_5_HMG20B_AAAAAPAGGK.pdf]

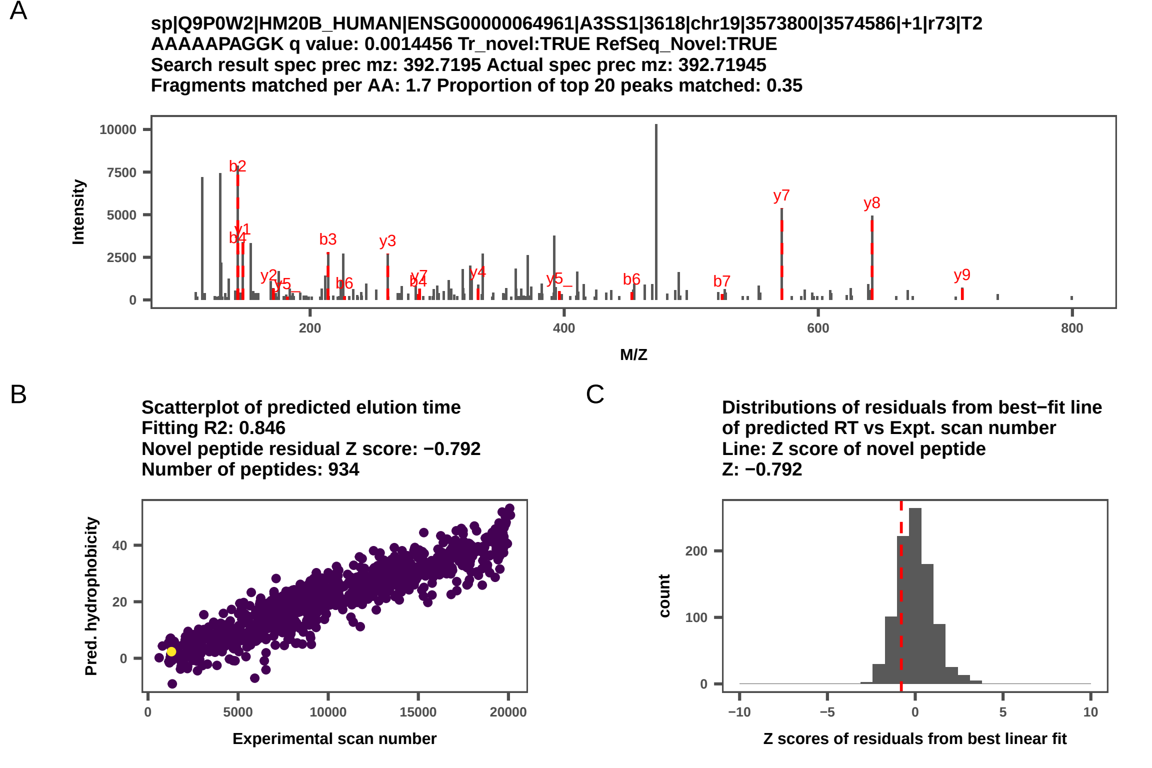
A
sp|Q9P0W2|HM20B_HUMAN|ENSG00000064961|A3SS1|3618|chr19|3573800|3574586|+1|r73|T2
AAAAAPAGGK q value: 0.0014456 Tr_novel:TRUE RefSeq_Novel:TRUE
Search result spec prec mz: 392.7195 Actual spec prec mz: 392.71945
Fragments matched per AA: 1.7 Proportion of top 20 peaks matched: 0.35
Intensity
10000
7500
5000
2500
0
b2
b4
y1
b3
y3
y2
y5_
b6
y7
b4
y4
y5_
b6
b7
y7
y8
y9
200
400
600
800
M/Z
B
Scatterplot of predicted elution time
Fitting R2: 0.846
Novel peptide residual Z score: −0.792
Number of peptides: 934
Pred. hydrophobicity
40
20
0
0
5000
10000
15000
20000
Experimental scan number
C
Distributions of residuals from best−fit line
of predicted RT vs Expt. scan number
Line: Z score of novel peptide
Z: −0.792
count
200
100
0
−10
−5
0
5
10
Z scores of residuals from best linear fit
