# Supplementary material for: Splice-Junction-Based Mapping of Alternative Isoforms in the Human Proteome
Source: Cell Rep. Author manuscript; Available in PMC 2020 Jan 15. (PMC6961840; doi:10.1016/j.celrep.2019.11.026)
Supplement: 2 [file NIHMS1546469-supplement-2.zip › DF1/PXD000561/Prostate/Prostate_3_ARRDC1_SSCFLSLSHGR.pdf]

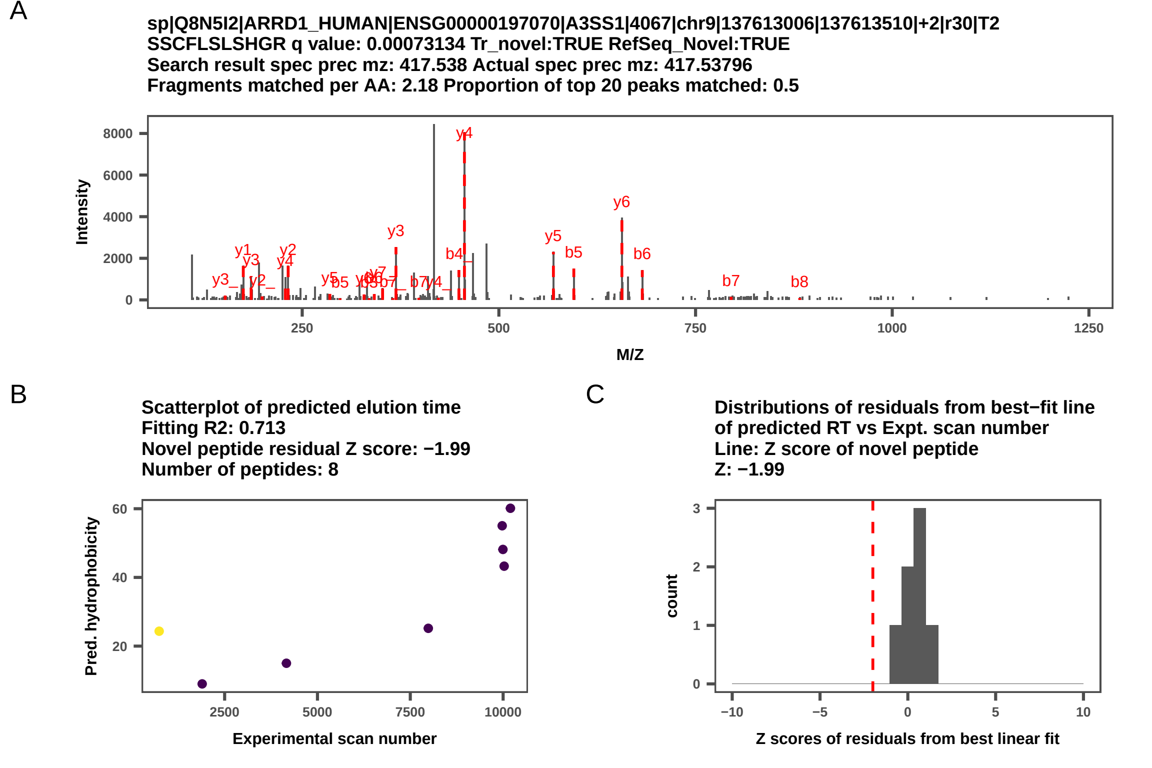
A
sp|Q8N5I2|ARRD1_HUMAN|ENSG00000197070|A3SS1|4067|chr9|137613006|137613510|+2|r30|T2
SSCFLSLSHGR q value: 0.00073134 Tr_novel:TRUE RefSeq_Novel:TRUE
Search result spec prec mz: 417.538 Actual spec prec mz: 417.53796
Fragments matched per AA: 2.18 Proportion of top 20 peaks matched: 0.5
Intensity
8000
6000
4000
2000
0
y4
y6
y3
y5
b5
b6
b4_
y1
y2
b7
b8
250
500
750
1000
1250
M/Z
B
Scatterplot of predicted elution time
Fitting R2: 0.713
Novel peptide residual Z score: −1.99
Number of peptides: 8
Pred. hydrophobicity
60
40
20
2500
5000
7500
10000
Experimental scan number
C
Distributions of residuals from best−fit line
of predicted RT vs Expt. scan number
Line: Z score of novel peptide
Z: −1.99
count
3
2
1
0
−10
−5
0
5
10
Z scores of residuals from best linear fit
