# Supplementary material for: Splice-Junction-Based Mapping of Alternative Isoforms in the Human Proteome
Source: Cell Rep. Author manuscript; Available in PMC 2020 Jan 15. (PMC6961840; doi:10.1016/j.celrep.2019.11.026)
Supplement: 2 [file NIHMS1546469-supplement-2.zip › DF1/PXD000561/Prostate/Prostate_4_SYPL1_ETSLHSPSNTSAPHSQGGIPPPTGL.pdf]

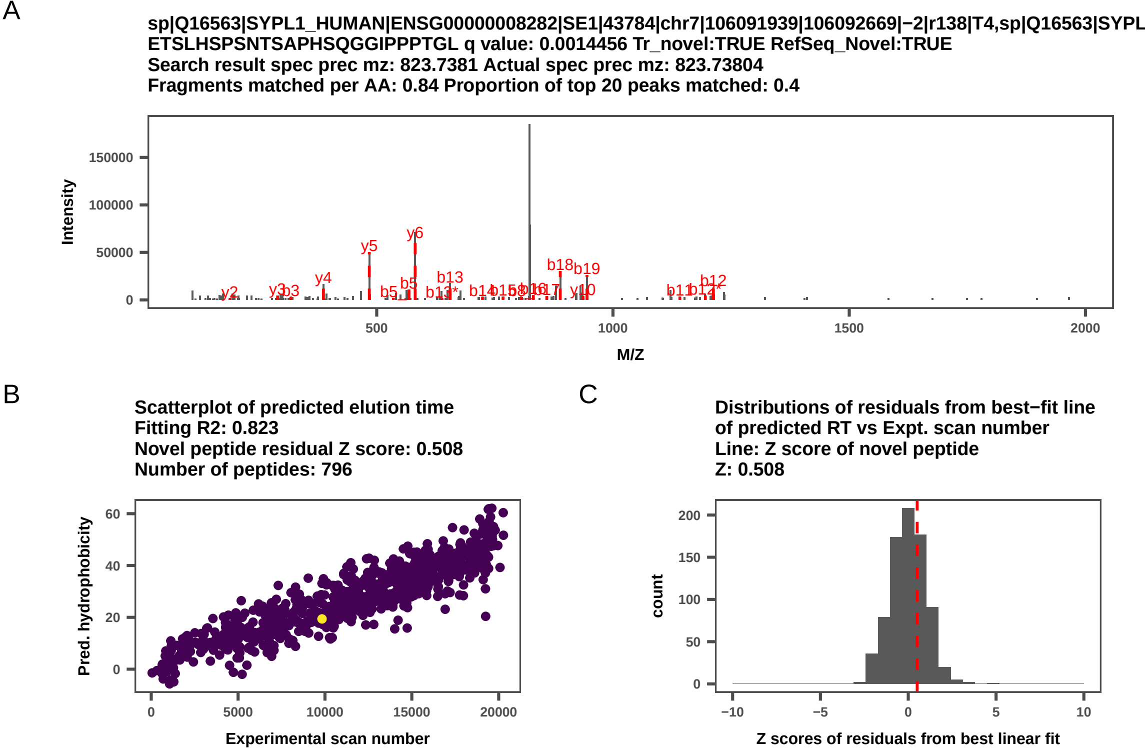
A
sp|Q16563|SYPL1_HUMAN|ENSG00000008282|SE1|43784|chr7|106091939|106092669|−2|r138|T4,sp|Q16563|SYPL
ETSLHSPSNTSAPHSQGGIPPPTGL q value: 0.0014456 Tr_novel:TRUE RefSeq_Novel:TRUE
Search result spec prec mz: 823.7381 Actual spec prec mz: 823.73804
Fragments matched per AA: 0.84 Proportion of top 20 peaks matched: 0.4
Intensity
150000
100000
50000
0
y2
y3
b3
y4
y5
y6
b5
b13
b13*
b18
b19
y10
b11
b12
500
1000
1500
2000
M/Z
B
Scatterplot of predicted elution time
Fitting R2: 0.823
Novel peptide residual Z score: 0.508
Number of peptides: 796
Pred. hydrophobicity
60
40
20
0
5000
10000
15000
20000
Experimental scan number
C
Distributions of residuals from best−fit line
of predicted RT vs Expt. scan number
Line: Z score of novel peptide
Z: 0.508
count
200
150
100
50
0
−10
−5
5
10
Z scores of residuals from best linear fit
