# Supplementary material for: Splice-Junction-Based Mapping of Alternative Isoforms in the Human Proteome
Source: Cell Rep. Author manuscript; Available in PMC 2020 Jan 15. (PMC6961840; doi:10.1016/j.celrep.2019.11.026)
Supplement: 2 [file NIHMS1546469-supplement-2.zip › DF1/PXD000561/Prostate/Prostate_1_SVIL_RPELCTSHSETPTVDDEEKVDER.pdf]

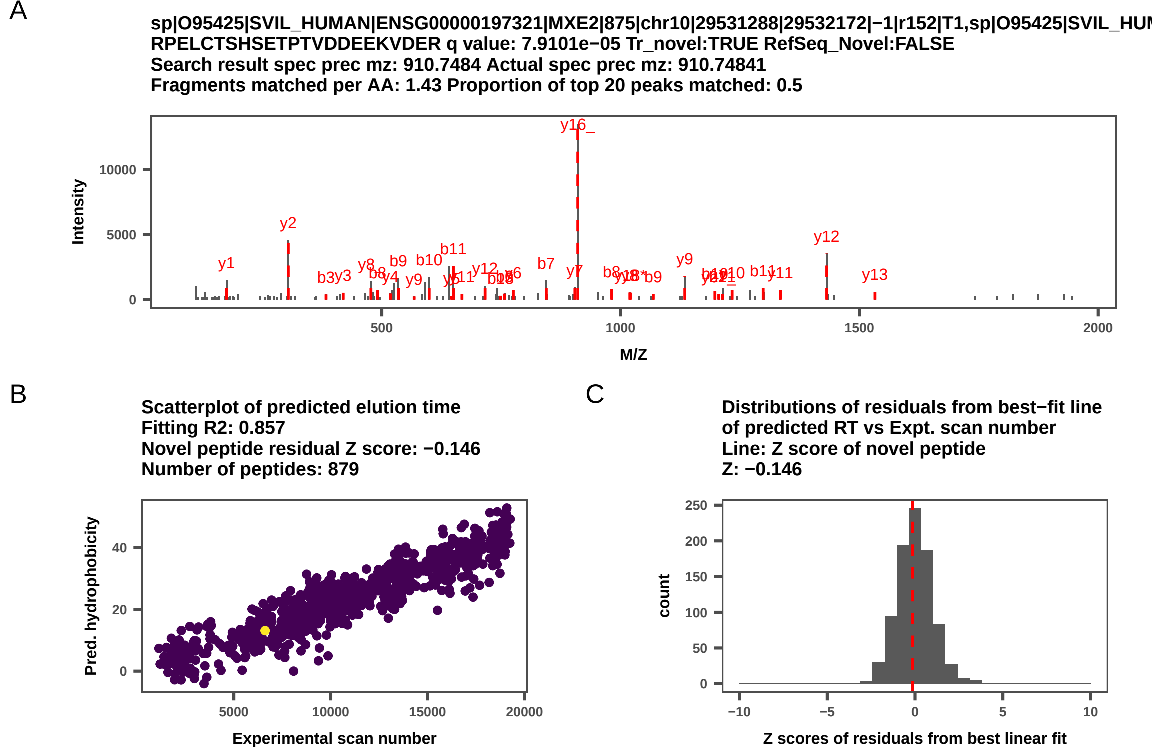
A
sp|O95425|SVIL_HUMAN|ENSG00000197321|MXE2|875|chr10|29531288|29532172|−1|r152|T1,sp|O95425|SVIL_HUM
RPELCTSHSETPTVDDEEKVDER q value: 7.9101e−05 Tr_novel:TRUE RefSeq_Novel:FALSE
Search result spec prec mz: 910.7484 Actual spec prec mz: 910.74841
Fragments matched per AA: 1.43 Proportion of top 20 peaks matched: 0.5
Intensity
10000
5000
0
500
1000
1500
2000
M/Z
B
Scatterplot of predicted elution time
Fitting R2: 0.857
Novel peptide residual Z score: −0.146
Number of peptides: 879
Pred. hydrophobicity
40
20
0
5000
10000
15000
20000
Experimental scan number
C
Distributions of residuals from best−fit line
of predicted RT vs Expt. scan number
Line: Z score of novel peptide
Z: −0.146
count
250
200
150
100
50
0
−10
−5
0
5
10
Z scores of residuals from best linear fit
