# Supplementary material for: Splice-Junction-Based Mapping of Alternative Isoforms in the Human Proteome
Source: Cell Rep. Author manuscript; Available in PMC 2020 Jan 15. (PMC6961840; doi:10.1016/j.celrep.2019.11.026)
Supplement: 2 [file NIHMS1546469-supplement-2.zip › DF1/PXD000561/Prostate/Prostate_6_PPFIBP1_KAAALEQMDGIIAGSK.pdf]

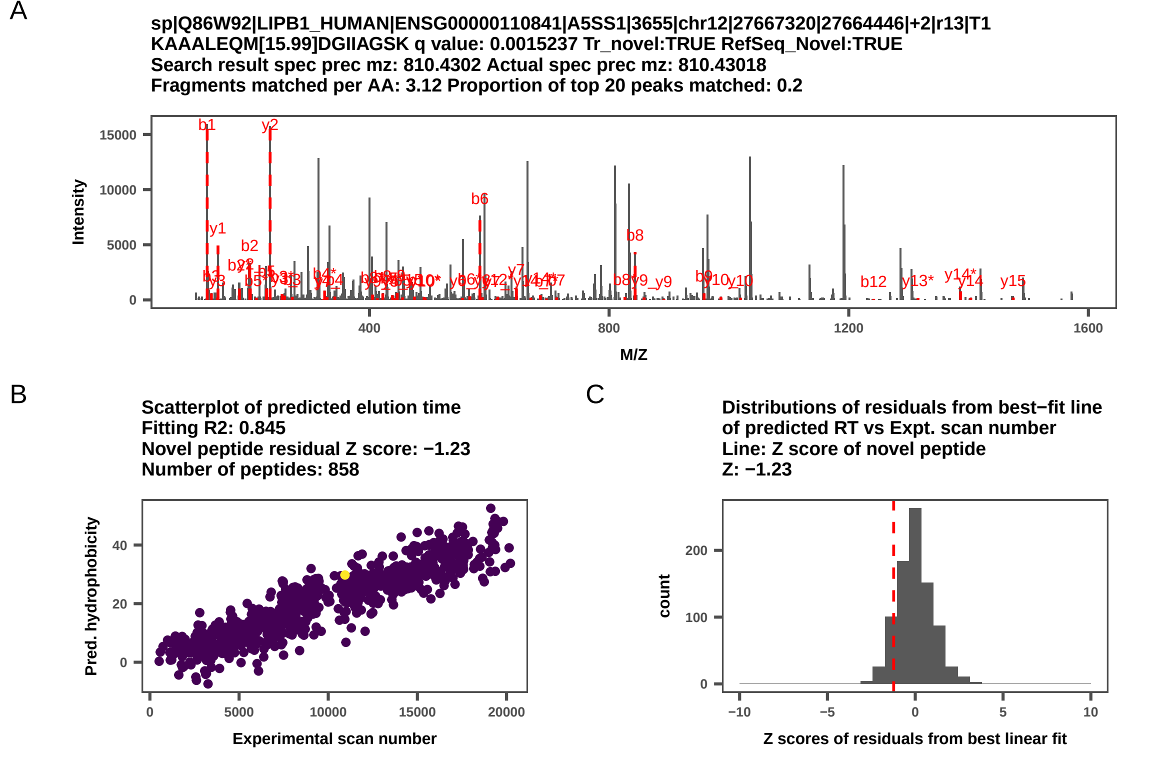
A
sp|Q86W92|LIPB1_HUMAN|ENSG00000110841|A5SS1|3655|chr12|27667320|27664446|+2|r13|T1
KAAALEQM[15.99]DGIIAGSK q value: 0.0015237 Tr_novel:TRUE RefSeq_Novel:TRUE
Search result spec prec mz: 810.4302 Actual spec prec mz: 810.43018
Fragments matched per AA: 3.12 Proportion of top 20 peaks matched: 0.2
Intensity
15000
10000
5000
0
b1
y2
y1
b2
b6
b8
b9
y10
b12
y13*
y14*
y14
y15
400
800
1200
1600
M/Z
B
Scatterplot of predicted elution time
Fitting R2: 0.845
Novel peptide residual Z score: −1.23
Number of peptides: 858
Pred. hydrophobicity
40
20
0
0
5000
10000
15000
20000
Experimental scan number
C
Distributions of residuals from best−fit line
of predicted RT vs Expt. scan number
Line: Z score of novel peptide
Z: −1.23
count
200
100
0
−10
−5
0
5
10
Z scores of residuals from best linear fit
