# Supplementary material for: Splice-Junction-Based Mapping of Alternative Isoforms in the Human Proteome
Source: Cell Rep. Author manuscript; Available in PMC 2020 Jan 15. (PMC6961840; doi:10.1016/j.celrep.2019.11.026)
Supplement: 2 [file NIHMS1546469-supplement-2.zip › DF1/PXD000561/Prostate/Prostate_8_C1QA_GHIYQGSEADSVFSGFLIFPSGPLASGQA.pdf]

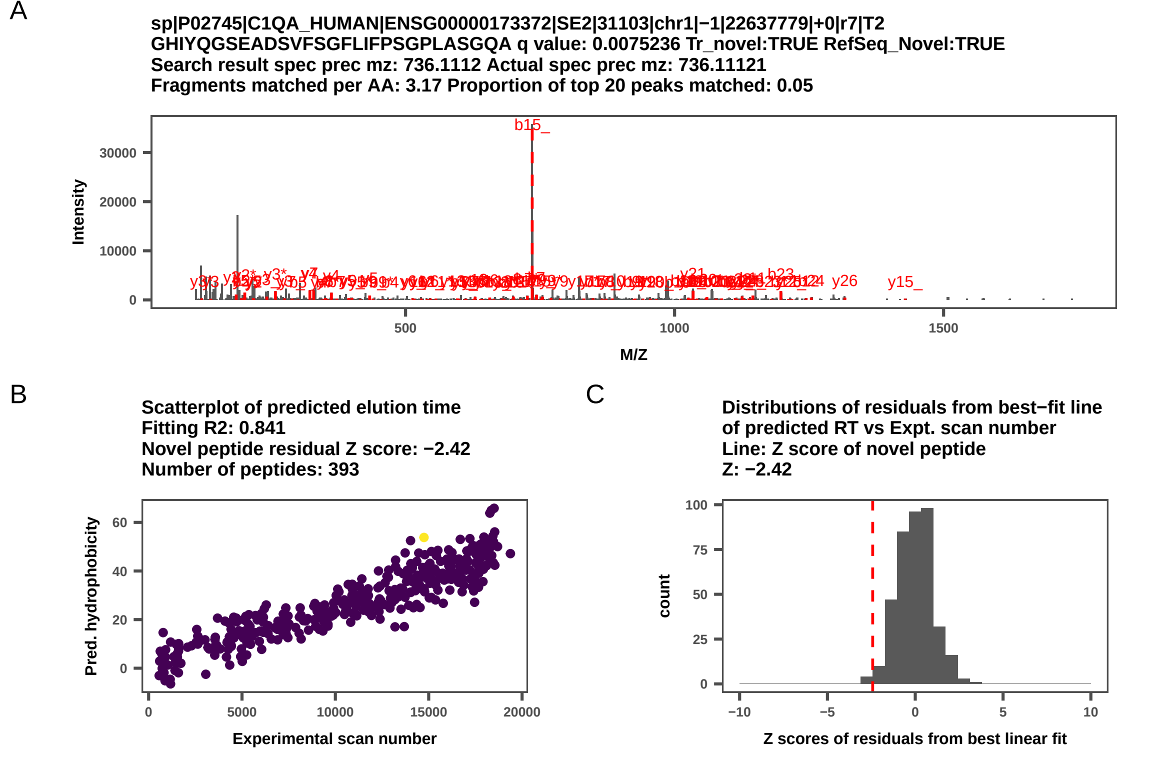
A
sp|P02745|C1QA_HUMAN|ENSG00000173372|SE2|31103|chr1|−1|22637779|+0|r7|T2
GHIYQGSEADSVFSGFLIFPSGPLASGQA q value: 0.0075236 Tr_novel:TRUE RefSeq_Novel:TRUE
Search result spec prec mz: 736.1112 Actual spec prec mz: 736.11121
Fragments matched per AA: 3.17 Proportion of top 20 peaks matched: 0.05
Intensity
30000
20000
10000
0
b15_
y26
y15_
500
1000
1500
M/Z
B
Scatterplot of predicted elution time
Fitting R2: 0.841
Novel peptide residual Z score: −2.42
Number of peptides: 393
Pred. hydrophobicity
60
40
20
0
0
5000
10000
15000
20000
Experimental scan number
C
Distributions of residuals from best−fit line
of predicted RT vs Expt. scan number
Line: Z score of novel peptide
Z: −2.42
count
100
75
50
25
0
−10
−5
0
5
10
Z scores of residuals from best linear fit
