# Supplementary material for: Splice-Junction-Based Mapping of Alternative Isoforms in the Human Proteome
Source: Cell Rep. Author manuscript; Available in PMC 2020 Jan 15. (PMC6961840; doi:10.1016/j.celrep.2019.11.026)
Supplement: 2 [file NIHMS1546469-supplement-2.zip › DF1/PXD000561/Prostate/Prostate_9_MAPKBP1_LSHQTVTR.pdf]

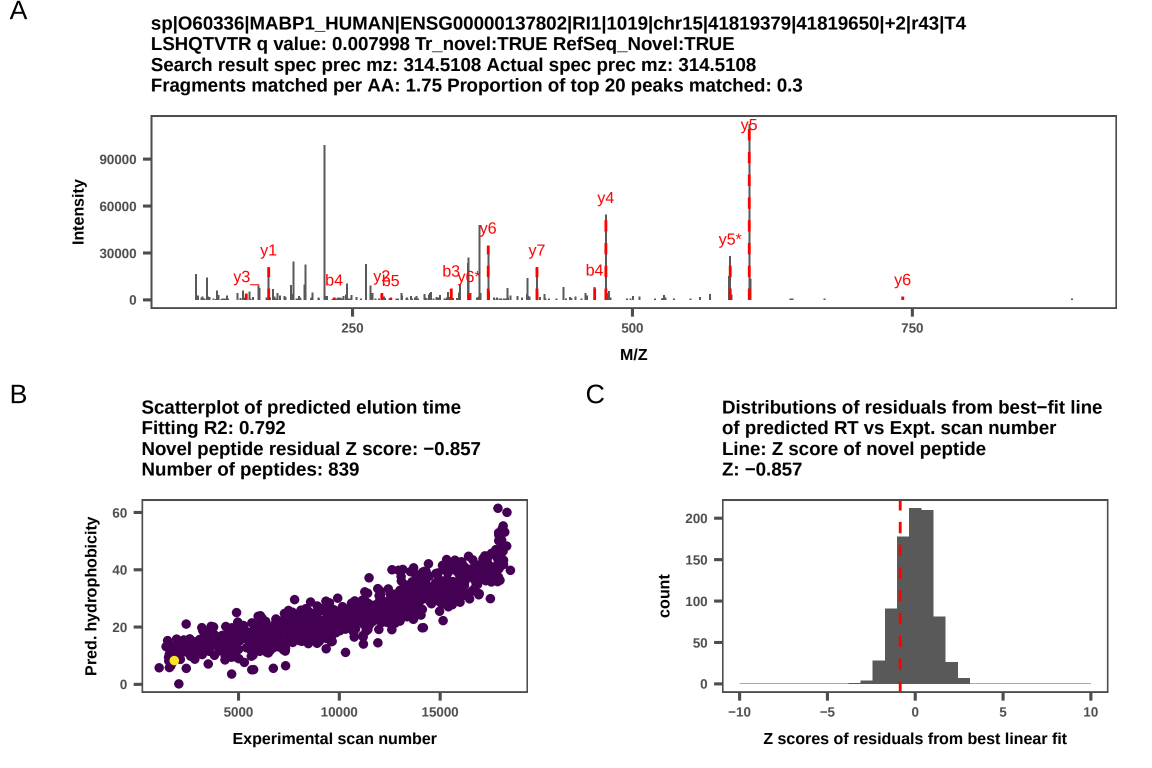
A
sp|O60336|MABP1_HUMAN|ENSG00000137802|RI1|1019|chr15|41819379|41819650|+2|r43|T4
LSHQTVTR q value: 0.007998 Tr_novel:TRUE RefSeq_Novel:TRUE
Search result spec prec mz: 314.5108 Actual spec prec mz: 314.5108
Fragments matched per AA: 1.75 Proportion of top 20 peaks matched: 0.3
Intensity
90000
60000
30000
0
y5
y4
y6
y1
y7
y5*
y3_
b4
y2
b5
b3
y6*
b4
y6
250
500
750
M/Z
B
Scatterplot of predicted elution time
Fitting R2: 0.792
Novel peptide residual Z score: −0.857
Number of peptides: 839
Pred. hydrophobicity
60
40
20
0
5000
10000
15000
Experimental scan number
C
Distributions of residuals from best−fit line
of predicted RT vs Expt. scan number
Line: Z score of novel peptide
Z: −0.857
count
200
150
100
50
0
−10
−5
0
5
10
Z scores of residuals from best linear fit
