# Supplementary material for: Splice-Junction-Based Mapping of Alternative Isoforms in the Human Proteome
Source: Cell Rep. Author manuscript; Available in PMC 2020 Jan 15. (PMC6961840; doi:10.1016/j.celrep.2019.11.026)
Supplement: 2 [file NIHMS1546469-supplement-2.zip › DF1/PXD000561/Testis/Testis_8_KIAA1210_NLQVIVEPKEEEPNLPLVSEEEK.pdf]

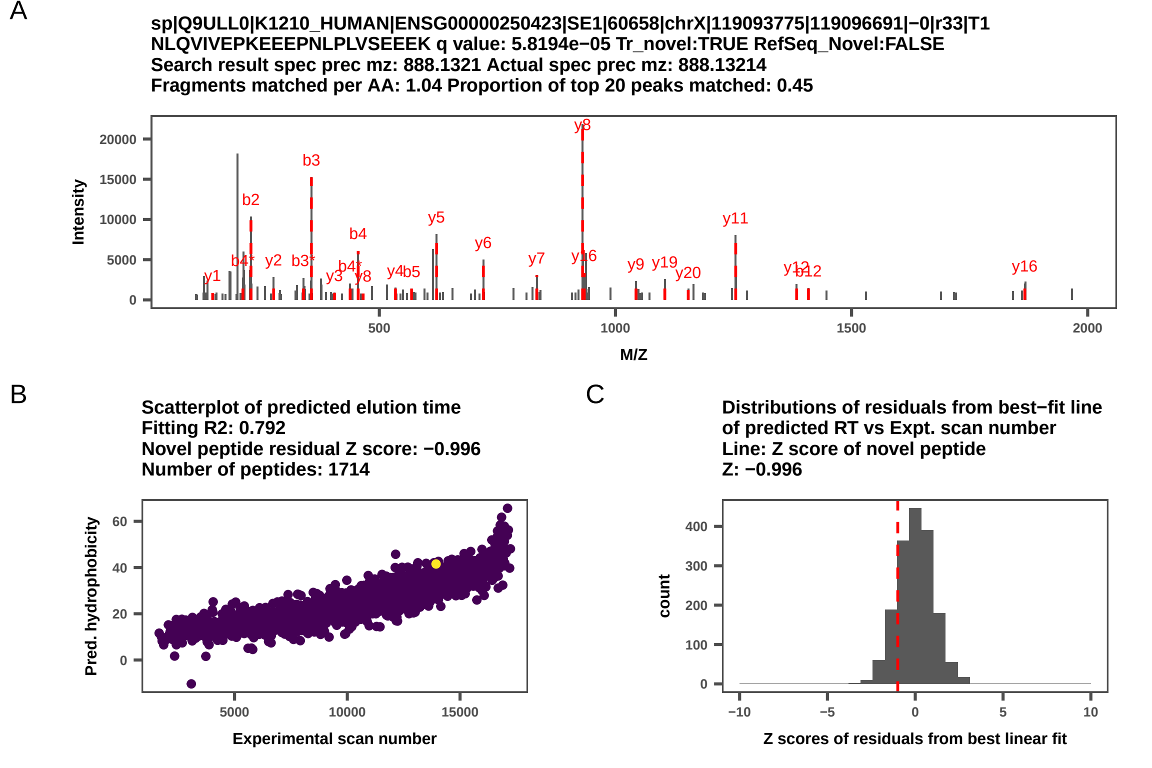
A
sp|Q9ULL0|K1210_HUMAN|ENSG00000250423|SE1|60658|chrX|119093775|119096691|−0|r33|T1
NLQVIVEPKEEEPNLPLVSEEEK q value: 5.8194e−05 Tr_novel:TRUE RefSeq_Novel:FALSE
Search result spec prec mz: 888.1321 Actual spec prec mz: 888.13214
Fragments matched per AA: 1.04 Proportion of top 20 peaks matched: 0.45
Intensity
20000
15000
10000
5000
0
500
1000
1500
2000
M/Z
y8
b3
b2
y5
b4
y11
y6
y7
y16
y1
b4*
y2
b3*
y3
b4*
y8
y4
b5
y9
y19
y20
y12
b12
y16
B
Scatterplot of predicted elution time
Fitting R2: 0.792
Novel peptide residual Z score: −0.996
Number of peptides: 1714
Pred. hydrophobicity
60
40
20
0
5000
10000
15000
Experimental scan number
C
Distributions of residuals from best−fit line
of predicted RT vs Expt. scan number
Line: Z score of novel peptide
Z: −0.996
count
400
300
200
100
0
−10
−5
0
5
10
Z scores of residuals from best linear fit
