# Supplementary material for: Splice-Junction-Based Mapping of Alternative Isoforms in the Human Proteome
Source: Cell Rep. Author manuscript; Available in PMC 2020 Jan 15. (PMC6961840; doi:10.1016/j.celrep.2019.11.026)
Supplement: 2 [file NIHMS1546469-supplement-2.zip › DF1/PXD000561/Testis/Testis_7_PCBP2_YSTGSDSASFPHTTPSMCLNPDLEGPPLELTK.pdf]

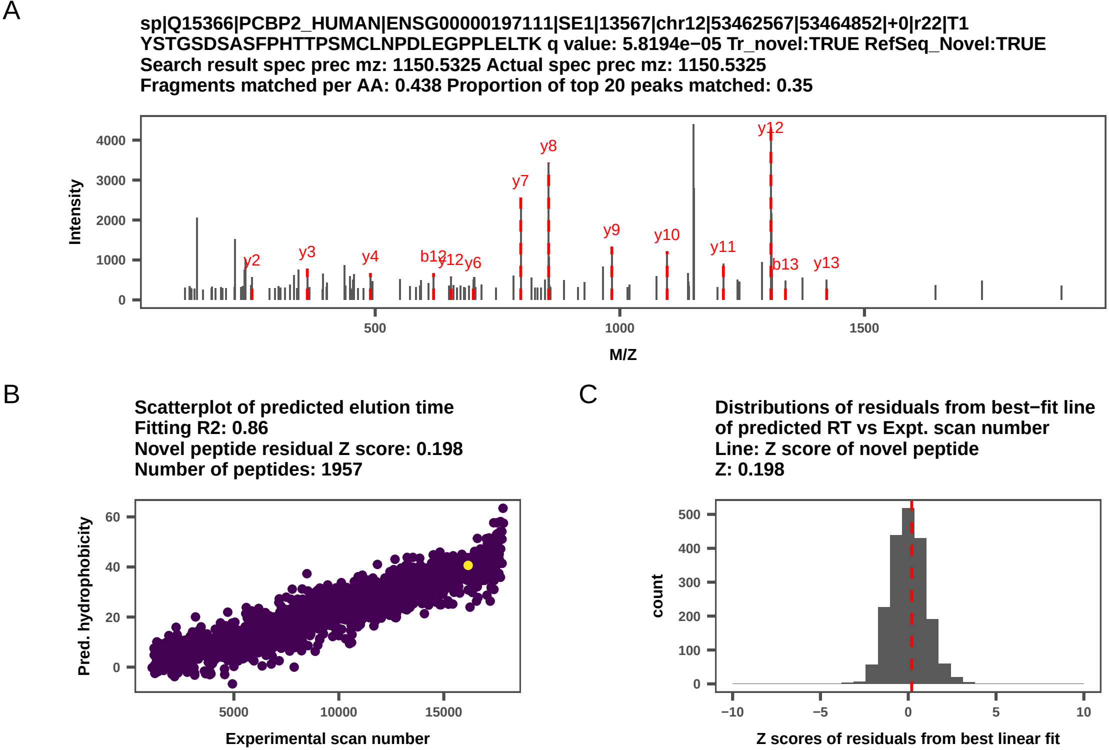
A
sp|Q15366|PCBP2_HUMAN|ENSG00000197111|SE1|13567|chr12|53462567|53464852|+0|r22|T1
YSTGSDSASFPHTTPSMCLNPDLEGPPLELTK q value: 5.8194e−05 Tr_novel:TRUE RefSeq_Novel:TRUE
Search result spec prec mz: 1150.5325 Actual spec prec mz: 1150.5325
Fragments matched per AA: 0.438 Proportion of top 20 peaks matched: 0.35
Intensity
4000
3000
2000
1000
0
y2
y3
y4
b12
y12
y6
y7
y8
y9
y10
y11
y12
b13
y13
500
1000
1500
M/Z
B
Scatterplot of predicted elution time
Fitting R2: 0.86
Novel peptide residual Z score: 0.198
Number of peptides: 1957
Pred. hydrophobicity
60
40
20
0
5000
10000
15000
Experimental scan number
C
Distributions of residuals from best−fit line
of predicted RT vs Expt. scan number
Line: Z score of novel peptide
Z: 0.198
count
500
400
300
200
100
0
−10
−5
0
5
10
Z scores of residuals from best linear fit
