# Supplementary material for: Splice-Junction-Based Mapping of Alternative Isoforms in the Human Proteome
Source: Cell Rep. Author manuscript; Available in PMC 2020 Jan 15. (PMC6961840; doi:10.1016/j.celrep.2019.11.026)
Supplement: 2 [file NIHMS1546469-supplement-2.zip › DF1/PXD000561/Testis/Testis_6_STK39_VKEENPEGPPNANEDYR.pdf]

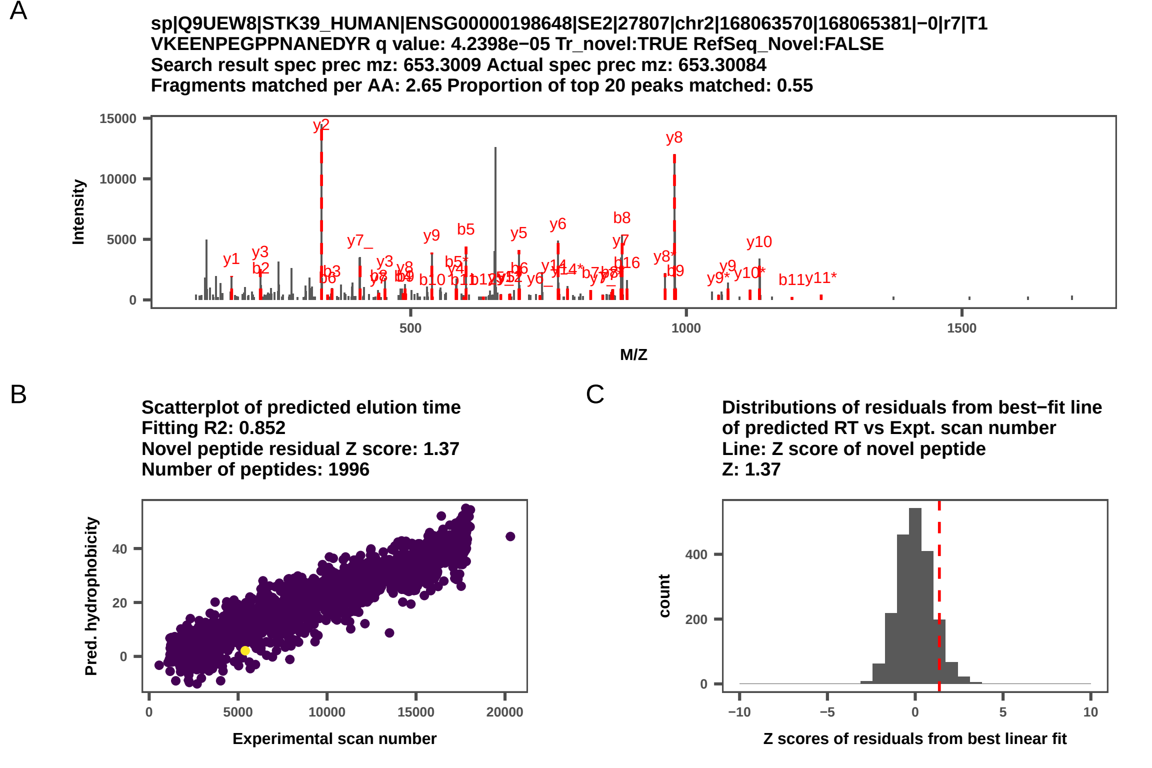

A
sp|Q9UEW8|STK39_HUMAN|ENSG00000198648|SE2|27807|chr2|168063570|168065381|−0|r7|T1
VKEENPEGPPNANEDYR q value: 4.2398e−05 Tr_novel:TRUE RefSeq_Novel:FALSE
Search result spec prec mz: 653.3009 Actual spec prec mz: 653.30084
Fragments matched per AA: 2.65 Proportion of top 20 peaks matched: 0.55
Intensity
15000
10000
5000
0
M/Z
500
1000
1500
y2
y8
b8
y6
y5
b5
y9
y7_
y10
y1
y3
b2
B
Scatterplot of predicted elution time
Fitting R2: 0.852
Novel peptide residual Z score: 1.37
Number of peptides: 1996
Pred. hydrophobicity
40
20
0
Experimental scan number
0
5000
10000
15000
20000
C
Distributions of residuals from best−fit line
of predicted RT vs Expt. scan number
Line: Z score of novel peptide
Z: 1.37
count
400
200
0
Z scores of residuals from best linear fit
−10
−5
0
5
10
