# Supplementary material for: Splice-Junction-Based Mapping of Alternative Isoforms in the Human Proteome
Source: Cell Rep. Author manuscript; Available in PMC 2020 Jan 15. (PMC6961840; doi:10.1016/j.celrep.2019.11.026)
Supplement: 2 [file NIHMS1546469-supplement-2.zip › DF1/PXD000561/Testis/Testis_4_PCBP2_YSTGSDSASFPHTTPSMCLNPDLEGPPLELTK.pdf]

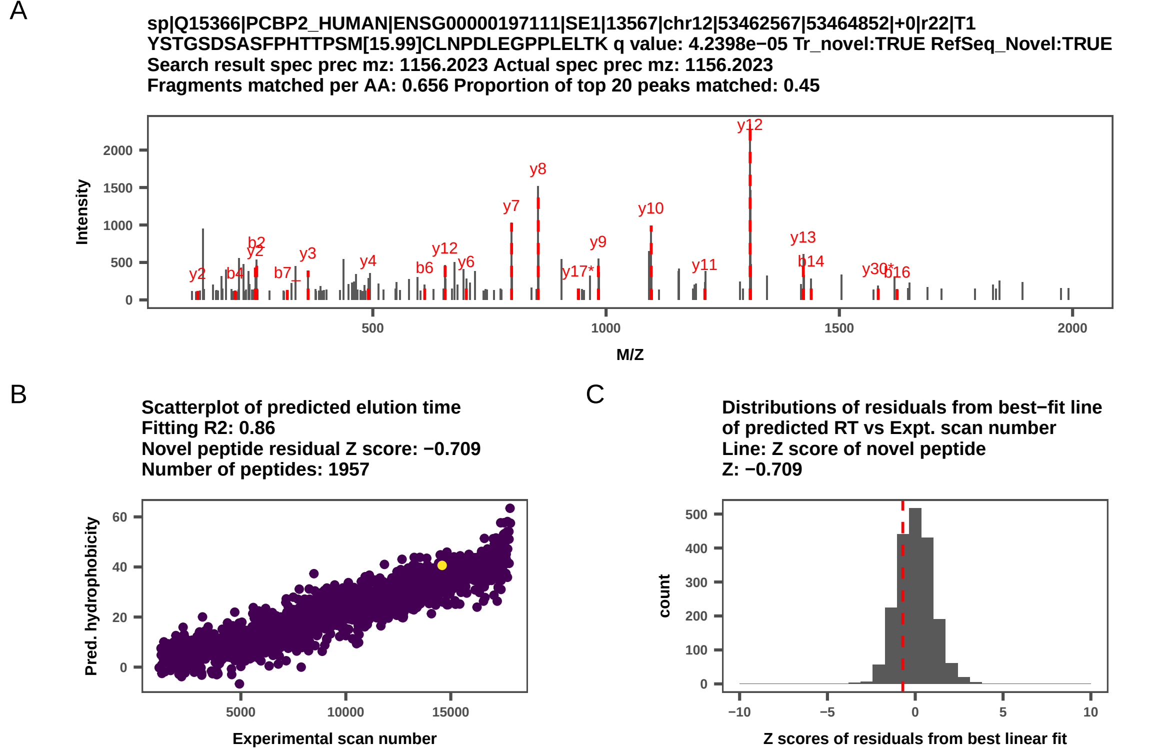
A
sp|Q15366|PCBP2_HUMAN|ENSG00000197111|SE1|13567|chr12|53462567|53464852|+0|r22|T1
YSTGSDSASFPHTTPSM[15.99]CLNPDLEGPPLELTK q value: 4.2398e−05 Tr_novel:TRUE RefSeq_Novel:TRUE
Search result spec prec mz: 1156.2023 Actual spec prec mz: 1156.2023
Fragments matched per AA: 0.656 Proportion of top 20 peaks matched: 0.45
Intensity
M/Z
y2
b2
y2
b4
b7
y3
y4
b6
y12
y6
y7
y8
y17*
y9
y10
y11
y12
y13
b14
y30*
b16
B
Scatterplot of predicted elution time
Fitting R2: 0.86
Novel peptide residual Z score: −0.709
Number of peptides: 1957
Pred. hydrophobicity
Experimental scan number
C
Distributions of residuals from best−fit line
of predicted RT vs Expt. scan number
Line: Z score of novel peptide
Z: −0.709
count
Z scores of residuals from best linear fit
