# Supplementary material for: Splice-Junction-Based Mapping of Alternative Isoforms in the Human Proteome
Source: Cell Rep. Author manuscript; Available in PMC 2020 Jan 15. (PMC6961840; doi:10.1016/j.celrep.2019.11.026)
Supplement: 2 [file NIHMS1546469-supplement-2.zip › DF1/PXD000561/Testis/Testis_18_ME3_PKEAMNVQTTSFSR.pdf]

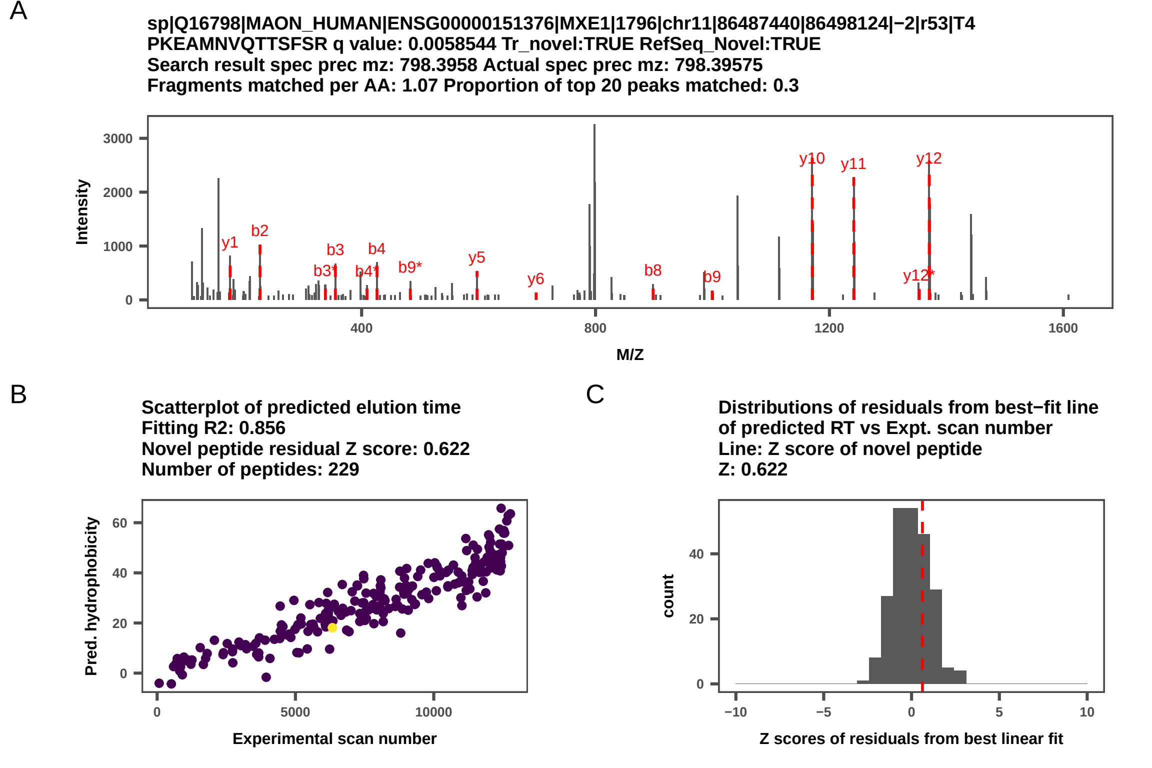
A
sp|Q16798|MAON_HUMAN|ENSG00000151376|MXE1|1796|chr11|86487440|86498124|−2|r53|T4
PKEAMNVQTTSFSR q value: 0.0058544 Tr_novel:TRUE RefSeq_Novel:TRUE
Search result spec prec mz: 798.3958 Actual spec prec mz: 798.39575
Fragments matched per AA: 1.07 Proportion of top 20 peaks matched: 0.3
Intensity
3000
2000
1000
0
y1
b2
b3
b3*
b4
b4*
b9*
y5
y6
b8
b9
y10
y11
y12
y12*
400
800
1200
1600
M/Z
B
Scatterplot of predicted elution time
Fitting R2: 0.856
Novel peptide residual Z score: 0.622
Number of peptides: 229
Pred. hydrophobicity
60
40
20
0
0
5000
10000
Experimental scan number
C
Distributions of residuals from best−fit line
of predicted RT vs Expt. scan number
Line: Z score of novel peptide
Z: 0.622
count
40
20
0
−10
−5
0
5
10
Z scores of residuals from best linear fit
