# Supplementary material for: Splice-Junction-Based Mapping of Alternative Isoforms in the Human Proteome
Source: Cell Rep. Author manuscript; Available in PMC 2020 Jan 15. (PMC6961840; doi:10.1016/j.celrep.2019.11.026)
Supplement: 2 [file NIHMS1546469-supplement-2.zip › DF1/PXD000561/Testis/Testis_15_LGALS1_GGSLNLKPGECLR.pdf]

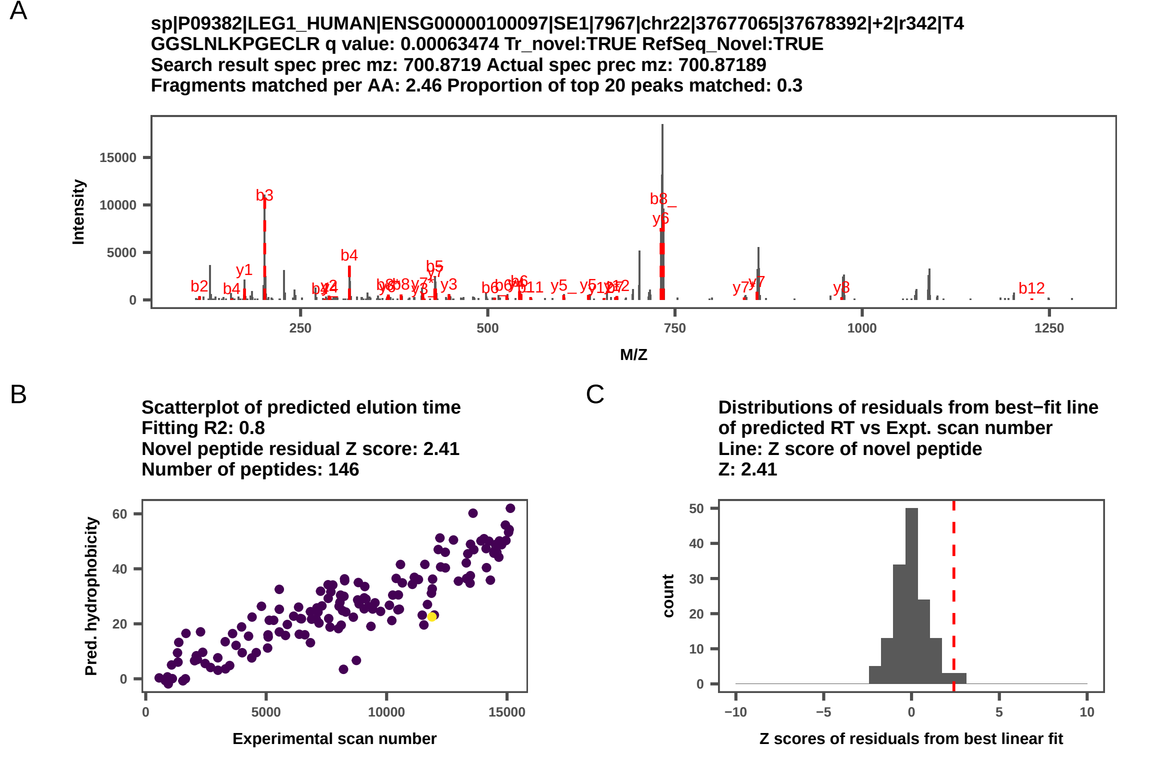
A
sp|P09382|LEG1_HUMAN|ENSG00000100097|SE1|7967|chr22|37677065|37678392|+2|r342|T4
GGSLNLKPGECLR q value: 0.00063474 Tr_novel:TRUE RefSeq_Novel:TRUE
Search result spec prec mz: 700.8719 Actual spec prec mz: 700.87189
Fragments matched per AA: 2.46 Proportion of top 20 peaks matched: 0.3
Intensity
15000
10000
5000
0
b2
b4
y1
b3
b4
b5
y3
b8_
y6
y7
y8
b12
250
500
750
1000
1250
M/Z
B
Scatterplot of predicted elution time
Fitting R2: 0.8
Novel peptide residual Z score: 2.41
Number of peptides: 146
Pred. hydrophobicity
60
40
20
0
0
5000
10000
15000
Experimental scan number
C
Distributions of residuals from best−fit line
of predicted RT vs Expt. scan number
Line: Z score of novel peptide
Z: 2.41
count
50
40
30
20
10
0
−10
−5
0
5
10
Z scores of residuals from best linear fit
