# Supplementary material for: Splice-Junction-Based Mapping of Alternative Isoforms in the Human Proteome
Source: Cell Rep. Author manuscript; Available in PMC 2020 Jan 15. (PMC6961840; doi:10.1016/j.celrep.2019.11.026)
Supplement: 2 [file NIHMS1546469-supplement-2.zip › DF1/PXD000561/Testis/Testis_2_KIAA1210_SLTATQVEPKEEEPNLPLVSEEEK.pdf]

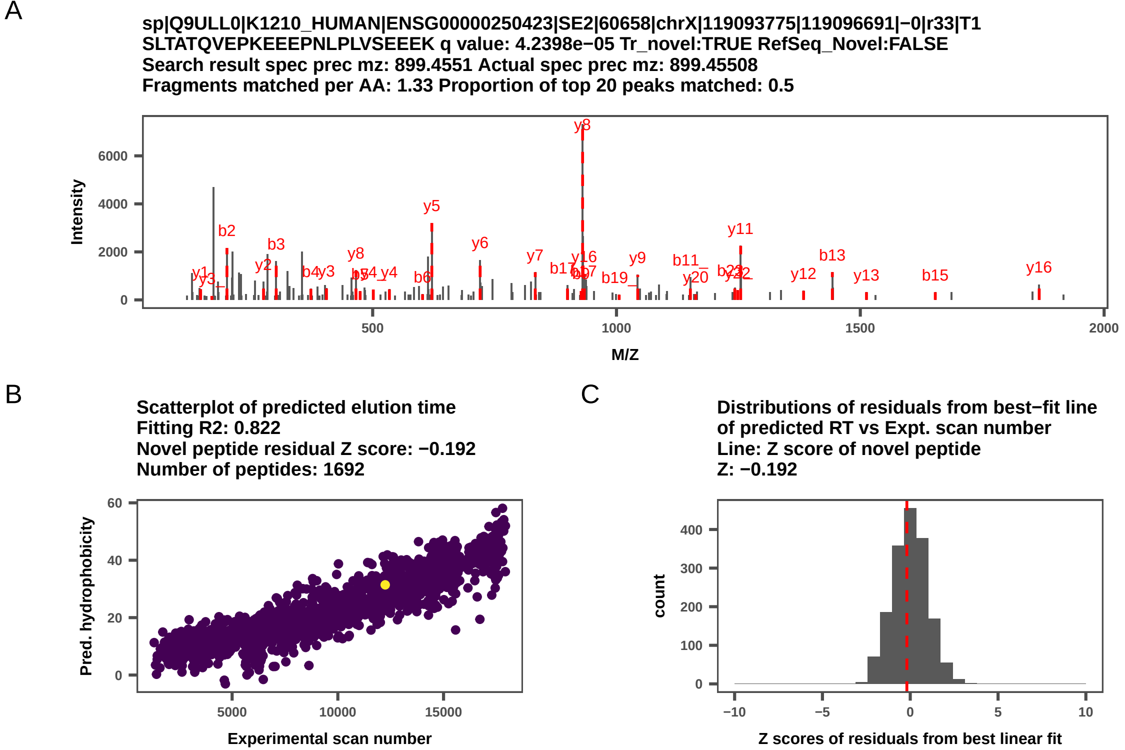
A
sp|Q9ULL0|K1210_HUMAN|ENSG00000250423|SE2|60658|chrX|119093775|119096691|−0|r33|T1
SLTATQVEPKEEEPNLPLVSEEEK q value: 4.2398e−05 Tr_novel:TRUE RefSeq_Novel:FALSE
Search result spec prec mz: 899.4551 Actual spec prec mz: 899.45508
Fragments matched per AA: 1.33 Proportion of top 20 peaks matched: 0.5
Intensity
0
2000
4000
6000
M/Z
500
1000
1500
2000
y1
y3
b2
y2
b3
b4
y3
y8
y4
y5
b6
y6
y7
y16
y9
b11
y20
y11
y12
b13
y13
b15
y16
b19
B
Scatterplot of predicted elution time
Fitting R2: 0.822
Novel peptide residual Z score: −0.192
Number of peptides: 1692
Pred. hydrophobicity
0
20
40
60
Experimental scan number
5000
10000
15000
C
Distributions of residuals from best−fit line
of predicted RT vs Expt. scan number
Line: Z score of novel peptide
Z: −0.192
count
0
100
200
300
400
Z scores of residuals from best linear fit
−10
−5
0
5
10
