# Supplementary material for: Splice-Junction-Based Mapping of Alternative Isoforms in the Human Proteome
Source: Cell Rep. Author manuscript; Available in PMC 2020 Jan 15. (PMC6961840; doi:10.1016/j.celrep.2019.11.026)
Supplement: 2 [file NIHMS1546469-supplement-2.zip › DF1/PXD000561/Testis/Testis_19_RBBP4_GPDAVEERVINEEYK.pdf]

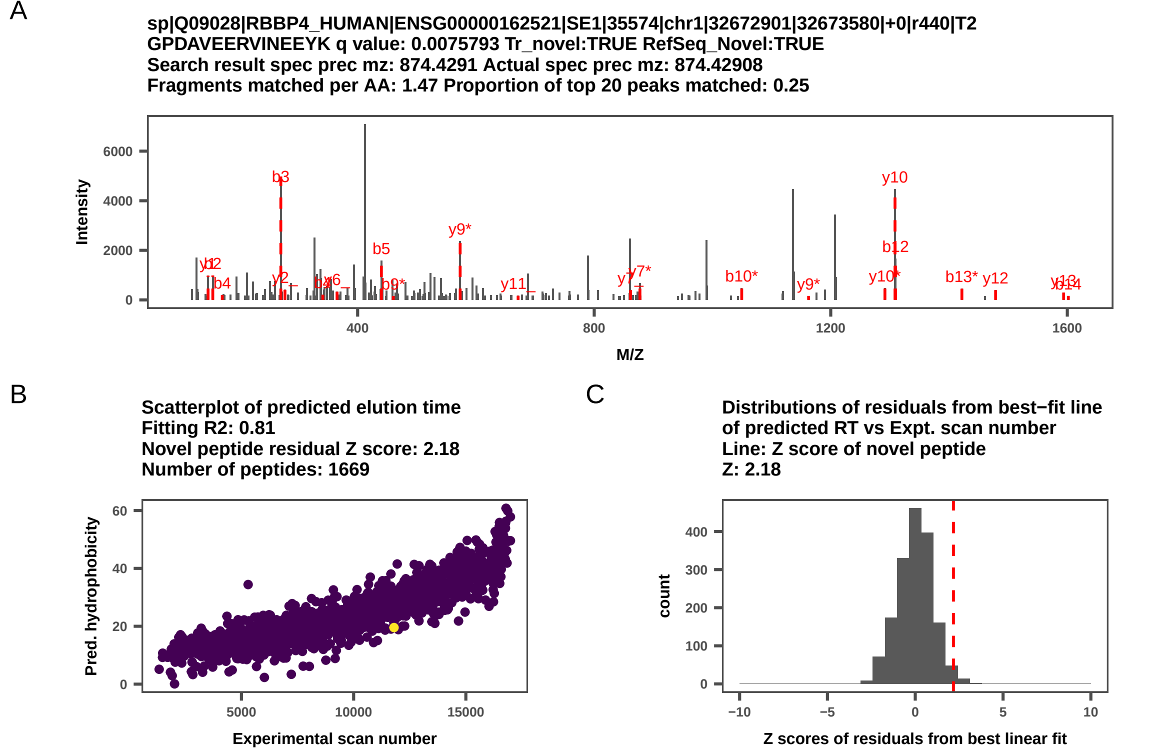
A
sp|Q09028|RBBP4_HUMAN|ENSG00000162521|SE1|35574|chr1|32672901|32673580|+0|r440|T2
GPDAVEERVINEEYK q value: 0.0075793 Tr_novel:TRUE RefSeq_Novel:TRUE
Search result spec prec mz: 874.4291 Actual spec prec mz: 874.42908
Fragments matched per AA: 1.47 Proportion of top 20 peaks matched: 0.25
Intensity
M/Z
b3
y10
y9*
b5
b12
b4
y2
y6
b9*
y11
y7*
b10*
y9*
y10*
b13*
y12
b14
0
2000
4000
6000
400
800
1200
1600
B
Scatterplot of predicted elution time
Fitting R2: 0.81
Novel peptide residual Z score: 2.18
Number of peptides: 1669
Pred. hydrophobicity
Experimental scan number
0
20
40
60
5000
10000
15000
C
Distributions of residuals from best−fit line
of predicted RT vs Expt. scan number
Line: Z score of novel peptide
Z: 2.18
count
Z scores of residuals from best linear fit
0
100
200
300
400
−10
−5
0
5
10
