# Supplementary material for: Splice-Junction-Based Mapping of Alternative Isoforms in the Human Proteome
Source: Cell Rep. Author manuscript; Available in PMC 2020 Jan 15. (PMC6961840; doi:10.1016/j.celrep.2019.11.026)
Supplement: 2 [file NIHMS1546469-supplement-2.zip › DF1/PXD000561/Testis/Testis_11_SMARCC1_PGDVQGPGTAVGATGSGIAAAAAGLAVYR.pdf]

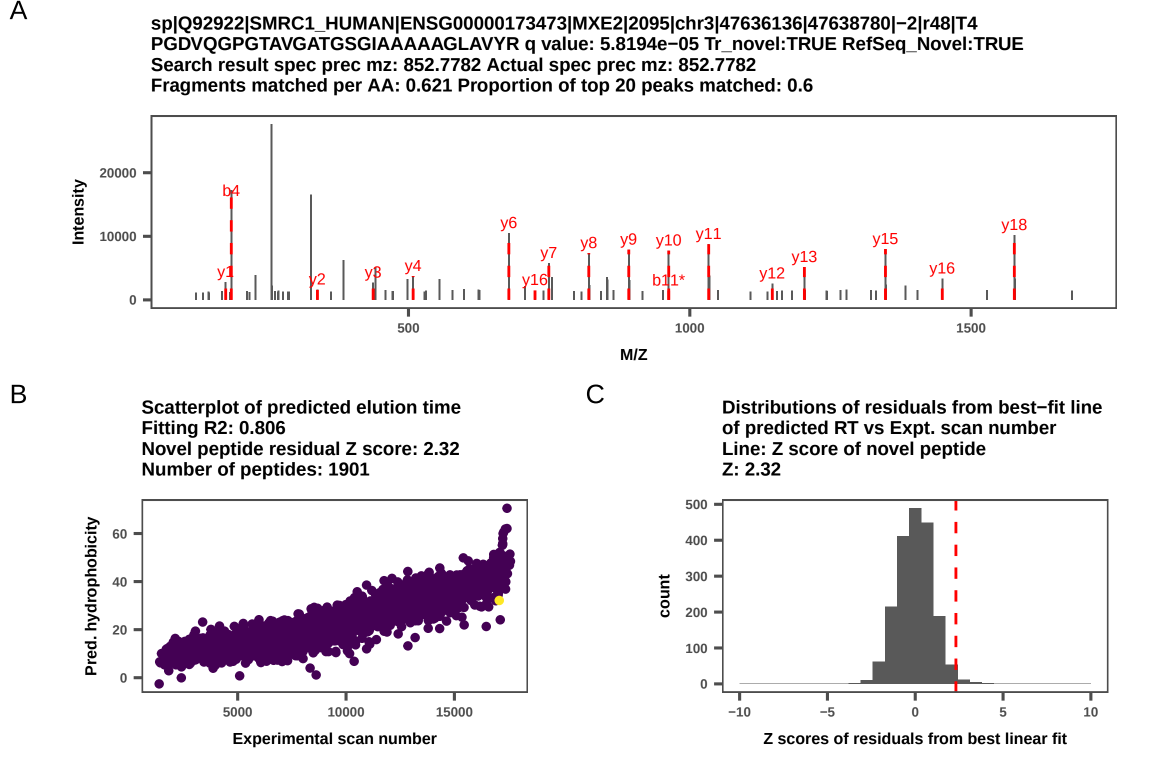
A
sp|Q92922|SMRC1_HUMAN|ENSG00000173473|MXE2|2095|chr3|47636136|47638780|−2|r48|T4
PGDVQGPGTAVGATGSGIAAAAAGLAVYR q value: 5.8194e−05 Tr_novel:TRUE RefSeq_Novel:TRUE
Search result spec prec mz: 852.7782 Actual spec prec mz: 852.7782
Fragments matched per AA: 0.621 Proportion of top 20 peaks matched: 0.6
Intensity
M/Z
b4
y1
y2
y3
y4
y6
y16
y7
y8
y9
y10
b11*
y11
y12
y13
y15
y16
y18
B
Scatterplot of predicted elution time
Fitting R2: 0.806
Novel peptide residual Z score: 2.32
Number of peptides: 1901
Pred. hydrophobicity
Experimental scan number
C
Distributions of residuals from best−fit line
of predicted RT vs Expt. scan number
Line: Z score of novel peptide
Z: 2.32
count
Z scores of residuals from best linear fit
