# Supplementary material for: Splice-Junction-Based Mapping of Alternative Isoforms in the Human Proteome
Source: Cell Rep. Author manuscript; Available in PMC 2020 Jan 15. (PMC6961840; doi:10.1016/j.celrep.2019.11.026)
Supplement: 2 [file NIHMS1546469-supplement-2.zip › DF1/PXD000561/Testis/Testis_10_ART3_NQKLEDHAPGPVPVPGPK.pdf]

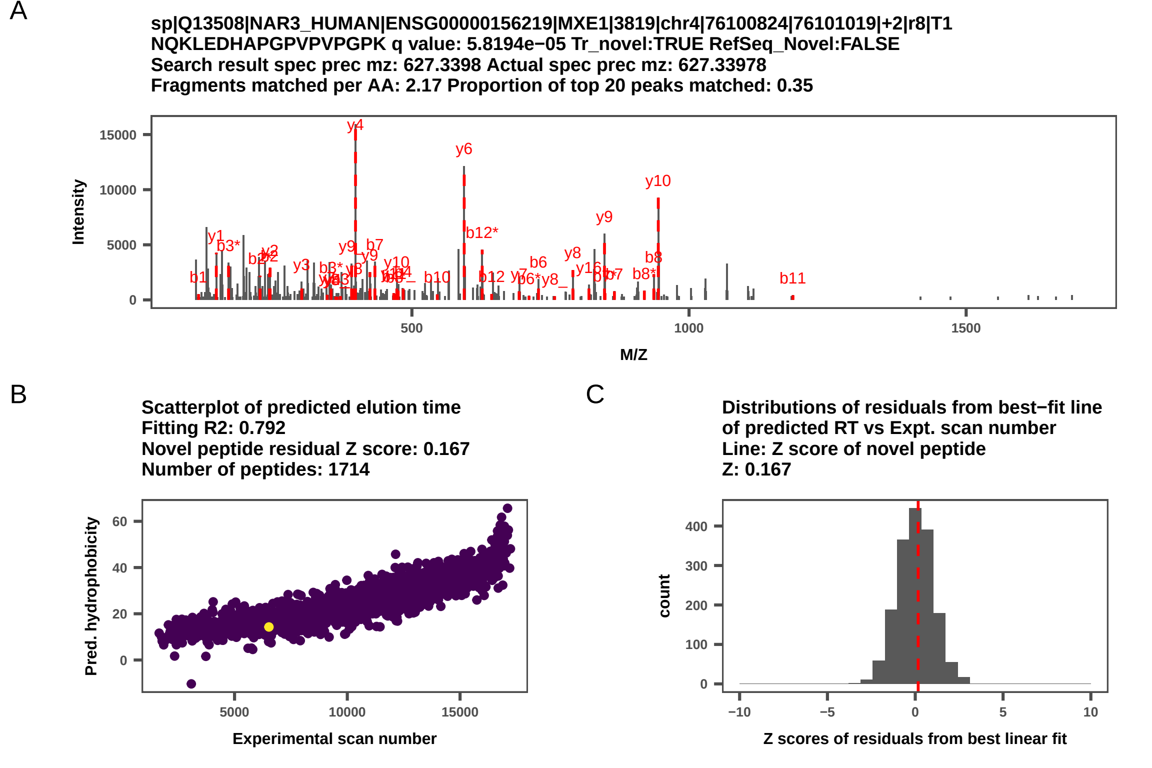
A
sp|Q13508|NAR3_HUMAN|ENSG00000156219|MXE1|3819|chr4|76100824|76101019|+2|r8|T1
NQKLEDHAPGPVPVPGPK q value: 5.8194e−05 Tr_novel:TRUE RefSeq_Novel:FALSE
Search result spec prec mz: 627.3398 Actual spec prec mz: 627.33978
Fragments matched per AA: 2.17 Proportion of top 20 peaks matched: 0.35
Intensity
15000
10000
5000
0
M/Z
500
1000
1500
y4
y6
y10
y9
b12*
y1
b3*
b1
y3
b7
b10
b12
b6
y8
b8
b8*
b11
B
Scatterplot of predicted elution time
Fitting R2: 0.792
Novel peptide residual Z score: 0.167
Number of peptides: 1714
Pred. hydrophobicity
60
40
20
0
Experimental scan number
5000
10000
15000
C
Distributions of residuals from best−fit line
of predicted RT vs Expt. scan number
Line: Z score of novel peptide
Z: 0.167
count
400
300
200
100
0
Z scores of residuals from best linear fit
−10
−5
0
5
10
