# Supplementary material for: Splice-Junction-Based Mapping of Alternative Isoforms in the Human Proteome
Source: Cell Rep. Author manuscript; Available in PMC 2020 Jan 15. (PMC6961840; doi:10.1016/j.celrep.2019.11.026)
Supplement: 2 [file NIHMS1546469-supplement-2.zip › DF1/PXD000561/Testis/Testis_5_ART3_LEDHAPGPVPVPGPK.pdf]

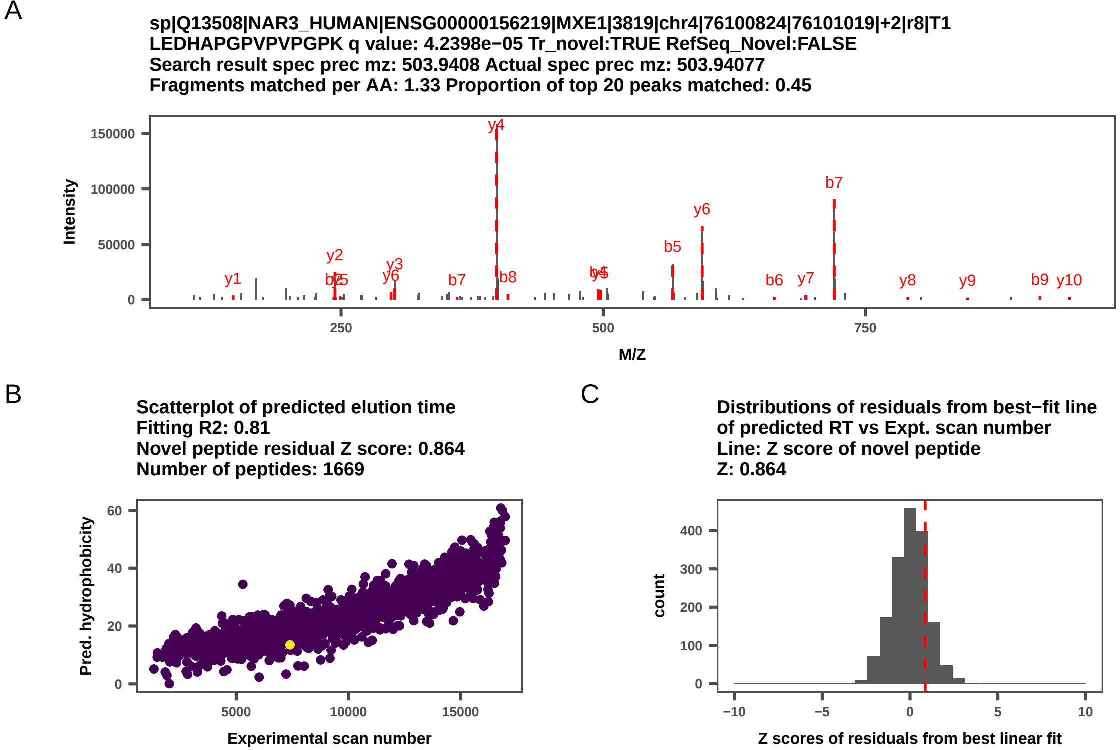
A
sp|Q13508|NAR3_HUMAN|ENSG00000156219|MXE1|3819|chr4|76100824|76101019|+2|r8|T1
LEDHAPGPVPVPGPK q value: 4.2398e−05 Tr_novel:TRUE RefSeq_Novel:FALSE
Search result spec prec mz: 503.9408 Actual spec prec mz: 503.94077
Fragments matched per AA: 1.33 Proportion of top 20 peaks matched: 0.45
Intensity
150000
100000
50000
0
250
500
750
M/Z
y1
y2
b2
y5
y3
y6
b7
y4
b8
b4
y5
b5
y6
b6
y7
b7
y8
y9
b9
y10
B
Scatterplot of predicted elution time
Fitting R2: 0.81
Novel peptide residual Z score: 0.864
Number of peptides: 1669
Pred. hydrophobicity
60
40
20
0
5000
10000
15000
Experimental scan number
C
Distributions of residuals from best−fit line
of predicted RT vs Expt. scan number
Line: Z score of novel peptide
Z: 0.864
count
400
300
200
100
0
−10
−5
0
5
10
Z scores of residuals from best linear fit
