# Supplementary material for: Splice-Junction-Based Mapping of Alternative Isoforms in the Human Proteome
Source: Cell Rep. Author manuscript; Available in PMC 2020 Jan 15. (PMC6961840; doi:10.1016/j.celrep.2019.11.026)
Supplement: 2 [file NIHMS1546469-supplement-2.zip › DF1/PXD000561/Testis/Testis_13_CD99_GEEEQGEVDMESHR.pdf]

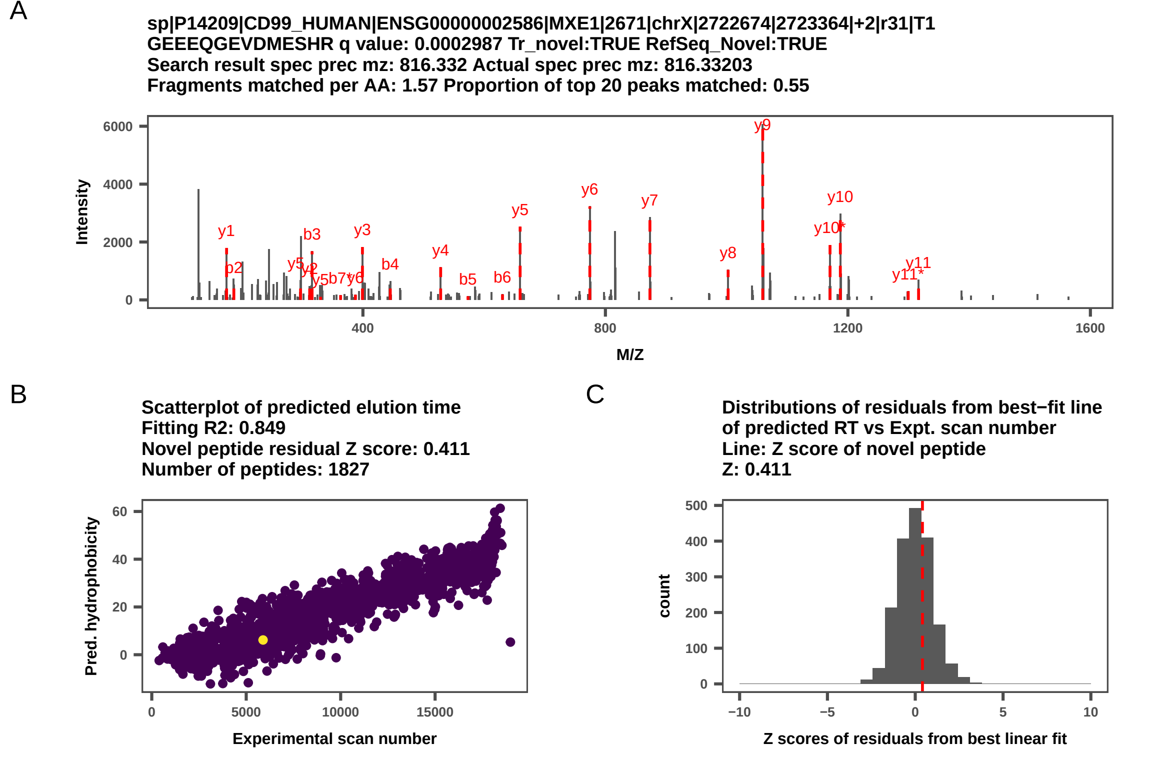
A
sp|P14209|CD99_HUMAN|ENSG00000002586|MXE1|2671|chrX|2722674|2723364|+2|r31|T1
GEEEQGEVDMESHR q value: 0.0002987 Tr_novel:TRUE RefSeq_Novel:TRUE
Search result spec prec mz: 816.332 Actual spec prec mz: 816.33203
Fragments matched per AA: 1.57 Proportion of top 20 peaks matched: 0.55
Intensity
6000
4000
2000
0
M/Z
400
800
1200
1600
y1
b2
b3
y3
b4
y4
b5
b6
y5
y6
y7
y8
y9
y10
y10*
y11
y11*
B
Scatterplot of predicted elution time
Fitting R2: 0.849
Novel peptide residual Z score: 0.411
Number of peptides: 1827
Pred. hydrophobicity
60
40
20
0
0
5000
10000
15000
Experimental scan number
C
Distributions of residuals from best−fit line
of predicted RT vs Expt. scan number
Line: Z score of novel peptide
Z: 0.411
count
500
400
300
200
100
0
−10
−5
0
5
10
Z scores of residuals from best linear fit
