# Supplementary material for: Splice-Junction-Based Mapping of Alternative Isoforms in the Human Proteome
Source: Cell Rep. Author manuscript; Available in PMC 2020 Jan 15. (PMC6961840; doi:10.1016/j.celrep.2019.11.026)
Supplement: 2 [file NIHMS1546469-supplement-2.zip › DF1/PXD000561/Testis/Testis_17_C1orf167_TLSYQWEPGACPAAPR.pdf]

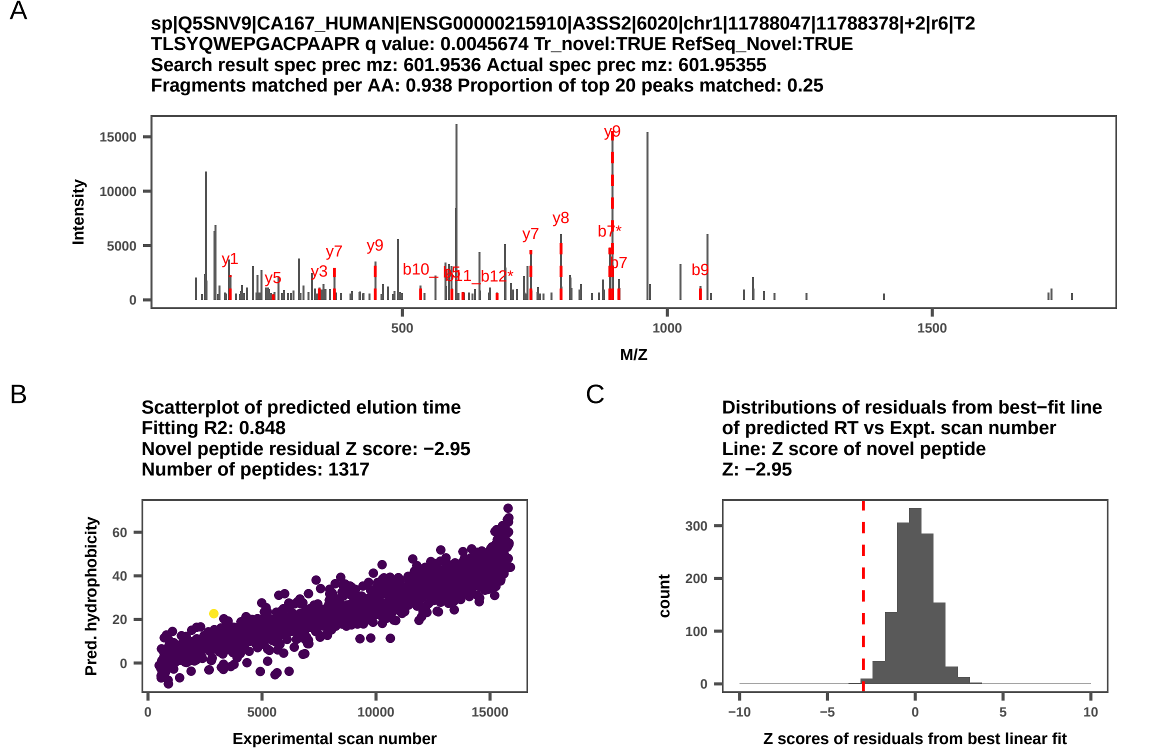
A
sp|Q5SNV9|CA167_HUMAN|ENSG00000215910|A3SS2|6020|chr1|11788047|11788378|+2|r6|T2
TLSYQWEPGACPAAPR q value: 0.0045674 Tr_novel:TRUE RefSeq_Novel:TRUE
Search result spec prec mz: 601.9536 Actual spec prec mz: 601.95355
Fragments matched per AA: 0.938 Proportion of top 20 peaks matched: 0.25
Intensity
15000
10000
5000
0
y1
y5
y3
y7
y9
b10_
b12*
y7
y8
y9
b7*
b7
b9
500
1000
1500
M/Z
B
Scatterplot of predicted elution time
Fitting R2: 0.848
Novel peptide residual Z score: −2.95
Number of peptides: 1317
Pred. hydrophobicity
60
40
20
0
0
5000
10000
15000
Experimental scan number
C
Distributions of residuals from best−fit line
of predicted RT vs Expt. scan number
Line: Z score of novel peptide
Z: −2.95
count
300
200
100
0
−10
−5
0
5
10
Z scores of residuals from best linear fit
