# Supplementary material for: Splice-Junction-Based Mapping of Alternative Isoforms in the Human Proteome
Source: Cell Rep. Author manuscript; Available in PMC 2020 Jan 15. (PMC6961840; doi:10.1016/j.celrep.2019.11.026)
Supplement: 2 [file NIHMS1546469-supplement-2.zip › DF1/PXD000561/Testis/Testis_1_SRRM1_KVELSESEDKGGK.pdf]

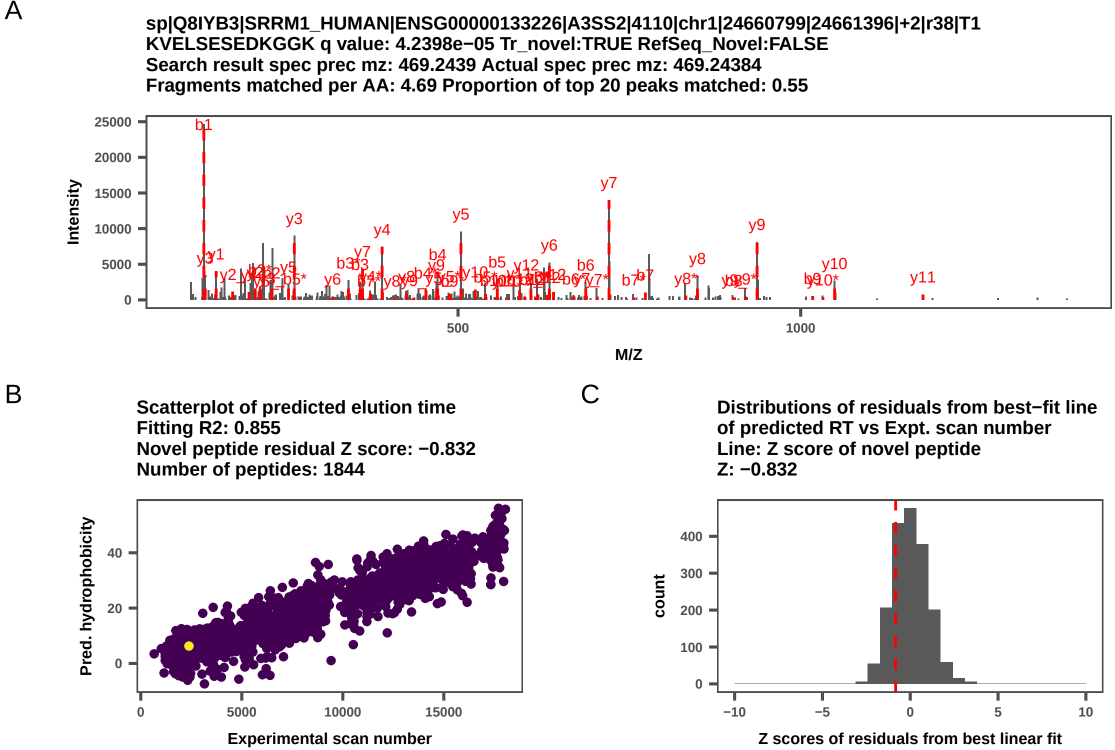

A
sp|Q8IYB3|SRRM1_HUMAN|ENSG00000133226|A3SS2|4110|chr1|24660799|24661396|+2|r38|T1
KVELSESEDKGGK q value: 4.2398e−05 Tr_novel:TRUE RefSeq_Novel:FALSE
Search result spec prec mz: 469.2439 Actual spec prec mz: 469.24384
Fragments matched per AA: 4.69 Proportion of top 20 peaks matched: 0.55
Intensity
25000
20000
15000
10000
5000
0
b1
y3
y4
y5
y7
y9
y6
y8
y10
y11
500
1000
M/Z
B
Scatterplot of predicted elution time
Fitting R2: 0.855
Novel peptide residual Z score: −0.832
Number of peptides: 1844
Pred. hydrophobicity
40
20
0
0
5000
10000
15000
Experimental scan number
C
Distributions of residuals from best−fit line
of predicted RT vs Expt. scan number
Line: Z score of novel peptide
Z: −0.832
count
400
300
200
100
0
−10
−5
0
5
10
Z scores of residuals from best linear fit
