# Supplementary material for: Splice-Junction-Based Mapping of Alternative Isoforms in the Human Proteome
Source: Cell Rep. Author manuscript; Available in PMC 2020 Jan 15. (PMC6961840; doi:10.1016/j.celrep.2019.11.026)
Supplement: 2 [file NIHMS1546469-supplement-2.zip › DF1/PXD000561/Testis/Testis_12_SYPL1_ETSLHSPSNTSAPHSQGGIPPPTGL.pdf]

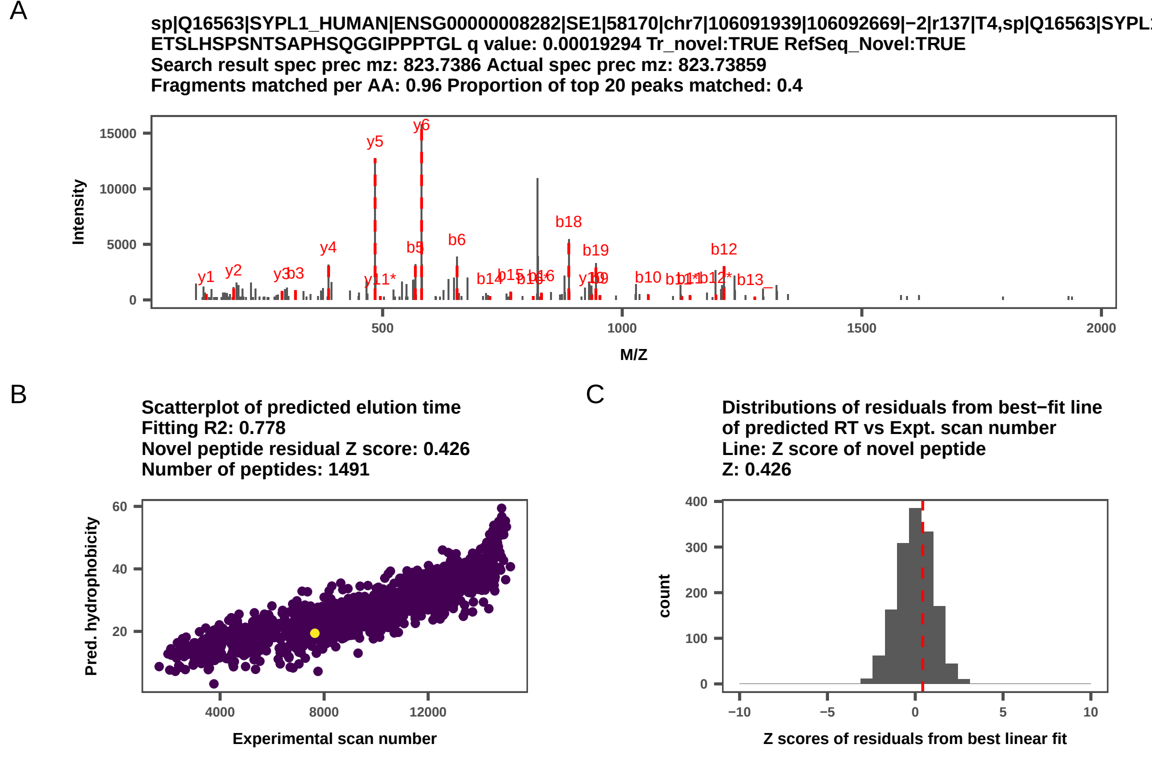
A
sp|Q16563|SYPL1_HUMAN|ENSG00000008282|SE1|58170|chr7|106091939|106092669|−2|r137|T4,sp|Q16563|SYPL
ETSLHSPSNTSAPHSQGGIPPPTGL q value: 0.00019294 Tr_novel:TRUE RefSeq_Novel:TRUE
Search result spec prec mz: 823.7386 Actual spec prec mz: 823.73859
Fragments matched per AA: 0.96 Proportion of top 20 peaks matched: 0.4
Intensity
15000
10000
5000
0
y1
y2
b3
y4
y5
y6
y11*
b5
b6
b14
b15
b16
b18
b19
b10
b12
b12*
b13
500
1000
1500
2000
M/Z
B
Scatterplot of predicted elution time
Fitting R2: 0.778
Novel peptide residual Z score: 0.426
Number of peptides: 1491
Pred. hydrophobicity
60
40
20
4000
8000
12000
Experimental scan number
C
Distributions of residuals from best−fit line
of predicted RT vs Expt. scan number
Line: Z score of novel peptide
Z: 0.426
count
400
300
200
100
0
−10
−5
0
5
10
Z scores of residuals from best linear fit
