# Supplementary material for: Splice-Junction-Based Mapping of Alternative Isoforms in the Human Proteome
Source: Cell Rep. Author manuscript; Available in PMC 2020 Jan 15. (PMC6961840; doi:10.1016/j.celrep.2019.11.026)
Supplement: 2 [file NIHMS1546469-supplement-2.zip › DF1/PXD000561/Testis/Testis_16_KIAA1210_NLQVIVEPK.pdf]

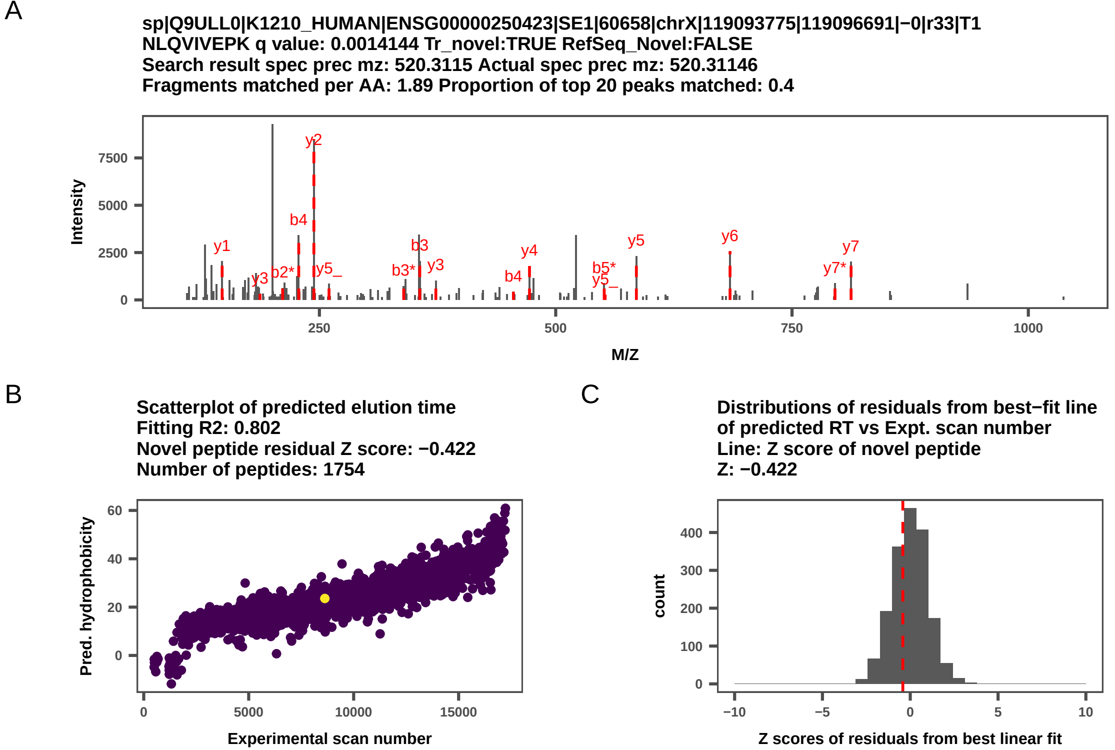
A
sp|Q9ULL0|K1210_HUMAN|ENSG00000250423|SE1|60658|chrX|119093775|119096691|−0|r33|T1
NLQVIVEPK q value: 0.0014144 Tr_novel:TRUE RefSeq_Novel:FALSE
Search result spec prec mz: 520.3115 Actual spec prec mz: 520.31146
Fragments matched per AA: 1.89 Proportion of top 20 peaks matched: 0.4
Intensity
7500
5000
2500
0
y1
y3
b2*
b4
y2
y5_
b3*
b3
y3
b4
y4
b5*
y5_
y5
y6
y7*
y7
250
500
750
1000
M/Z
B
Scatterplot of predicted elution time
Fitting R2: 0.802
Novel peptide residual Z score: −0.422
Number of peptides: 1754
Pred. hydrophobicity
60
40
20
0
0
5000
10000
15000
Experimental scan number
C
Distributions of residuals from best−fit line
of predicted RT vs Expt. scan number
Line: Z score of novel peptide
Z: −0.422
count
400
300
200
100
0
−10
−5
0
5
10
Z scores of residuals from best linear fit
