# Supplementary material for: Splice-Junction-Based Mapping of Alternative Isoforms in the Human Proteome
Source: Cell Rep. Author manuscript; Available in PMC 2020 Jan 15. (PMC6961840; doi:10.1016/j.celrep.2019.11.026)
Supplement: 2 [file NIHMS1546469-supplement-2.zip › DF1/PXD000561/Lung/Lung_2_PSMD2_GFGGSGSQVDSAR.pdf]

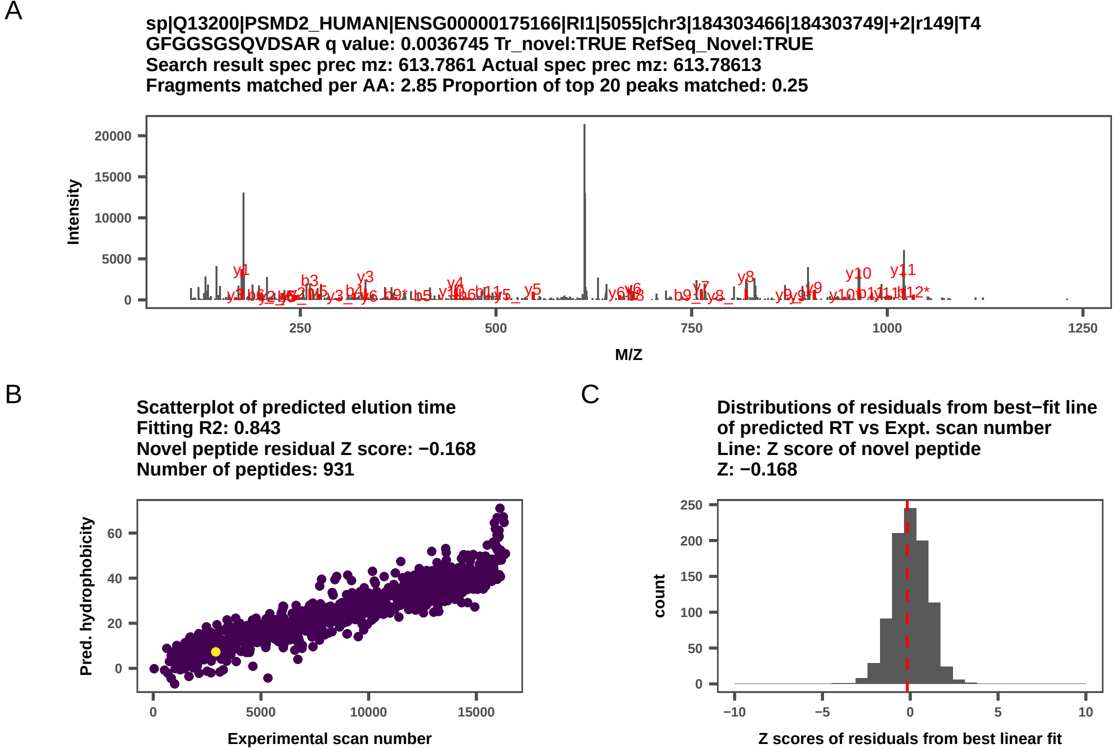
A
sp|Q13200|PSMD2_HUMAN|ENSG00000175166|RI1|5055|chr3|184303466|184303749|+2|r149|T4
GFGGSGSQVDSAR q value: 0.0036745 Tr_novel:TRUE RefSeq_Novel:TRUE
Search result spec prec mz: 613.7861 Actual spec prec mz: 613.78613
Fragments matched per AA: 2.85 Proportion of top 20 peaks matched: 0.25
Intensity
20000
15000
10000
5000
0
y1
b3
y3
y4
y5
y6
y7
y8
y9
y10
y11
b12*
250
500
750
1000
1250
M/Z
B
Scatterplot of predicted elution time
Fitting R2: 0.843
Novel peptide residual Z score: −0.168
Number of peptides: 931
Pred. hydrophobicity
60
40
20
0
0
5000
10000
15000
Experimental scan number
C
Distributions of residuals from best−fit line
of predicted RT vs Expt. scan number
Line: Z score of novel peptide
Z: −0.168
count
250
200
150
100
50
0
−10
−5
0
5
10
Z scores of residuals from best linear fit
