# Supplementary material for: Splice-Junction-Based Mapping of Alternative Isoforms in the Human Proteome
Source: Cell Rep. Author manuscript; Available in PMC 2020 Jan 15. (PMC6961840; doi:10.1016/j.celrep.2019.11.026)
Supplement: 2 [file NIHMS1546469-supplement-2.zip › DF1/PXD000561/Lung/Lung_3_SLC35E1_LLYDPHLGSGGPLGFPGQQR.pdf]

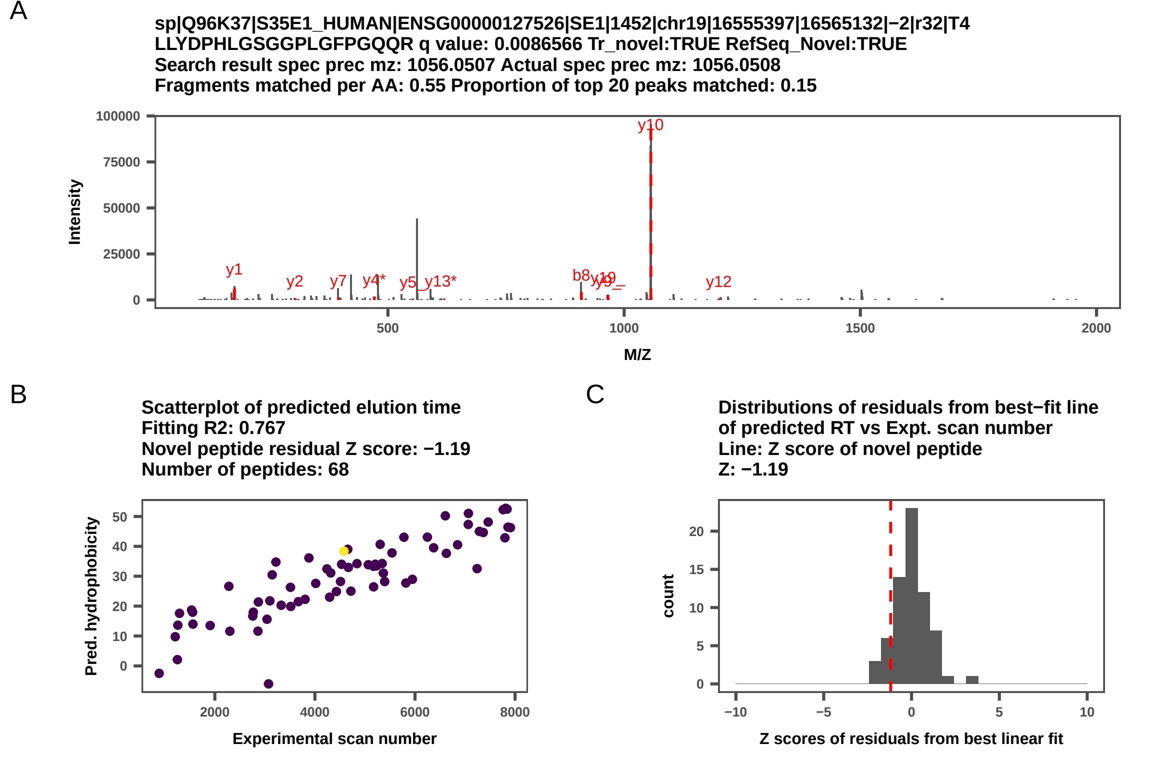
A
sp|Q96K37|S35E1_HUMAN|ENSG00000127526|SE1|1452|chr19|16555397|16565132|−2|r32|T4
LLYDPHLGSGGPLGFPGQQR q value: 0.0086566 Tr_novel:TRUE RefSeq_Novel:TRUE
Search result spec prec mz: 1056.0507 Actual spec prec mz: 1056.0508
Fragments matched per AA: 0.55 Proportion of top 20 peaks matched: 0.15
Intensity
100000
75000
50000
25000
0
y10
y1
y2
y7
y4*
y5_y13*
b8
y19
y9_
y12
500
1000
1500
2000
M/Z
B
Scatterplot of predicted elution time
Fitting R2: 0.767
Novel peptide residual Z score: −1.19
Number of peptides: 68
Pred. hydrophobicity
50
40
30
20
10
0
2000
4000
6000
8000
Experimental scan number
C
Distributions of residuals from best−fit line
of predicted RT vs Expt. scan number
Line: Z score of novel peptide
Z: −1.19
count
20
15
10
5
0
−10
−5
0
5
10
Z scores of residuals from best linear fit
