# Supplementary material for: Splice-Junction-Based Mapping of Alternative Isoforms in the Human Proteome
Source: Cell Rep. Author manuscript; Available in PMC 2020 Jan 15. (PMC6961840; doi:10.1016/j.celrep.2019.11.026)
Supplement: 2 [file NIHMS1546469-supplement-2.zip › DF1/PXD000561/AdrenalGland/AdrenalGland_3_SYPL1_ETSLHSPSNTSAPHSQGGIPPPTGL.pdf]

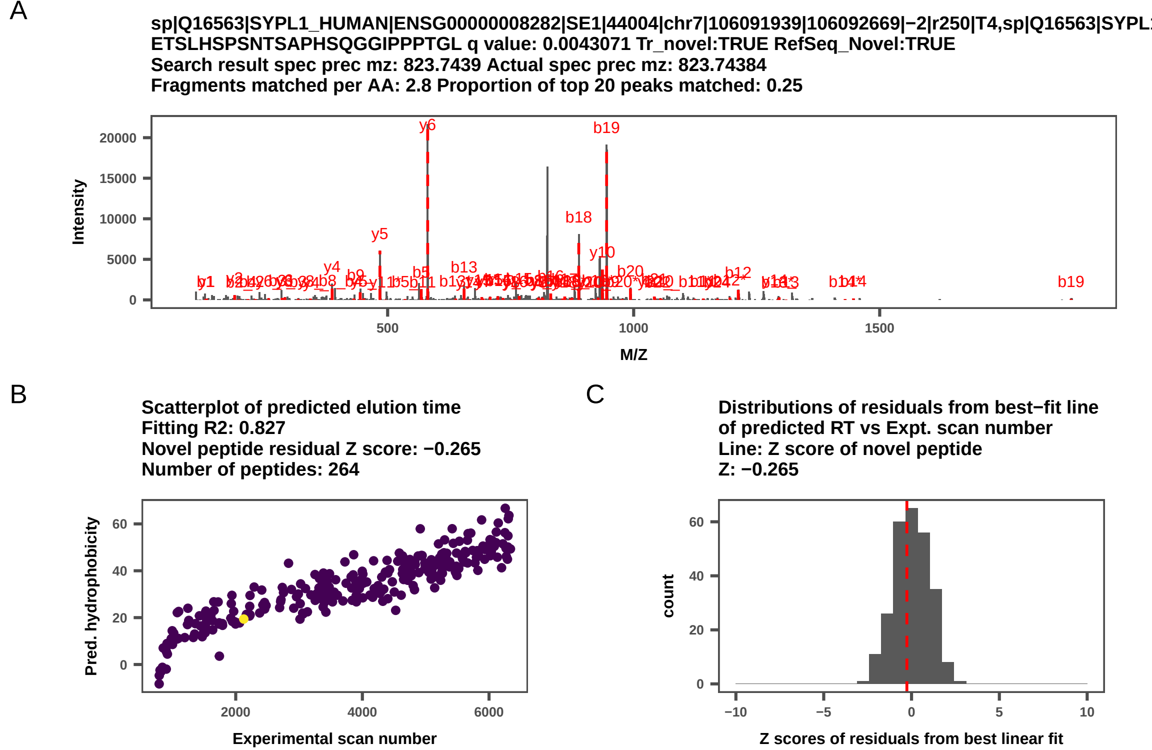
A
sp|Q16563|SYPL1_HUMAN|ENSG00000008282|SE1|44004|chr7|106091939|106092669|−2|r250|T4,sp|Q16563|SYPL
ETSLHSPSNTSAPHSQGGIPPPTGL q value: 0.0043071 Tr_novel:TRUE RefSeq_Novel:TRUE
Search result spec prec mz: 823.7439 Actual spec prec mz: 823.74384
Fragments matched per AA: 2.8 Proportion of top 20 peaks matched: 0.25
Intensity
20000
15000
10000
5000
0
y6
b19
b18
y5
y10
y4
b13
b20
b12
y1
y2
b9
b5
b19
500
1000
1500
M/Z
B
Scatterplot of predicted elution time
Fitting R2: 0.827
Novel peptide residual Z score: −0.265
Number of peptides: 264
Pred. hydrophobicity
60
40
20
0
2000
4000
6000
Experimental scan number
C
Distributions of residuals from best−fit line
of predicted RT vs Expt. scan number
Line: Z score of novel peptide
Z: −0.265
count
60
40
20
0
−10
−5
0
5
10
Z scores of residuals from best linear fit
