# Supplementary material for: Splice-Junction-Based Mapping of Alternative Isoforms in the Human Proteome
Source: Cell Rep. Author manuscript; Available in PMC 2020 Jan 15. (PMC6961840; doi:10.1016/j.celrep.2019.11.026)
Supplement: 2 [file NIHMS1546469-supplement-2.zip › DF1/PXD000561/AdrenalGland/AdrenalGland_6_COMMD7_VGGPVPEAVGGDMQQLNQLGAQQFSALTEVLFHFLTEPK.pdf]

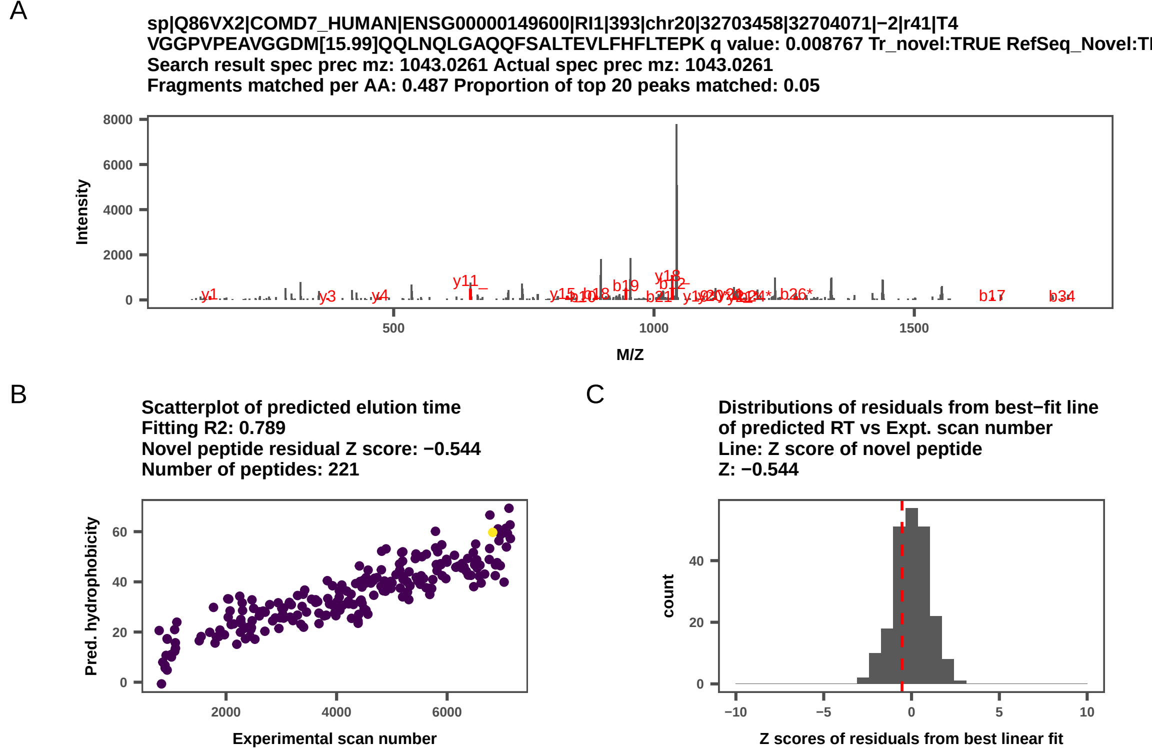
A
sp|Q86VX2|COMD7_HUMAN|ENSG00000149600|RI1|393|chr20|32703458|32704071|−2|r41|T4
VGGPVPEAVGGDM[15.99]QQLNQLGAQQFSALTEVLFHFLTEPK q value: 0.008767 Tr_novel:TRUE RefSeq_Novel:TI
Search result spec prec mz: 1043.0261 Actual spec prec mz: 1043.0261
Fragments matched per AA: 0.487 Proportion of top 20 peaks matched: 0.05
Intensity
8000
6000
4000
2000
0
y1
y3
y4
y11_
y15
b19
y18
b21
b26*
b17
b34
500
1000
1500
M/Z
B
Scatterplot of predicted elution time
Fitting R2: 0.789
Novel peptide residual Z score: −0.544
Number of peptides: 221
Pred. hydrophobicity
60
40
20
0
2000
4000
6000
Experimental scan number
C
Distributions of residuals from best−fit line
of predicted RT vs Expt. scan number
Line: Z score of novel peptide
Z: −0.544
count
40
20
0
−10
−5
0
5
10
Z scores of residuals from best linear fit
