# Supplementary material for: Splice-Junction-Based Mapping of Alternative Isoforms in the Human Proteome
Source: Cell Rep. Author manuscript; Available in PMC 2020 Jan 15. (PMC6961840; doi:10.1016/j.celrep.2019.11.026)
Supplement: 2 [file NIHMS1546469-supplement-2.zip › DF1/PXD000561/AdrenalGland/AdrenalGland_1_RBBP4_GPDAVEERVINEEYK.pdf]

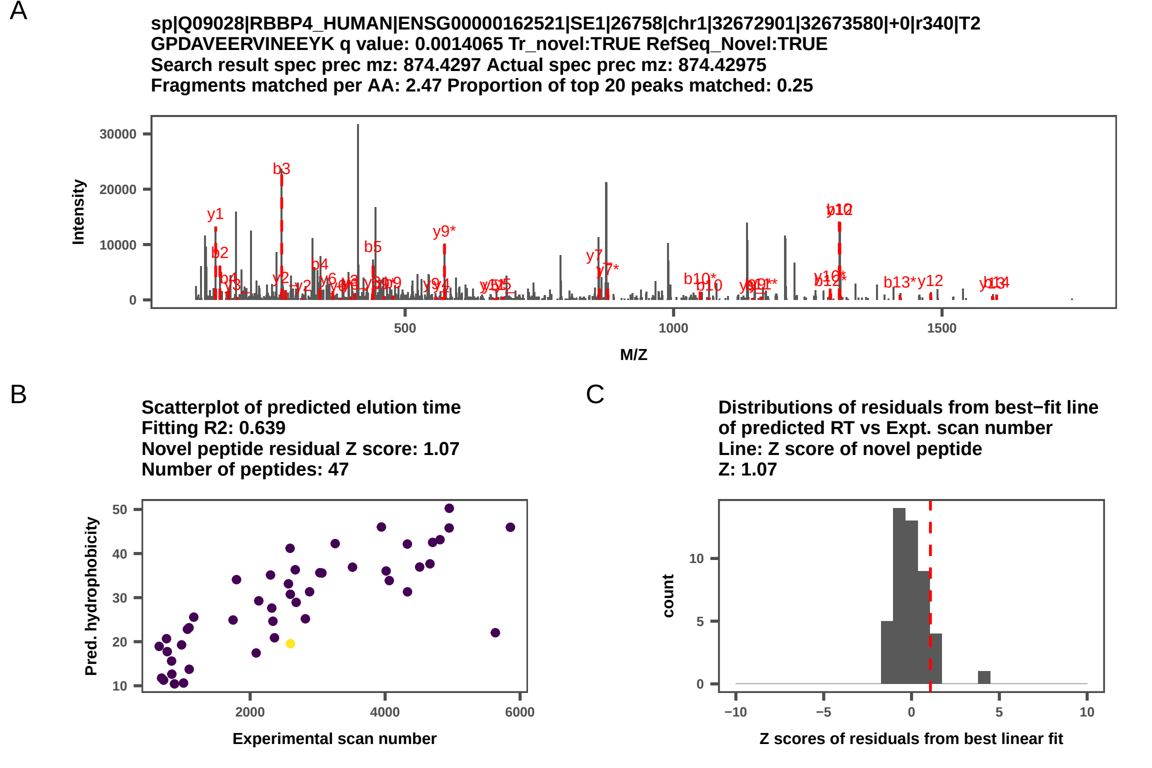
A
sp|Q09028|RBBP4_HUMAN|ENSG00000162521|SE1|26758|chr1|32672901|32673580|+0|r340|T2
GPDAVEERVINEEYK q value: 0.0014065 Tr_novel:TRUE RefSeq_Novel:TRUE
Search result spec prec mz: 874.4297 Actual spec prec mz: 874.42975
Fragments matched per AA: 2.47 Proportion of top 20 peaks matched: 0.25
Intensity
30000
20000
10000
0
500
1000
1500
M/Z
y1
b2
b3
b4
b5
y9*
y7
y7*
b10*
b10
b13*
y12
B
Scatterplot of predicted elution time
Fitting R2: 0.639
Novel peptide residual Z score: 1.07
Number of peptides: 47
Pred. hydrophobicity
50
40
30
20
10
2000
4000
6000
Experimental scan number
C
Distributions of residuals from best−fit line
of predicted RT vs Expt. scan number
Line: Z score of novel peptide
Z: 1.07
count
10
5
0
−10
−5
0
5
10
Z scores of residuals from best linear fit
