# Supplementary material for: Splice-Junction-Based Mapping of Alternative Isoforms in the Human Proteome
Source: Cell Rep. Author manuscript; Available in PMC 2020 Jan 15. (PMC6961840; doi:10.1016/j.celrep.2019.11.026)
Supplement: 2 [file NIHMS1546469-supplement-2.zip › DF1/PXD000561/AdrenalGland/AdrenalGland_2_BBS1_EGQSAPLLSAHVNMPGSEGLAAPNRPLNPE.pdf]

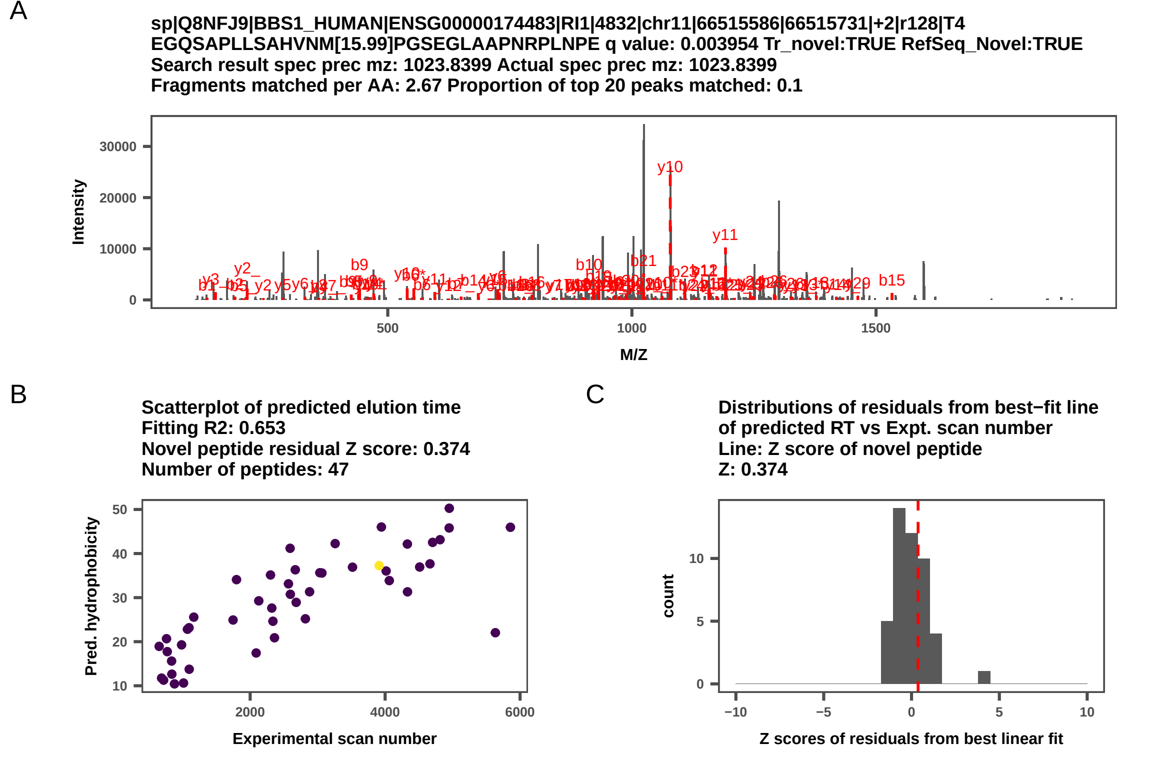
A
sp|Q8NFJ9|BBS1_HUMAN|ENSG00000174483|RI1|4832|chr11|66515586|66515731|+2|r128|T4
EGQSAPLLSAHVNM[15.99]PGSEGLAAPNRPLNPE q value: 0.003954 Tr_novel:TRUE RefSeq_Novel:TRUE
Search result spec prec mz: 1023.8399 Actual spec prec mz: 1023.8399
Fragments matched per AA: 2.67 Proportion of top 20 peaks matched: 0.1
Intensity
30000
20000
10000
0
y10
y11
b9
b10
b21
b15
500
1000
1500
M/Z
B
Scatterplot of predicted elution time
Fitting R2: 0.653
Novel peptide residual Z score: 0.374
Number of peptides: 47
Pred. hydrophobicity
50
40
30
20
10
2000
4000
6000
Experimental scan number
C
Distributions of residuals from best−fit line
of predicted RT vs Expt. scan number
Line: Z score of novel peptide
Z: 0.374
count
10
5
0
−10
−5
0
5
10
Z scores of residuals from best linear fit
