# Supplementary material for: Splice-Junction-Based Mapping of Alternative Isoforms in the Human Proteome
Source: Cell Rep. Author manuscript; Available in PMC 2020 Jan 15. (PMC6961840; doi:10.1016/j.celrep.2019.11.026)
Supplement: 2 [file NIHMS1546469-supplement-2.zip › DF1/PXD000561/AdrenalGland/AdrenalGland_5_EIF4A1_DIETFYNTSIEEMPLNVADLMSASQDSR.pdf]

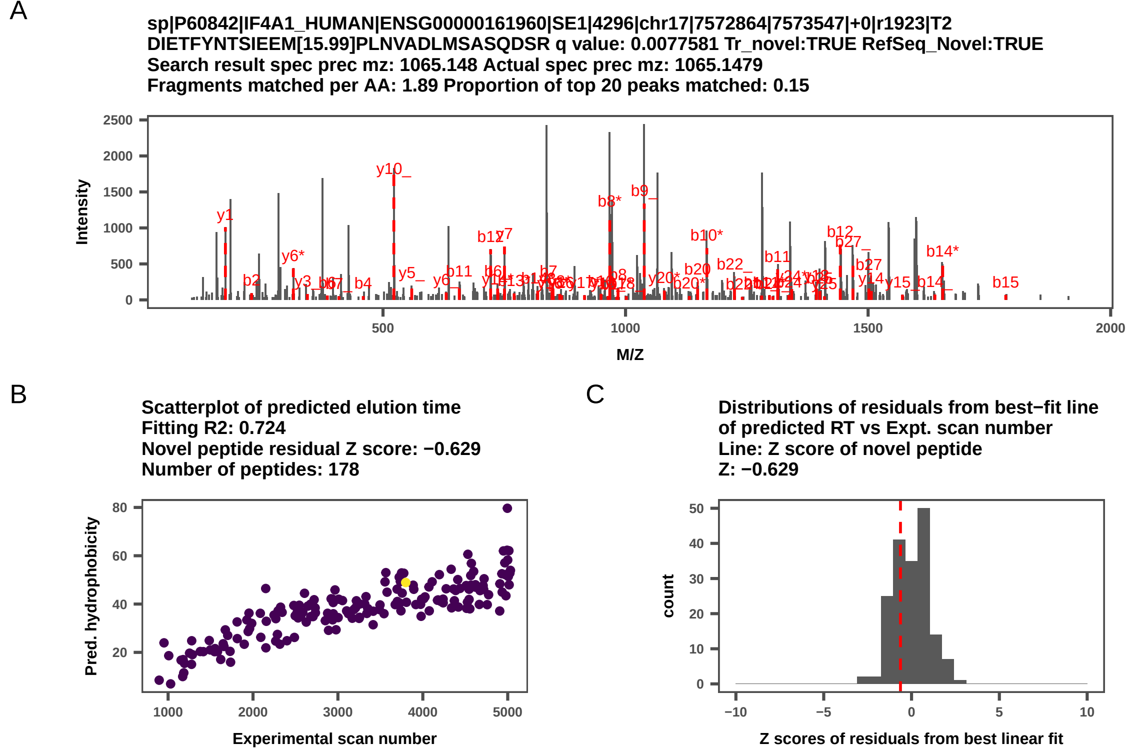
A
sp|P60842|IF4A1_HUMAN|ENSG00000161960|SE1|4296|chr17|7572864|7573547|+0|r1923|T2
DIETFYNTSIEEM[15.99]PLNVADLMSASQDSR q value: 0.0077581 Tr_novel:TRUE RefSeq_Novel:TRUE
Search result spec prec mz: 1065.148 Actual spec prec mz: 1065.1479
Fragments matched per AA: 1.89 Proportion of top 20 peaks matched: 0.15
Intensity
0
500
1000
1500
2000
2500
M/Z
500
1000
1500
2000
y1
b2
y6*
y3_
y10_
b4
y5_
b11
b8*
b9_
b10*
b20
b22_
b11
b12
b27_
b27
b14*
y14
y15_
b14_
b15
B
Scatterplot of predicted elution time
Fitting R2: 0.724
Novel peptide residual Z score: −0.629
Number of peptides: 178
Pred. hydrophobicity
20
40
60
80
Experimental scan number
1000
2000
3000
4000
5000
C
Distributions of residuals from best−fit line
of predicted RT vs Expt. scan number
Line: Z score of novel peptide
Z: −0.629
count
0
10
20
30
40
50
Z scores of residuals from best linear fit
−10
−5
0
5
10
