# Supplementary material for: Splice-Junction-Based Mapping of Alternative Isoforms in the Human Proteome
Source: Cell Rep. Author manuscript; Available in PMC 2020 Jan 15. (PMC6961840; doi:10.1016/j.celrep.2019.11.026)
Supplement: 2 [file NIHMS1546469-supplement-2.zip › DF1/PXD000561/AdrenalGland/AdrenalGland_4_ATXN2L_CLPWGVLSGPGAGMGGQK.pdf]

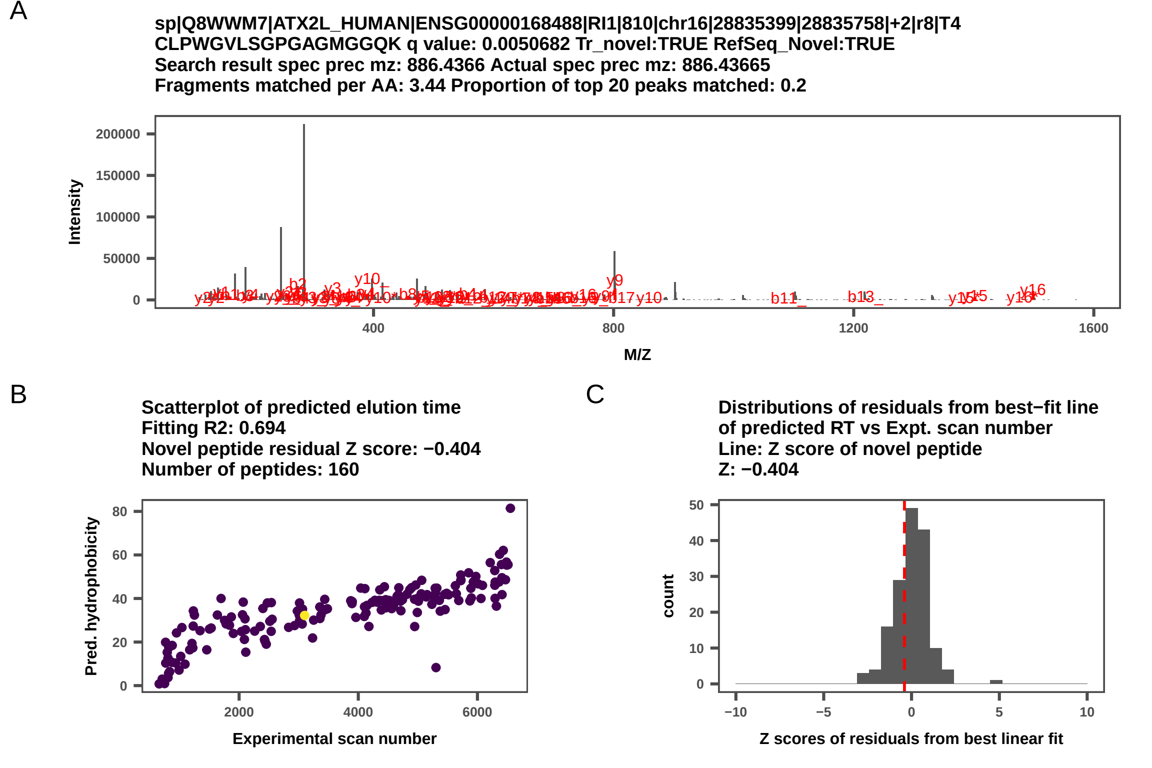
A
sp|Q8WWM7|ATX2L_HUMAN|ENSG00000168488|RI1|810|chr16|28835399|28835758|+2|r8|T4
CLPWGVLSGPGAGMGGQK q value: 0.0050682 Tr_novel:TRUE RefSeq_Novel:TRUE
Search result spec prec mz: 886.4366 Actual spec prec mz: 886.43665
Fragments matched per AA: 3.44 Proportion of top 20 peaks matched: 0.2
Intensity
M/Z
B
Scatterplot of predicted elution time
Fitting R2: 0.694
Novel peptide residual Z score: −0.404
Number of peptides: 160
Pred. hydrophobicity
Experimental scan number
C
Distributions of residuals from best−fit line
of predicted RT vs Expt. scan number
Line: Z score of novel peptide
Z: −0.404
count
Z scores of residuals from best linear fit
