# Supplementary material for: Splice-Junction-Based Mapping of Alternative Isoforms in the Human Proteome
Source: Cell Rep. Author manuscript; Available in PMC 2020 Jan 15. (PMC6961840; doi:10.1016/j.celrep.2019.11.026)
Supplement: 2 [file NIHMS1546469-supplement-2.zip › DF1/PXD000561/Heart/Heart_8_HDAC5_AVEITGAGPGDAPSAPSPPSGQLPQPV.pdf]

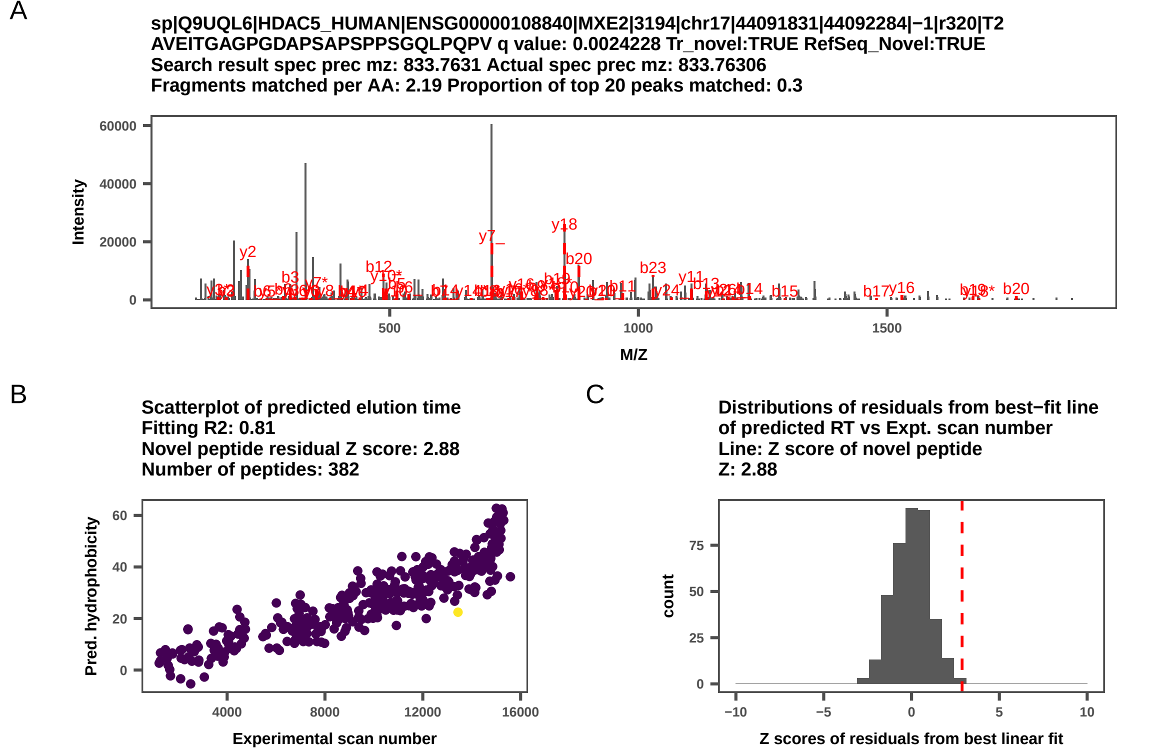
A
sp|Q9UQL6|HDAC5_HUMAN|ENSG00000108840|MXE2|3194|chr17|44091831|44092284|−1|r320|T2
AVEITGAGPGDAPSAPSPPSGQLPQPV q value: 0.0024228 Tr_novel:TRUE RefSeq_Novel:TRUE
Search result spec prec mz: 833.7631 Actual spec prec mz: 833.76306
Fragments matched per AA: 2.19 Proportion of top 20 peaks matched: 0.3
Intensity
60000
40000
20000
0
y2
y7_
y18
b20
b23
b12
y10*
b3
y11
b13
b15
b17
y16
b20
500
1000
1500
M/Z
B
Scatterplot of predicted elution time
Fitting R2: 0.81
Novel peptide residual Z score: 2.88
Number of peptides: 382
Pred. hydrophobicity
60
40
20
0
4000
8000
12000
16000
Experimental scan number
C
Distributions of residuals from best−fit line
of predicted RT vs Expt. scan number
Line: Z score of novel peptide
Z: 2.88
count
75
50
25
0
−10
−5
0
5
10
Z scores of residuals from best linear fit
