# Supplementary material for: Splice-Junction-Based Mapping of Alternative Isoforms in the Human Proteome
Source: Cell Rep. Author manuscript; Available in PMC 2020 Jan 15. (PMC6961840; doi:10.1016/j.celrep.2019.11.026)
Supplement: 2 [file NIHMS1546469-supplement-2.zip › DF1/PXD000561/Heart/Heart_15_TNS1_TPLSALGLKPHNPADILLHPTGEEDEGK.pdf]

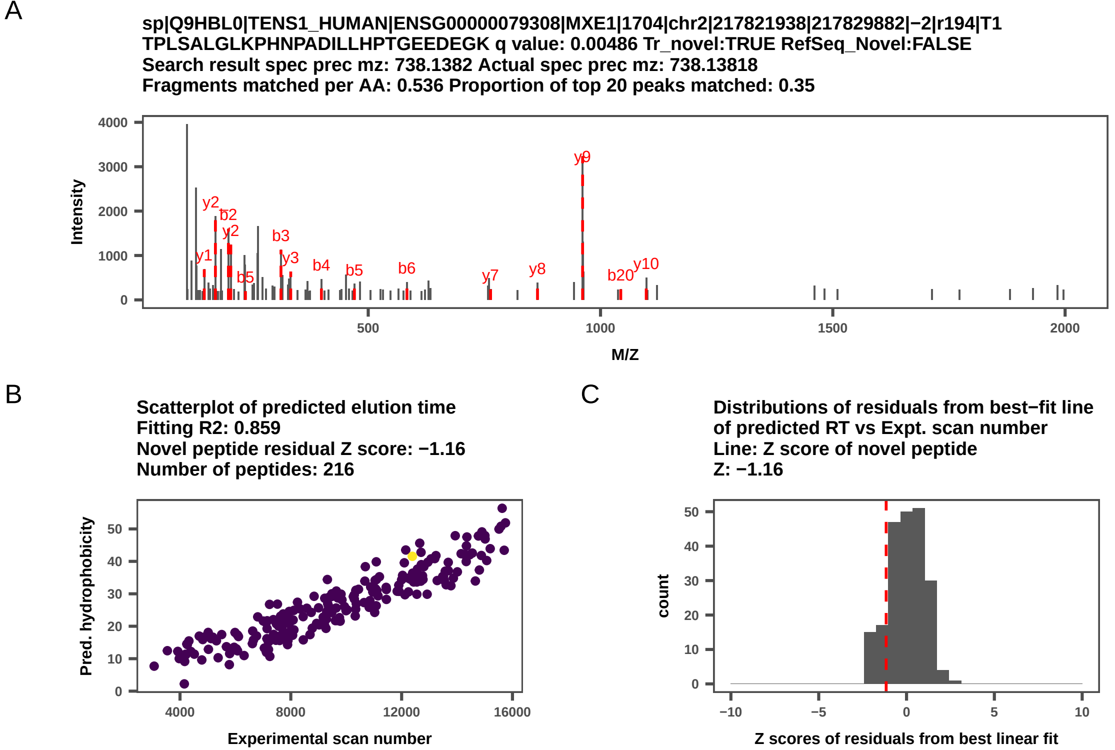
A
sp|Q9HBL0|TENS1_HUMAN|ENSG00000079308|MXE1|1704|chr2|217821938|217829882|−2|r194|T1
TPLSALGLKPHNPADILLHPTGEEDEGK q value: 0.00486 Tr_novel:TRUE RefSeq_Novel:FALSE
Search result spec prec mz: 738.1382 Actual spec prec mz: 738.13818
Fragments matched per AA: 0.536 Proportion of top 20 peaks matched: 0.35
Intensity
0
1000
2000
3000
4000
y1
y2
b2
y2
b5
b3
y3
b4
b5
b6
y7
y8
y9
b20
y10
500
1000
1500
2000
M/Z
B
Scatterplot of predicted elution time
Fitting R2: 0.859
Novel peptide residual Z score: −1.16
Number of peptides: 216
Pred. hydrophobicity
0
10
20
30
40
50
4000
8000
12000
16000
Experimental scan number
C
Distributions of residuals from best−fit line
of predicted RT vs Expt. scan number
Line: Z score of novel peptide
Z: −1.16
count
0
10
20
30
40
50
−10
−5
0
5
10
Z scores of residuals from best linear fit
