# Supplementary material for: Splice-Junction-Based Mapping of Alternative Isoforms in the Human Proteome
Source: Cell Rep. Author manuscript; Available in PMC 2020 Jan 15. (PMC6961840; doi:10.1016/j.celrep.2019.11.026)
Supplement: 2 [file NIHMS1546469-supplement-2.zip › DF1/PXD000561/Heart/Heart_16_SETDB1_KLDAATATVESEEIAELQQAVV.pdf]

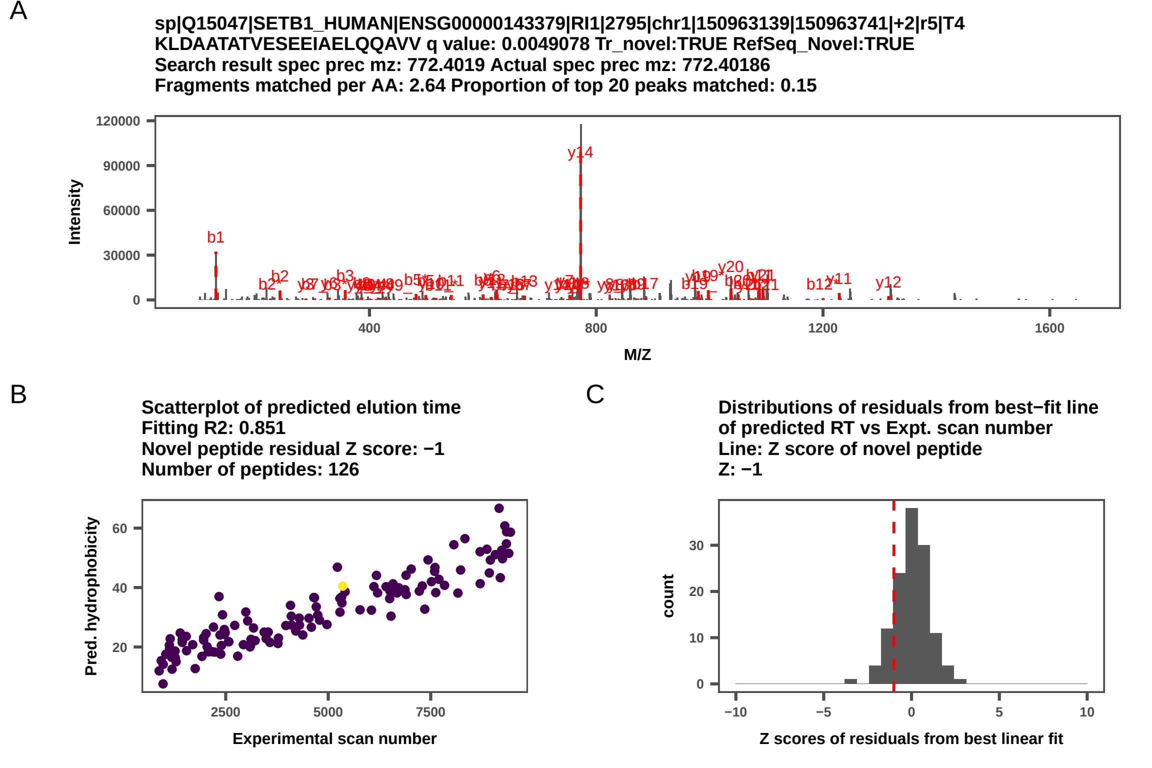
A
sp|Q15047|SETB1_HUMAN|ENSG00000143379|RI1|2795|chr1|150963139|150963741|+2|r5|T4
KLDAATATVESEEIAELQQAVV q value: 0.0049078 Tr_novel:TRUE RefSeq_Novel:TRUE
Search result spec prec mz: 772.4019 Actual spec prec mz: 772.40186
Fragments matched per AA: 2.64 Proportion of top 20 peaks matched: 0.15
Intensity
120000
90000
60000
30000
0
y14
b1
b2
b2*
b3
y20
y11
b12*
y12
400
800
1200
1600
M/Z
B
Scatterplot of predicted elution time
Fitting R2: 0.851
Novel peptide residual Z score: −1
Number of peptides: 126
Pred. hydrophobicity
60
40
20
2500
5000
7500
Experimental scan number
C
Distributions of residuals from best−fit line
of predicted RT vs Expt. scan number
Line: Z score of novel peptide
Z: −1
count
30
20
10
0
−10
−5
0
5
10
Z scores of residuals from best linear fit
