# Supplementary material for: Splice-Junction-Based Mapping of Alternative Isoforms in the Human Proteome
Source: Cell Rep. Author manuscript; Available in PMC 2020 Jan 15. (PMC6961840; doi:10.1016/j.celrep.2019.11.026)
Supplement: 2 [file NIHMS1546469-supplement-2.zip › DF1/PXD000561/Heart/Heart_9_TPM3_AISEELDHALNDMTSGSQMLKLDK.pdf]

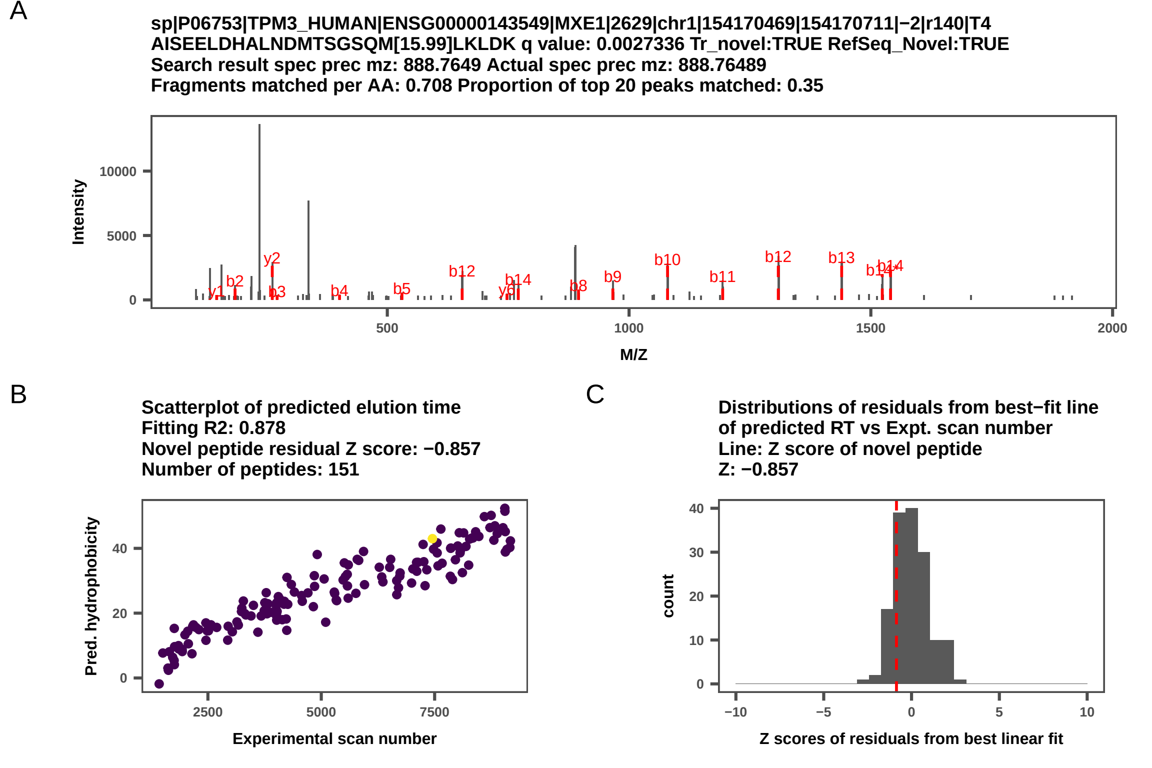
A
sp|P06753|TPM3_HUMAN|ENSG00000143549|MXE1|2629|chr1|154170469|154170711|−2|r140|T4
AISEELDHALNDMTSGSQM[15.99]LKLDK q value: 0.0027336 Tr_novel:TRUE RefSeq_Novel:TRUE
Search result spec prec mz: 888.7649 Actual spec prec mz: 888.76489
Fragments matched per AA: 0.708 Proportion of top 20 peaks matched: 0.35
Intensity
10000
5000
0
y1
b2
y2
b3
b4
b5
b12
y6
b14
b8
b9
b10
b11
b12
b13
b14
b14
500
1000
1500
2000
M/Z
B
Scatterplot of predicted elution time
Fitting R2: 0.878
Novel peptide residual Z score: −0.857
Number of peptides: 151
Pred. hydrophobicity
40
20
0
2500
5000
7500
Experimental scan number
C
Distributions of residuals from best−fit line
of predicted RT vs Expt. scan number
Line: Z score of novel peptide
Z: −0.857
count
40
30
20
10
0
−10
−5
0
5
10
Z scores of residuals from best linear fit
