# Supplementary material for: Splice-Junction-Based Mapping of Alternative Isoforms in the Human Proteome
Source: Cell Rep. Author manuscript; Available in PMC 2020 Jan 15. (PMC6961840; doi:10.1016/j.celrep.2019.11.026)
Supplement: 2 [file NIHMS1546469-supplement-2.zip › DF1/PXD000561/Heart/Heart_4_ART3_IPEPFPLPAPGPVPVPGPK.pdf]

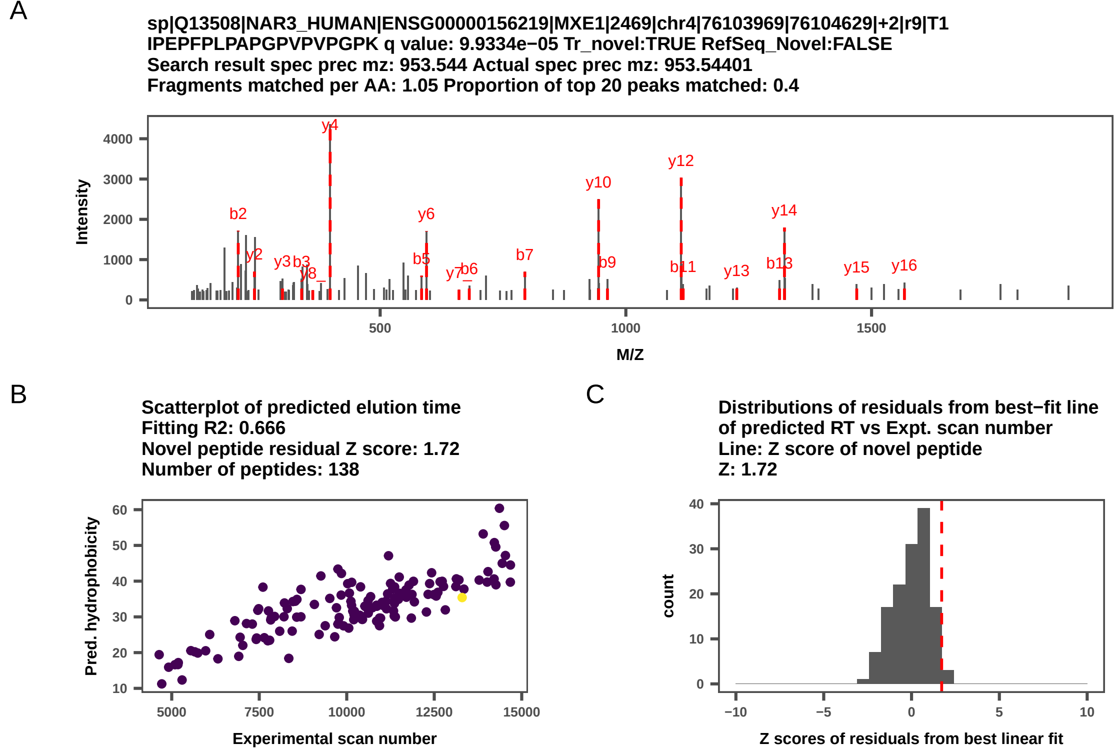
A
sp|Q13508|NAR3_HUMAN|ENSG00000156219|MXE1|2469|chr4|76103969|76104629|+2|r9|T1
IPEPFPLPAPGPVPVPGPK q value: 9.9334e−05 Tr_novel:TRUE RefSeq_Novel:FALSE
Search result spec prec mz: 953.544 Actual spec prec mz: 953.54401
Fragments matched per AA: 1.05 Proportion of top 20 peaks matched: 0.4
Intensity
M/Z
y4
y12
y10
y14
b2
y6
y2
y3
b3
y8_
b5
y7_b6
b7
b9
b11
y13
b13
y15
y16
B
Scatterplot of predicted elution time
Fitting R2: 0.666
Novel peptide residual Z score: 1.72
Number of peptides: 138
Pred. hydrophobicity
Experimental scan number
C
Distributions of residuals from best−fit line
of predicted RT vs Expt. scan number
Line: Z score of novel peptide
Z: 1.72
count
Z scores of residuals from best linear fit
