# Supplementary material for: Splice-Junction-Based Mapping of Alternative Isoforms in the Human Proteome
Source: Cell Rep. Author manuscript; Available in PMC 2020 Jan 15. (PMC6961840; doi:10.1016/j.celrep.2019.11.026)
Supplement: 2 [file NIHMS1546469-supplement-2.zip › DF1/PXD000561/Heart/Heart_22_WWP2_LLYAIEETEGFGQK.pdf]

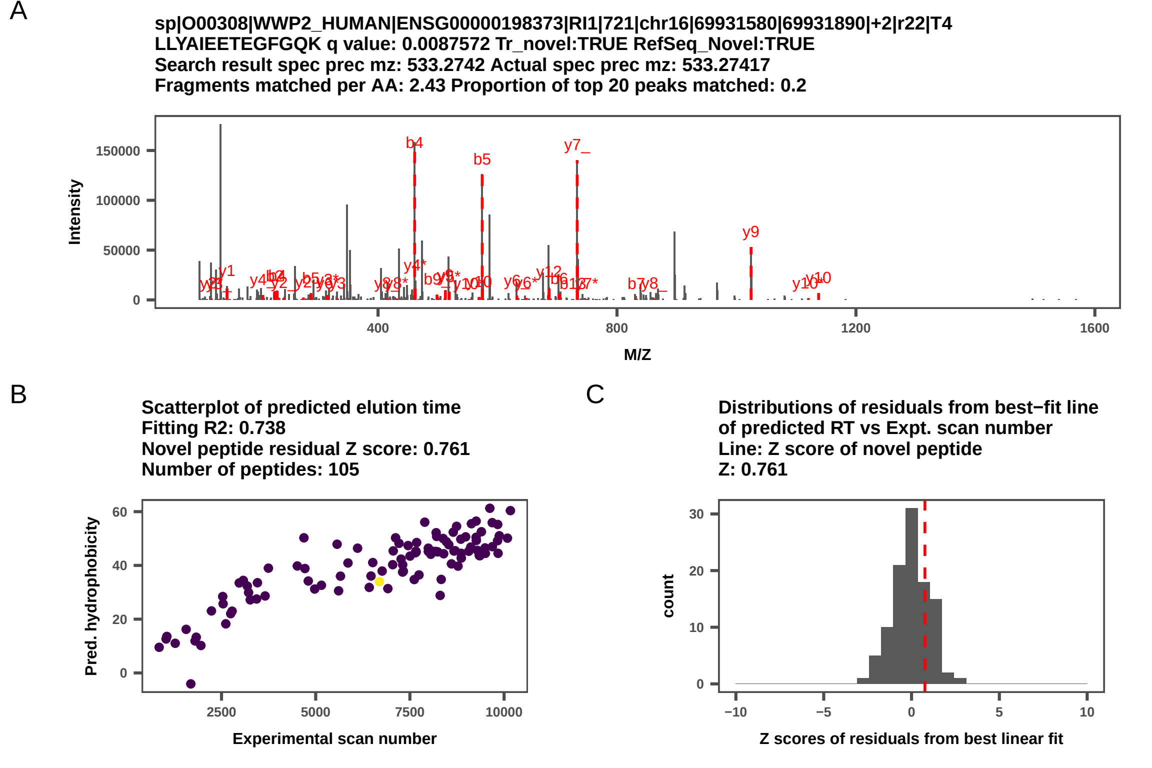
A
sp|O00308|WWP2_HUMAN|ENSG00000198373|RI1|721|chr16|69931580|69931890|+2|r22|T4
LLYAIEETEGFGQK q value: 0.0087572 Tr_novel:TRUE RefSeq_Novel:TRUE
Search result spec prec mz: 533.2742 Actual spec prec mz: 533.27417
Fragments matched per AA: 2.43 Proportion of top 20 peaks matched: 0.2
Intensity
150000
100000
50000
0
b4
b5
y7_
y9
y1
y4*
M/Z
400
800
1200
1600
B
Scatterplot of predicted elution time
Fitting R2: 0.738
Novel peptide residual Z score: 0.761
Number of peptides: 105
Pred. hydrophobicity
60
40
20
0
2500
5000
7500
10000
Experimental scan number
C
Distributions of residuals from best−fit line
of predicted RT vs Expt. scan number
Line: Z score of novel peptide
Z: 0.761
count
30
20
10
0
−10
−5
0
5
10
Z scores of residuals from best linear fit
