# Supplementary material for: Splice-Junction-Based Mapping of Alternative Isoforms in the Human Proteome
Source: Cell Rep. Author manuscript; Available in PMC 2020 Jan 15. (PMC6961840; doi:10.1016/j.celrep.2019.11.026)
Supplement: 2 [file NIHMS1546469-supplement-2.zip › DF1/PXD000561/Heart/Heart_23_MTM1_KLSDPPTSPSSPSQMMPHVQTHVSR.pdf]

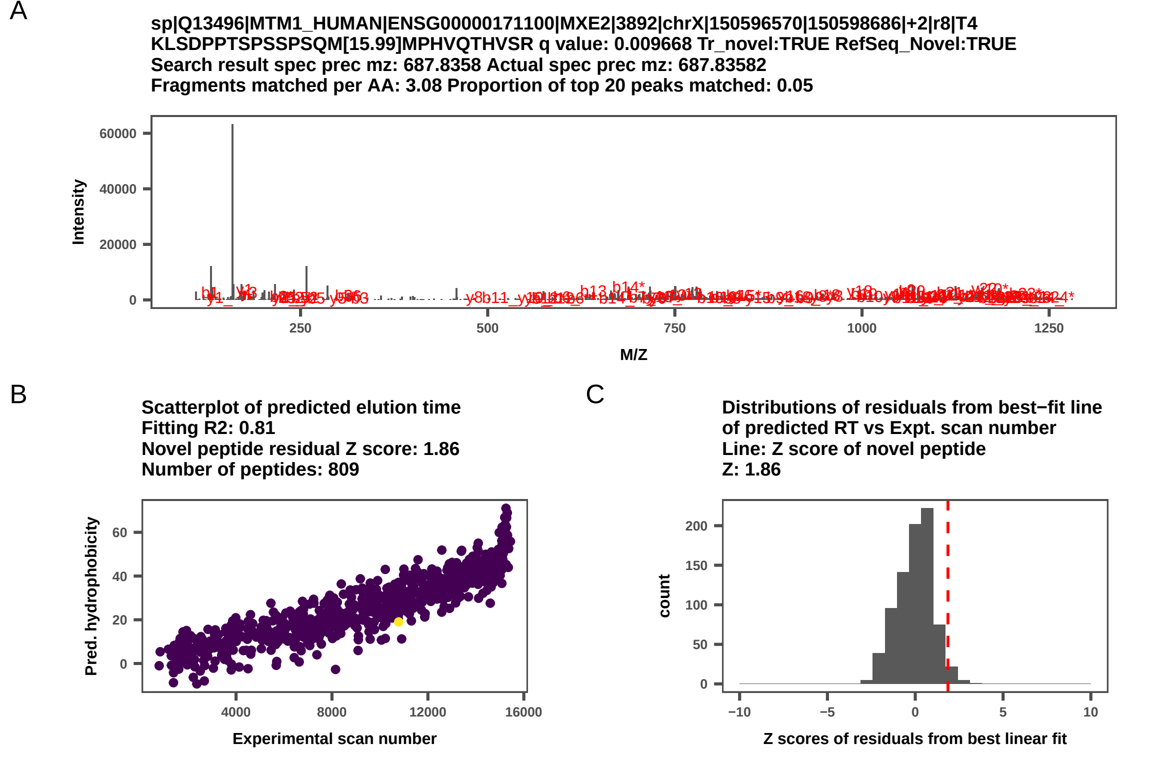
A
sp|Q13496|MTM1_HUMAN|ENSG00000171100|MXE2|3892|chrX|150596570|150598686|+2|r8|T4
KLSDPPTSPSSPSQM[15.99]MPHVQTHVSR q value: 0.009668 Tr_novel:TRUE RefSeq_Novel:TRUE
Search result spec prec mz: 687.8358 Actual spec prec mz: 687.83582
Fragments matched per AA: 3.08 Proportion of top 20 peaks matched: 0.05
Intensity
60000
40000
20000
0
250
500
750
1000
1250
M/Z
B
Scatterplot of predicted elution time
Fitting R2: 0.81
Novel peptide residual Z score: 1.86
Number of peptides: 809
Pred. hydrophobicity
60
40
20
0
4000
8000
12000
16000
Experimental scan number
C
Distributions of residuals from best−fit line
of predicted RT vs Expt. scan number
Line: Z score of novel peptide
Z: 1.86
count
200
150
100
50
0
−10
−5
0
5
10
Z scores of residuals from best linear fit
