# Supplementary material for: Splice-Junction-Based Mapping of Alternative Isoforms in the Human Proteome
Source: Cell Rep. Author manuscript; Available in PMC 2020 Jan 15. (PMC6961840; doi:10.1016/j.celrep.2019.11.026)
Supplement: 2 [file NIHMS1546469-supplement-2.zip › DF1/PXD000561/Heart/Heart_19_LRCH4_VGGAAAVSTQAMHNLLKPGLR.pdf]

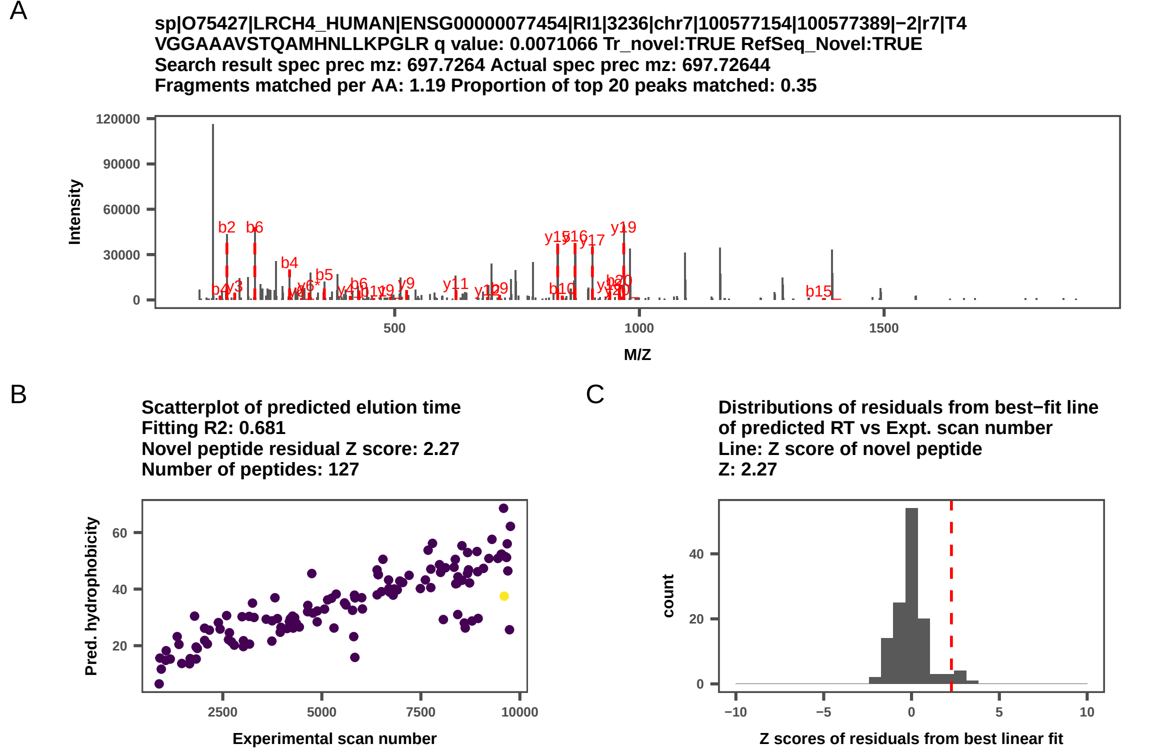
A
sp|O75427|LRCH4_HUMAN|ENSG00000077454|RI1|3236|chr7|100577154|100577389|−2|r7|T4
VGGAAAVSTQAMHNLLKPGLR q value: 0.0071066 Tr_novel:TRUE RefSeq_Novel:TRUE
Search result spec prec mz: 697.7264 Actual spec prec mz: 697.72644
Fragments matched per AA: 1.19 Proportion of top 20 peaks matched: 0.35
Intensity
120000
90000
60000
30000
0
b2
b6
b4
b5
y3
y9
y11
y15
y16
y17
y19
b10
b15
500
1000
1500
M/Z
B
Scatterplot of predicted elution time
Fitting R2: 0.681
Novel peptide residual Z score: 2.27
Number of peptides: 127
Pred. hydrophobicity
60
40
20
2500
5000
7500
10000
Experimental scan number
C
Distributions of residuals from best−fit line
of predicted RT vs Expt. scan number
Line: Z score of novel peptide
Z: 2.27
count
40
20
0
−10
−5
0
5
10
Z scores of residuals from best linear fit
