# Supplementary material for: Splice-Junction-Based Mapping of Alternative Isoforms in the Human Proteome
Source: Cell Rep. Author manuscript; Available in PMC 2020 Jan 15. (PMC6961840; doi:10.1016/j.celrep.2019.11.026)
Supplement: 2 [file NIHMS1546469-supplement-2.zip › DF1/PXD000561/Heart/Heart_17_MINK1_ATAAGAGAAEAAGEGAAAAAGGHAGSAAGGGAAAGGAR.pdf]

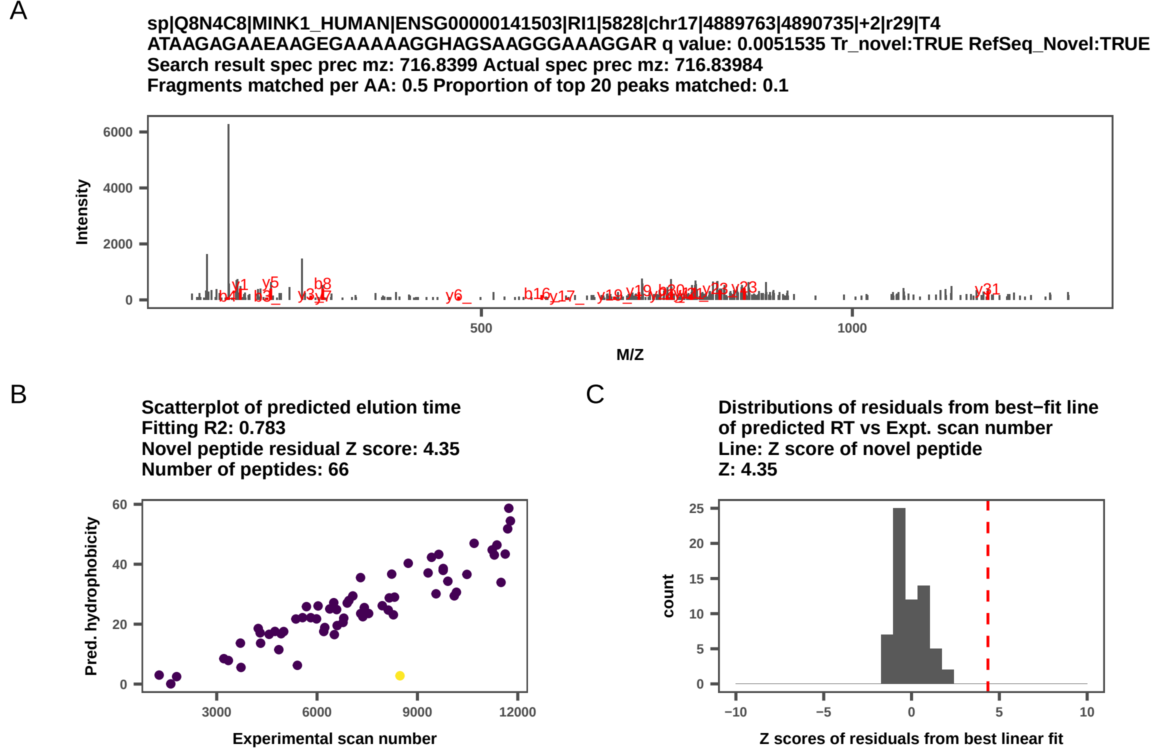
A
sp|Q8N4C8|MINK1_HUMAN|ENSG00000141503|RI1|5828|chr17|4889763|4890735|+2|r29|T4
ATAAGAGAAEAAGEGAAAAAGGHAGSAAGGGAAAGGAR q value: 0.0051535 Tr_novel:TRUE RefSeq_Novel:TRUE
Search result spec prec mz: 716.8399 Actual spec prec mz: 716.83984
Fragments matched per AA: 0.5 Proportion of top 20 peaks matched: 0.1
Intensity
6000
4000
2000
0
500
1000
M/Z
B
Scatterplot of predicted elution time
Fitting R2: 0.783
Novel peptide residual Z score: 4.35
Number of peptides: 66
Pred. hydrophobicity
60
40
20
0
3000
6000
9000
12000
Experimental scan number
C
Distributions of residuals from best−fit line
of predicted RT vs Expt. scan number
Line: Z score of novel peptide
Z: 4.35
count
25
20
15
10
5
0
−10
−5
0
5
10
Z scores of residuals from best linear fit
