# Supplementary material for: Splice-Junction-Based Mapping of Alternative Isoforms in the Human Proteome
Source: Cell Rep. Author manuscript; Available in PMC 2020 Jan 15. (PMC6961840; doi:10.1016/j.celrep.2019.11.026)
Supplement: 2 [file NIHMS1546469-supplement-2.zip › DF1/PXD000561/Heart/Heart_7_GOT2_LAHAIHQVTR.pdf]

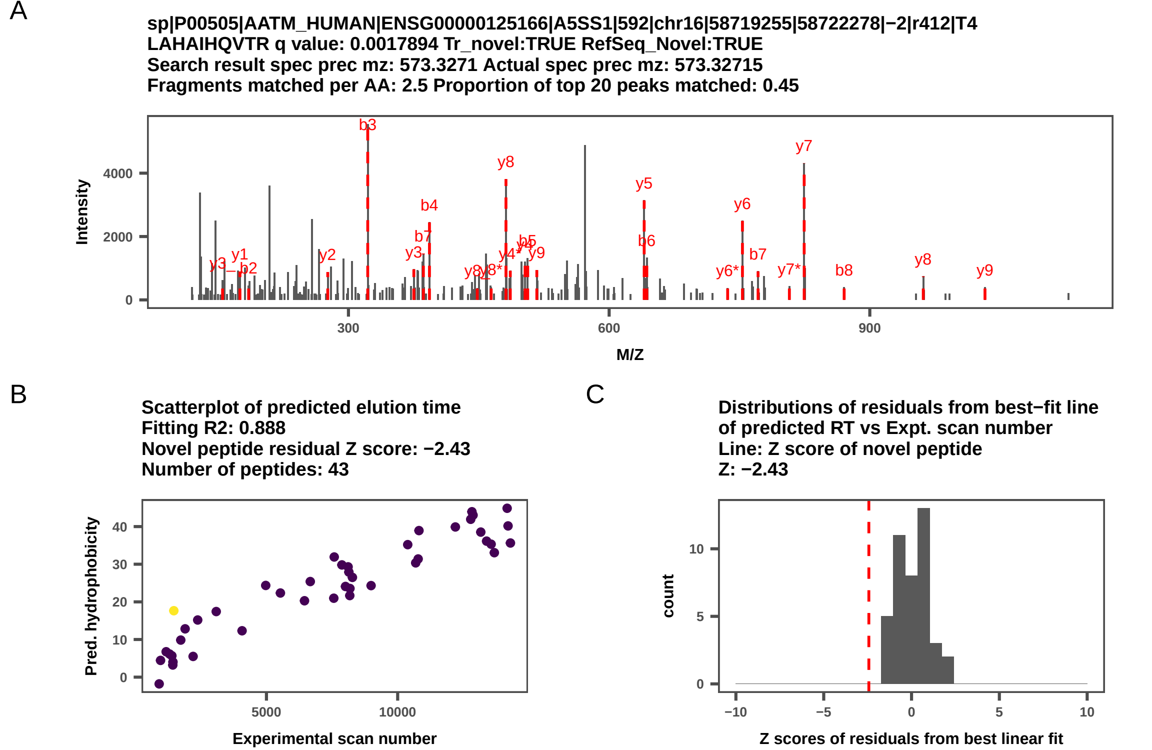
A
sp|P00505|AATM_HUMAN|ENSG00000125166|A5SS1|592|chr16|58719255|58722278|−2|r412|T4
LAHAIHQVTR q value: 0.0017894 Tr_novel:TRUE RefSeq_Novel:TRUE
Search result spec prec mz: 573.3271 Actual spec prec mz: 573.32715
Fragments matched per AA: 2.5 Proportion of top 20 peaks matched: 0.45
Intensity
4000
2000
0
M/Z
300
600
900
b3
y7
y8
y5
b4
y6
b7
b6
y3
y1
b2
y2
b5
y9
y4*
y8*
b8
y6*
y7*
B
Scatterplot of predicted elution time
Fitting R2: 0.888
Novel peptide residual Z score: −2.43
Number of peptides: 43
Pred. hydrophobicity
40
30
20
10
0
5000
10000
Experimental scan number
C
Distributions of residuals from best−fit line
of predicted RT vs Expt. scan number
Line: Z score of novel peptide
Z: −2.43
count
10
5
0
−10
−5
0
5
10
Z scores of residuals from best linear fit
