# Supplementary material for: Splice-Junction-Based Mapping of Alternative Isoforms in the Human Proteome
Source: Cell Rep. Author manuscript; Available in PMC 2020 Jan 15. (PMC6961840; doi:10.1016/j.celrep.2019.11.026)
Supplement: 2 [file NIHMS1546469-supplement-2.zip › DF1/PXD000561/Heart/Heart_3_TTN_SNCTVSVHVSEPPR.pdf]

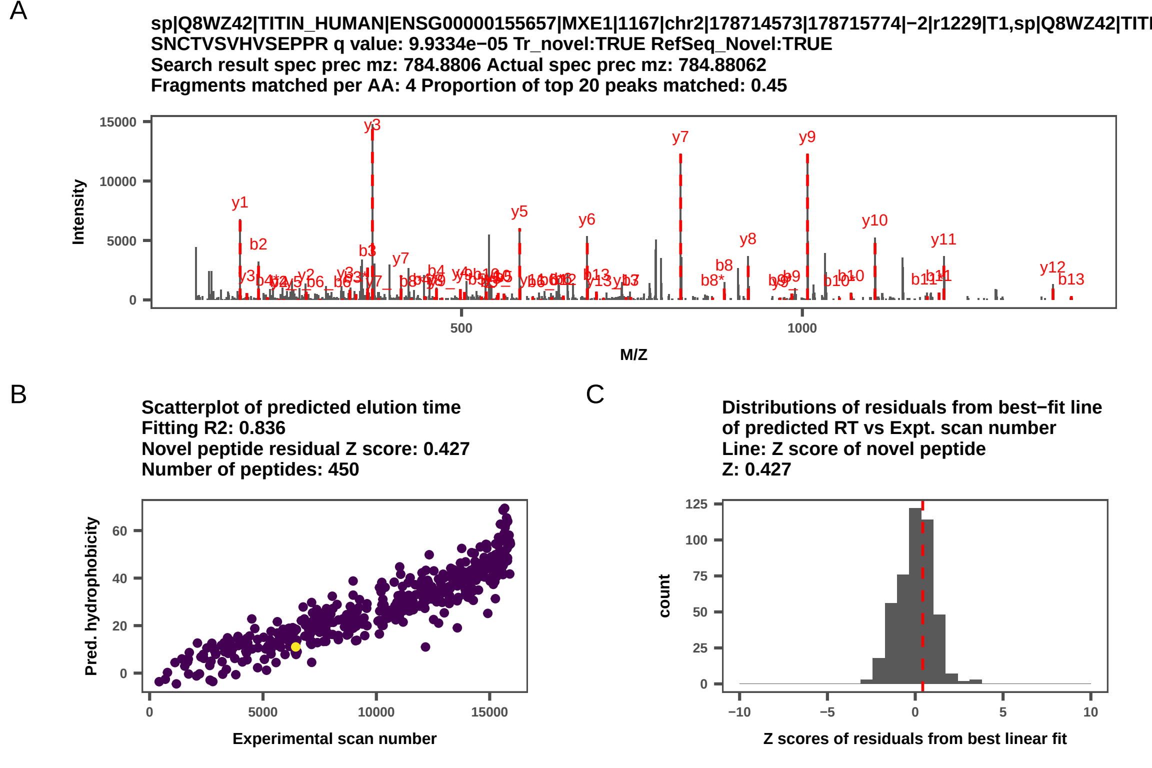
A
sp|Q8WZ42|TITIN_HUMAN|ENSG00000155657|MXE1|1167|chr2|178714573|178715774|−2|r1229|T1,sp|Q8WZ42|TITI
SNCTVSVHVSEPPR q value: 9.9334e−05 Tr_novel:TRUE RefSeq_Novel:TRUE
Search result spec prec mz: 784.8806 Actual spec prec mz: 784.88062
Fragments matched per AA: 4 Proportion of top 20 peaks matched: 0.45
Intensity
15000
10000
5000
0
y1
b2
y3
b3
y7
b4
y5
y6
y7
b8
y8
b8*
y9
y10
b10
y11
b11
y12
b13
500
1000
M/Z
B
Scatterplot of predicted elution time
Fitting R2: 0.836
Novel peptide residual Z score: 0.427
Number of peptides: 450
Pred. hydrophobicity
60
40
20
0
0
5000
10000
15000
Experimental scan number
C
Distributions of residuals from best−fit line
of predicted RT vs Expt. scan number
Line: Z score of novel peptide
Z: 0.427
count
125
100
75
50
25
0
−10
−5
0
5
10
Z scores of residuals from best linear fit
