# Supplementary material for: Splice-Junction-Based Mapping of Alternative Isoforms in the Human Proteome
Source: Cell Rep. Author manuscript; Available in PMC 2020 Jan 15. (PMC6961840; doi:10.1016/j.celrep.2019.11.026)
Supplement: 2 [file NIHMS1546469-supplement-2.zip › DF1/PXD000561/Heart/Heart_2_TTN_APHVEFLRPLTDLQVR.pdf]

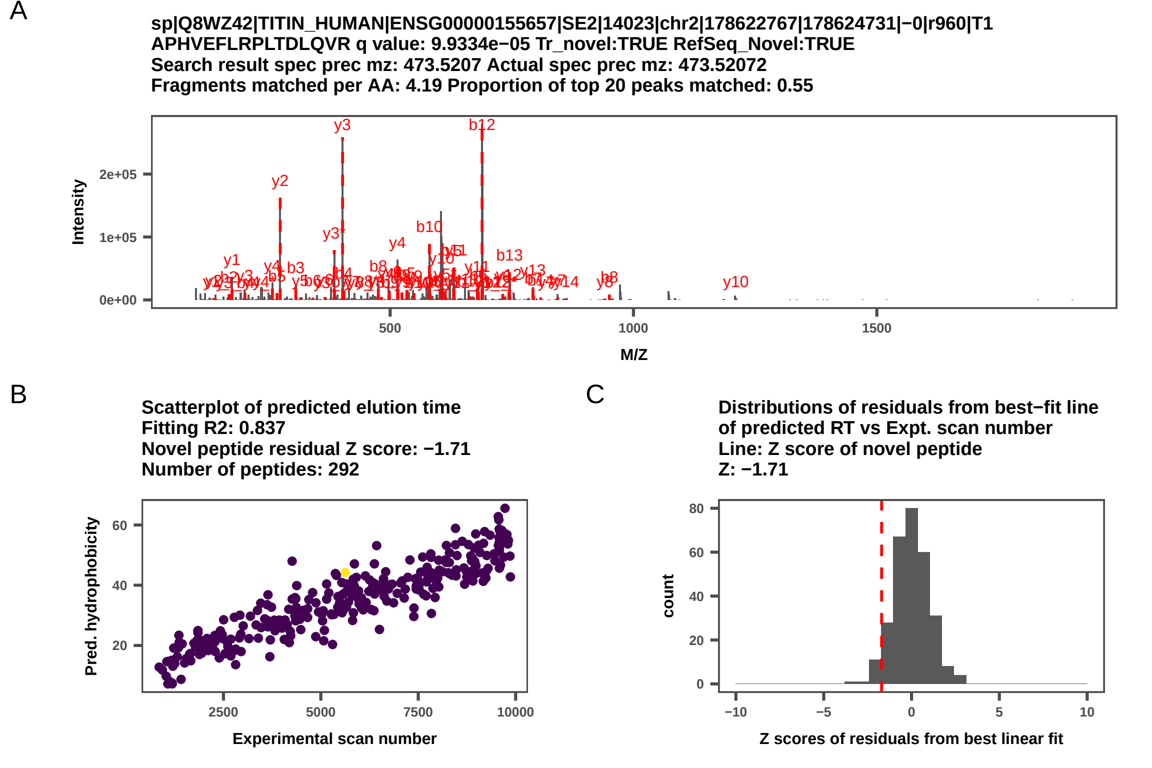
A
sp|Q8WZ42|TITIN_HUMAN|ENSG00000155657|SE2|14023|chr2|178622767|178624731|−0|r960|T1
APHVEFLRPLTDLQVR q value: 9.9334e−05 Tr_novel:TRUE RefSeq_Novel:TRUE
Search result spec prec mz: 473.5207 Actual spec prec mz: 473.52072
Fragments matched per AA: 4.19 Proportion of top 20 peaks matched: 0.55
Intensity
2e+05
1e+05
0e+00
y3
b12
y2
b10
y4
y1
b3
b13
y10
M/Z
500
1000
1500
B
Scatterplot of predicted elution time
Fitting R2: 0.837
Novel peptide residual Z score: −1.71
Number of peptides: 292
Pred. hydrophobicity
60
40
20
2500
5000
7500
10000
Experimental scan number
C
Distributions of residuals from best−fit line
of predicted RT vs Expt. scan number
Line: Z score of novel peptide
Z: −1.71
count
80
60
40
20
0
−10
−5
0
5
10
Z scores of residuals from best linear fit
