# Supplementary material for: Splice-Junction-Based Mapping of Alternative Isoforms in the Human Proteome
Source: Cell Rep. Author manuscript; Available in PMC 2020 Jan 15. (PMC6961840; doi:10.1016/j.celrep.2019.11.026)
Supplement: 2 [file NIHMS1546469-supplement-2.zip › DF1/PXD000561/Heart/Heart_24_CARM1_AAPTTSAAGWPWQVSR.pdf]

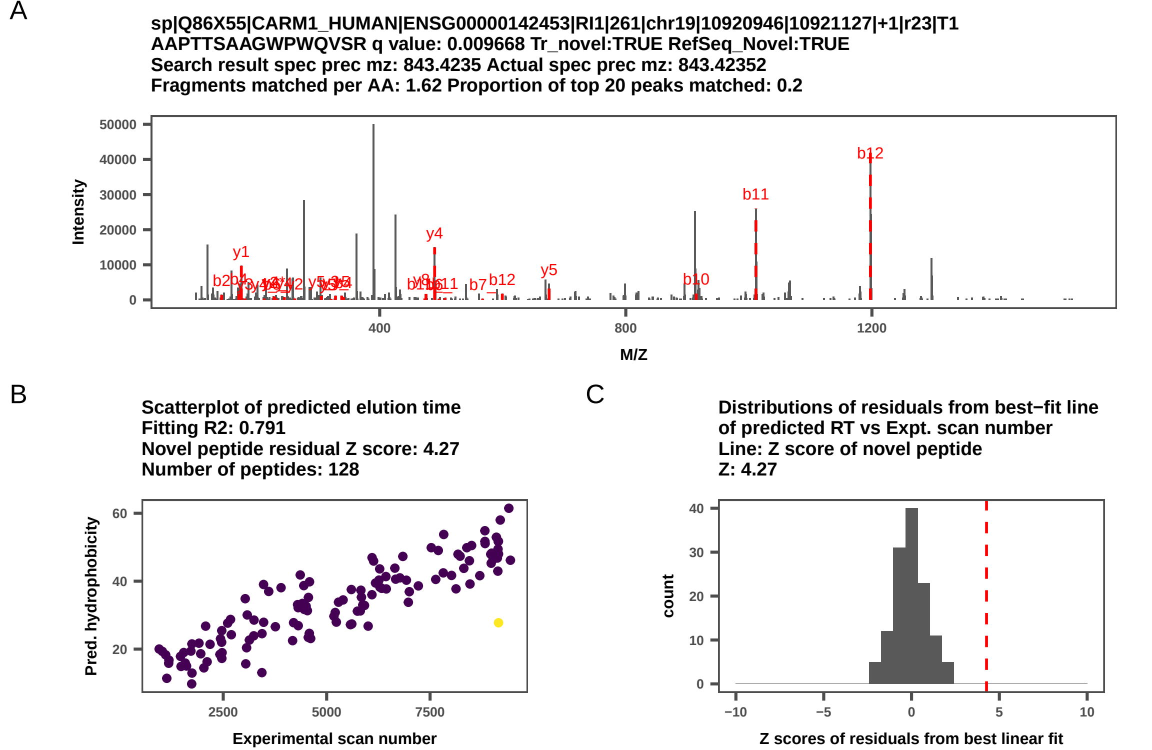
A
sp|Q86X55|CARM1_HUMAN|ENSG00000142453|RI1|261|chr19|10920946|10921127|+1|r23|T1
AAPTTSAAGWPWQVSR q value: 0.009668 Tr_novel:TRUE RefSeq_Novel:TRUE
Search result spec prec mz: 843.4235 Actual spec prec mz: 843.42352
Fragments matched per AA: 1.62 Proportion of top 20 peaks matched: 0.2
Intensity
50000
40000
30000
20000
10000
0
M/Z
400
800
1200
y1
y4
y5
b10
b11
b12
b7
B
Scatterplot of predicted elution time
Fitting R2: 0.791
Novel peptide residual Z score: 4.27
Number of peptides: 128
Pred. hydrophobicity
60
40
20
2500
5000
7500
Experimental scan number
C
Distributions of residuals from best−fit line
of predicted RT vs Expt. scan number
Line: Z score of novel peptide
Z: 4.27
count
40
30
20
10
0
−10
−5
0
5
10
Z scores of residuals from best linear fit
