# Supplementary material for: Splice-Junction-Based Mapping of Alternative Isoforms in the Human Proteome
Source: Cell Rep. Author manuscript; Available in PMC 2020 Jan 15. (PMC6961840; doi:10.1016/j.celrep.2019.11.026)
Supplement: 2 [file NIHMS1546469-supplement-2.zip › DF1/PXD000561/Heart/Heart_14_NCL_FNLVMWILNLLK.pdf]

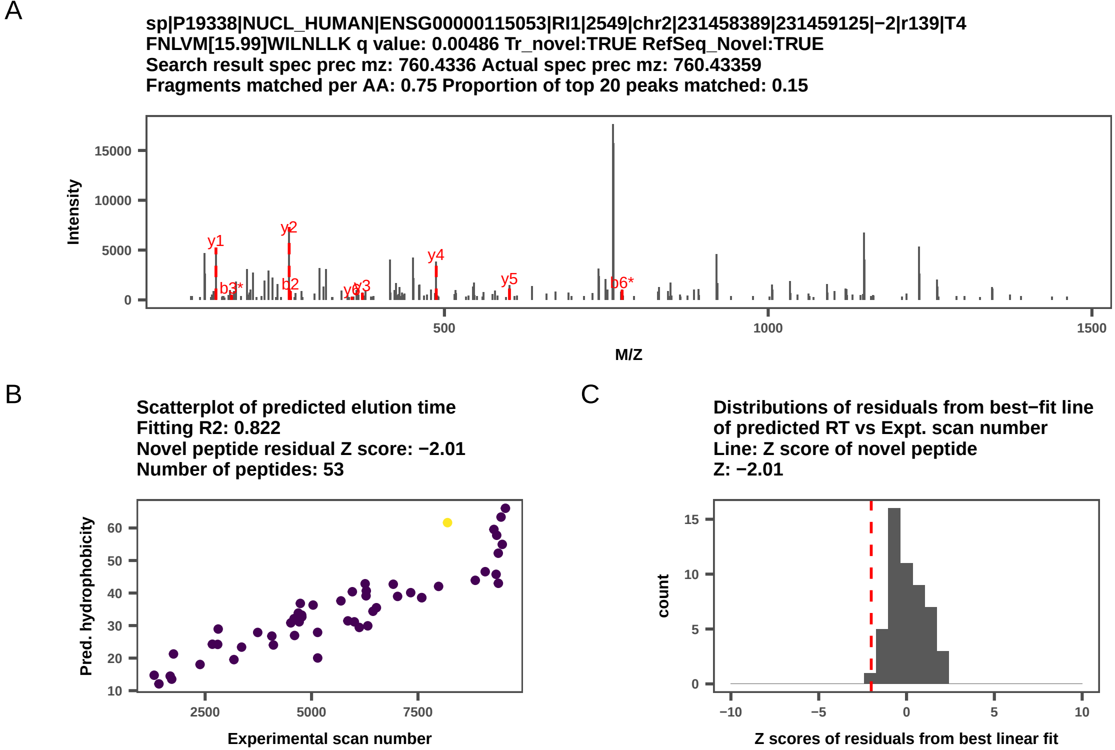
A
sp|P19338|NUCL_HUMAN|ENSG00000115053|RI1|2549|chr2|231458389|231459125|−2|r139|T4
FNLVM[15.99]WILNLLK q value: 0.00486 Tr_novel:TRUE RefSeq_Novel:TRUE
Search result spec prec mz: 760.4336 Actual spec prec mz: 760.43359
Fragments matched per AA: 0.75 Proportion of top 20 peaks matched: 0.15
Intensity
15000
10000
5000
0
y1
y2
b3*
b2
y4
y6
y3
y5
b6*
500
1000
1500
M/Z
B
Scatterplot of predicted elution time
Fitting R2: 0.822
Novel peptide residual Z score: −2.01
Number of peptides: 53
Pred. hydrophobicity
60
50
40
30
20
10
2500
5000
7500
Experimental scan number
C
Distributions of residuals from best−fit line
of predicted RT vs Expt. scan number
Line: Z score of novel peptide
Z: −2.01
count
15
10
5
0
−10
−5
0
5
10
Z scores of residuals from best linear fit
