# Supplementary material for: Splice-Junction-Based Mapping of Alternative Isoforms in the Human Proteome
Source: Cell Rep. Author manuscript; Available in PMC 2020 Jan 15. (PMC6961840; doi:10.1016/j.celrep.2019.11.026)
Supplement: 2 [file NIHMS1546469-supplement-2.zip › DF1/PXD000561/Heart/Heart_11_ATP5A1_EIVTNFLAGFEYFCTR.pdf]

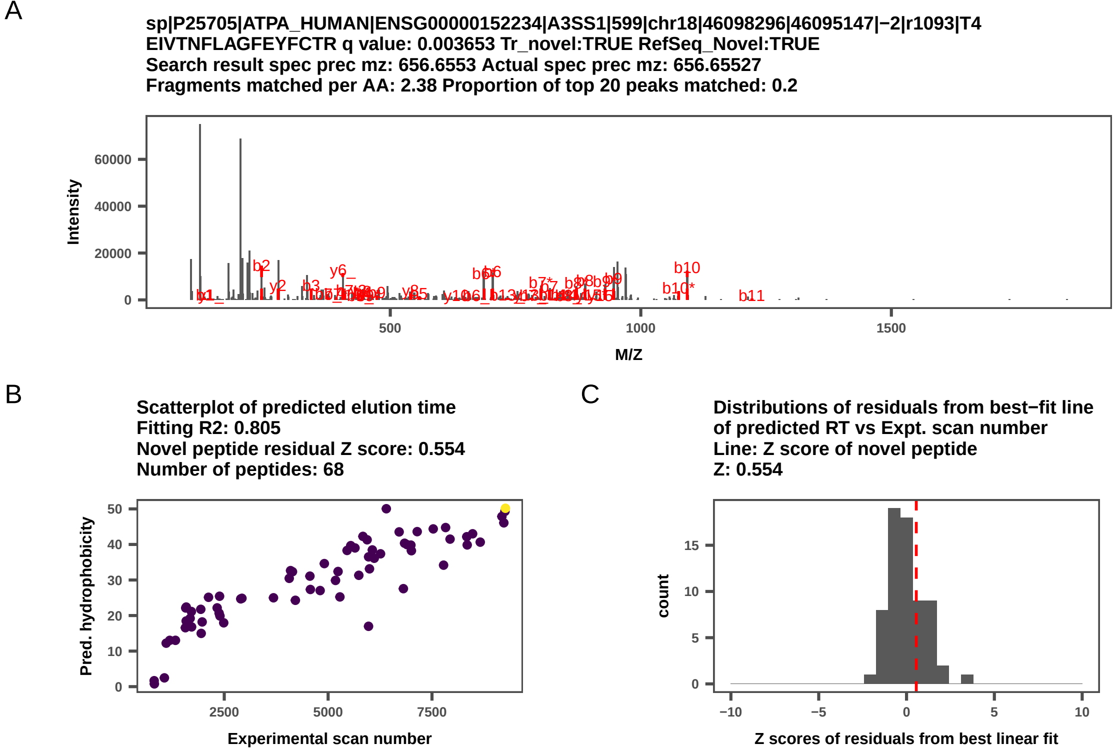
A
sp|P25705|ATPA_HUMAN|ENSG00000152234|A3SS1|599|chr18|46098296|46095147|−2|r1093|T4
EIVTNFLAGFEYFCTR q value: 0.003653 Tr_novel:TRUE RefSeq_Novel:TRUE
Search result spec prec mz: 656.6553 Actual spec prec mz: 656.65527
Fragments matched per AA: 2.38 Proportion of top 20 peaks matched: 0.2
Intensity
60000
40000
20000
0
b2
y2
y6_
b3
y8
b6
b7*
b8
b9
b10
b10*
b11
500
1000
1500
M/Z
B
Scatterplot of predicted elution time
Fitting R2: 0.805
Novel peptide residual Z score: 0.554
Number of peptides: 68
Pred. hydrophobicity
50
40
30
20
10
0
2500
5000
7500
Experimental scan number
C
Distributions of residuals from best−fit line
of predicted RT vs Expt. scan number
Line: Z score of novel peptide
Z: 0.554
count
15
10
5
0
−10
−5
0
5
10
Z scores of residuals from best linear fit
