# Supplementary material for: Splice-Junction-Based Mapping of Alternative Isoforms in the Human Proteome
Source: Cell Rep. Author manuscript; Available in PMC 2020 Jan 15. (PMC6961840; doi:10.1016/j.celrep.2019.11.026)

A

sp|Q8WZ42|TITIN_HUMAN|ENSG00000155657|MXE2|1096|chr2|178682903|178683291|−2|r762|T1,sp|Q8WZ42|TITIN
RVEAEPAEEVTIMEEK q value: 0.00013375 Tr_novel:TRUE RefSeq_Novel:TRUE
Search result spec prec mz: 930.4529 Actual spec prec mz: 930.45288
Fragments matched per AA: 2.06 Proportion of top 20 peaks matched: 0.3

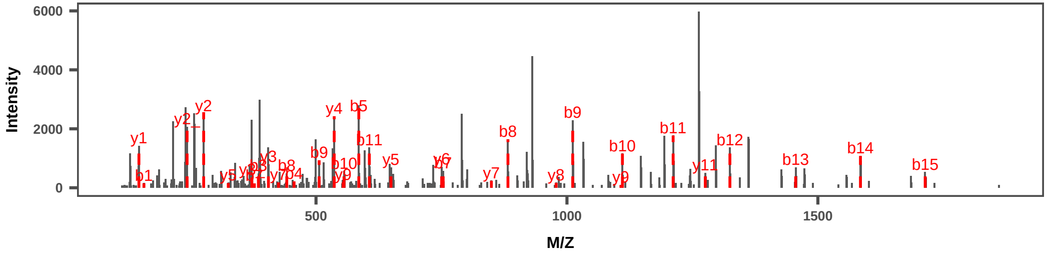

B

Scatterplot of predicted elution time
Fitting R2: 0.853
Novel peptide residual Z score: 0.346
Number of peptides: 716

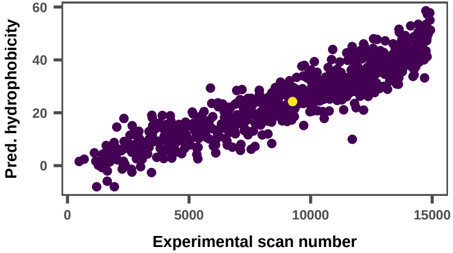

C

Distributions of residuals from best−fit line
of predicted RT vs Expt. scan number
Line: Z score of novel peptide
Z: 0.346

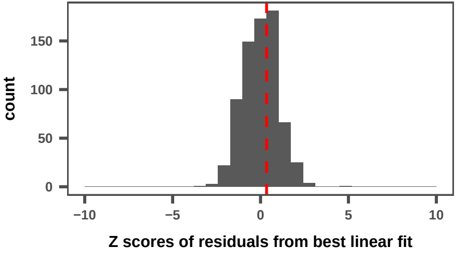

Supplement: 2 [file NIHMS1546469-supplement-2.zip › DF1/PXD000561/Heart/Heart_5_TTN_RVEAEPAEEVTIMEEK.pdf]
