# Supplementary material for: Splice-Junction-Based Mapping of Alternative Isoforms in the Human Proteome
Source: Cell Rep. Author manuscript; Available in PMC 2020 Jan 15. (PMC6961840; doi:10.1016/j.celrep.2019.11.026)
Supplement: 2 [file NIHMS1546469-supplement-2.zip › DF1/PXD000561/Heart/Heart_21_ZNF568_IQPGLNSCVTMER.pdf]

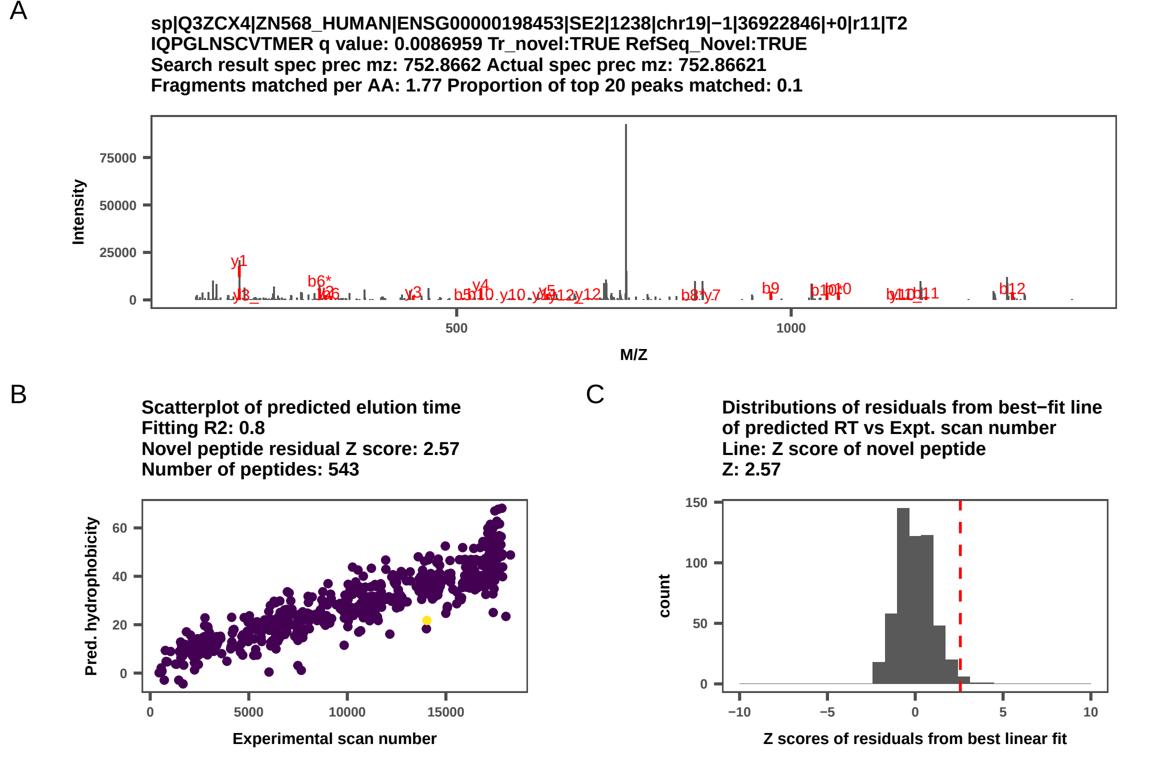
A
sp|Q3ZCX4|ZN568_HUMAN|ENSG00000198453|SE2|1238|chr19|−1|36922846|+0|r11|T2
IQPGLNSCVTMER q value: 0.0086959 Tr_novel:TRUE RefSeq_Novel:TRUE
Search result spec prec mz: 752.8662 Actual spec prec mz: 752.86621
Fragments matched per AA: 1.77 Proportion of top 20 peaks matched: 0.1
Intensity
75000
50000
25000
0
y1
b6*
y3
y4
y5
b9
b12
b8*y7
500
1000
M/Z
B
Scatterplot of predicted elution time
Fitting R2: 0.8
Novel peptide residual Z score: 2.57
Number of peptides: 543
Pred. hydrophobicity
60
40
20
0
0
5000
10000
15000
Experimental scan number
C
Distributions of residuals from best−fit line
of predicted RT vs Expt. scan number
Line: Z score of novel peptide
Z: 2.57
count
150
100
50
0
−10
−5
0
5
10
Z scores of residuals from best linear fit
