# Supplementary material for: Splice-Junction-Based Mapping of Alternative Isoforms in the Human Proteome
Source: Cell Rep. Author manuscript; Available in PMC 2020 Jan 15. (PMC6961840; doi:10.1016/j.celrep.2019.11.026)
Supplement: 2 [file NIHMS1546469-supplement-2.zip › DF1/PXD000561/Heart/Heart_10_SETMAR_QENLPVGAWPPGAAPAPFQTMK.pdf]

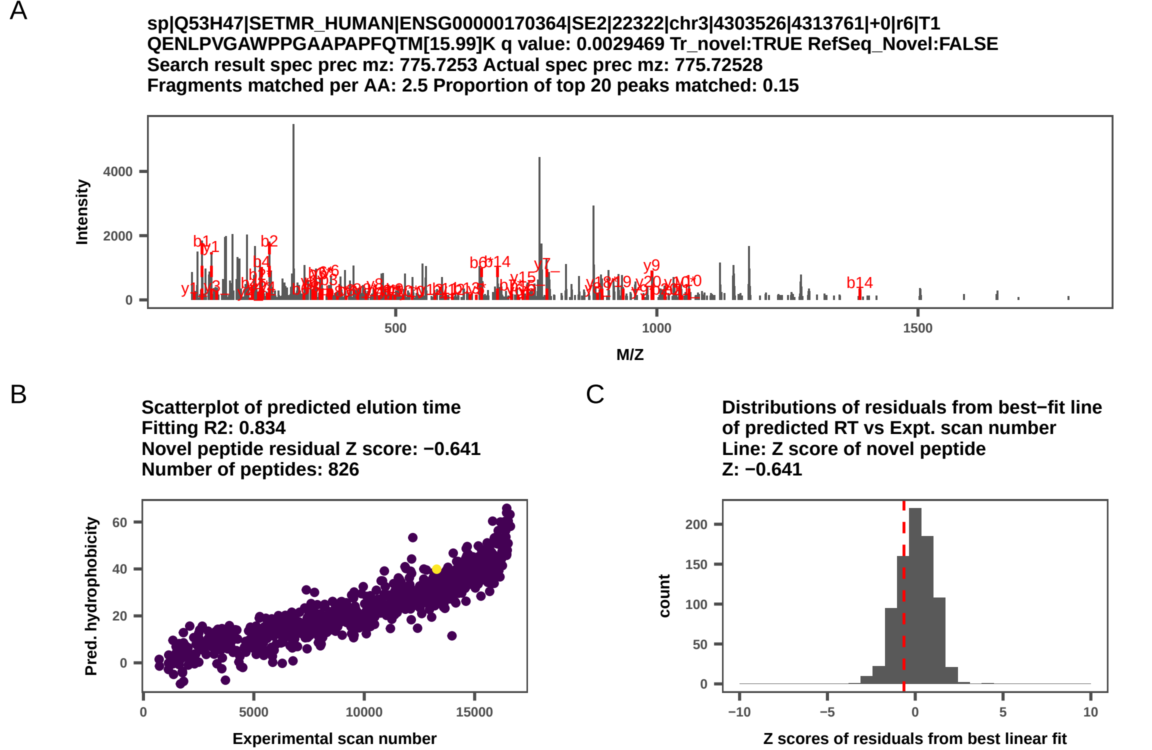
A
sp|Q53H47|SETMR_HUMAN|ENSG00000170364|SE2|22322|chr3|4303526|4313761|+0|r6|T1
QENLPVGAWPPGAAPAPFQTM[15.99]K q value: 0.0029469 Tr_novel:TRUE RefSeq_Novel:FALSE
Search result spec prec mz: 775.7253 Actual spec prec mz: 775.72528
Fragments matched per AA: 2.5 Proportion of top 20 peaks matched: 0.15
Intensity
4000
2000
0
500
1000
1500
M/Z
b1
y1
b2
b4
y9
b14
B
Scatterplot of predicted elution time
Fitting R2: 0.834
Novel peptide residual Z score: −0.641
Number of peptides: 826
Pred. hydrophobicity
60
40
20
0
5000
10000
15000
Experimental scan number
C
Distributions of residuals from best−fit line
of predicted RT vs Expt. scan number
Line: Z score of novel peptide
Z: −0.641
count
200
150
100
50
−10
−5
5
10
Z scores of residuals from best linear fit
