# Supplementary material for: Splice-Junction-Based Mapping of Alternative Isoforms in the Human Proteome
Source: Cell Rep. Author manuscript; Available in PMC 2020 Jan 15. (PMC6961840; doi:10.1016/j.celrep.2019.11.026)
Supplement: 2 [file NIHMS1546469-supplement-2.zip › DF1/PXD000561/Heart/Heart_6_MYBPC3_AITQLLCETEGR.pdf]

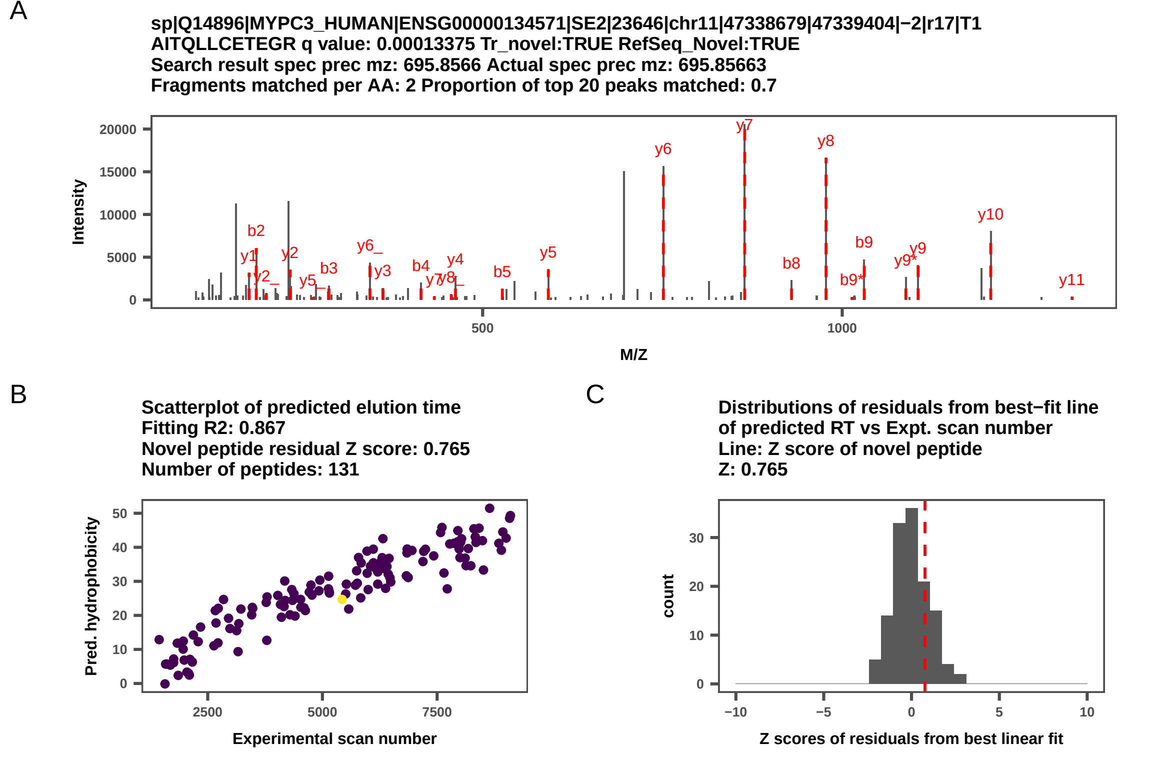

A
sp|Q14896|MYPC3_HUMAN|ENSG00000134571|SE2|23646|chr11|47338679|47339404|−2|r17|T1
AITQLLCETEGR q value: 0.00013375 Tr_novel:TRUE RefSeq_Novel:TRUE
Search result spec prec mz: 695.8566 Actual spec prec mz: 695.85663
Fragments matched per AA: 2 Proportion of top 20 peaks matched: 0.7
Intensity
20000
15000
10000
5000
0
b2
y1
y2_
y2
y5_
b3
y6_
y3
b4
y4
b5
y5
y6
y7
b8
y8
b9*
b9
y9*
y9
y10
y11
500
1000
M/Z
B
Scatterplot of predicted elution time
Fitting R2: 0.867
Novel peptide residual Z score: 0.765
Number of peptides: 131
Pred. hydrophobicity
50
40
30
20
10
0
2500
5000
7500
Experimental scan number
C
Distributions of residuals from best−fit line
of predicted RT vs Expt. scan number
Line: Z score of novel peptide
Z: 0.765
count
30
20
10
0
−10
−5
0
5
10
Z scores of residuals from best linear fit
