# Supplementary material for: Splice-Junction-Based Mapping of Alternative Isoforms in the Human Proteome
Source: Cell Rep. Author manuscript; Available in PMC 2020 Jan 15. (PMC6961840; doi:10.1016/j.celrep.2019.11.026)
Supplement: 2 [file NIHMS1546469-supplement-2.zip › DF1/PXD000561/Heart/Heart_13_BBS1_EGQSAPLLSAHVNMPGSEGLAAPNRPLNPE.pdf]

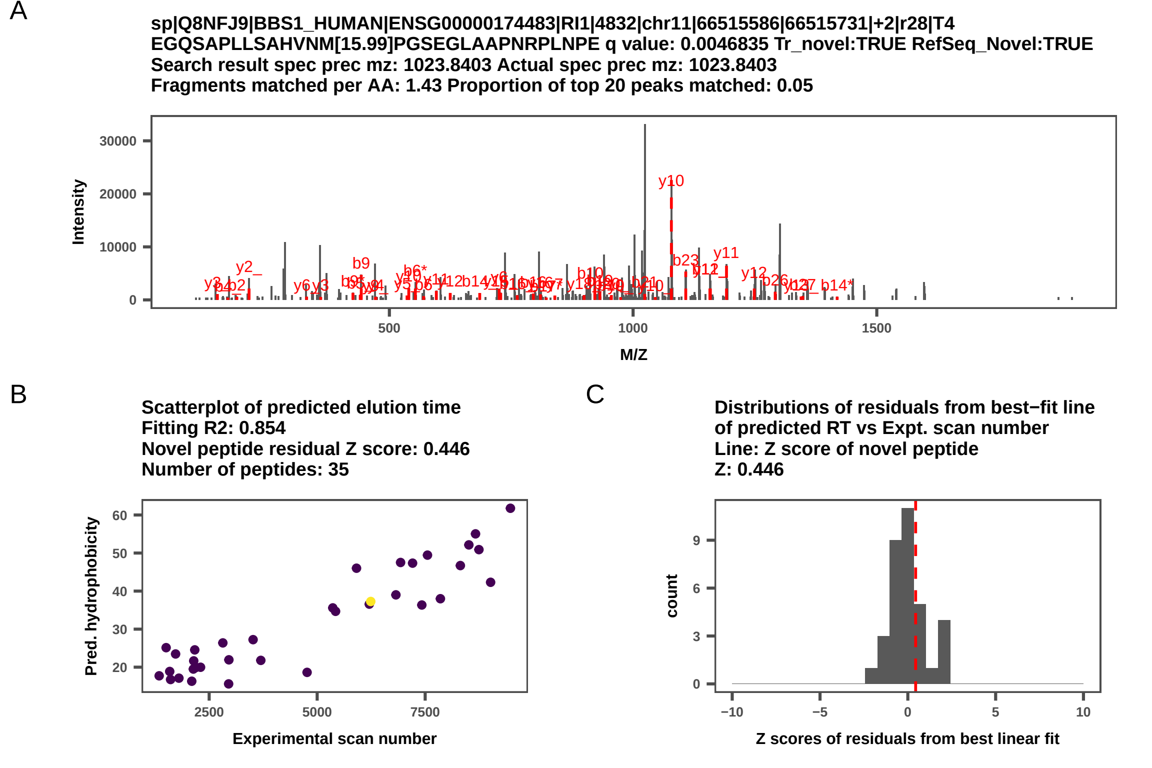
A
sp|Q8NFJ9|BBS1_HUMAN|ENSG00000174483|RI1|4832|chr11|66515586|66515731|+2|r28|T4
EGQSAPLLSAHVNM[15.99]PGSEGLAAPNRPLNPE q value: 0.0046835 Tr_novel:TRUE RefSeq_Novel:TRUE
Search result spec prec mz: 1023.8403 Actual spec prec mz: 1023.8403
Fragments matched per AA: 1.43 Proportion of top 20 peaks matched: 0.05
Intensity
30000
20000
10000
0
500
1000
1500
M/Z
y10
y11
b23
y12
b9
y2_
b26
b14*
b6*
B
Scatterplot of predicted elution time
Fitting R2: 0.854
Novel peptide residual Z score: 0.446
Number of peptides: 35
Pred. hydrophobicity
60
50
40
30
20
2500
5000
7500
Experimental scan number
C
Distributions of residuals from best−fit line
of predicted RT vs Expt. scan number
Line: Z score of novel peptide
Z: 0.446
count
9
6
3
0
−10
−5
0
5
10
Z scores of residuals from best linear fit
