# Supplementary material for: Splice-Junction-Based Mapping of Alternative Isoforms in the Human Proteome
Source: Cell Rep. Author manuscript; Available in PMC 2020 Jan 15. (PMC6961840; doi:10.1016/j.celrep.2019.11.026)
Supplement: 2 [file NIHMS1546469-supplement-2.zip › DF1/PXD000561/Heart/Heart_1_MYBPC3_RTDSHEDTGILDFSSLLK.pdf]

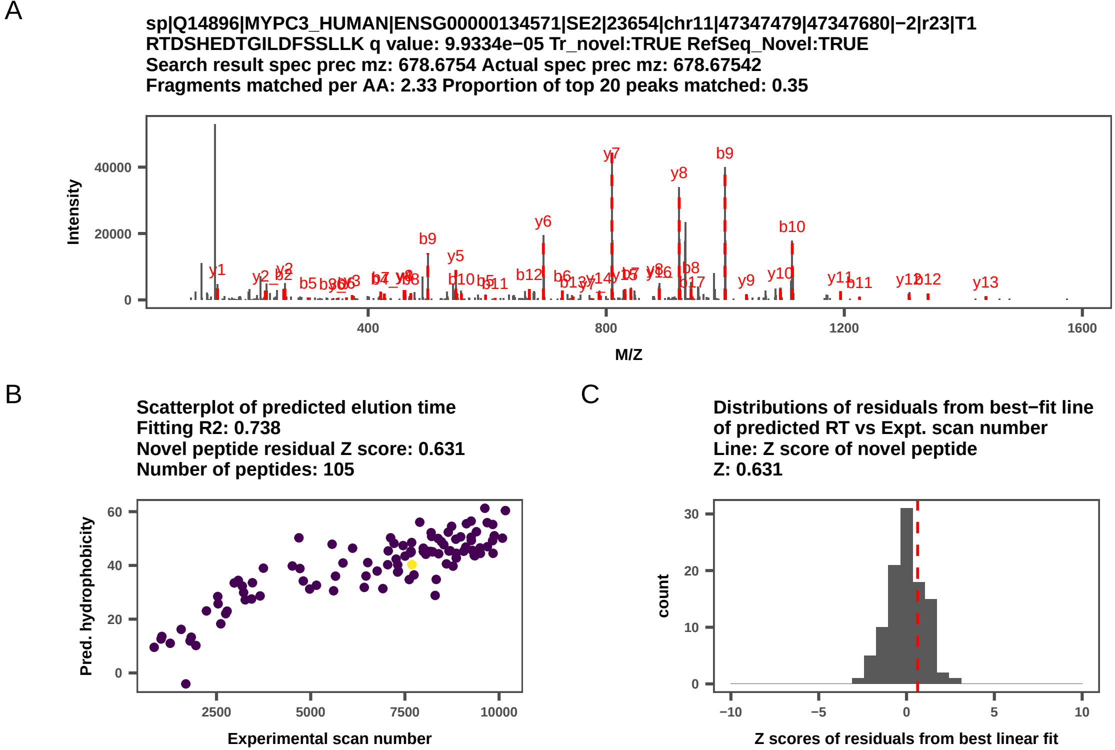
A
sp|Q14896|MYPC3_HUMAN|ENSG00000134571|SE2|23654|chr11|47347479|47347680|−2|r23|T1
RTDSHEDTGILDFSSLLK q value: 9.9334e−05 Tr_novel:TRUE RefSeq_Novel:TRUE
Search result spec prec mz: 678.6754 Actual spec prec mz: 678.67542
Fragments matched per AA: 2.33 Proportion of top 20 peaks matched: 0.35
Intensity
0
20000
40000
y7
b9
y8
y6
b10
b9
y5
y1
M/Z
400
800
1200
1600
B
Scatterplot of predicted elution time
Fitting R2: 0.738
Novel peptide residual Z score: 0.631
Number of peptides: 105
Pred. hydrophobicity
0
20
40
60
2500
5000
7500
10000
Experimental scan number
C
Distributions of residuals from best−fit line
of predicted RT vs Expt. scan number
Line: Z score of novel peptide
Z: 0.631
count
0
10
20
30
−10
−5
0
5
10
Z scores of residuals from best linear fit
