# Supplementary material for: Splice-Junction-Based Mapping of Alternative Isoforms in the Human Proteome
Source: Cell Rep. Author manuscript; Available in PMC 2020 Jan 15. (PMC6961840; doi:10.1016/j.celrep.2019.11.026)
Supplement: 2 [file NIHMS1546469-supplement-2.zip › DF1/PXD000561/Heart/Heart_20_NDUFA5_DSGLVGLAVCNTPHER.pdf]

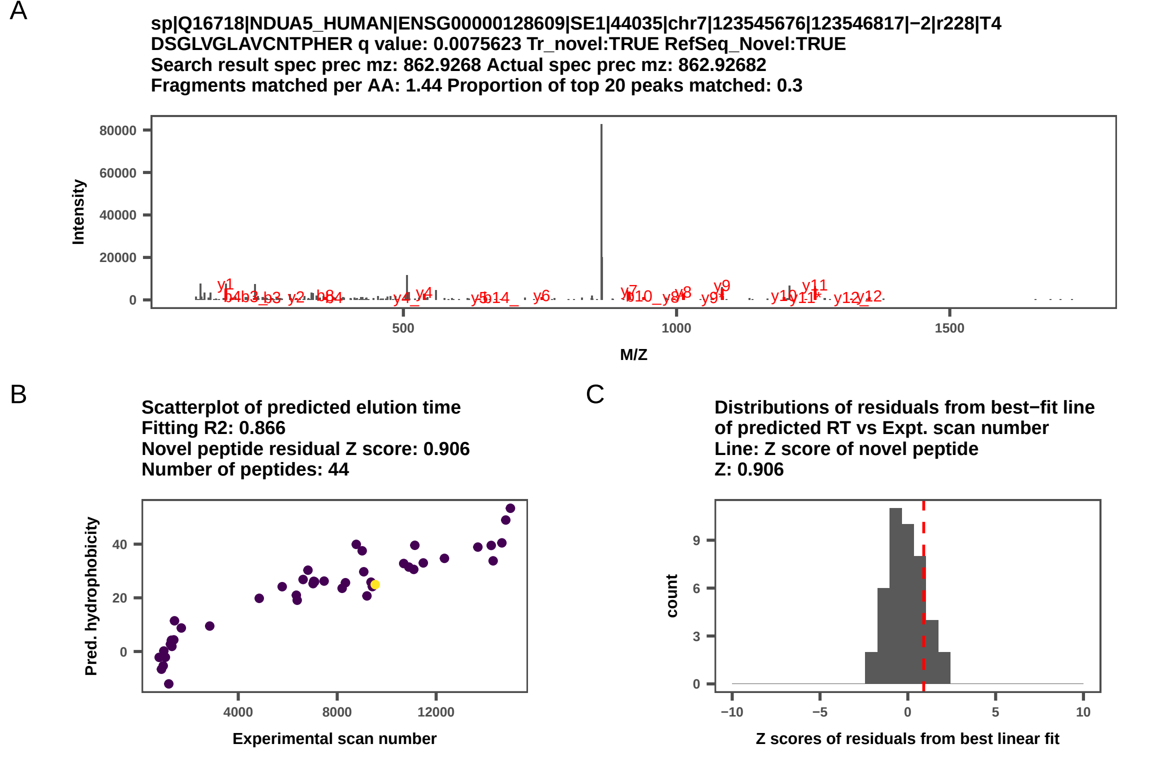
A
sp|Q16718|NDUA5_HUMAN|ENSG00000128609|SE1|44035|chr7|123545676|123546817|−2|r228|T4
DSGLVGLAVCNTPHER q value: 0.0075623 Tr_novel:TRUE RefSeq_Novel:TRUE
Search result spec prec mz: 862.9268 Actual spec prec mz: 862.92682
Fragments matched per AA: 1.44 Proportion of top 20 peaks matched: 0.3
Intensity
80000
60000
40000
20000
0
500
1000
1500
M/Z
B
Scatterplot of predicted elution time
Fitting R2: 0.866
Novel peptide residual Z score: 0.906
Number of peptides: 44
Pred. hydrophobicity
40
20
0
4000
8000
12000
Experimental scan number
C
Distributions of residuals from best−fit line
of predicted RT vs Expt. scan number
Line: Z score of novel peptide
Z: 0.906
count
9
6
3
0
−10
−5
0
5
10
Z scores of residuals from best linear fit
