# Supplementary material for: Splice-Junction-Based Mapping of Alternative Isoforms in the Human Proteome
Source: Cell Rep. Author manuscript; Available in PMC 2020 Jan 15. (PMC6961840; doi:10.1016/j.celrep.2019.11.026)
Supplement: 2 [file NIHMS1546469-supplement-2.zip › DF1/PXD000561/Colon/Colon_3_CNKSR1_VGASSHYLHLQVPVSRPGPPTPR.pdf]

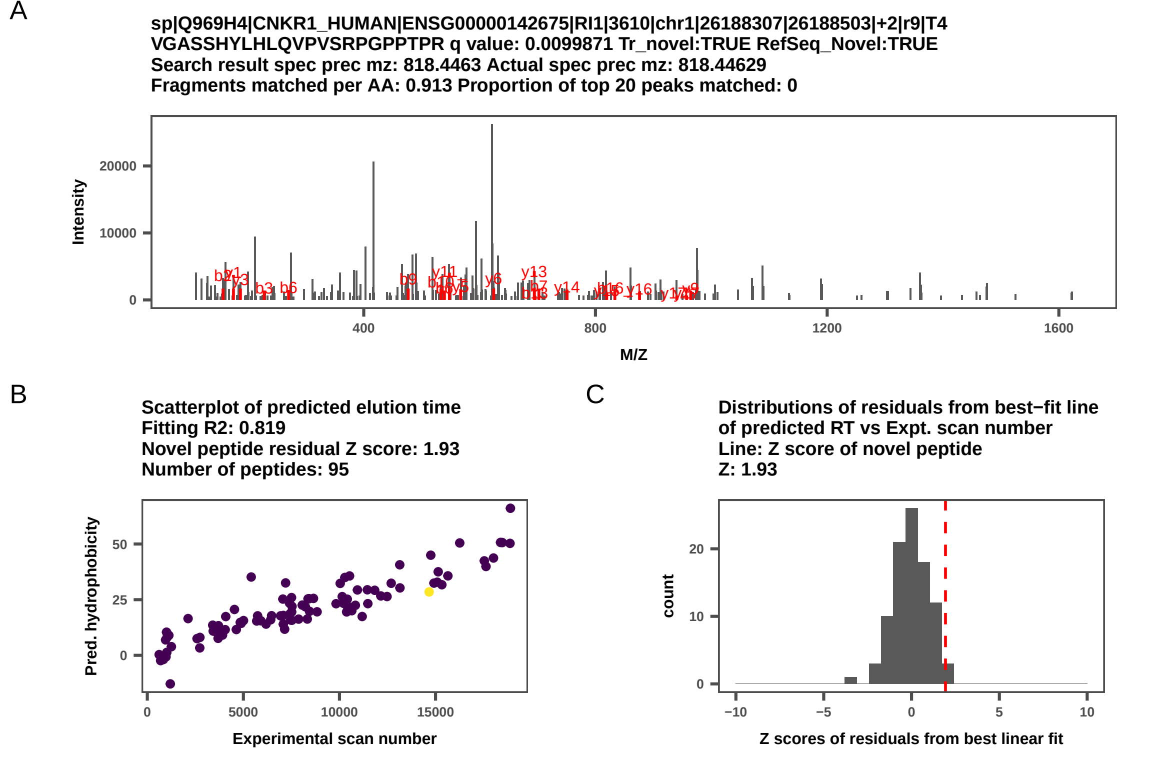
A
sp|Q969H4|CNKR1_HUMAN|ENSG00000142675|RI1|3610|chr1|26188307|26188503|+2|r9|T4
VGASSHYLHLQVPVSRPGPPTPR q value: 0.0099871 Tr_novel:TRUE RefSeq_Novel:TRUE
Search result spec prec mz: 818.4463 Actual spec prec mz: 818.44629
Fragments matched per AA: 0.913 Proportion of top 20 peaks matched: 0
Intensity
20000
10000
0
b3
b6
b9
y11
y6
y13
y14
y16
400
800
1200
1600
M/Z
B
Scatterplot of predicted elution time
Fitting R2: 0.819
Novel peptide residual Z score: 1.93
Number of peptides: 95
Pred. hydrophobicity
50
25
0
0
5000
10000
15000
Experimental scan number
C
Distributions of residuals from best−fit line
of predicted RT vs Expt. scan number
Line: Z score of novel peptide
Z: 1.93
count
20
10
0
−10
−5
0
5
10
Z scores of residuals from best linear fit
