# Supplementary material for: Splice-Junction-Based Mapping of Alternative Isoforms in the Human Proteome
Source: Cell Rep. Author manuscript; Available in PMC 2020 Jan 15. (PMC6961840; doi:10.1016/j.celrep.2019.11.026)
Supplement: 2 [file NIHMS1546469-supplement-2.zip › DF1/PXD000561/Colon/Colon_2_GABBR1_THSPTLFLRPPGAGGAQTPNATSEGCQI.pdf]

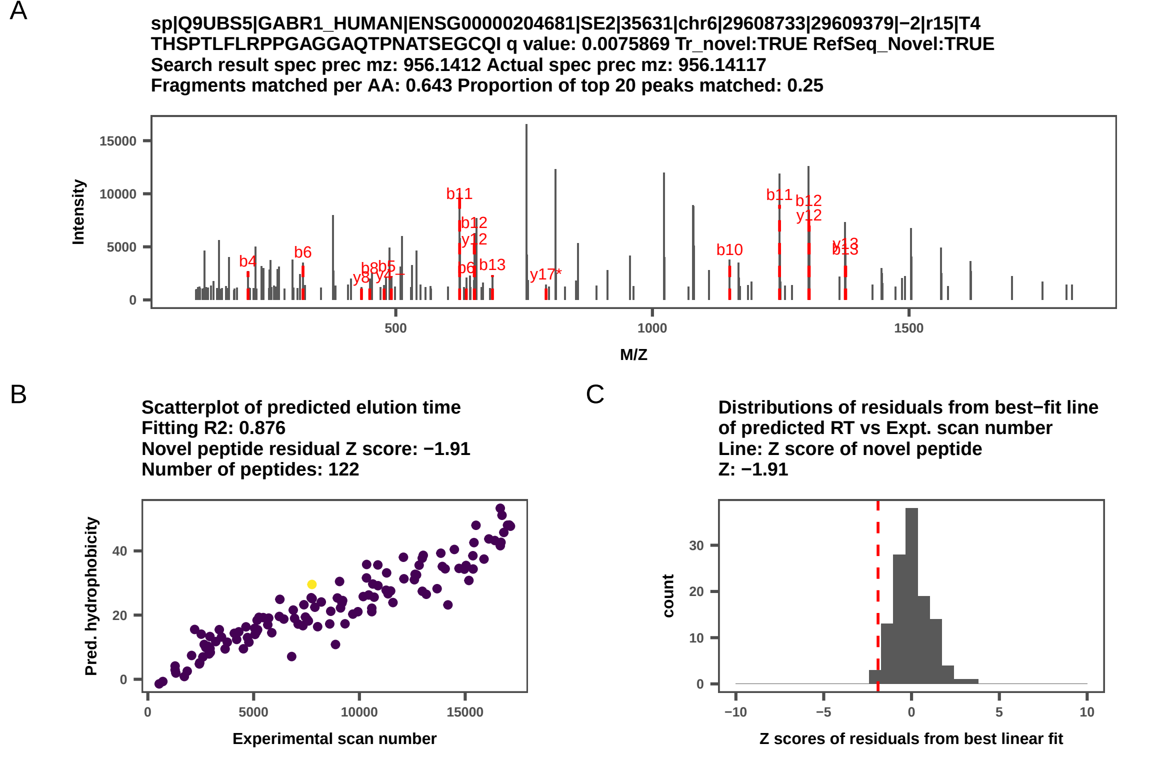
A
sp|Q9UBS5|GABR1_HUMAN|ENSG00000204681|SE2|35631|chr6|29608733|29609379|−2|r15|T4
THSPTLFLRPPGAGGAQTPNATSEGCQI q value: 0.0075869 Tr_novel:TRUE RefSeq_Novel:TRUE
Search result spec prec mz: 956.1412 Actual spec prec mz: 956.14117
Fragments matched per AA: 0.643 Proportion of top 20 peaks matched: 0.25
Intensity
15000
10000
5000
0
b4
b6
y8
b8b5
y4−
b11
b12
y12
b6
b13
y17*
b10
b11
b12
y12
y13
b13
500
1000
1500
M/Z
B
Scatterplot of predicted elution time
Fitting R2: 0.876
Novel peptide residual Z score: −1.91
Number of peptides: 122
Pred. hydrophobicity
40
20
0
0
5000
10000
15000
Experimental scan number
C
Distributions of residuals from best−fit line
of predicted RT vs Expt. scan number
Line: Z score of novel peptide
Z: −1.91
count
30
20
10
0
−10
−5
0
5
10
Z scores of residuals from best linear fit
