# Supplementary material for: Splice-Junction-Based Mapping of Alternative Isoforms in the Human Proteome
Source: Cell Rep. Author manuscript; Available in PMC 2020 Jan 15. (PMC6961840; doi:10.1016/j.celrep.2019.11.026)
Supplement: 2 [file NIHMS1546469-supplement-2.zip › DF1/PXD000561/Esophagus/Esophagus_1_SRSF11_VTAQPDVLEVQAEYITAGPGPSGGPGGGG.pdf]

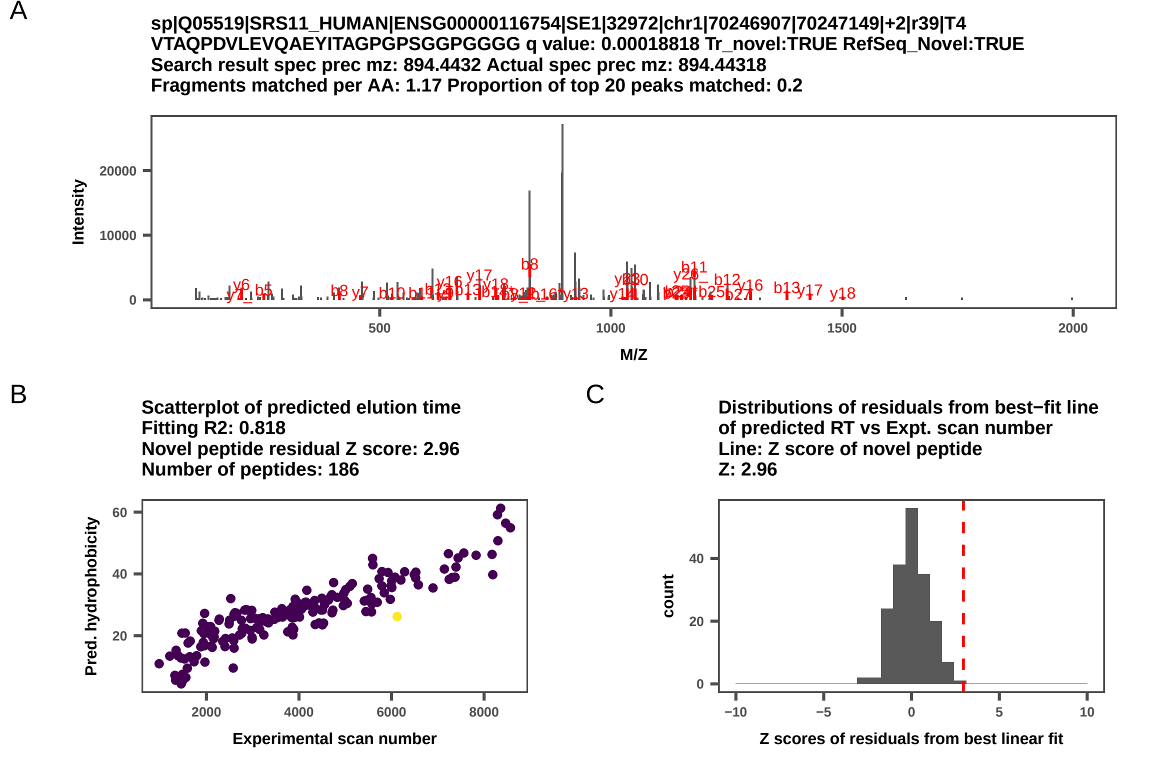
A
sp|Q05519|SRS11_HUMAN|ENSG00000116754|SE1|32972|chr1|70246907|70247149|+2|r39|T4
VTAQPDVLEVQAEYITAGPGPSGGPGGGG q value: 0.00018818 Tr_novel:TRUE RefSeq_Novel:TRUE
Search result spec prec mz: 894.4432 Actual spec prec mz: 894.44318
Fragments matched per AA: 1.17 Proportion of top 20 peaks matched: 0.2
Intensity
20000
10000
0
500
1000
1500
2000
M/Z
B
Scatterplot of predicted elution time
Fitting R2: 0.818
Novel peptide residual Z score: 2.96
Number of peptides: 186
Pred. hydrophobicity
60
40
20
2000
4000
6000
8000
Experimental scan number
C
Distributions of residuals from best−fit line
of predicted RT vs Expt. scan number
Line: Z score of novel peptide
Z: 2.96
count
40
20
0
−10
−5
0
5
10
Z scores of residuals from best linear fit
