# Supplementary material for: Splice-Junction-Based Mapping of Alternative Isoforms in the Human Proteome
Source: Cell Rep. Author manuscript; Available in PMC 2020 Jan 15. (PMC6961840; doi:10.1016/j.celrep.2019.11.026)
Supplement: 2 [file NIHMS1546469-supplement-2.zip › DF1/PXD000561/Esophagus/Esophagus_3_HMGB1_ENILACPLVMLR.pdf]

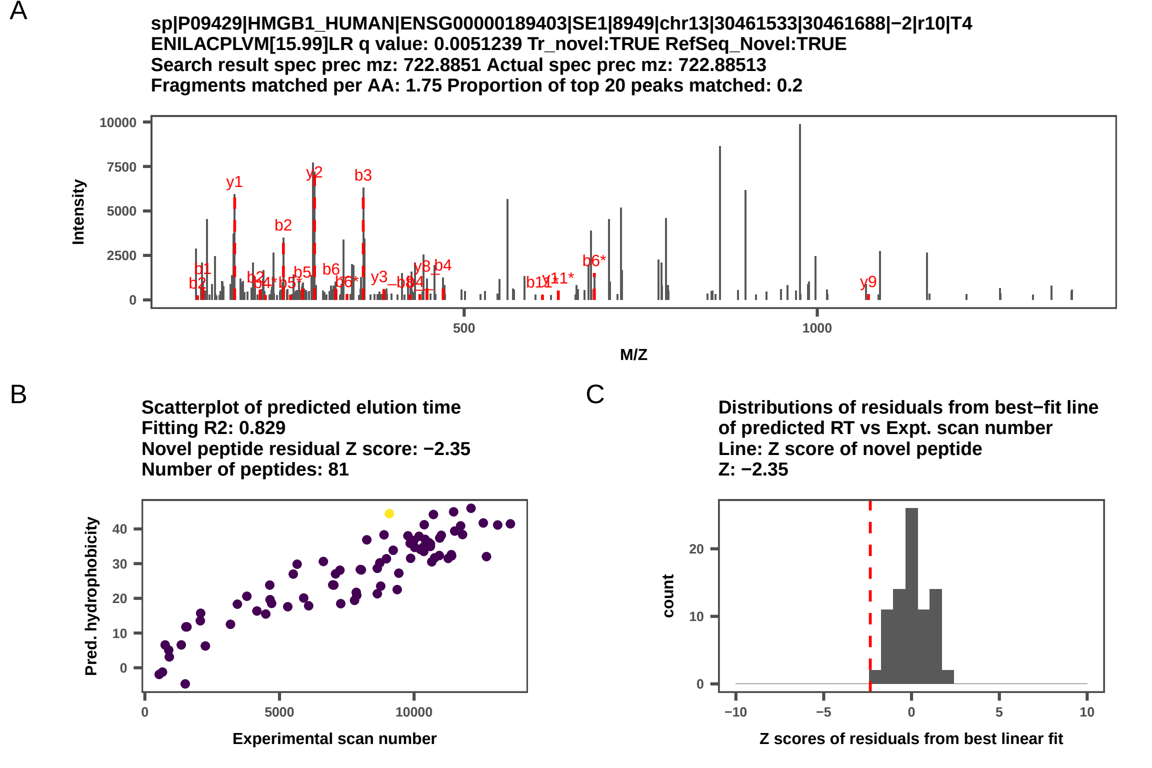
A
sp|P09429|HMGB1_HUMAN|ENSG00000189403|SE1|8949|chr13|30461533|30461688|−2|r10|T4
ENILACPLVM[15.99]LR q value: 0.0051239 Tr_novel:TRUE RefSeq_Novel:TRUE
Search result spec prec mz: 722.8851 Actual spec prec mz: 722.88513
Fragments matched per AA: 1.75 Proportion of top 20 peaks matched: 0.2
Intensity
10000
7500
5000
2500
0
y1
y2
b3
b2
b6*
y9
500
1000
M/Z
B
Scatterplot of predicted elution time
Fitting R2: 0.829
Novel peptide residual Z score: −2.35
Number of peptides: 81
Pred. hydrophobicity
40
30
20
10
0
0
5000
10000
Experimental scan number
C
Distributions of residuals from best−fit line
of predicted RT vs Expt. scan number
Line: Z score of novel peptide
Z: −2.35
count
20
10
0
−10
−5
0
5
10
Z scores of residuals from best linear fit
