# Supplementary material for: Splice-Junction-Based Mapping of Alternative Isoforms in the Human Proteome
Source: Cell Rep. Author manuscript; Available in PMC 2020 Jan 15. (PMC6961840; doi:10.1016/j.celrep.2019.11.026)
Supplement: 2 [file NIHMS1546469-supplement-2.zip › DF1/PXD000561/Esophagus/Esophagus_2_RFWD2_QLEAQLEQIQK.pdf]

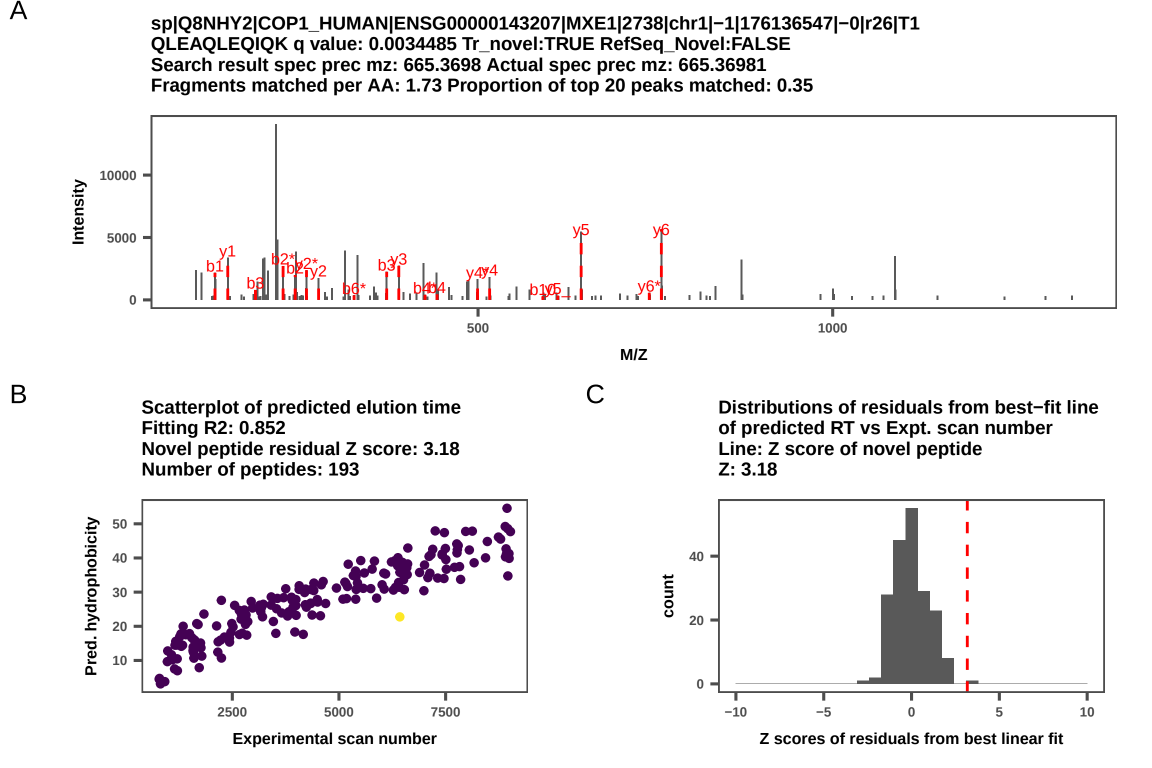
A
sp|Q8NHY2|COP1_HUMAN|ENSG00000143207|MXE1|2738|chr1|−1|176136547|−0|r26|T1
QLEAQLEQIQK q value: 0.0034485 Tr_novel:TRUE RefSeq_Novel:FALSE
Search result spec prec mz: 665.3698 Actual spec prec mz: 665.36981
Fragments matched per AA: 1.73 Proportion of top 20 peaks matched: 0.35
Intensity
10000
5000
0
y1
b1
b3
b2*
b2
y2*
y2
b6*
b3
y3
b4*
b4
y4*
y4
y5
y6
y6*
500
1000
M/Z
B
Scatterplot of predicted elution time
Fitting R2: 0.852
Novel peptide residual Z score: 3.18
Number of peptides: 193
Pred. hydrophobicity
50
40
30
20
10
2500
5000
7500
Experimental scan number
C
Distributions of residuals from best−fit line
of predicted RT vs Expt. scan number
Line: Z score of novel peptide
Z: 3.18
count
40
20
0
−10
−5
0
5
10
Z scores of residuals from best linear fit
