# Supplementary material for: Splice-Junction-Based Mapping of Alternative Isoforms in the Human Proteome
Source: Cell Rep. Author manuscript; Available in PMC 2020 Jan 15. (PMC6961840; doi:10.1016/j.celrep.2019.11.026)
Supplement: 2 [file NIHMS1546469-supplement-2.zip › DF1/PXD000561/Pancreas/Pancreas_3_HMGB1_ENILACPLVMLR.pdf]

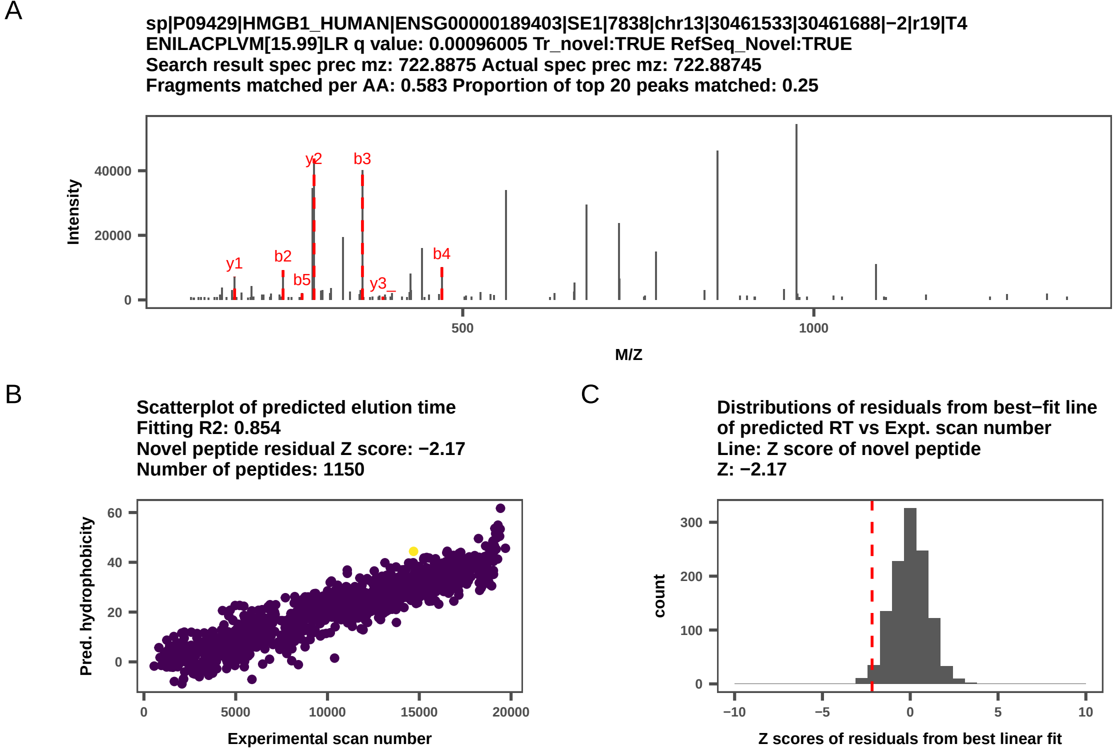
A
sp|P09429|HMGB1_HUMAN|ENSG00000189403|SE1|7838|chr13|30461533|30461688|−2|r19|T4
ENILACPLVM[15.99]LR q value: 0.00096005 Tr_novel:TRUE RefSeq_Novel:TRUE
Search result spec prec mz: 722.8875 Actual spec prec mz: 722.88745
Fragments matched per AA: 0.583 Proportion of top 20 peaks matched: 0.25
Intensity
40000
20000
0
y1
b2
b5
y2
b3
y3_
b4
500
1000
M/Z
B
Scatterplot of predicted elution time
Fitting R2: 0.854
Novel peptide residual Z score: −2.17
Number of peptides: 1150
Pred. hydrophobicity
60
40
20
0
0
5000
10000
15000
20000
Experimental scan number
C
Distributions of residuals from best−fit line
of predicted RT vs Expt. scan number
Line: Z score of novel peptide
Z: −2.17
count
300
200
100
0
−10
−5
0
5
10
Z scores of residuals from best linear fit
