# Supplementary material for: Splice-Junction-Based Mapping of Alternative Isoforms in the Human Proteome
Source: Cell Rep. Author manuscript; Available in PMC 2020 Jan 15. (PMC6961840; doi:10.1016/j.celrep.2019.11.026)
Supplement: 2 [file NIHMS1546469-supplement-2.zip › DF1/PXD000561/Pancreas/Pancreas_6_SYPL1_ETSLHSPSNTSAPHSQGGIPPPTGL.pdf]

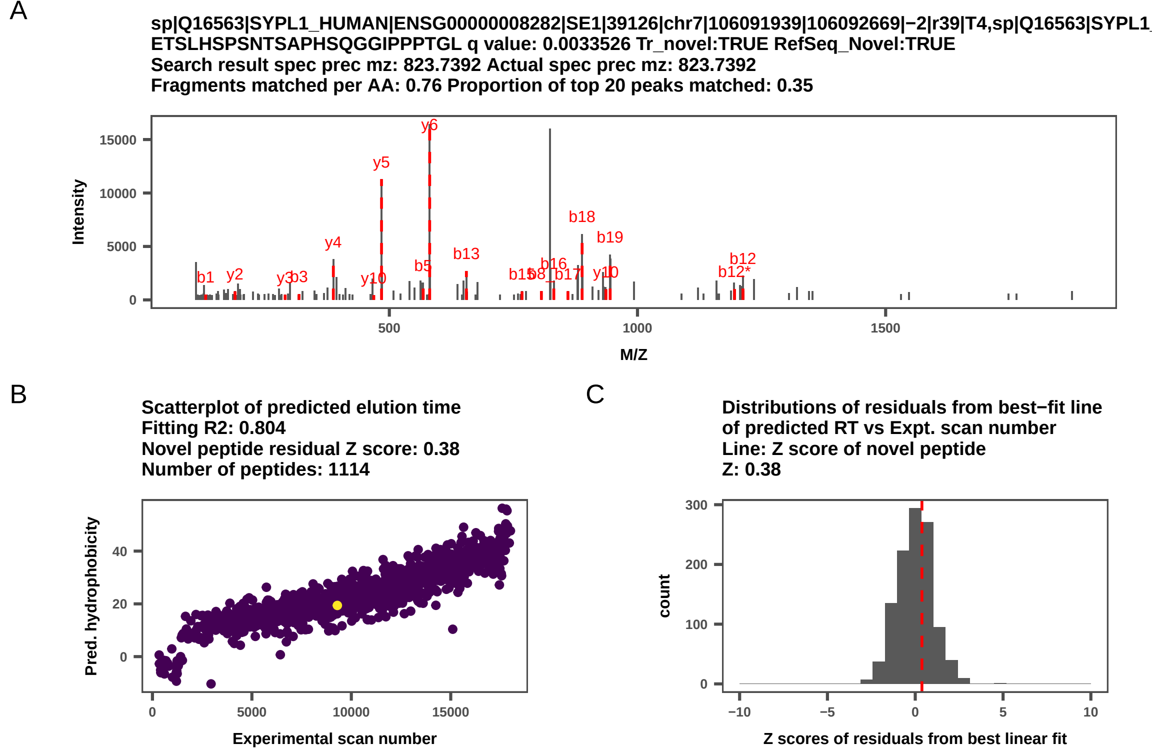
A
sp|Q16563|SYPL1_HUMAN|ENSG00000008282|SE1|39126|chr7|106091939|106092669|−2|r39|T4,sp|Q16563|SYPL1_
ETSLHSPSNTSAPHSQGGIPPPTGL q value: 0.0033526 Tr_novel:TRUE RefSeq_Novel:TRUE
Search result spec prec mz: 823.7392 Actual spec prec mz: 823.7392
Fragments matched per AA: 0.76 Proportion of top 20 peaks matched: 0.35
Intensity
15000
10000
5000
0
y6
y5
y4
b1
y2
y3
b3
y10
b5
b13
b18
b19
b16
b17
y10
b12
b12*
500
1000
1500
M/Z
B
Scatterplot of predicted elution time
Fitting R2: 0.804
Novel peptide residual Z score: 0.38
Number of peptides: 1114
Pred. hydrophobicity
40
20
0
0
5000
10000
15000
Experimental scan number
C
Distributions of residuals from best−fit line
of predicted RT vs Expt. scan number
Line: Z score of novel peptide
Z: 0.38
count
300
200
100
0
−10
−5
0
5
10
Z scores of residuals from best linear fit
