# Supplementary material for: Splice-Junction-Based Mapping of Alternative Isoforms in the Human Proteome
Source: Cell Rep. Author manuscript; Available in PMC 2020 Jan 15. (PMC6961840; doi:10.1016/j.celrep.2019.11.026)
Supplement: 2 [file NIHMS1546469-supplement-2.zip › DF1/PXD000561/Pancreas/Pancreas_2_EIF3G_TCPHLQDPSWADQVEEEGEDDKCVTSELLK.pdf]

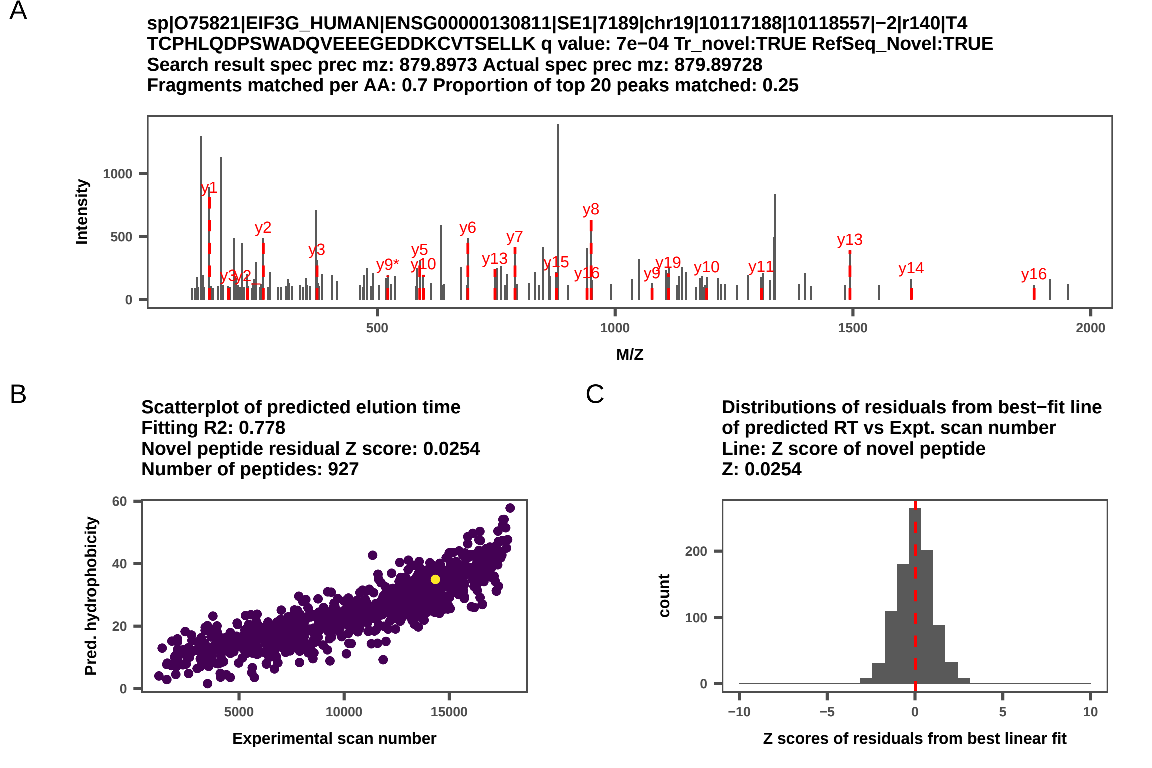
A
sp|O75821|EIF3G_HUMAN|ENSG00000130811|SE1|7189|chr19|10117188|10118557|−2|r140|T4
TCPHLQDPSWADQVEEEGEDDKCVTSELLK q value: 7e−04 Tr_novel:TRUE RefSeq_Novel:TRUE
Search result spec prec mz: 879.8973 Actual spec prec mz: 879.89728
Fragments matched per AA: 0.7 Proportion of top 20 peaks matched: 0.25
Intensity
0
500
1000
M/Z
500
1000
1500
2000
y1
y2
y3
y9*
y5
y10
y6
y13
y7
y15
y8
y16
y9
y19
y10
y11
y13
y14
y16
B
Scatterplot of predicted elution time
Fitting R2: 0.778
Novel peptide residual Z score: 0.0254
Number of peptides: 927
Pred. hydrophobicity
0
20
40
60
Experimental scan number
5000
10000
15000
C
Distributions of residuals from best−fit line
of predicted RT vs Expt. scan number
Line: Z score of novel peptide
Z: 0.0254
count
0
100
200
Z scores of residuals from best linear fit
−10
−5
0
5
10
