# Supplementary material for: Splice-Junction-Based Mapping of Alternative Isoforms in the Human Proteome
Source: Cell Rep. Author manuscript; Available in PMC 2020 Jan 15. (PMC6961840; doi:10.1016/j.celrep.2019.11.026)
Supplement: 2 [file NIHMS1546469-supplement-2.zip › DF1/PXD000561/Pancreas/Pancreas_4_FARSA_LAQAGAPVGR.pdf]

A

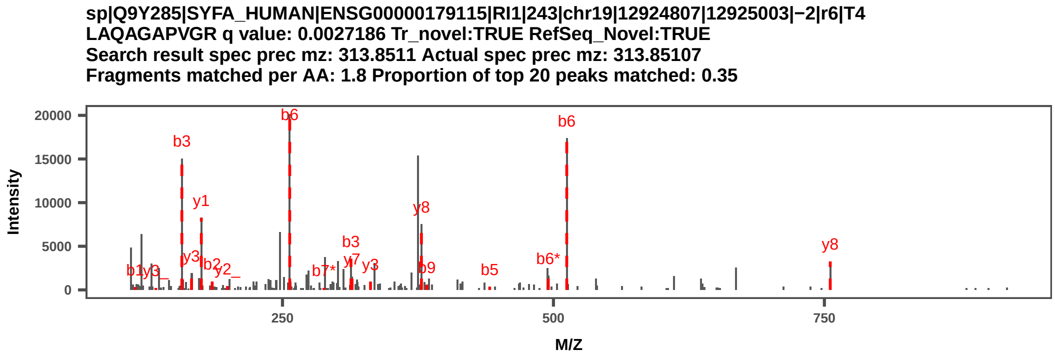
sp|Q9Y285|SYFA_HUMAN|ENSG00000179115|RI1|243|chr19|12924807|12925003|−2|r6|T4
LAQAGAPVGR q value: 0.0027186 Tr_novel:TRUE RefSeq_Novel:TRUE
Search result spec prec mz: 313.8511 Actual spec prec mz: 313.85107
Fragments matched per AA: 1.8 Proportion of top 20 peaks matched: 0.35
Intensity
M/Z
b6
b3
y1
y8
b3
y7
y3
b9
b5
b6*
y8
b7*
b1
y3_
y3
b2
y2_
20000
15000
10000
5000
0
250
500
750

B

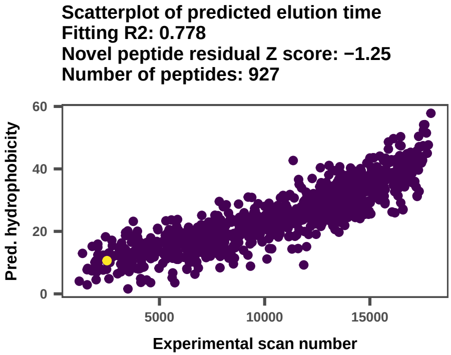
Scatterplot of predicted elution time
Fitting R2: 0.778
Novel peptide residual Z score: −1.25
Number of peptides: 927
Pred. hydrophobicity
Experimental scan number
60
40
20
0
5000
10000
15000

C

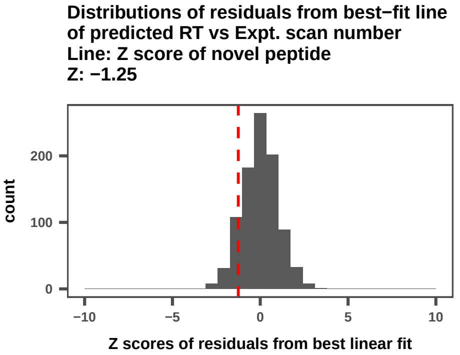
Distributions of residuals from best−fit line
of predicted RT vs Expt. scan number
Line: Z score of novel peptide
Z: −1.25
count
Z scores of residuals from best linear fit
200
100
0
−10
−5
0
5
10
