# Supplementary material for: Splice-Junction-Based Mapping of Alternative Isoforms in the Human Proteome
Source: Cell Rep. Author manuscript; Available in PMC 2020 Jan 15. (PMC6961840; doi:10.1016/j.celrep.2019.11.026)
Supplement: 2 [file NIHMS1546469-supplement-2.zip › DF1/PXD000561/Pancreas/Pancreas_5_LRCH4_VGGAAAVSTQAMHNLLKPGLR.pdf]

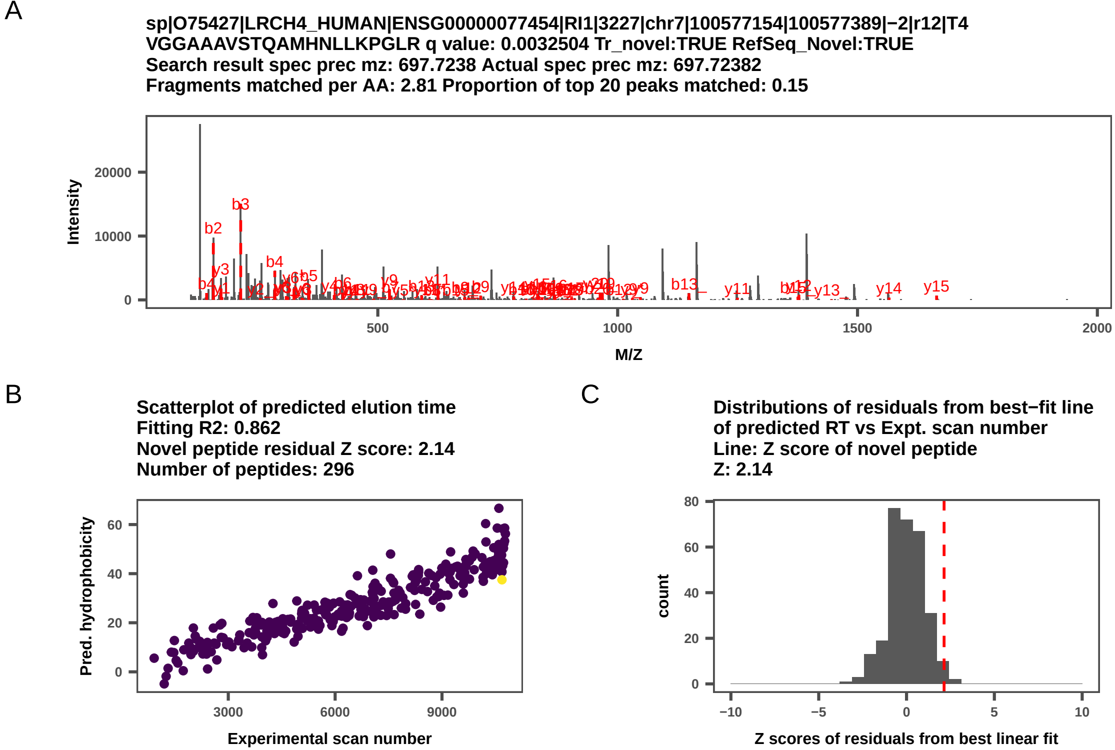
A
sp|O75427|LRCH4_HUMAN|ENSG00000077454|RI1|3227|chr7|100577154|100577389|−2|r12|T4
VGGAAAVSTQAMHNLLKPGLR q value: 0.0032504 Tr_novel:TRUE RefSeq_Novel:TRUE
Search result spec prec mz: 697.7238 Actual spec prec mz: 697.72382
Fragments matched per AA: 2.81 Proportion of top 20 peaks matched: 0.15
Intensity
20000
10000
0
b2
b3
b4
y3
b5
y9
y11
b13
y11
y13
y14
y15
500
1000
1500
2000
M/Z
B
Scatterplot of predicted elution time
Fitting R2: 0.862
Novel peptide residual Z score: 2.14
Number of peptides: 296
Pred. hydrophobicity
60
40
20
0
3000
6000
9000
Experimental scan number
C
Distributions of residuals from best−fit line
of predicted RT vs Expt. scan number
Line: Z score of novel peptide
Z: 2.14
count
80
60
40
20
0
−10
−5
0
5
10
Z scores of residuals from best linear fit
