# Supplementary material for: Splice-Junction-Based Mapping of Alternative Isoforms in the Human Proteome
Source: Cell Rep. Author manuscript; Available in PMC 2020 Jan 15. (PMC6961840; doi:10.1016/j.celrep.2019.11.026)

A

sp|P08218|CEL2B_HUMAN|ENSG00000215704|MXE1|1775|chr1|15481195|15482393|+2|r532|T4
DAGPALVAGALSCGVSTYAPDMSR q value: 0.0050408 Tr_novel:TRUE RefSeq_Novel:TRUE
Search result spec prec mz: 592.2788 Actual spec prec mz: 592.27881
Fragments matched per AA: 0.542 Proportion of top 20 peaks matched: 0.2

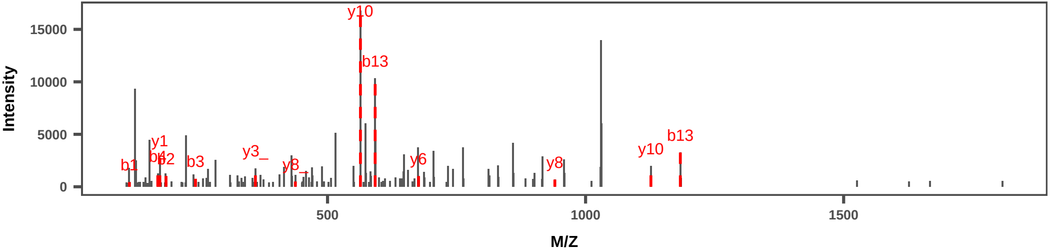

B

Scatterplot of predicted elution time
Fitting R2: 0.849
Novel peptide residual Z score: −6.4
Number of peptides: 1150

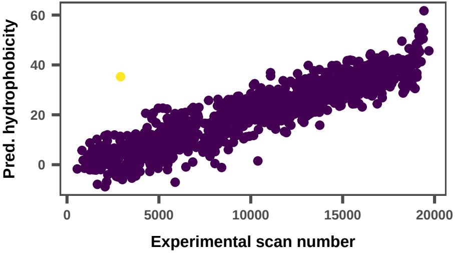

C

Distributions of residuals from best−fit line
of predicted RT vs Expt. scan number
Line: Z score of novel peptide
Z: −6.4

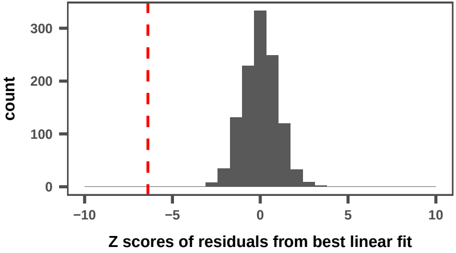

Supplement: 2 [file NIHMS1546469-supplement-2.zip › DF1/PXD000561/Pancreas/Pancreas_7_CELA2B_DAGPALVAGALSCGVSTYAPDMSR.pdf]
