# Supplementary material for: Splice-Junction-Based Mapping of Alternative Isoforms in the Human Proteome
Source: Cell Rep. Author manuscript; Available in PMC 2020 Jan 15. (PMC6961840; doi:10.1016/j.celrep.2019.11.026)
Supplement: 2 [file NIHMS1546469-supplement-2.zip › DF1/PXD000561/Liver/Liver_9_AFMID_HPGPHGRPPSK.pdf]

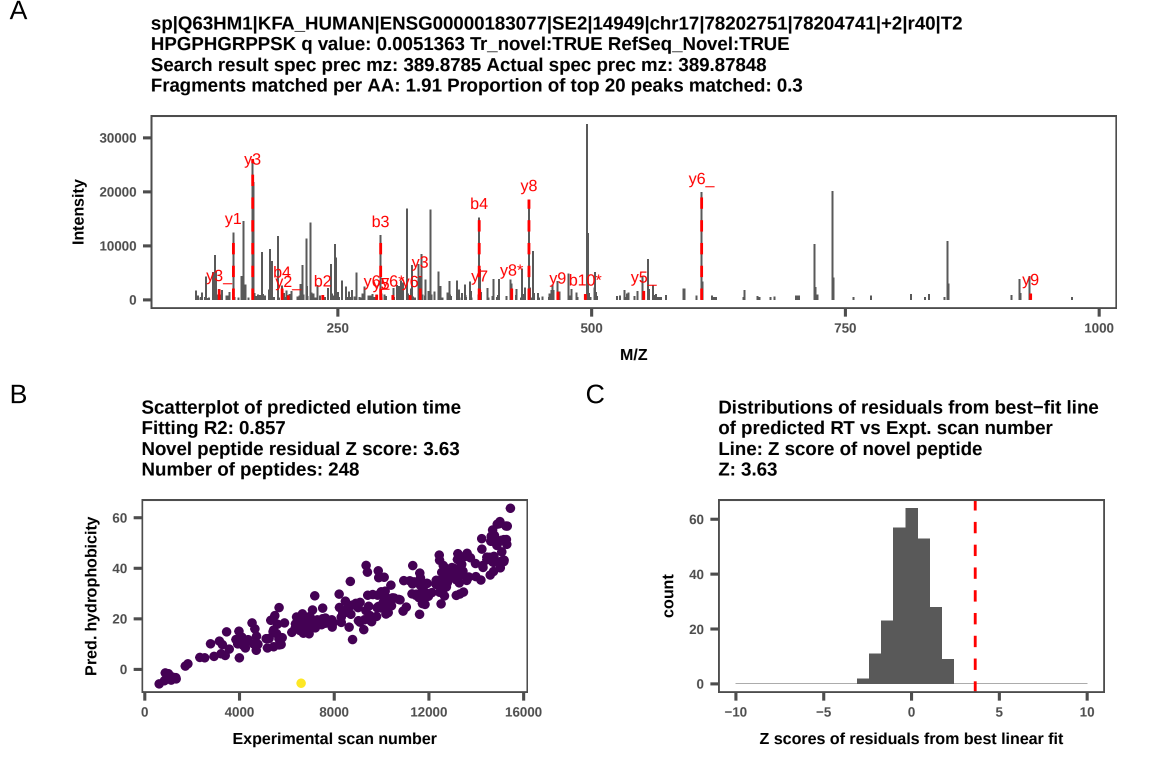
A
sp|Q63HM1|KFA_HUMAN|ENSG00000183077|SE2|14949|chr17|78202751|78204741|+2|r40|T2
HPGPHGRPPSK q value: 0.0051363 Tr_novel:TRUE RefSeq_Novel:TRUE
Search result spec prec mz: 389.8785 Actual spec prec mz: 389.87848
Fragments matched per AA: 1.91 Proportion of top 20 peaks matched: 0.3
Intensity
0
10000
20000
30000
M/Z
250
500
750
1000
y1
y3
b3
b4
y8
y6_
y9
B
Scatterplot of predicted elution time
Fitting R2: 0.857
Novel peptide residual Z score: 3.63
Number of peptides: 248
Pred. hydrophobicity
Experimental scan number
0
4000
8000
12000
16000
C
Distributions of residuals from best−fit line
of predicted RT vs Expt. scan number
Line: Z score of novel peptide
Z: 3.63
count
Z scores of residuals from best linear fit
−10
−5
0
5
10
