# Supplementary material for: Splice-Junction-Based Mapping of Alternative Isoforms in the Human Proteome
Source: Cell Rep. Author manuscript; Available in PMC 2020 Jan 15. (PMC6961840; doi:10.1016/j.celrep.2019.11.026)
Supplement: 2 [file NIHMS1546469-supplement-2.zip › DF1/PXD000561/Liver/Liver_7_ZC3H11A_KQGGEEPLVR.pdf]

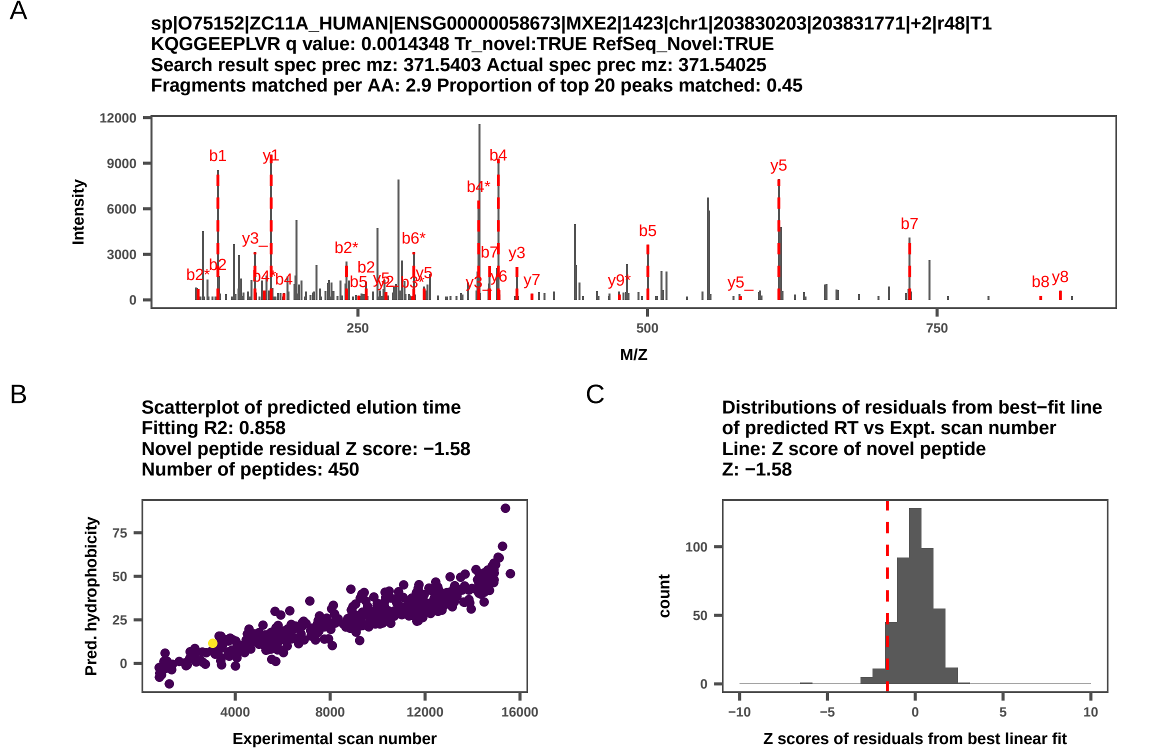
A
sp|O75152|ZC11A_HUMAN|ENSG00000058673|MXE2|1423|chr1|203830203|203831771|+2|r48|T1
KQGGEEPLVR q value: 0.0014348 Tr_novel:TRUE RefSeq_Novel:TRUE
Search result spec prec mz: 371.5403 Actual spec prec mz: 371.54025
Fragments matched per AA: 2.9 Proportion of top 20 peaks matched: 0.45
Intensity
12000
9000
6000
3000
0
b1
y1
b4
b4*
y5
b7
b5
y3_
b2*
b6*
b2
b7
y3
y7
y9*
y5_
b8
y8
M/Z
250
500
750
B
Scatterplot of predicted elution time
Fitting R2: 0.858
Novel peptide residual Z score: −1.58
Number of peptides: 450
Pred. hydrophobicity
75
50
25
0
4000
8000
12000
16000
Experimental scan number
C
Distributions of residuals from best−fit line
of predicted RT vs Expt. scan number
Line: Z score of novel peptide
Z: −1.58
count
100
50
0
−10
−5
0
5
10
Z scores of residuals from best linear fit
