# Supplementary material for: Splice-Junction-Based Mapping of Alternative Isoforms in the Human Proteome
Source: Cell Rep. Author manuscript; Available in PMC 2020 Jan 15. (PMC6961840; doi:10.1016/j.celrep.2019.11.026)
Supplement: 2 [file NIHMS1546469-supplement-2.zip › DF1/PXD000561/Liver/Liver_1_AOX1_GEGQPFEYFVYGAACSEVEIDCLTGDHKG.pdf]

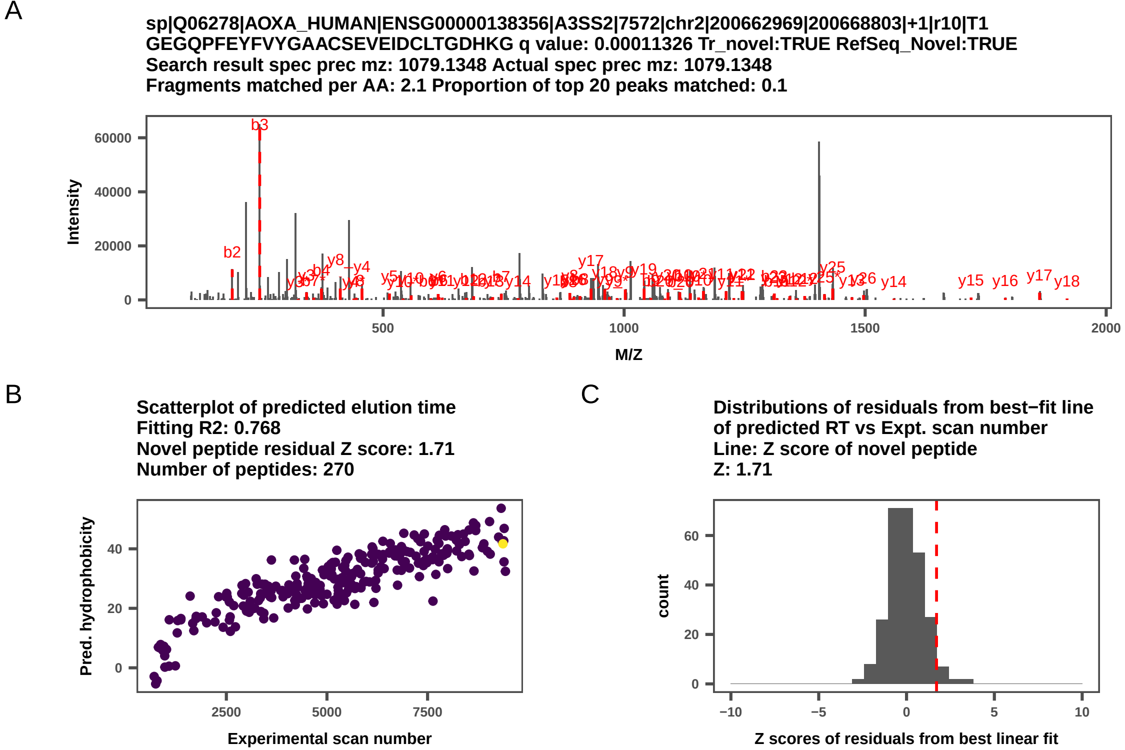
A
sp|Q06278|AOXA_HUMAN|ENSG00000138356|A3SS2|7572|chr2|200662969|200668803|+1|r10|T1
GEGQPFEYFVYGAACSEVEIDCLTGDHKG q value: 0.00011326 Tr_novel:TRUE RefSeq_Novel:TRUE
Search result spec prec mz: 1079.1348 Actual spec prec mz: 1079.1348
Fragments matched per AA: 2.1 Proportion of top 20 peaks matched: 0.1
Intensity
60000
40000
20000
0
b3
b2
y8
y17
y25
y14
y15
y16
y17
y18
500
1000
1500
2000
M/Z
B
Scatterplot of predicted elution time
Fitting R2: 0.768
Novel peptide residual Z score: 1.71
Number of peptides: 270
Pred. hydrophobicity
40
20
0
2500
5000
7500
Experimental scan number
C
Distributions of residuals from best−fit line
of predicted RT vs Expt. scan number
Line: Z score of novel peptide
Z: 1.71
count
60
40
20
0
−10
−5
0
5
10
Z scores of residuals from best linear fit
