# Supplementary material for: Splice-Junction-Based Mapping of Alternative Isoforms in the Human Proteome
Source: Cell Rep. Author manuscript; Available in PMC 2020 Jan 15. (PMC6961840; doi:10.1016/j.celrep.2019.11.026)
Supplement: 2 [file NIHMS1546469-supplement-2.zip › DF1/PXD000561/Liver/Liver_13_DHRS1_KPQNCVVTGASRGIGR.pdf]

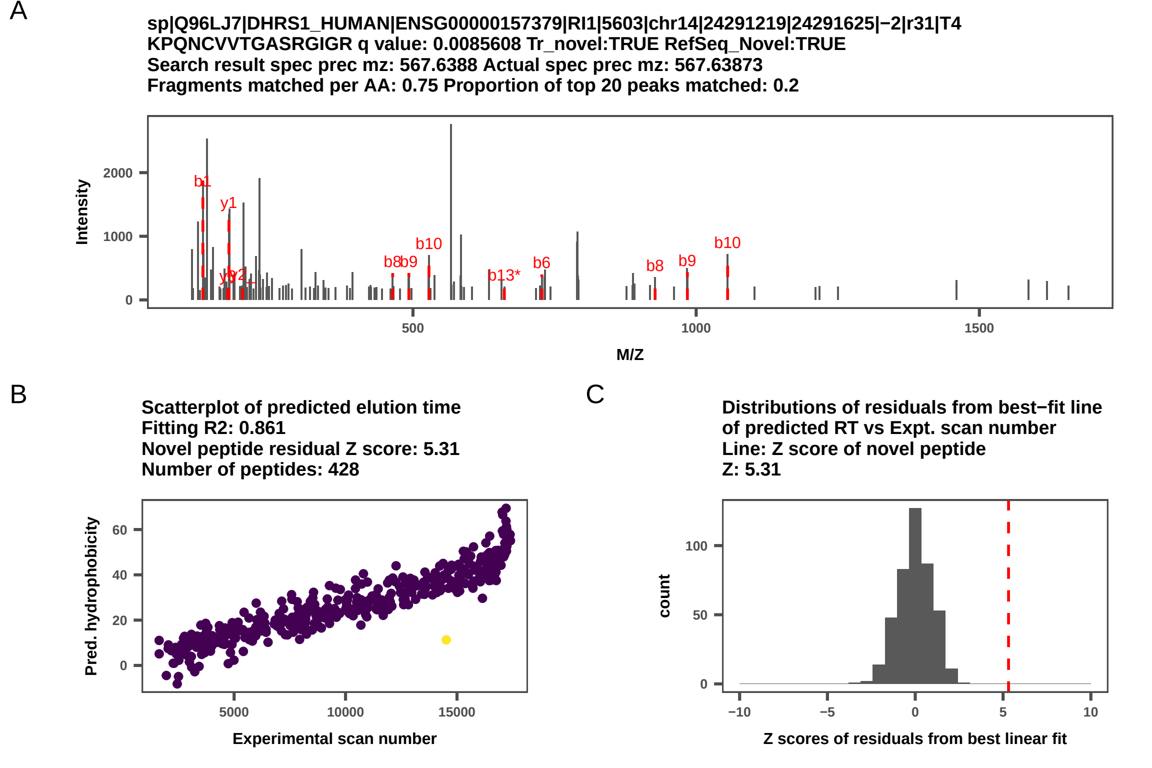
A
sp|Q96LJ7|DHRS1_HUMAN|ENSG00000157379|RI1|5603|chr14|24291219|24291625|−2|r31|T4
KPQNCVVTGASRGIGR q value: 0.0085608 Tr_novel:TRUE RefSeq_Novel:TRUE
Search result spec prec mz: 567.6388 Actual spec prec mz: 567.63873
Fragments matched per AA: 0.75 Proportion of top 20 peaks matched: 0.2
Intensity
M/Z
b1
y1
b8
b9
b10
b13*
b6
b8
b9
b10
B
Scatterplot of predicted elution time
Fitting R2: 0.861
Novel peptide residual Z score: 5.31
Number of peptides: 428
Pred. hydrophobicity
Experimental scan number
C
Distributions of residuals from best−fit line
of predicted RT vs Expt. scan number
Line: Z score of novel peptide
Z: 5.31
count
Z scores of residuals from best linear fit
