# Supplementary material for: Splice-Junction-Based Mapping of Alternative Isoforms in the Human Proteome
Source: Cell Rep. Author manuscript; Available in PMC 2020 Jan 15. (PMC6961840; doi:10.1016/j.celrep.2019.11.026)
Supplement: 2 [file NIHMS1546469-supplement-2.zip › DF1/PXD000561/Liver/Liver_8_HMGB1_ENILACPLVMLR.pdf]

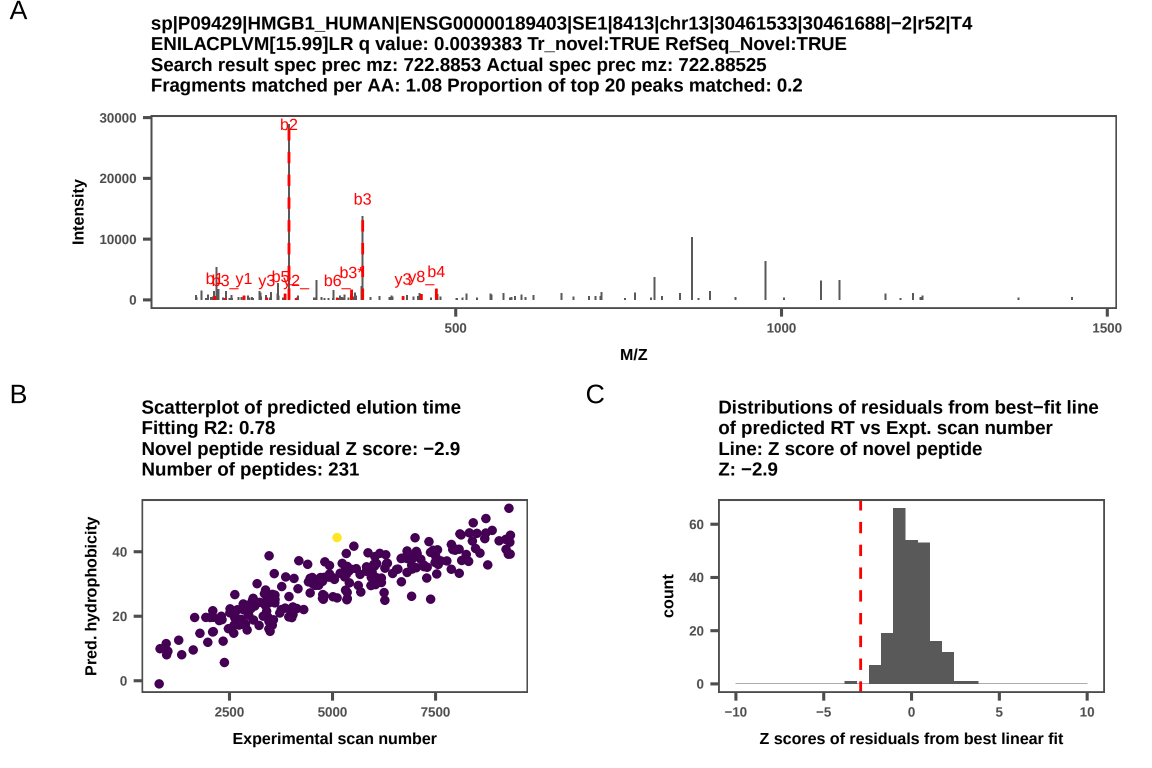
A
sp|P09429|HMGB1_HUMAN|ENSG00000189403|SE1|8413|chr13|30461533|30461688|−2|r52|T4
ENILACPLVM[15.99]LR q value: 0.0039383 Tr_novel:TRUE RefSeq_Novel:TRUE
Search result spec prec mz: 722.8853 Actual spec prec mz: 722.88525
Fragments matched per AA: 1.08 Proportion of top 20 peaks matched: 0.2
Intensity
30000
20000
10000
0
b2
b3
b4
b6_
b3*
y1
y8_
M/Z
500
1000
1500
B
Scatterplot of predicted elution time
Fitting R2: 0.78
Novel peptide residual Z score: −2.9
Number of peptides: 231
Pred. hydrophobicity
40
20
0
2500
5000
7500
Experimental scan number
C
Distributions of residuals from best−fit line
of predicted RT vs Expt. scan number
Line: Z score of novel peptide
Z: −2.9
count
60
40
20
0
−10
−5
0
5
10
Z scores of residuals from best linear fit
