# Supplementary material for: Splice-Junction-Based Mapping of Alternative Isoforms in the Human Proteome
Source: Cell Rep. Author manuscript; Available in PMC 2020 Jan 15. (PMC6961840; doi:10.1016/j.celrep.2019.11.026)
Supplement: 2 [file NIHMS1546469-supplement-2.zip › DF1/PXD000561/Liver/Liver_6_BBS1_EGQSAPLLSAHVNMPGSEGLAAPNRPLNPE.pdf]

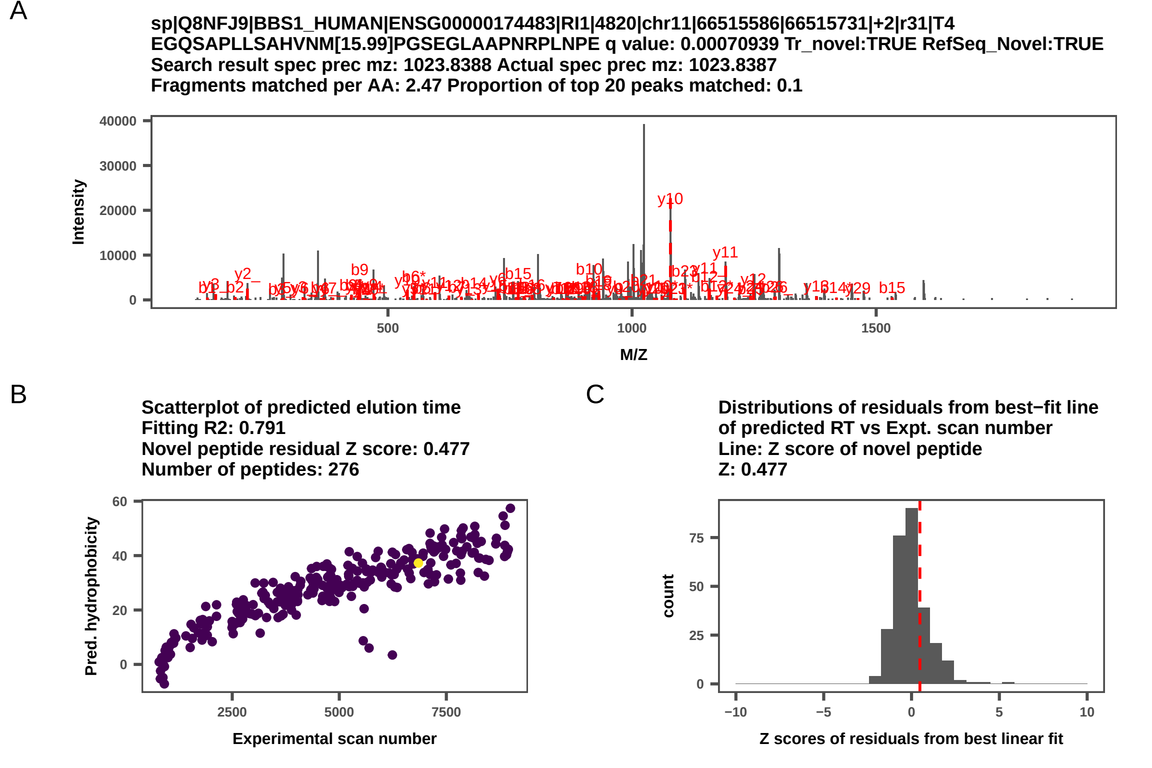
A
sp|Q8NFJ9|BBS1_HUMAN|ENSG00000174483|RI1|4820|chr11|66515586|66515731|+2|r31|T4
EGQSAPLLSAHVNM[15.99]PGSEGLAAPNRPLNPE q value: 0.00070939 Tr_novel:TRUE RefSeq_Novel:TRUE
Search result spec prec mz: 1023.8388 Actual spec prec mz: 1023.8387
Fragments matched per AA: 2.47 Proportion of top 20 peaks matched: 0.1
Intensity
40000
30000
20000
10000
0
M/Z
500
1000
1500
y10
y11
b9
b15
b10
B
Scatterplot of predicted elution time
Fitting R2: 0.791
Novel peptide residual Z score: 0.477
Number of peptides: 276
Pred. hydrophobicity
60
40
20
0
Experimental scan number
2500
5000
7500
C
Distributions of residuals from best−fit line
of predicted RT vs Expt. scan number
Line: Z score of novel peptide
Z: 0.477
count
75
50
25
0
Z scores of residuals from best linear fit
−10
−5
0
5
10
