# Supplementary material for: Splice-Junction-Based Mapping of Alternative Isoforms in the Human Proteome
Source: Cell Rep. Author manuscript; Available in PMC 2020 Jan 15. (PMC6961840; doi:10.1016/j.celrep.2019.11.026)
Supplement: 2 [file NIHMS1546469-supplement-2.zip › DF1/PXD000561/Liver/Liver_15_FGG_QVRPEHPAETEYDSLYPEDDI.pdf]

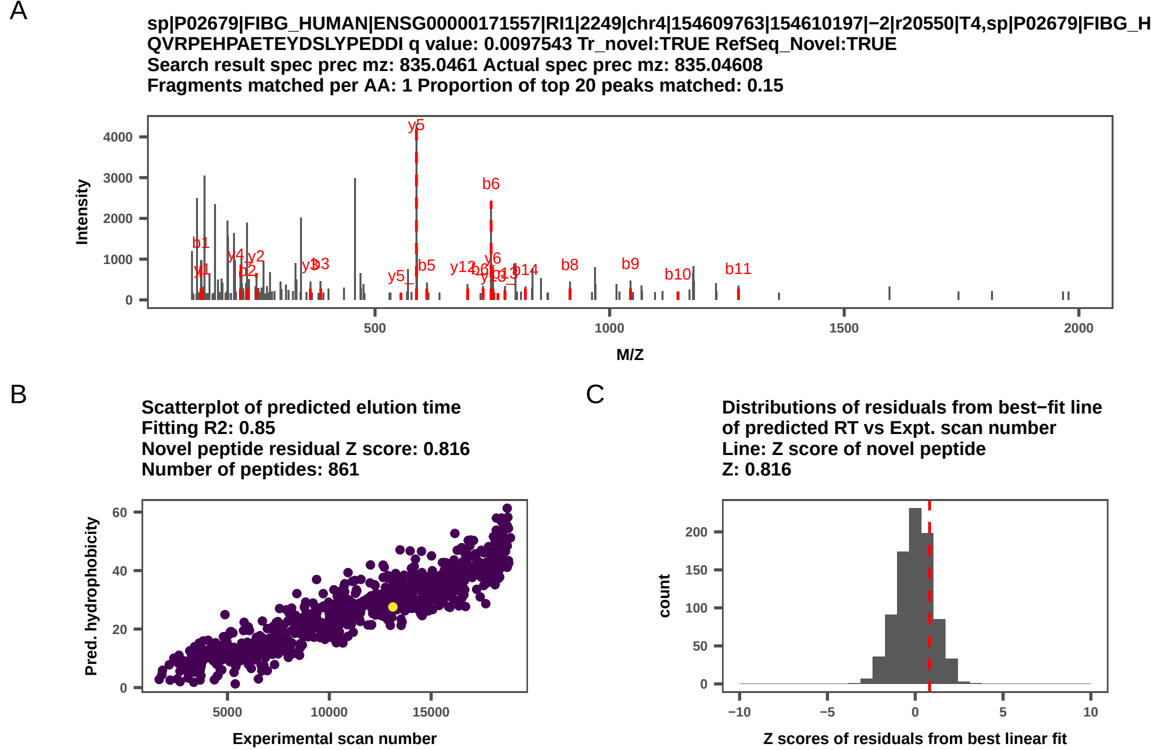
A
sp|P02679|FIBG_HUMAN|ENSG00000171557|RI1|2249|chr4|154609763|154610197|−2|r20550|T4,sp|P02679|FIBG_H
QVRPEHPAETEYDSLYPEDDI q value: 0.0097543 Tr_novel:TRUE RefSeq_Novel:TRUE
Search result spec prec mz: 835.0461 Actual spec prec mz: 835.04608
Fragments matched per AA: 1 Proportion of top 20 peaks matched: 0.15
Intensity
0
1000
2000
3000
4000
y5
b6
b1
y4
y2
b2
b3
b5
y12
y6
b14
b8
b9
b10
b11
500
1000
1500
2000
M/Z
B
Scatterplot of predicted elution time
Fitting R2: 0.85
Novel peptide residual Z score: 0.816
Number of peptides: 861
Pred. hydrophobicity
0
20
40
60
5000
10000
15000
Experimental scan number
C
Distributions of residuals from best−fit line
of predicted RT vs Expt. scan number
Line: Z score of novel peptide
Z: 0.816
count
0
50
100
150
200
−10
−5
0
5
10
Z scores of residuals from best linear fit
