# Supplementary material for: Splice-Junction-Based Mapping of Alternative Isoforms in the Human Proteome
Source: Cell Rep. Author manuscript; Available in PMC 2020 Jan 15. (PMC6961840; doi:10.1016/j.celrep.2019.11.026)
Supplement: 2 [file NIHMS1546469-supplement-2.zip › DF1/PXD000561/Liver/Liver_10_LRCH4_VGGAAAVSTQAMHNLLKPGLR.pdf]

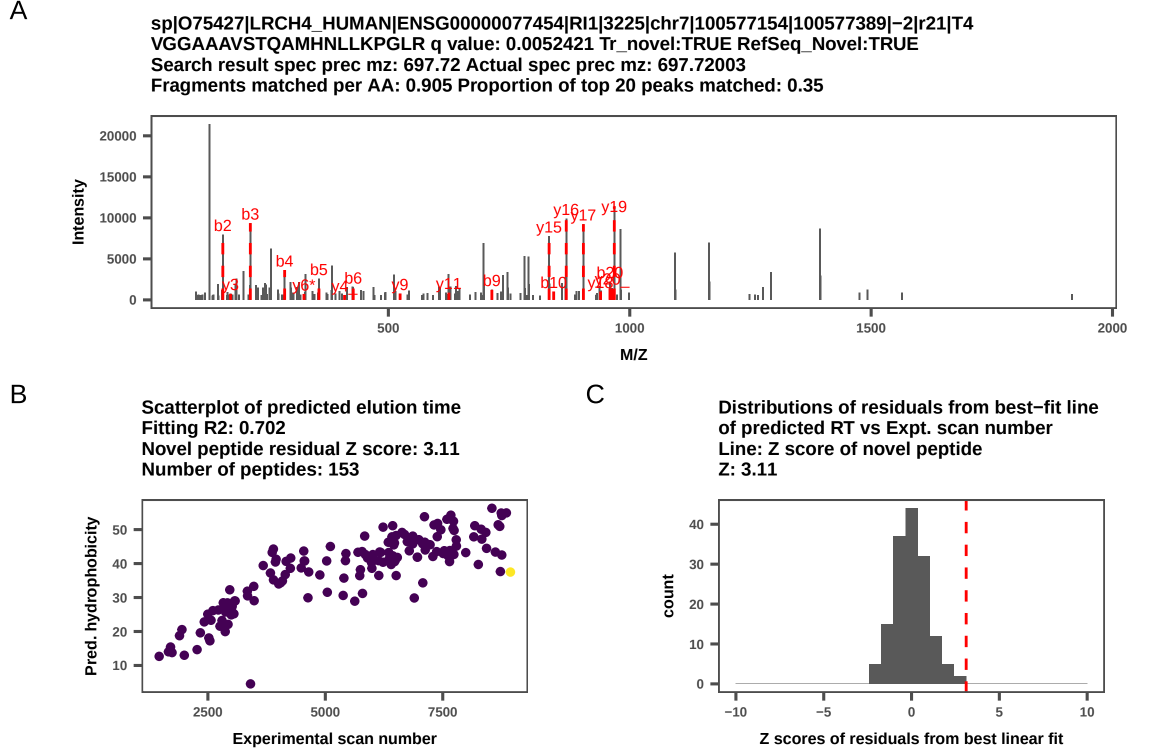
A
sp|O75427|LRCH4_HUMAN|ENSG00000077454|RI1|3225|chr7|100577154|100577389|−2|r21|T4
VGGAAAVSTQAMHNLLKPGLR q value: 0.0052421 Tr_novel:TRUE RefSeq_Novel:TRUE
Search result spec prec mz: 697.72 Actual spec prec mz: 697.72003
Fragments matched per AA: 0.905 Proportion of top 20 peaks matched: 0.35
Intensity
20000
15000
10000
5000
0
b2
b3
b4
b5
b6
y3
y4
y9
y11
b9
b10
y15
y16
y17
y19
M/Z
500
1000
1500
2000
B
Scatterplot of predicted elution time
Fitting R2: 0.702
Novel peptide residual Z score: 3.11
Number of peptides: 153
Pred. hydrophobicity
50
40
30
20
10
2500
5000
7500
Experimental scan number
C
Distributions of residuals from best−fit line
of predicted RT vs Expt. scan number
Line: Z score of novel peptide
Z: 3.11
count
40
30
20
10
0
−10
−5
0
5
10
Z scores of residuals from best linear fit
