# Supplementary material for: Splice-Junction-Based Mapping of Alternative Isoforms in the Human Proteome
Source: Cell Rep. Author manuscript; Available in PMC 2020 Jan 15. (PMC6961840; doi:10.1016/j.celrep.2019.11.026)
Supplement: 2 [file NIHMS1546469-supplement-2.zip › DF1/PXD000561/Liver/Liver_3_SRSF11_VTAQPDVLEVQAEYITAGPGPSGGPGGGG.pdf]

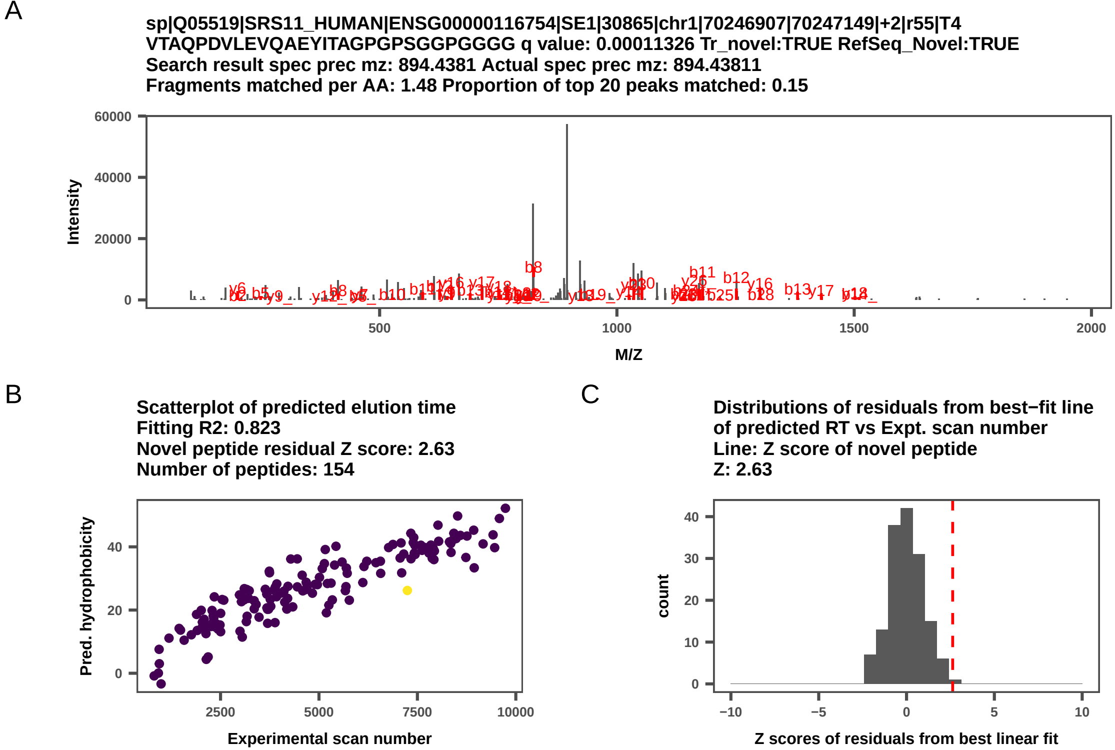
A
sp|Q05519|SRS11_HUMAN|ENSG00000116754|SE1|30865|chr1|70246907|70247149|+2|r55|T4
VTAQPDVLEVQAEYITAGPGPSGGPGGGG q value: 0.00011326 Tr_novel:TRUE RefSeq_Novel:TRUE
Search result spec prec mz: 894.4381 Actual spec prec mz: 894.43811
Fragments matched per AA: 1.48 Proportion of top 20 peaks matched: 0.15
Intensity
60000
40000
20000
0
b8
b11
b12
y16
b13
y17
500
1000
1500
2000
M/Z
B
Scatterplot of predicted elution time
Fitting R2: 0.823
Novel peptide residual Z score: 2.63
Number of peptides: 154
Pred. hydrophobicity
40
20
0
2500
5000
7500
10000
Experimental scan number
C
Distributions of residuals from best−fit line
of predicted RT vs Expt. scan number
Line: Z score of novel peptide
Z: 2.63
count
40
30
20
10
0
−10
−5
0
5
10
Z scores of residuals from best linear fit
