# Supplementary material for: Splice-Junction-Based Mapping of Alternative Isoforms in the Human Proteome
Source: Cell Rep. Author manuscript; Available in PMC 2020 Jan 15. (PMC6961840; doi:10.1016/j.celrep.2019.11.026)
Supplement: 2 [file NIHMS1546469-supplement-2.zip › DF1/PXD000561/Liver/Liver_11_MTX3_LSLGDGWGGGWGLPSVHSESLVVMAYAK.pdf]

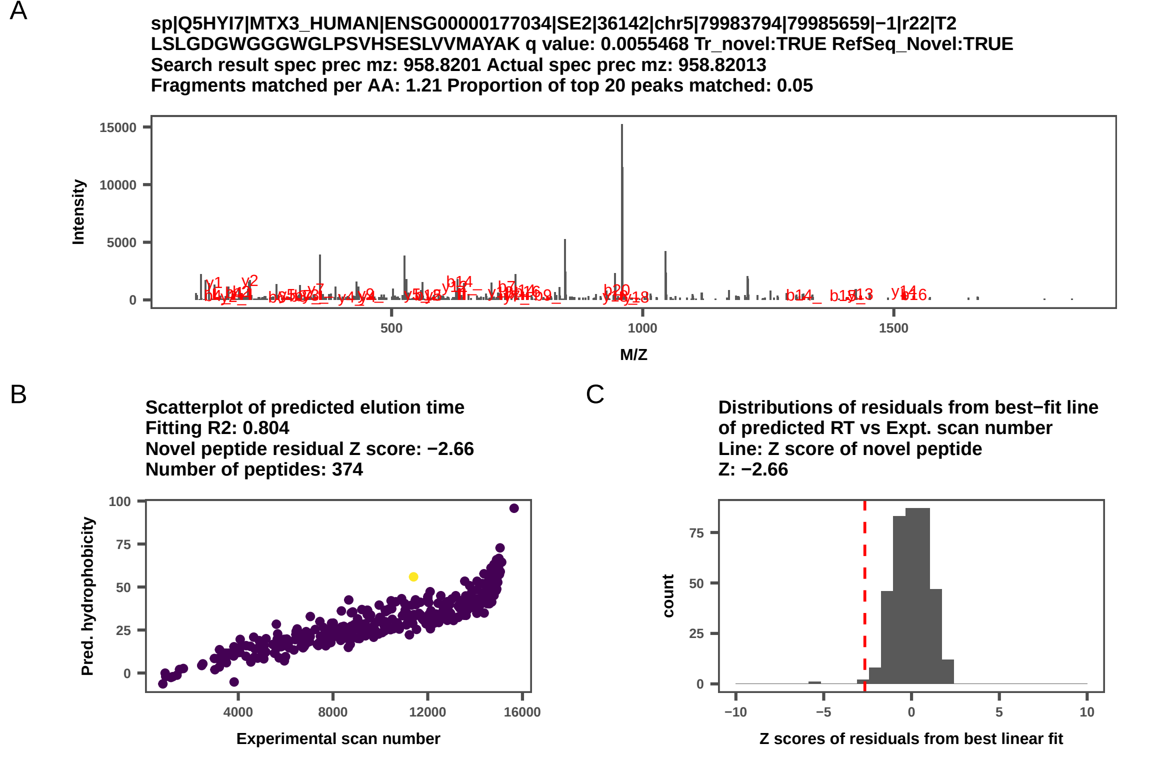
A
sp|Q5HYI7|MTX3_HUMAN|ENSG00000177034|SE2|36142|chr5|79983794|79985659|−1|r22|T2
LSLGDGWGGGWGLPSVHSESLVVMAYAK q value: 0.0055468 Tr_novel:TRUE RefSeq_Novel:TRUE
Search result spec prec mz: 958.8201 Actual spec prec mz: 958.82013
Fragments matched per AA: 1.21 Proportion of top 20 peaks matched: 0.05
Intensity
15000
10000
5000
0
500
1000
1500
M/Z
B
Scatterplot of predicted elution time
Fitting R2: 0.804
Novel peptide residual Z score: −2.66
Number of peptides: 374
Pred. hydrophobicity
100
75
50
25
0
4000
8000
12000
16000
Experimental scan number
C
Distributions of residuals from best−fit line
of predicted RT vs Expt. scan number
Line: Z score of novel peptide
Z: −2.66
count
75
50
25
0
−10
−5
0
5
10
Z scores of residuals from best linear fit
