# Supplementary material for: Splice-Junction-Based Mapping of Alternative Isoforms in the Human Proteome
Source: Cell Rep. Author manuscript; Available in PMC 2020 Jan 15. (PMC6961840; doi:10.1016/j.celrep.2019.11.026)
Supplement: 2 [file NIHMS1546469-supplement-2.zip › DF1/PXD000561/Liver/Liver_12_RFWD2_QLEAQLEQIQK.pdf]

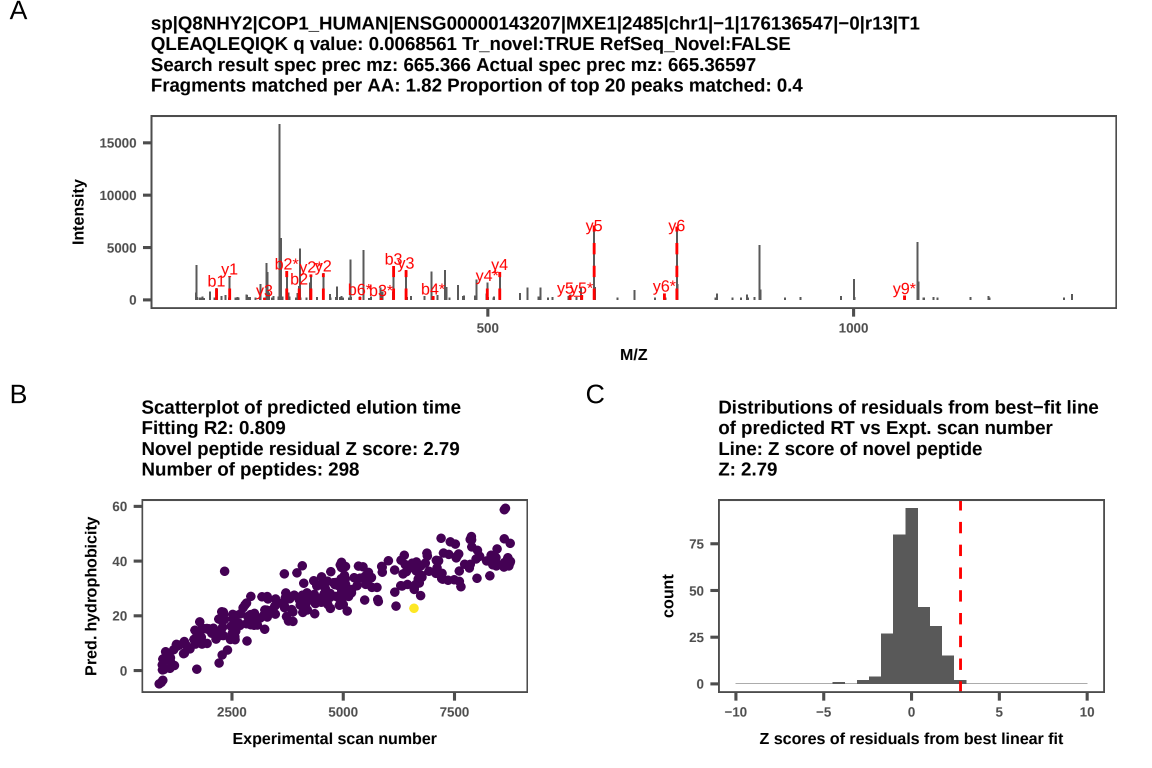
A
sp|Q8NHY2|COP1_HUMAN|ENSG00000143207|MXE1|2485|chr1|−1|176136547|−0|r13|T1
QLEAQLEQIQK q value: 0.0068561 Tr_novel:TRUE RefSeq_Novel:FALSE
Search result spec prec mz: 665.366 Actual spec prec mz: 665.36597
Fragments matched per AA: 1.82 Proportion of top 20 peaks matched: 0.4
Intensity
15000
10000
5000
0
y1
b1
y3
b2*
b2
y2*y2
b6*b3*
b3
y3
b4*
y4*
y4
y5y5*
y5
y6*
y6
y9*
500
1000
M/Z
B
Scatterplot of predicted elution time
Fitting R2: 0.809
Novel peptide residual Z score: 2.79
Number of peptides: 298
Pred. hydrophobicity
60
40
20
0
2500
5000
7500
Experimental scan number
C
Distributions of residuals from best−fit line
of predicted RT vs Expt. scan number
Line: Z score of novel peptide
Z: 2.79
count
75
50
25
0
−10
−5
0
5
10
Z scores of residuals from best linear fit
