# Supplementary material for: Splice-Junction-Based Mapping of Alternative Isoforms in the Human Proteome
Source: Cell Rep. Author manuscript; Available in PMC 2020 Jan 15. (PMC6961840; doi:10.1016/j.celrep.2019.11.026)

A

sp|P02675|FIBB_HUMAN|ENSG00000171564|MXE1|1118|chr4|154566672|154567820|+0|r30104|T1
TPCTVSCNIPVVSGKG q value: 0.00011326 Tr_novel:TRUE RefSeq_Novel:TRUE
Search result spec prec mz: 838.4124 Actual spec prec mz: 838.41235
Fragments matched per AA: 1.56 Proportion of top 20 peaks matched: 0.55

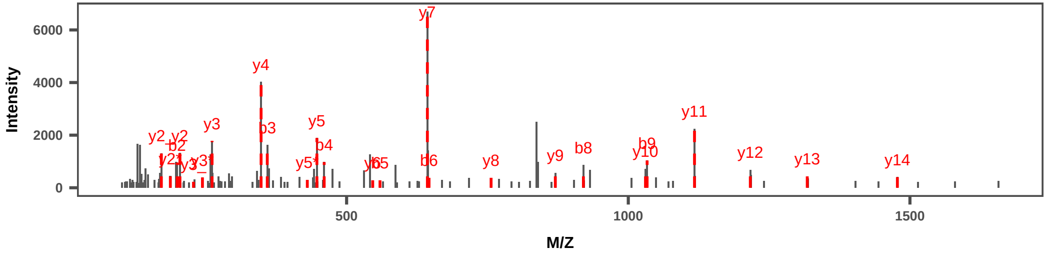

B

Scatterplot of predicted elution time
Fitting R2: 0.825
Novel peptide residual Z score: 0.668
Number of peptides: 28

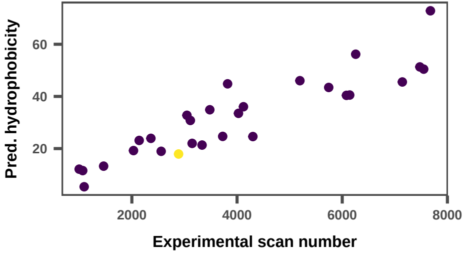

C

Distributions of residuals from best−fit line
of predicted RT vs Expt. scan number
Line: Z score of novel peptide
Z: 0.668

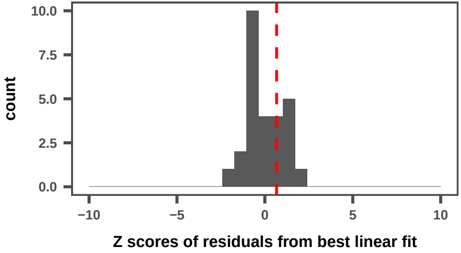

Supplement: 2 [file NIHMS1546469-supplement-2.zip › DF1/PXD000561/Liver/Liver_4_FGB_TPCTVSCNIPVVSGKG.pdf]
