# Supplementary material for: Splice-Junction-Based Mapping of Alternative Isoforms in the Human Proteome
Source: Cell Rep. Author manuscript; Available in PMC 2020 Jan 15. (PMC6961840; doi:10.1016/j.celrep.2019.11.026)
Supplement: 2 [file NIHMS1546469-supplement-2.zip › DF1/PXD000561/Liver/Liver_14_HP_AVGDKLPECEAGV.pdf]

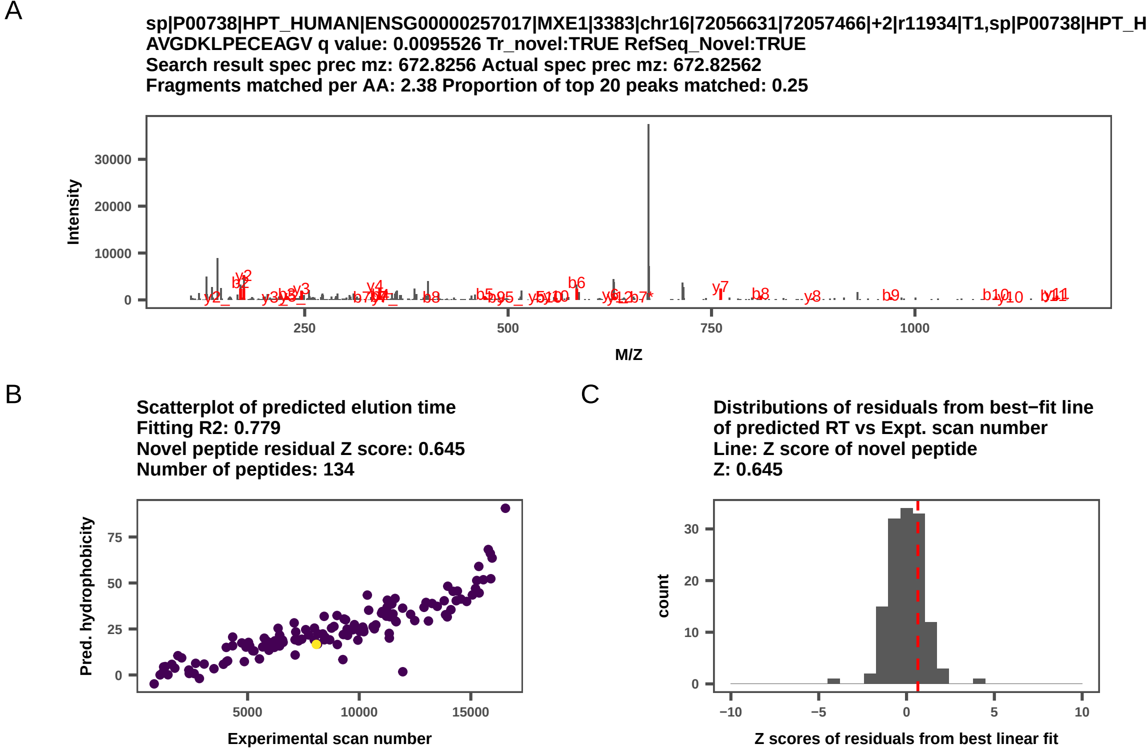
A
sp|P00738|HPT_HUMAN|ENSG00000257017|MXE1|3383|chr16|72056631|72057466|+2|r11934|T1,sp|P00738|HPT_H
AVGDKLPECEAGV q value: 0.0095526 Tr_novel:TRUE RefSeq_Novel:TRUE
Search result spec prec mz: 672.8256 Actual spec prec mz: 672.82562
Fragments matched per AA: 2.38 Proportion of top 20 peaks matched: 0.25
Intensity
30000
20000
10000
0
250
500
750
1000
M/Z
B
Scatterplot of predicted elution time
Fitting R2: 0.779
Novel peptide residual Z score: 0.645
Number of peptides: 134
Pred. hydrophobicity
75
50
25
0
5000
10000
15000
Experimental scan number
C
Distributions of residuals from best−fit line
of predicted RT vs Expt. scan number
Line: Z score of novel peptide
Z: 0.645
count
30
20
10
0
−10
−5
0
5
10
Z scores of residuals from best linear fit
