# Supplementary material for: Splice-Junction-Based Mapping of Alternative Isoforms in the Human Proteome
Source: Cell Rep. Author manuscript; Available in PMC 2020 Jan 15. (PMC6961840; doi:10.1016/j.celrep.2019.11.026)
Supplement: 2 [file NIHMS1546469-supplement-2.zip › DF1/PXD000561/Liver/Liver_2_ALB_LVRPEVDVMCTAFHDNEETFLKN.pdf]

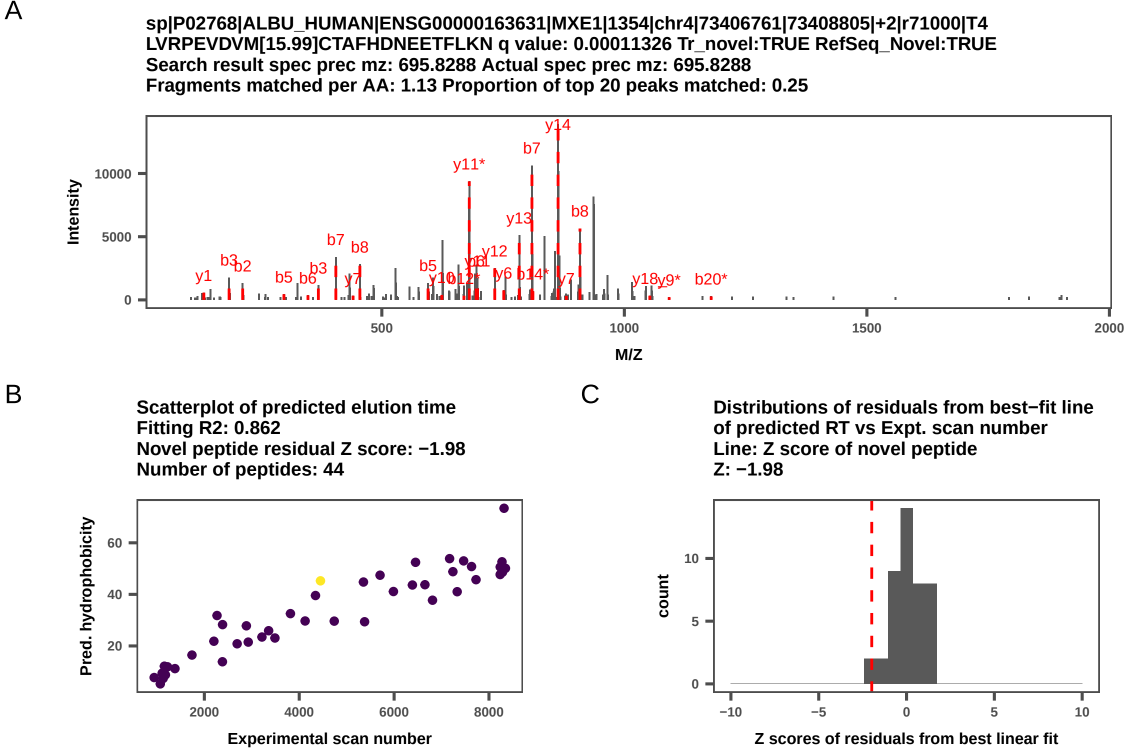
A
sp|P02768|ALBU_HUMAN|ENSG00000163631|MXE1|1354|chr4|73406761|73408805|+2|r71000|T4
LVRPEVDVM[15.99]CTAFHDNEETFLKN q value: 0.00011326 Tr_novel:TRUE RefSeq_Novel:TRUE
Search result spec prec mz: 695.8288 Actual spec prec mz: 695.8288
Fragments matched per AA: 1.13 Proportion of top 20 peaks matched: 0.25
Intensity
10000
5000
0
M/Z
500
1000
1500
2000
y1
b3
b2
b5
b6
b3
b7
b8
y7
b5
y10
b12*
y11*
y12
y6
y13
b14*
b7
y14
y7
b8
y18
y9*
b20*
B
Scatterplot of predicted elution time
Fitting R2: 0.862
Novel peptide residual Z score: −1.98
Number of peptides: 44
Pred. hydrophobicity
60
40
20
2000
4000
6000
8000
Experimental scan number
C
Distributions of residuals from best−fit line
of predicted RT vs Expt. scan number
Line: Z score of novel peptide
Z: −1.98
count
10
5
0
−10
−5
0
5
10
Z scores of residuals from best linear fit
