# Supplementary material for: Splice-Junction-Based Mapping of Alternative Isoforms in the Human Proteome
Source: Cell Rep. Author manuscript; Available in PMC 2020 Jan 15. (PMC6961840; doi:10.1016/j.celrep.2019.11.026)
Supplement: 2 [file NIHMS1546469-supplement-2.zip › DF1/PXD000561/Liver/Liver_5_PSMD2_GFGGSGSQVDSAR.pdf]

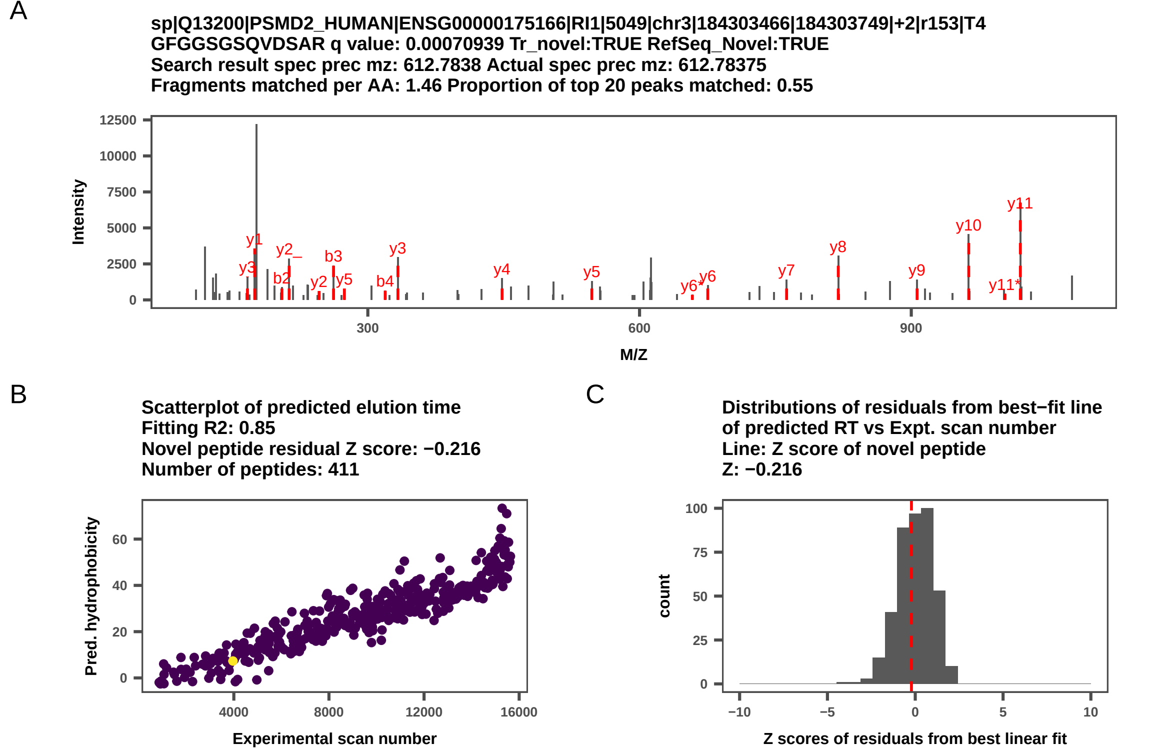
A
sp|Q13200|PSMD2_HUMAN|ENSG00000175166|RI1|5049|chr3|184303466|184303749|+2|r153|T4
GFGGSGSQVDSAR q value: 0.00070939 Tr_novel:TRUE RefSeq_Novel:TRUE
Search result spec prec mz: 612.7838 Actual spec prec mz: 612.78375
Fragments matched per AA: 1.46 Proportion of top 20 peaks matched: 0.55
Intensity
12500
10000
7500
5000
2500
0
y1
y3
y2_
b2
b3
y2
y5
y3
b4
y4
y5
y6*
y6
y7
y8
y9
y10
y11*
y11
300
600
900
M/Z
B
Scatterplot of predicted elution time
Fitting R2: 0.85
Novel peptide residual Z score: −0.216
Number of peptides: 411
Pred. hydrophobicity
60
40
20
0
4000
8000
12000
16000
Experimental scan number
C
Distributions of residuals from best−fit line
of predicted RT vs Expt. scan number
Line: Z score of novel peptide
Z: −0.216
count
100
75
50
25
0
−10
−5
0
5
10
Z scores of residuals from best linear fit
