# Supplementary material for: Splice-Junction-Based Mapping of Alternative Isoforms in the Human Proteome
Source: Cell Rep. Author manuscript; Available in PMC 2020 Jan 15. (PMC6961840; doi:10.1016/j.celrep.2019.11.026)
Supplement: 2 [file NIHMS1546469-supplement-2.zip › DF1/PXD000561/Ovary/Ovary_9_MKS1_WLTSPTHSR.pdf]

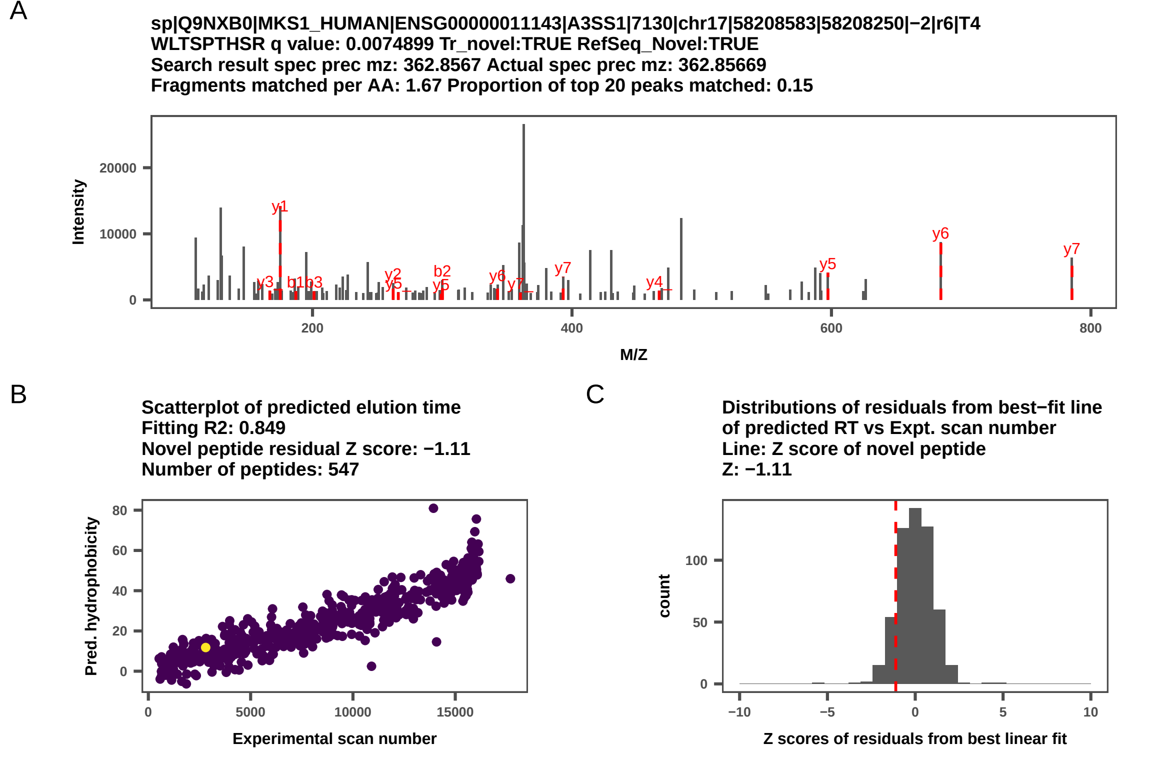
A
sp|Q9NXB0|MKS1_HUMAN|ENSG00000011143|A3SS1|7130|chr17|58208583|58208250|−2|r6|T4
WLTSPTHSR q value: 0.0074899 Tr_novel:TRUE RefSeq_Novel:TRUE
Search result spec prec mz: 362.8567 Actual spec prec mz: 362.85669
Fragments matched per AA: 1.67 Proportion of top 20 peaks matched: 0.15
Intensity
0
10000
20000
y1
y3
b1
b3
y2
y5
b2
y5
y6
y7
y7
y4
y5
y6
y7
200
400
600
800
M/Z
B
Scatterplot of predicted elution time
Fitting R2: 0.849
Novel peptide residual Z score: −1.11
Number of peptides: 547
Pred. hydrophobicity
0
20
40
60
80
0
5000
10000
15000
Experimental scan number
C
Distributions of residuals from best−fit line
of predicted RT vs Expt. scan number
Line: Z score of novel peptide
Z: −1.11
count
0
50
100
−10
−5
0
5
10
Z scores of residuals from best linear fit
