# Supplementary material for: Splice-Junction-Based Mapping of Alternative Isoforms in the Human Proteome
Source: Cell Rep. Author manuscript; Available in PMC 2020 Jan 15. (PMC6961840; doi:10.1016/j.celrep.2019.11.026)
Supplement: 2 [file NIHMS1546469-supplement-2.zip › DF1/PXD000561/Ovary/Ovary_2_SRRM1_KVELSESEDKGGK.pdf]

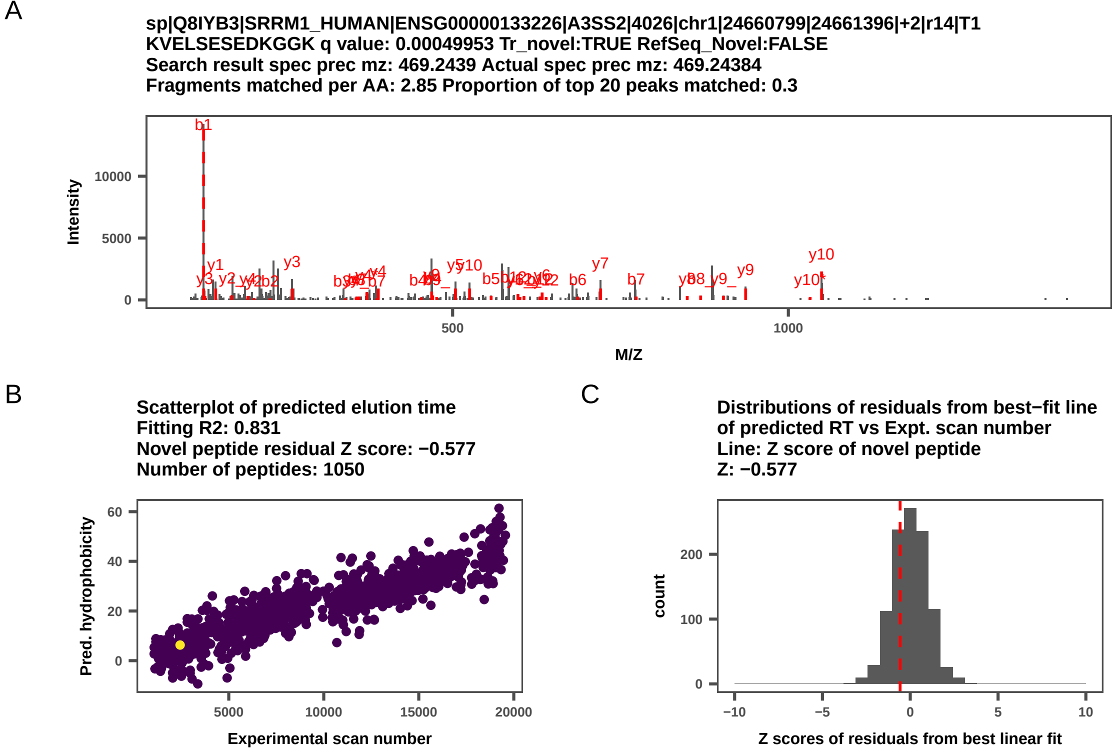
A
sp|Q8IYB3|SRRM1_HUMAN|ENSG00000133226|A3SS2|4026|chr1|24660799|24661396|+2|r14|T1
KVELSESEDKGGK q value: 0.00049953 Tr_novel:TRUE RefSeq_Novel:FALSE
Search result spec prec mz: 469.2439 Actual spec prec mz: 469.24384
Fragments matched per AA: 2.85 Proportion of top 20 peaks matched: 0.3
Intensity
10000
5000
0
b1
y1
y3
y7
b7
b6
y9
y10
y10*
500
1000
M/Z
B
Scatterplot of predicted elution time
Fitting R2: 0.831
Novel peptide residual Z score: −0.577
Number of peptides: 1050
Pred. hydrophobicity
60
40
20
0
5000
10000
15000
20000
Experimental scan number
C
Distributions of residuals from best−fit line
of predicted RT vs Expt. scan number
Line: Z score of novel peptide
Z: −0.577
count
200
100
0
−10
−5
0
5
10
Z scores of residuals from best linear fit
