# Supplementary material for: Splice-Junction-Based Mapping of Alternative Isoforms in the Human Proteome
Source: Cell Rep. Author manuscript; Available in PMC 2020 Jan 15. (PMC6961840; doi:10.1016/j.celrep.2019.11.026)
Supplement: 2 [file NIHMS1546469-supplement-2.zip › DF1/PXD000561/Ovary/Ovary_1_GRINA_HLLHASGDNYPPPNPGYPGGPQPPMPPYAQPPYPGAPYPQPPFQP.pdf]

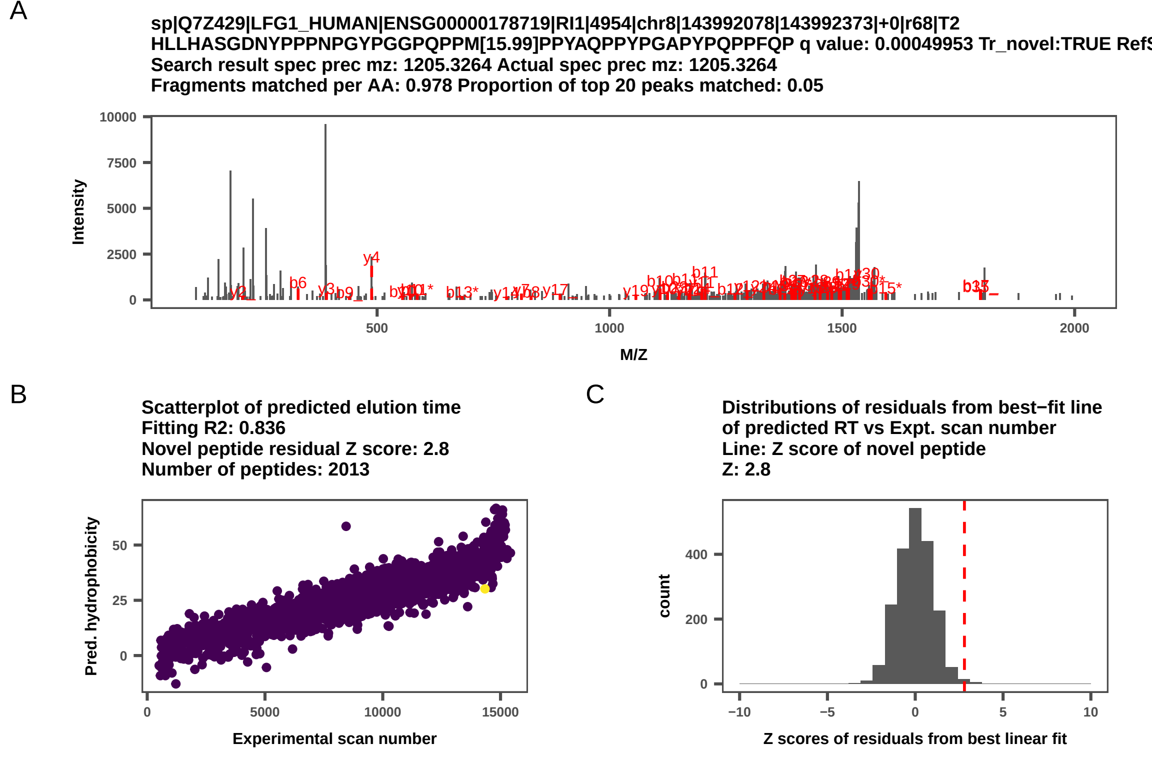
A
sp|Q7Z429|LFG1_HUMAN|ENSG00000178719|RI1|4954|chr8|143992078|143992373|+0|r68|T2
HLLHASGDNYPPPNPGYPGGPQPPM[15.99]PPYAQPPYPGAPYPQPPFQP q value: 0.00049953 Tr_novel:TRUE RefS
Search result spec prec mz: 1205.3264 Actual spec prec mz: 1205.3264
Fragments matched per AA: 0.978 Proportion of top 20 peaks matched: 0.05
Intensity
10000
7500
5000
2500
0
y4
b6
y2
y3
b9
b11
M/Z
500
1000
1500
2000
B
Scatterplot of predicted elution time
Fitting R2: 0.836
Novel peptide residual Z score: 2.8
Number of peptides: 2013
Pred. hydrophobicity
50
25
0
Experimental scan number
0
5000
10000
15000
C
Distributions of residuals from best−fit line
of predicted RT vs Expt. scan number
Line: Z score of novel peptide
Z: 2.8
count
400
200
0
Z scores of residuals from best linear fit
−10
−5
0
5
10
