# Supplementary material for: Splice-Junction-Based Mapping of Alternative Isoforms in the Human Proteome
Source: Cell Rep. Author manuscript; Available in PMC 2020 Jan 15. (PMC6961840; doi:10.1016/j.celrep.2019.11.026)
Supplement: 2 [file NIHMS1546469-supplement-2.zip › DF1/PXD000561/Ovary/Ovary_10_RBBP4_GPDAVEERVINEEYK.pdf]

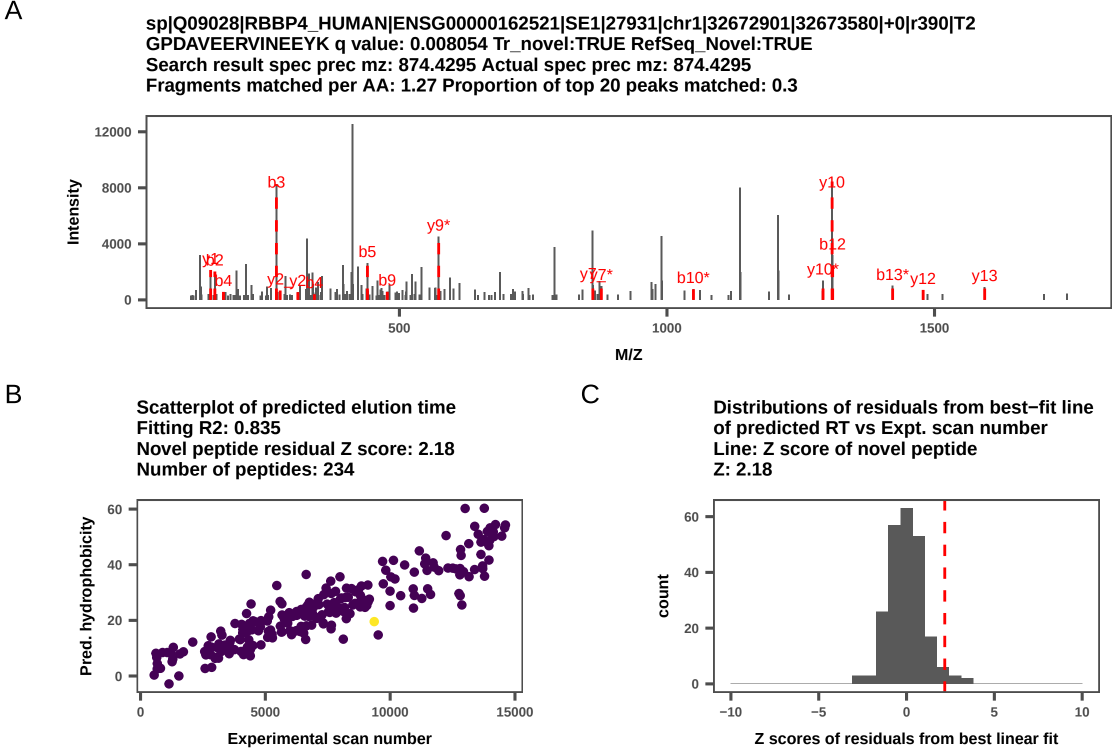
A
sp|Q09028|RBBP4_HUMAN|ENSG00000162521|SE1|27931|chr1|32672901|32673580|+0|r390|T2
GPDAVEERVINEEYK q value: 0.008054 Tr_novel:TRUE RefSeq_Novel:TRUE
Search result spec prec mz: 874.4295 Actual spec prec mz: 874.4295
Fragments matched per AA: 1.27 Proportion of top 20 peaks matched: 0.3
Intensity
12000
8000
4000
0
b3
y9*
b5
b9
b4
y2
b10*
y10
b12
y10*
b13*
y12
y13
500
1000
1500
M/Z
B
Scatterplot of predicted elution time
Fitting R2: 0.835
Novel peptide residual Z score: 2.18
Number of peptides: 234
Pred. hydrophobicity
60
40
20
0
0
5000
10000
15000
Experimental scan number
C
Distributions of residuals from best−fit line
of predicted RT vs Expt. scan number
Line: Z score of novel peptide
Z: 2.18
count
60
40
20
0
−10
−5
0
5
10
Z scores of residuals from best linear fit
