# Supplementary material for: Splice-Junction-Based Mapping of Alternative Isoforms in the Human Proteome
Source: Cell Rep. Author manuscript; Available in PMC 2020 Jan 15. (PMC6961840; doi:10.1016/j.celrep.2019.11.026)
Supplement: 2 [file NIHMS1546469-supplement-2.zip › DF1/PXD000561/Ovary/Ovary_4_ARFIP2_AADATMEIPIHGNGEAR.pdf]

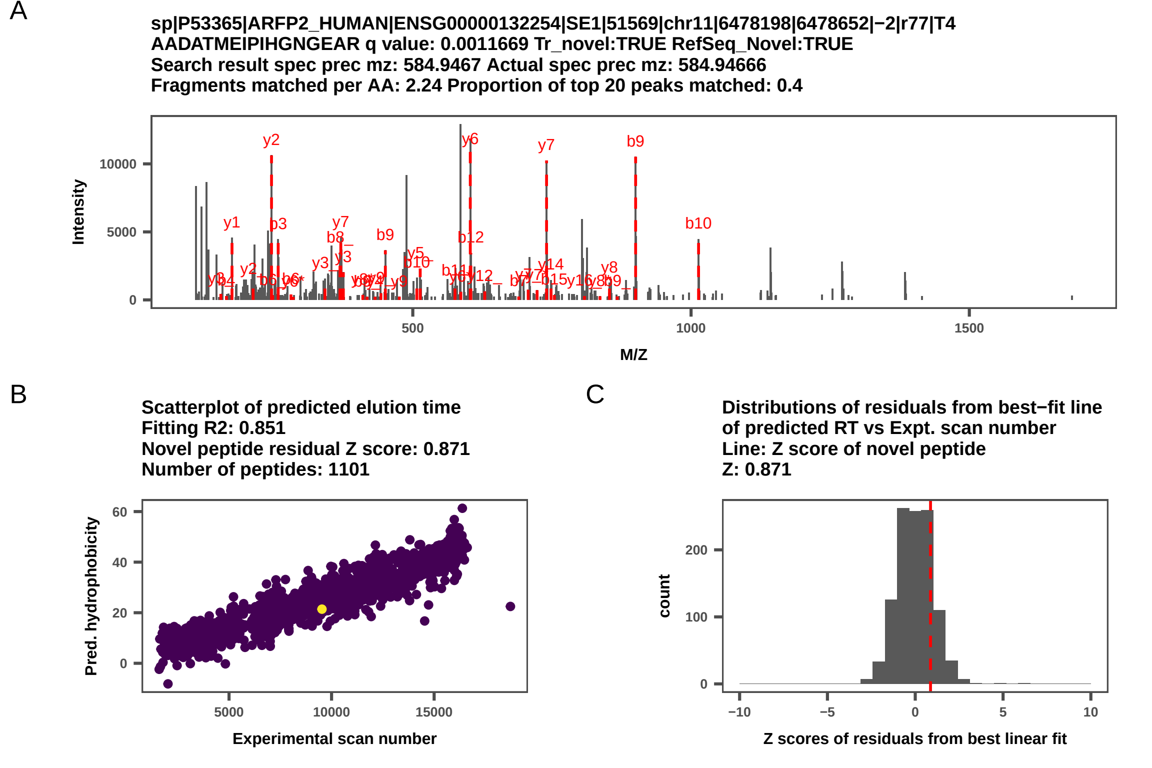
A
sp|P53365|ARFP2_HUMAN|ENSG00000132254|SE1|51569|chr11|6478198|6478652|−2|r77|T4
AADATMEIPIHGNGEAR q value: 0.0011669 Tr_novel:TRUE RefSeq_Novel:TRUE
Search result spec prec mz: 584.9467 Actual spec prec mz: 584.94666
Fragments matched per AA: 2.24 Proportion of top 20 peaks matched: 0.4
Intensity
10000
5000
0
y2
y6
y7
b9
y1
b3
y7
b8
b9
b12
b10
y3
y3
y5
b10
y8
y14
500
1000
1500
M/Z
B
Scatterplot of predicted elution time
Fitting R2: 0.851
Novel peptide residual Z score: 0.871
Number of peptides: 1101
Pred. hydrophobicity
60
40
20
0
5000
10000
15000
Experimental scan number
C
Distributions of residuals from best−fit line
of predicted RT vs Expt. scan number
Line: Z score of novel peptide
Z: 0.871
count
200
100
0
−10
−5
0
5
10
Z scores of residuals from best linear fit
