# Supplementary material for: Splice-Junction-Based Mapping of Alternative Isoforms in the Human Proteome
Source: Cell Rep. Author manuscript; Available in PMC 2020 Jan 15. (PMC6961840; doi:10.1016/j.celrep.2019.11.026)
Supplement: 2 [file NIHMS1546469-supplement-2.zip › DF1/PXD000561/Ovary/Ovary_3_PCBP2_YSTGSDSASFPHTTPSMCLNPDLEGPPLELTK.pdf]

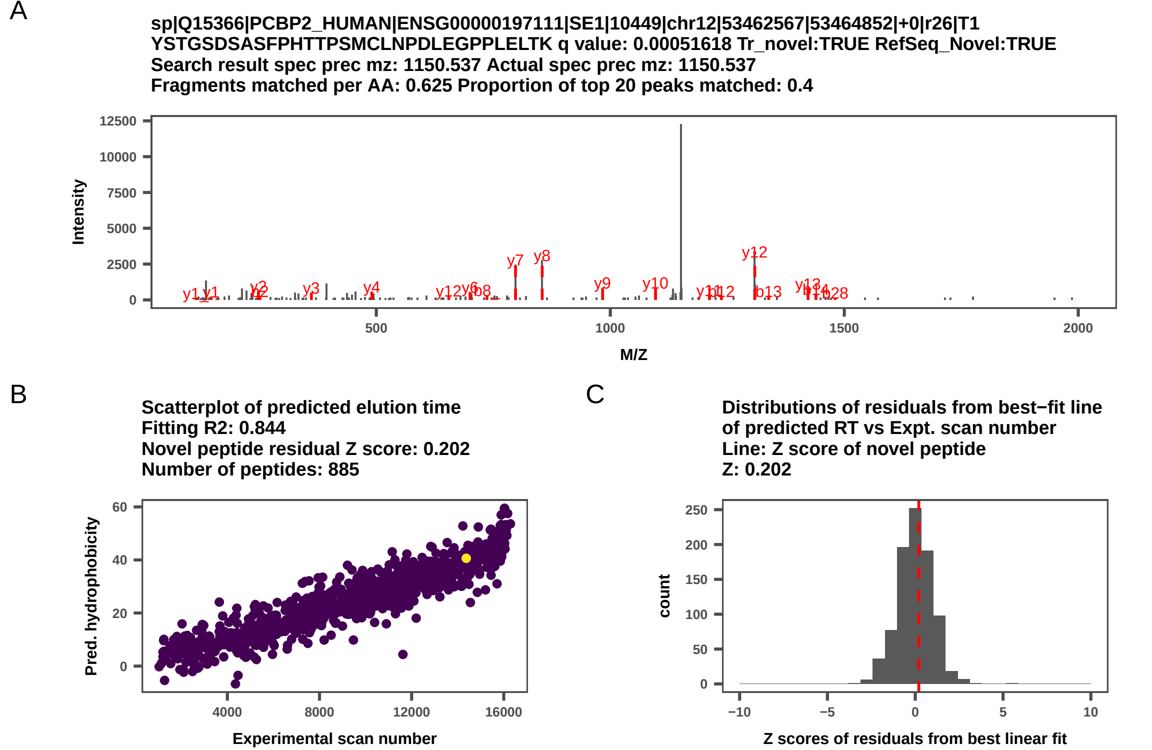
A
sp|Q15366|PCBP2_HUMAN|ENSG00000197111|SE1|10449|chr12|53462567|53464852|+0|r26|T1
YSTGSDSASFPHTTPSMCLNPDLEGPPLELTK q value: 0.00051618 Tr_novel:TRUE RefSeq_Novel:TRUE
Search result spec prec mz: 1150.537 Actual spec prec mz: 1150.537
Fragments matched per AA: 0.625 Proportion of top 20 peaks matched: 0.4
Intensity
M/Z
y7
y8
y9
y10
y12
b13
y4
y3
y2
B
Scatterplot of predicted elution time
Fitting R2: 0.844
Novel peptide residual Z score: 0.202
Number of peptides: 885
Pred. hydrophobicity
Experimental scan number
C
Distributions of residuals from best−fit line
of predicted RT vs Expt. scan number
Line: Z score of novel peptide
Z: 0.202
count
Z scores of residuals from best linear fit
