# Supplementary material for: Splice-Junction-Based Mapping of Alternative Isoforms in the Human Proteome
Source: Cell Rep. Author manuscript; Available in PMC 2020 Jan 15. (PMC6961840; doi:10.1016/j.celrep.2019.11.026)
Supplement: 2 [file NIHMS1546469-supplement-2.zip › DF1/PXD000561/Ovary/Ovary_6_DDX24_VQHVIHYQVGASENLPKQSSCGK.pdf]

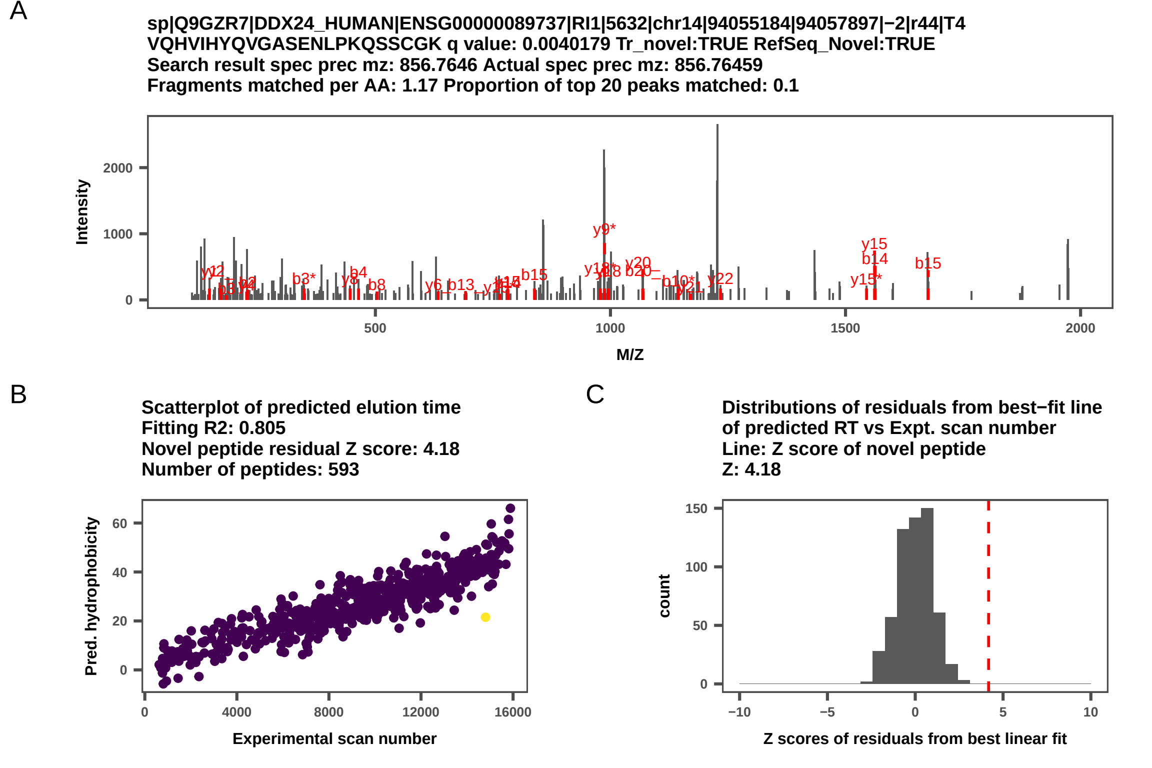
A
sp|Q9GZR7|DDX24_HUMAN|ENSG00000089737|RI1|5632|chr14|94055184|94057897|−2|r44|T4
VQHVIHYQVGASENLPKQSSCGK q value: 0.0040179 Tr_novel:TRUE RefSeq_Novel:TRUE
Search result spec prec mz: 856.7646 Actual spec prec mz: 856.76459
Fragments matched per AA: 1.17 Proportion of top 20 peaks matched: 0.1
Intensity
0
1000
2000
M/Z
500
1000
1500
2000
y9*
y15
b14
b15
y15*
y22
b3*
b4
b8
y6
b13
b15
y20
B
Scatterplot of predicted elution time
Fitting R2: 0.805
Novel peptide residual Z score: 4.18
Number of peptides: 593
Pred. hydrophobicity
0
20
40
60
Experimental scan number
0
4000
8000
12000
16000
C
Distributions of residuals from best−fit line
of predicted RT vs Expt. scan number
Line: Z score of novel peptide
Z: 4.18
count
0
50
100
150
Z scores of residuals from best linear fit
−10
−5
0
5
10
