# Supplementary material for: Splice-Junction-Based Mapping of Alternative Isoforms in the Human Proteome
Source: Cell Rep. Author manuscript; Available in PMC 2020 Jan 15. (PMC6961840; doi:10.1016/j.celrep.2019.11.026)
Supplement: 2 [file NIHMS1546469-supplement-2.zip › DF1/PXD000561/Ovary/Ovary_7_SLC20A1_MPLGLWLLYIWFMTQEMFLQK.pdf]

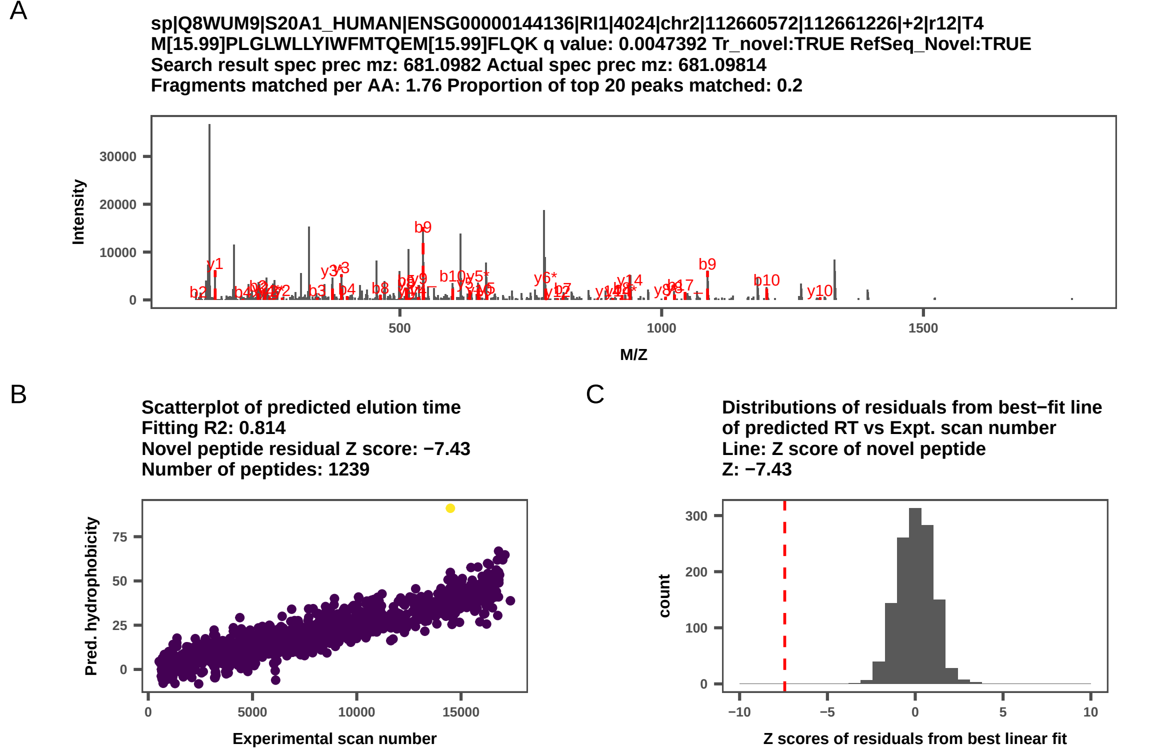
A
sp|Q8WUM9|S20A1_HUMAN|ENSG00000144136|RI1|4024|chr2|112660572|112661226|+2|r12|T4
M[15.99]PLGLWLLYIWFMTQEM[15.99]FLQK q value: 0.0047392 Tr_novel:TRUE RefSeq_Novel:TRUE
Search result spec prec mz: 681.0982 Actual spec prec mz: 681.09814
Fragments matched per AA: 1.76 Proportion of top 20 peaks matched: 0.2
Intensity
30000
20000
10000
0
500
1000
1500
M/Z
b9
y1
y3
b10
y5*
y6*
y14
b9
b10
y10
B
Scatterplot of predicted elution time
Fitting R2: 0.814
Novel peptide residual Z score: −7.43
Number of peptides: 1239
Pred. hydrophobicity
75
50
25
0
5000
10000
15000
Experimental scan number
C
Distributions of residuals from best−fit line
of predicted RT vs Expt. scan number
Line: Z score of novel peptide
Z: −7.43
count
300
200
100
0
−10
−5
0
5
10
Z scores of residuals from best linear fit
