# Supplementary material for: Splice-Junction-Based Mapping of Alternative Isoforms in the Human Proteome
Source: Cell Rep. Author manuscript; Available in PMC 2020 Jan 15. (PMC6961840; doi:10.1016/j.celrep.2019.11.026)
Supplement: 2 [file NIHMS1546469-supplement-2.zip › DF1/PXD000561/Ovary/Ovary_8_ACBD4_FSVIPWSSWSLSPDCQK.pdf]

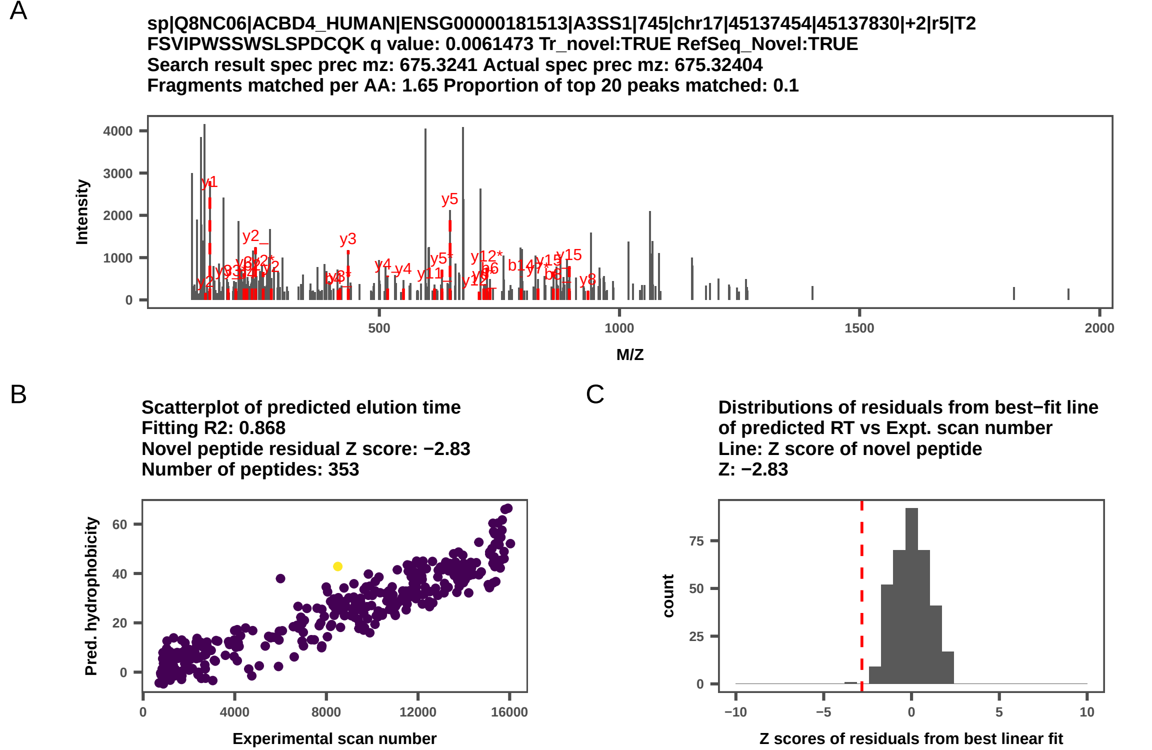
A
sp|Q8NC06|ACBD4_HUMAN|ENSG00000181513|A3SS1|745|chr17|45137454|45137830|+2|r5|T2
FSVIPWSSSWSLSPDCQK q value: 0.0061473 Tr_novel:TRUE RefSeq_Novel:TRUE
Search result spec prec mz: 675.3241 Actual spec prec mz: 675.32404
Fragments matched per AA: 1.65 Proportion of top 20 peaks matched: 0.1
Intensity
4000
3000
2000
1000
0
y1
y2_
y3
y5
y5*
y4
y11
y12*
b14
y15
y8
M/Z
500
1000
1500
2000
B
Scatterplot of predicted elution time
Fitting R2: 0.868
Novel peptide residual Z score: −2.83
Number of peptides: 353
Pred. hydrophobicity
60
40
20
0
0
4000
8000
12000
16000
Experimental scan number
C
Distributions of residuals from best−fit line
of predicted RT vs Expt. scan number
Line: Z score of novel peptide
Z: −2.83
count
75
50
25
0
−10
−5
0
5
10
Z scores of residuals from best linear fit
