# Supplementary material for: Splice-Junction-Based Mapping of Alternative Isoforms in the Human Proteome
Source: Cell Rep. Author manuscript; Available in PMC 2020 Jan 15. (PMC6961840; doi:10.1016/j.celrep.2019.11.026)
Supplement: 2 [file NIHMS1546469-supplement-2.zip › DF1/PXD009021/Liver/Liver_2_ARHGEF12_LQDGAVVTPSRPLGDTLTVSEAETDPGDVLGR.pdf]

A

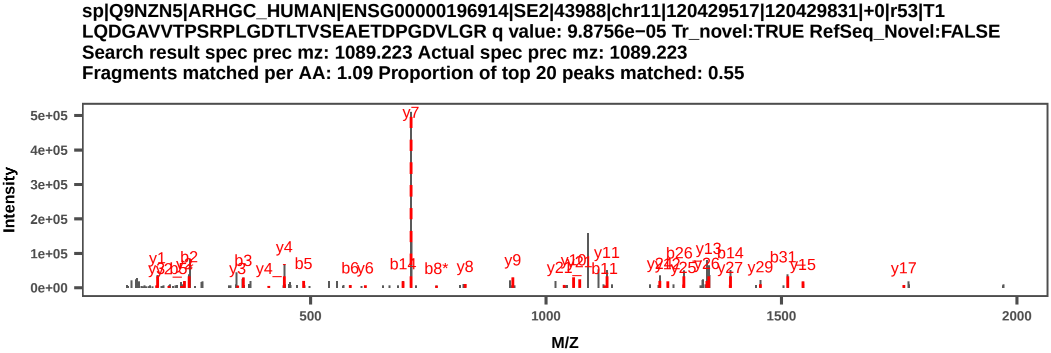
sp|Q9NZN5|ARHGC_HUMAN|ENSG00000196914|SE2|43988|chr11|120429517|120429831|+0|r53|T1
LQDGAVVTPSRPLGDTLTVSEAETDPGDVLGR q value: 9.8756e−05 Tr_novel:TRUE RefSeq_Novel:FALSE
Search result spec prec mz: 1089.223 Actual spec prec mz: 1089.223
Fragments matched per AA: 1.09 Proportion of top 20 peaks matched: 0.55
Intensity
5e+05
4e+05
3e+05
2e+05
1e+05
0e+00
M/Z
500
1000
1500
2000
y7
y1
b2
b3
y4
b5
b6
y6
b14
b8*
y8
y9
y11
b11
b26
y13
b14
y27
y29
b31
y15
y17

B

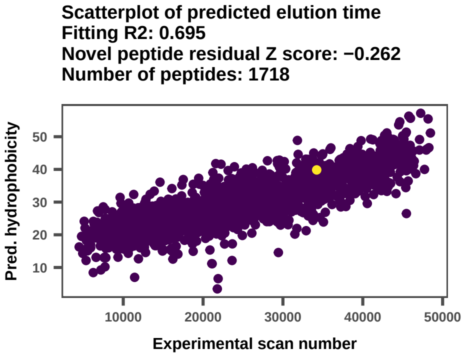
Scatterplot of predicted elution time
Fitting R2: 0.695
Novel peptide residual Z score: −0.262
Number of peptides: 1718
Pred. hydrophobicity
10
20
30
40
50
Experimental scan number
10000
20000
30000
40000
50000

C

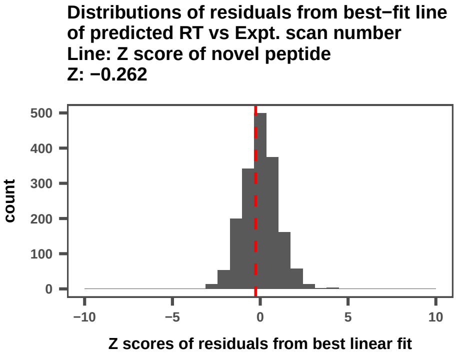
Distributions of residuals from best−fit line
of predicted RT vs Expt. scan number
Line: Z score of novel peptide
Z: −0.262
count
0
100
200
300
400
500
Z scores of residuals from best linear fit
−10
−5
0
5
10
