# Supplementary material for: Splice-Junction-Based Mapping of Alternative Isoforms in the Human Proteome
Source: Cell Rep. Author manuscript; Available in PMC 2020 Jan 15. (PMC6961840; doi:10.1016/j.celrep.2019.11.026)
Supplement: 2 [file NIHMS1546469-supplement-2.zip › DF1/PXD009021/Liver/Liver_14_PMPCA_AVHAHPWSWNEPR.pdf]

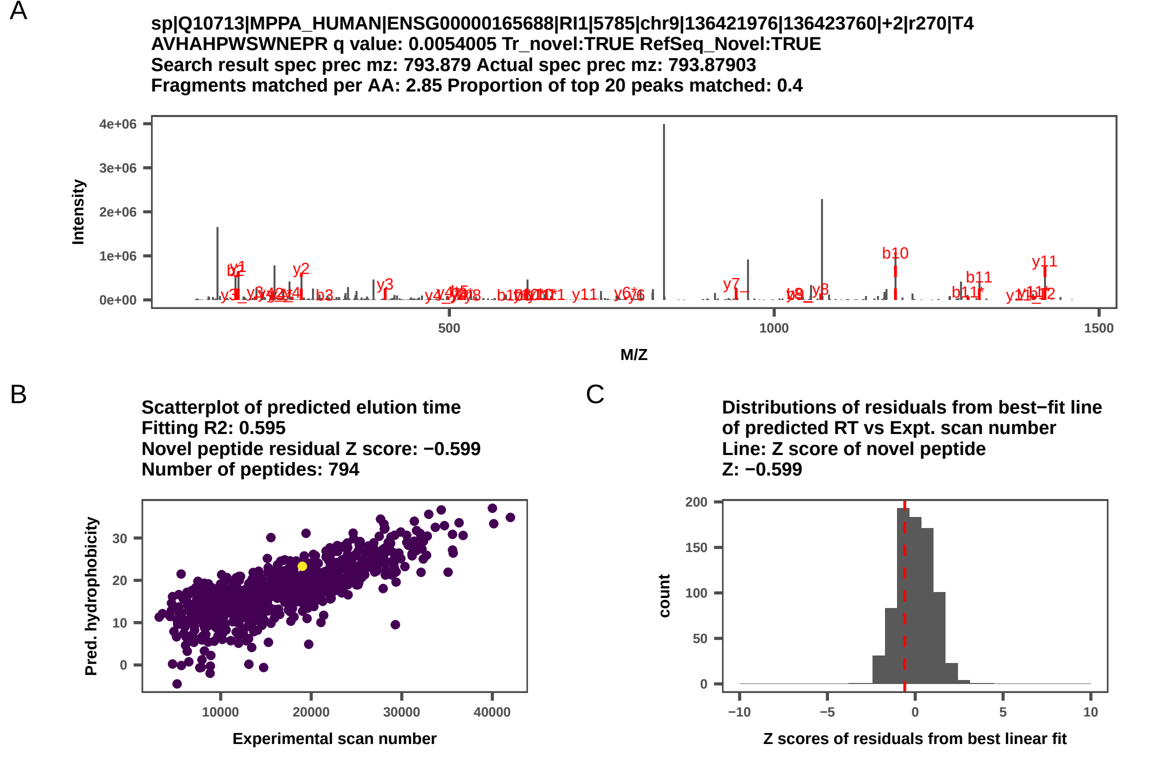
A
sp|Q10713|MPPA_HUMAN|ENSG00000165688|RI1|5785|chr9|136421976|136423760|+2|r270|T4
AVHAHPWSWNEPR q value: 0.0054005 Tr_novel:TRUE RefSeq_Novel:TRUE
Search result spec prec mz: 793.879 Actual spec prec mz: 793.87903
Fragments matched per AA: 2.85 Proportion of top 20 peaks matched: 0.4
Intensity
4e+06
3e+06
2e+06
1e+06
0e+00
y1
y2
b3
y3
y7
y8
b10
b11
y11
500
1000
1500
M/Z
B
Scatterplot of predicted elution time
Fitting R2: 0.595
Novel peptide residual Z score: −0.599
Number of peptides: 794
Pred. hydrophobicity
30
20
10
0
10000
20000
30000
40000
Experimental scan number
C
Distributions of residuals from best−fit line
of predicted RT vs Expt. scan number
Line: Z score of novel peptide
Z: −0.599
count
200
150
100
50
0
−10
−5
0
5
10
Z scores of residuals from best linear fit
