# Supplementary material for: Splice-Junction-Based Mapping of Alternative Isoforms in the Human Proteome
Source: Cell Rep. Author manuscript; Available in PMC 2020 Jan 15. (PMC6961840; doi:10.1016/j.celrep.2019.11.026)
Supplement: 2 [file NIHMS1546469-supplement-2.zip › DF1/PXD009021/Liver/Liver_15_C3_GNLDEDIIAEENIVSR.pdf]

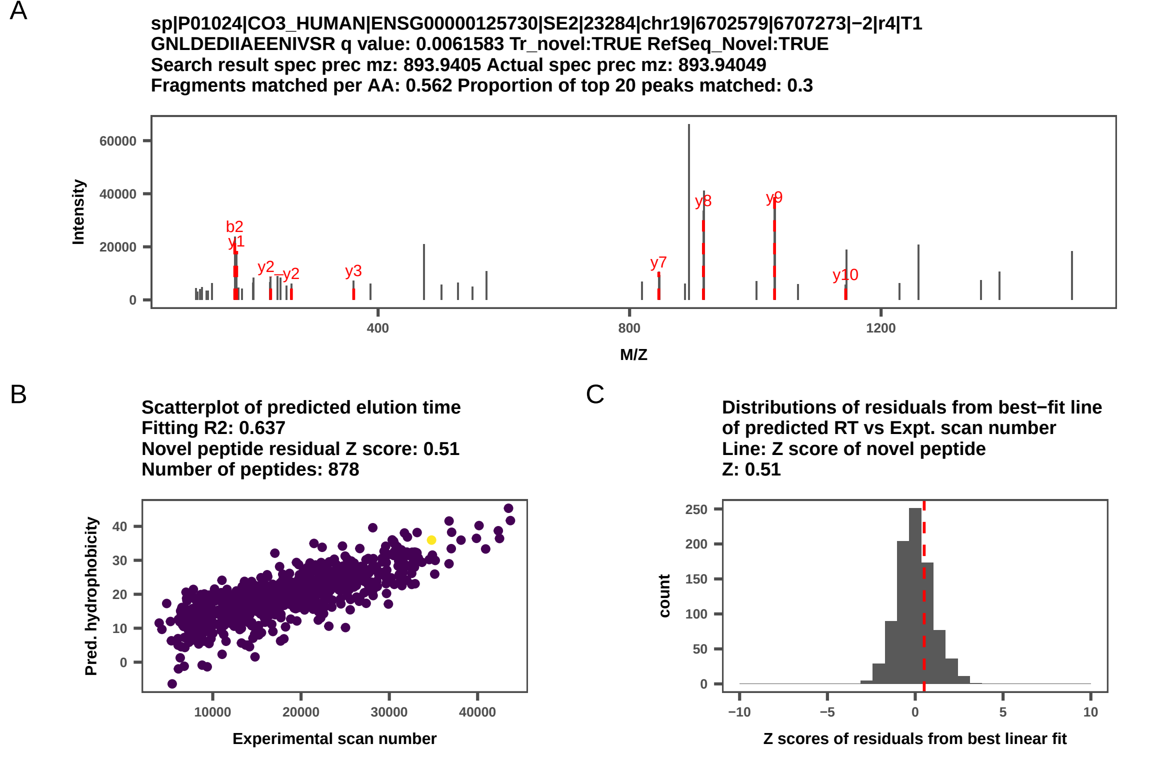
A
sp|P01024|CO3_HUMAN|ENSG00000125730|SE2|23284|chr19|6702579|6707273|−2|r4|T1
GNLDEDIIAEENIVSR q value: 0.0061583 Tr_novel:TRUE RefSeq_Novel:TRUE
Search result spec prec mz: 893.9405 Actual spec prec mz: 893.94049
Fragments matched per AA: 0.562 Proportion of top 20 peaks matched: 0.3
Intensity
60000
40000
20000
0
b2
y1
y2
y2
y3
y7
y8
y9
y10
400
800
1200
M/Z
B
Scatterplot of predicted elution time
Fitting R2: 0.637
Novel peptide residual Z score: 0.51
Number of peptides: 878
Pred. hydrophobicity
40
30
20
10
0
10000
20000
30000
40000
Experimental scan number
C
Distributions of residuals from best−fit line
of predicted RT vs Expt. scan number
Line: Z score of novel peptide
Z: 0.51
count
250
200
150
100
50
0
−10
−5
0
5
10
Z scores of residuals from best linear fit
