# Supplementary material for: Splice-Junction-Based Mapping of Alternative Isoforms in the Human Proteome
Source: Cell Rep. Author manuscript; Available in PMC 2020 Jan 15. (PMC6961840; doi:10.1016/j.celrep.2019.11.026)
Supplement: 2 [file NIHMS1546469-supplement-2.zip › DF1/PXD009021/Liver/Liver_7_FGG_QVRPEHPAETEYDSLYPEDDI.pdf]

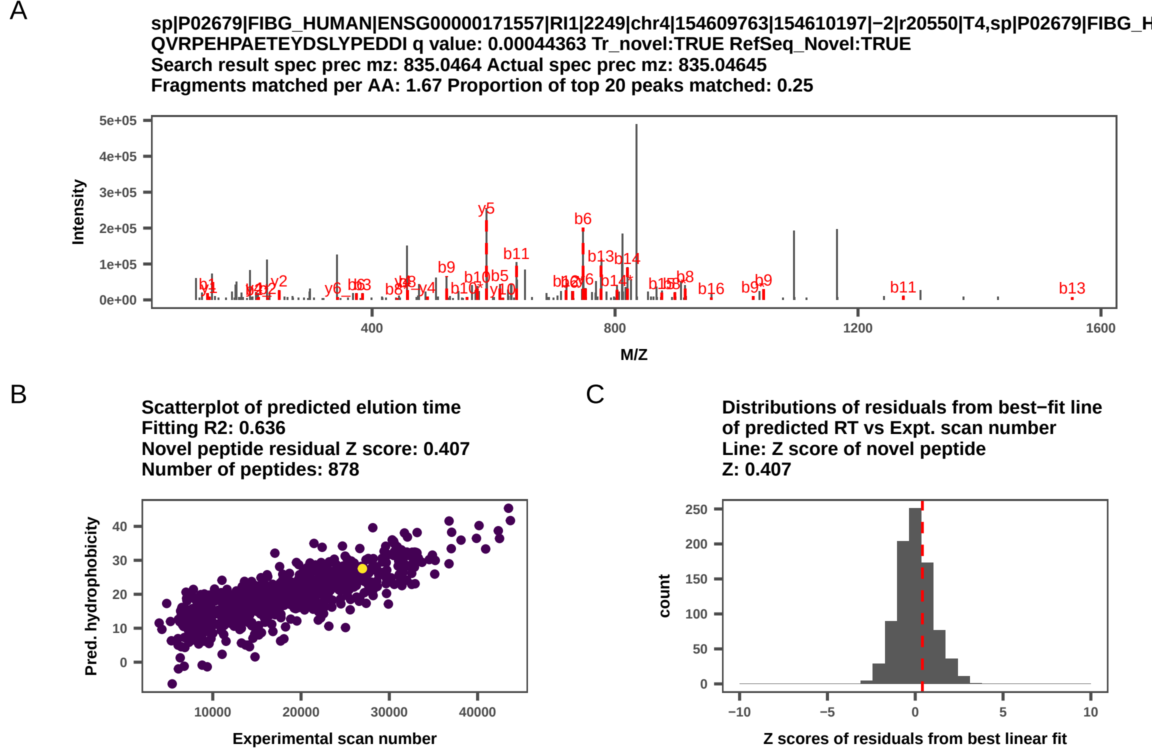
A
sp|P02679|FIBG_HUMAN|ENSG00000171557|RI1|2249|chr4|154609763|154610197|−2|r20550|T4,sp|P02679|FIBG_H
QVRPEHPAETEYDSLYPEDDI q value: 0.00044363 Tr_novel:TRUE RefSeq_Novel:TRUE
Search result spec prec mz: 835.0464 Actual spec prec mz: 835.04645
Fragments matched per AA: 1.67 Proportion of top 20 peaks matched: 0.25
Intensity
5e+05
4e+05
3e+05
2e+05
1e+05
0e+00
y5
b6
b11
b13
b14
b9
M/Z
400
800
1200
1600
B
Scatterplot of predicted elution time
Fitting R2: 0.636
Novel peptide residual Z score: 0.407
Number of peptides: 878
Pred. hydrophobicity
40
30
20
10
0
10000
20000
30000
40000
Experimental scan number
C
Distributions of residuals from best−fit line
of predicted RT vs Expt. scan number
Line: Z score of novel peptide
Z: 0.407
count
250
200
150
100
50
0
−10
−5
0
5
10
Z scores of residuals from best linear fit
