# Supplementary material for: Splice-Junction-Based Mapping of Alternative Isoforms in the Human Proteome
Source: Cell Rep. Author manuscript; Available in PMC 2020 Jan 15. (PMC6961840; doi:10.1016/j.celrep.2019.11.026)
Supplement: 2 [file NIHMS1546469-supplement-2.zip › DF1/PXD009021/Liver/Liver_1_CLTCL1_LQEHFQLQNLGINPANIGFSTLTMESDK.pdf]

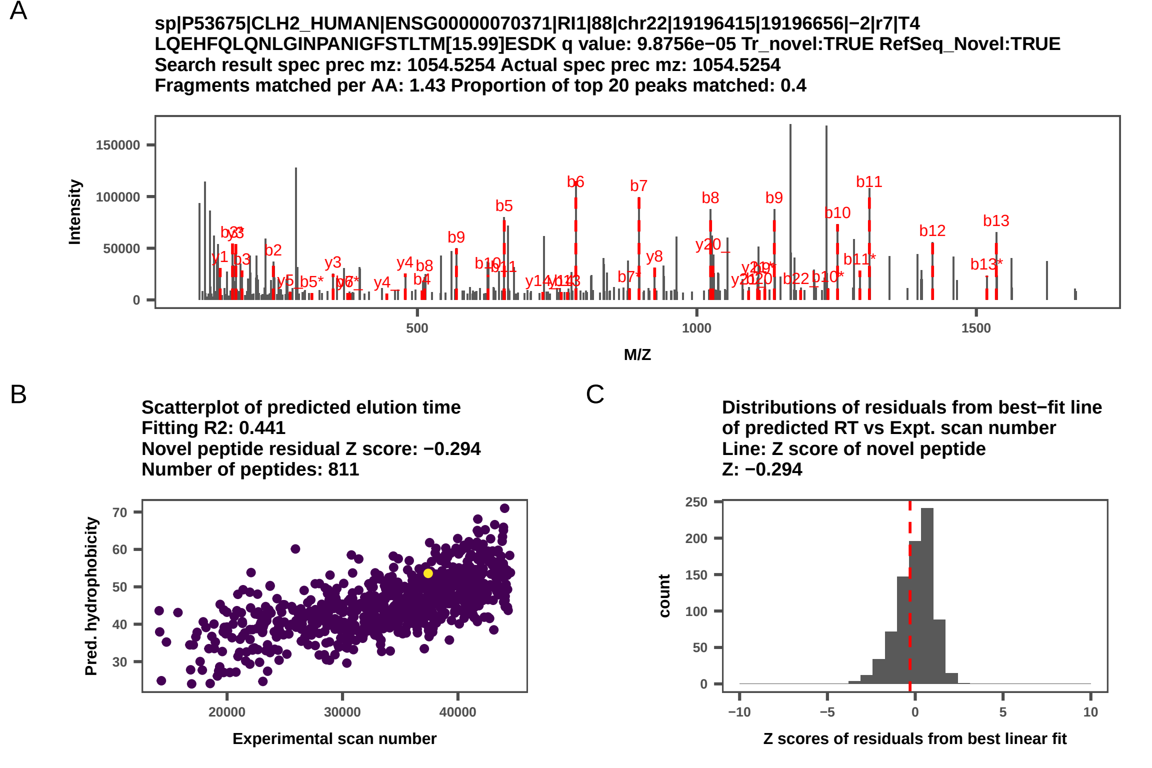
A
sp|P53675|CLH2_HUMAN|ENSG00000070371|RI1|88|chr22|19196415|19196656|−2|r7|T4
LQEHFQLQNLGINPANIGFSTLTM[15.99]ESDK q value: 9.8756e−05 Tr_novel:TRUE RefSeq_Novel:TRUE
Search result spec prec mz: 1054.5254 Actual spec prec mz: 1054.5254
Fragments matched per AA: 1.43 Proportion of top 20 peaks matched: 0.4
Intensity
150000
100000
50000
0
M/Z
500
1000
1500
b2
b3
b4
b5
b6
b7
b8
b9
b10
b11
b12
b13
b13*
b11*
b10*
y1
y3
y4
y8
B
Scatterplot of predicted elution time
Fitting R2: 0.441
Novel peptide residual Z score: −0.294
Number of peptides: 811
Pred. hydrophobicity
70
60
50
40
30
20000
30000
40000
Experimental scan number
C
Distributions of residuals from best−fit line
of predicted RT vs Expt. scan number
Line: Z score of novel peptide
Z: −0.294
count
250
200
150
100
50
0
−10
−5
0
5
10
Z scores of residuals from best linear fit
