# Supplementary material for: Splice-Junction-Based Mapping of Alternative Isoforms in the Human Proteome
Source: Cell Rep. Author manuscript; Available in PMC 2020 Jan 15. (PMC6961840; doi:10.1016/j.celrep.2019.11.026)
Supplement: 2 [file NIHMS1546469-supplement-2.zip › DF1/PXD009021/Liver/Liver_3_MTHFD1_AAQAPSSFQLLYDLKG.pdf]

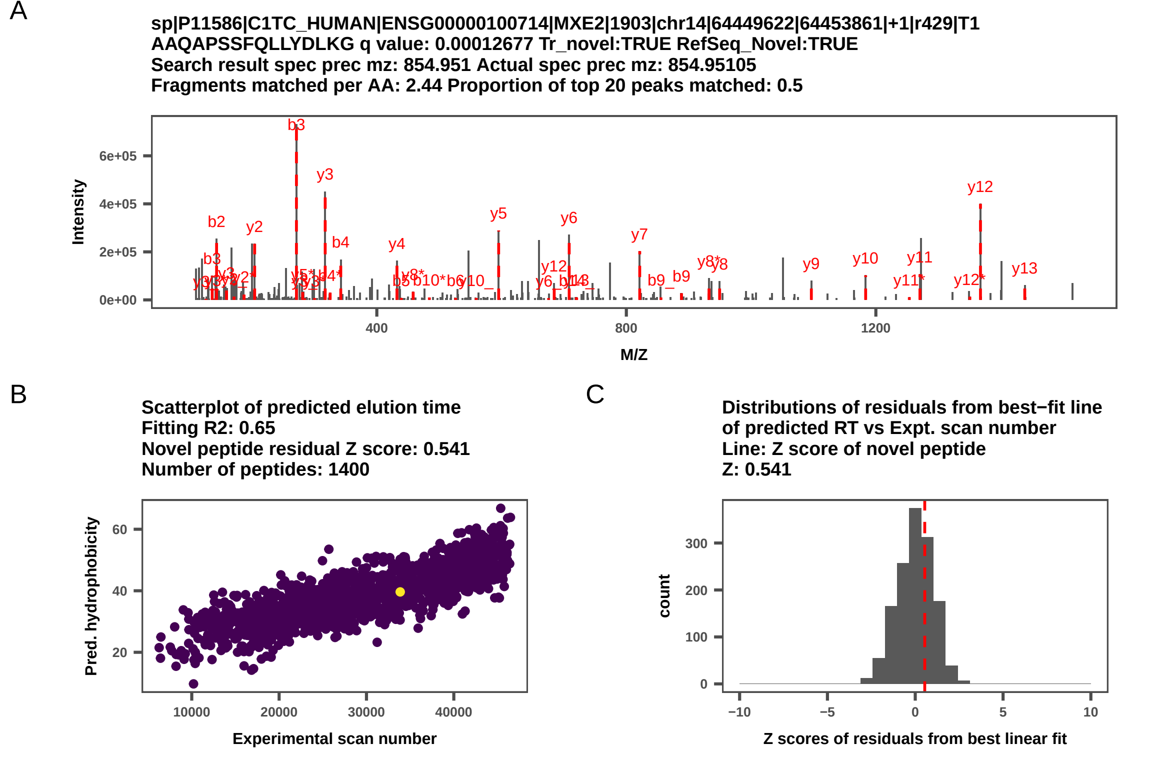
A
sp|P11586|C1TC_HUMAN|ENSG00000100714|MXE2|1903|chr14|64449622|64453861|+1|r429|T1
AAQAPSSFQLLYDLKG q value: 0.00012677 Tr_novel:TRUE RefSeq_Novel:TRUE
Search result spec prec mz: 854.951 Actual spec prec mz: 854.95105
Fragments matched per AA: 2.44 Proportion of top 20 peaks matched: 0.5
Intensity
6e+05
4e+05
2e+05
0e+00
b3
y3
b2
y2
b4
y4
y5
y6
y7
y12
y13
y10
y11
y9
y8
b9
y11*
y12*
M/Z
400
800
1200
B
Scatterplot of predicted elution time
Fitting R2: 0.65
Novel peptide residual Z score: 0.541
Number of peptides: 1400
Pred. hydrophobicity
60
40
20
10000
20000
30000
40000
Experimental scan number
C
Distributions of residuals from best−fit line
of predicted RT vs Expt. scan number
Line: Z score of novel peptide
Z: 0.541
count
300
200
100
0
−10
−5
0
5
10
Z scores of residuals from best linear fit
