# Supplementary material for: Splice-Junction-Based Mapping of Alternative Isoforms in the Human Proteome
Source: Cell Rep. Author manuscript; Available in PMC 2020 Jan 15. (PMC6961840; doi:10.1016/j.celrep.2019.11.026)
Supplement: 2 [file NIHMS1546469-supplement-2.zip › DF1/PXD009021/Liver/Liver_19_MCM3_ALLFSVELR.pdf]

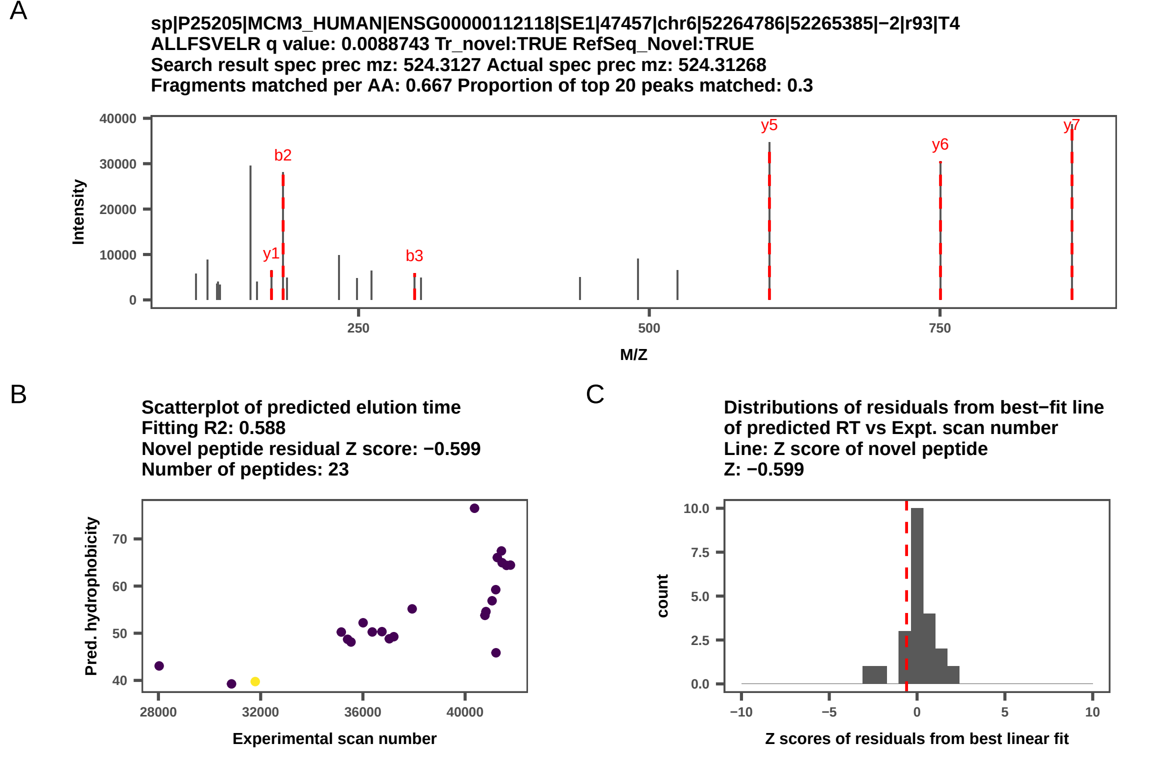

A
sp|P25205|MCM3_HUMAN|ENSG00000112118|SE1|47457|chr6|52264786|52265385|−2|r93|T4
ALLFSVELR q value: 0.0088743 Tr_novel:TRUE RefSeq_Novel:TRUE
Search result spec prec mz: 524.3127 Actual spec prec mz: 524.31268
Fragments matched per AA: 0.667 Proportion of top 20 peaks matched: 0.3
Intensity
40000
30000
20000
10000
0
y1
b2
b3
y5
y6
y7
250
500
750
M/Z
B
Scatterplot of predicted elution time
Fitting R2: 0.588
Novel peptide residual Z score: −0.599
Number of peptides: 23
Pred. hydrophobicity
70
60
50
40
28000
32000
36000
40000
Experimental scan number
C
Distributions of residuals from best−fit line
of predicted RT vs Expt. scan number
Line: Z score of novel peptide
Z: −0.599
count
10.0
7.5
5.0
2.5
0.0
−10
−5
0
5
10
Z scores of residuals from best linear fit
