# Supplementary material for: Splice-Junction-Based Mapping of Alternative Isoforms in the Human Proteome
Source: Cell Rep. Author manuscript; Available in PMC 2020 Jan 15. (PMC6961840; doi:10.1016/j.celrep.2019.11.026)
Supplement: 2 [file NIHMS1546469-supplement-2.zip › DF1/PXD009021/Liver/Liver_10_AMBP_TVGDEELLR.pdf]

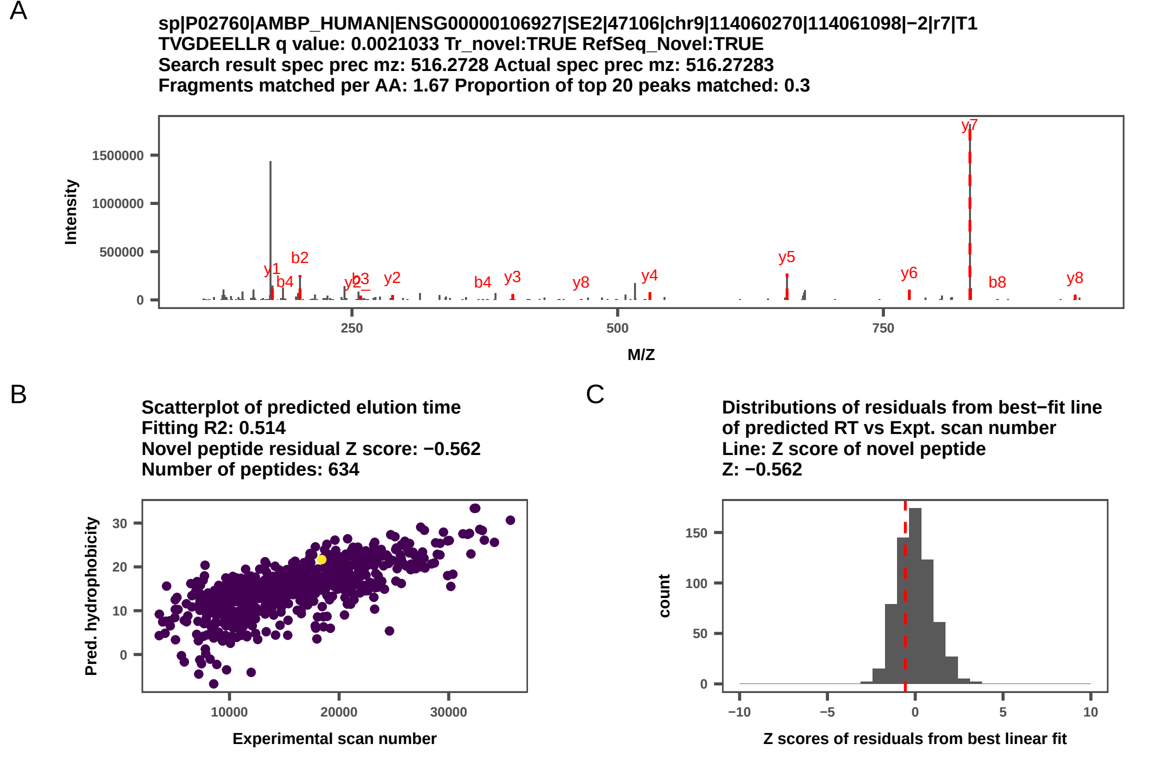

A
sp|P02760|AMBP_HUMAN|ENSG00000106927|SE2|47106|chr9|114060270|114061098|−2|r7|T1
TVGDEELLR q value: 0.0021033 Tr_novel:TRUE RefSeq_Novel:TRUE
Search result spec prec mz: 516.2728 Actual spec prec mz: 516.27283
Fragments matched per AA: 1.67 Proportion of top 20 peaks matched: 0.3
Intensity
1500000
1000000
500000
0
y7
y1
b2
b4
y2
b3
y2
b4
y3
y8
y4
y5
y6
b8
y8
250
500
750
M/Z
B
Scatterplot of predicted elution time
Fitting R2: 0.514
Novel peptide residual Z score: −0.562
Number of peptides: 634
Pred. hydrophobicity
30
20
10
0
10000
20000
30000
Experimental scan number
C
Distributions of residuals from best−fit line
of predicted RT vs Expt. scan number
Line: Z score of novel peptide
Z: −0.562
count
150
100
50
0
−10
−5
0
5
10
Z scores of residuals from best linear fit
