# Supplementary material for: Splice-Junction-Based Mapping of Alternative Isoforms in the Human Proteome
Source: Cell Rep. Author manuscript; Available in PMC 2020 Jan 15. (PMC6961840; doi:10.1016/j.celrep.2019.11.026)
Supplement: 2 [file NIHMS1546469-supplement-2.zip › DF1/PXD009021/Liver/Liver_11_RFWD2_QLEAQLEQIQK.pdf]

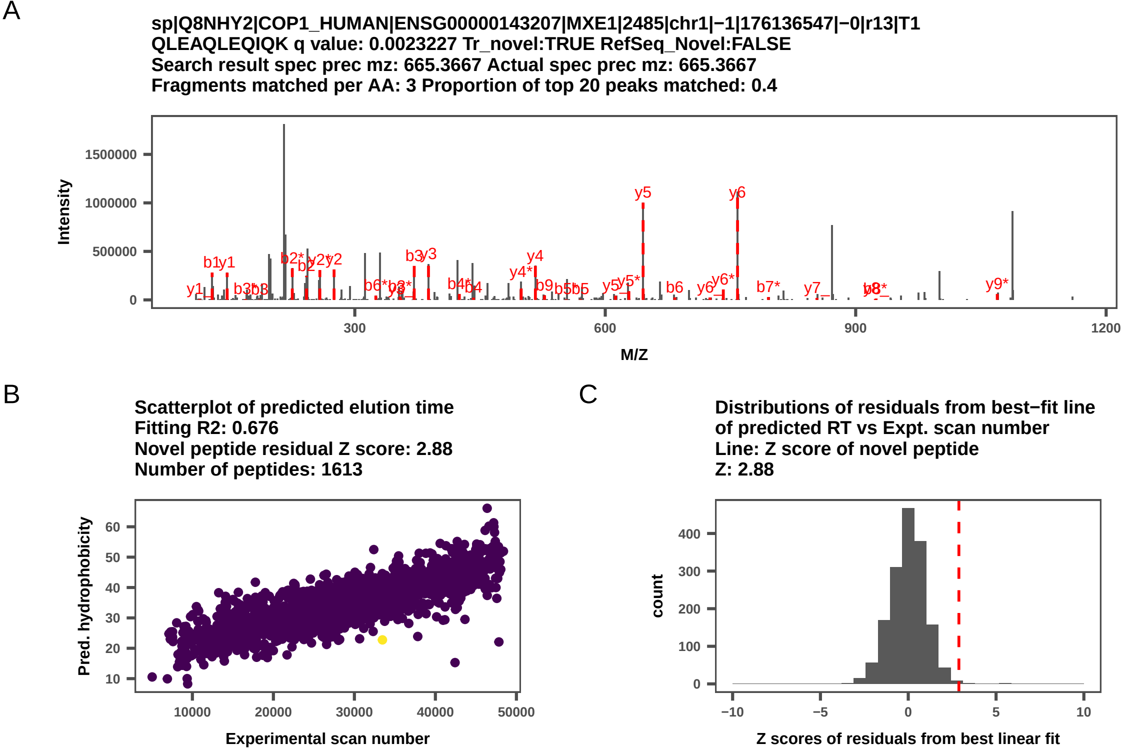

A
sp|Q8NHY2|COP1_HUMAN|ENSG00000143207|MXE1|2485|chr1|−1|176136547|−0|r13|T1
QLEAQLEQIQK q value: 0.0023227 Tr_novel:TRUE RefSeq_Novel:FALSE
Search result spec prec mz: 665.3667 Actual spec prec mz: 665.3667
Fragments matched per AA: 3 Proportion of top 20 peaks matched: 0.4
Intensity
1500000
1000000
500000
0
M/Z
300
600
900
1200
y5
y6
y4
y4*
b3
y3
b6
b7*
y7
y9*
B
Scatterplot of predicted elution time
Fitting R2: 0.676
Novel peptide residual Z score: 2.88
Number of peptides: 1613
Pred. hydrophobicity
60
50
40
30
20
10
Experimental scan number
10000
20000
30000
40000
50000
C
Distributions of residuals from best−fit line
of predicted RT vs Expt. scan number
Line: Z score of novel peptide
Z: 2.88
count
400
300
200
100
0
Z scores of residuals from best linear fit
−10
−5
0
5
10
