# Supplementary material for: Splice-Junction-Based Mapping of Alternative Isoforms in the Human Proteome
Source: Cell Rep. Author manuscript; Available in PMC 2020 Jan 15. (PMC6961840; doi:10.1016/j.celrep.2019.11.026)
Supplement: 2 [file NIHMS1546469-supplement-2.zip › DF1/PXD009021/Liver/Liver_8_HMGB1_ENILACPLVMLR.pdf]

A

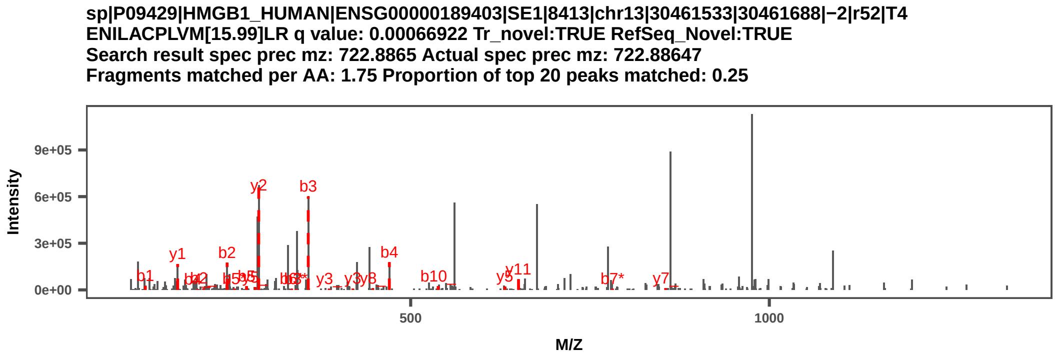
sp|P09429|HMGB1_HUMAN|ENSG00000189403|SE1|8413|chr13|30461533|30461688|−2|r52|T4
ENILACPLVM[15.99]LR q value: 0.00066922 Tr_novel:TRUE RefSeq_Novel:TRUE
Search result spec prec mz: 722.8865 Actual spec prec mz: 722.88647
Fragments matched per AA: 1.75 Proportion of top 20 peaks matched: 0.25
Intensity
9e+05
6e+05
3e+05
0e+00
y2
b3
y1
b2
b4
b1
b10
y11
b7*
y7
y3
500
1000
M/Z

B

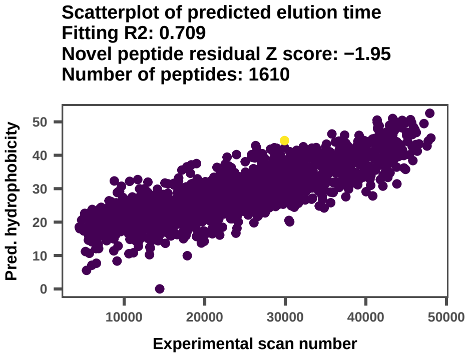
Scatterplot of predicted elution time
Fitting R2: 0.709
Novel peptide residual Z score: −1.95
Number of peptides: 1610
Pred. hydrophobicity
50
40
30
20
10
0
10000
20000
30000
40000
50000
Experimental scan number

C

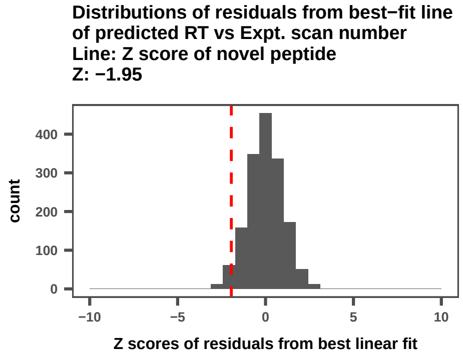
Distributions of residuals from best−fit line
of predicted RT vs Expt. scan number
Line: Z score of novel peptide
Z: −1.95
count
400
300
200
100
0
−10
−5
0
5
10
Z scores of residuals from best linear fit
