# Supplementary material for: Splice-Junction-Based Mapping of Alternative Isoforms in the Human Proteome
Source: Cell Rep. Author manuscript; Available in PMC 2020 Jan 15. (PMC6961840; doi:10.1016/j.celrep.2019.11.026)
Supplement: 2 [file NIHMS1546469-supplement-2.zip › DF1/PXD009021/Liver/Liver_4_SURF4_AMGQNDLMGTAEDFADQFLR.pdf]

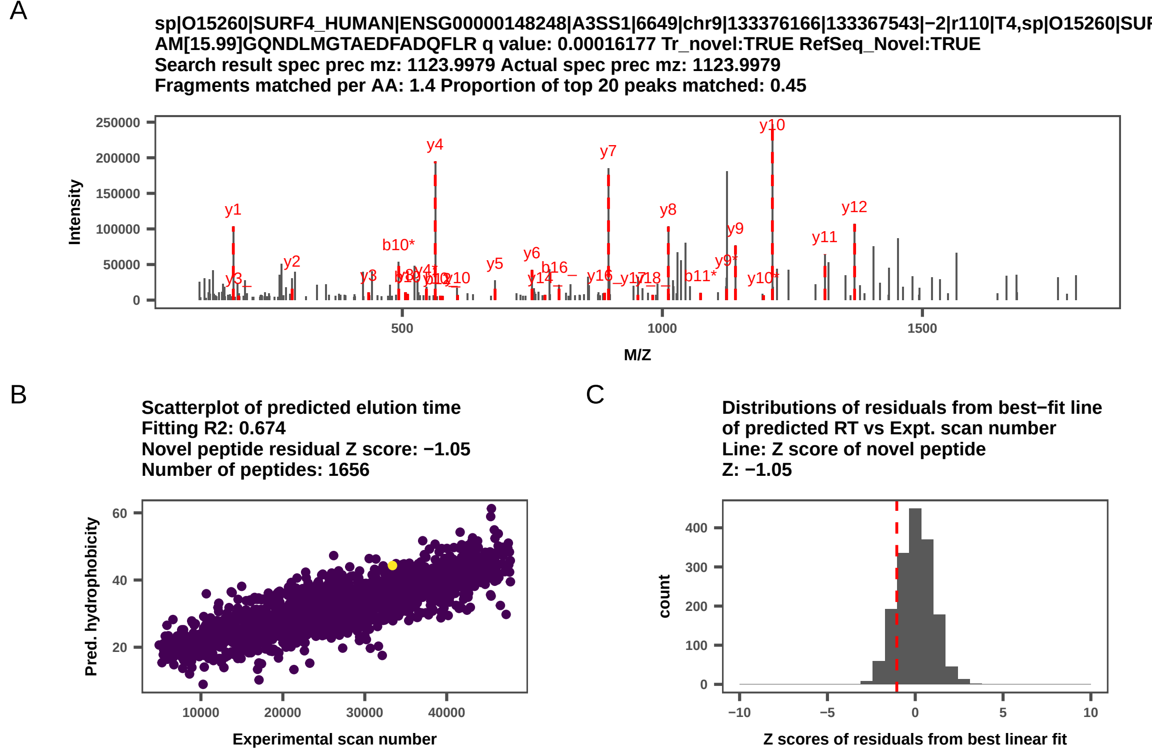
A
sp|O15260|SURF4_HUMAN|ENSG00000148248|A3SS1|6649|chr9|133376166|133367543|−2|r110|T4,sp|O15260|SUF
AM[15.99]GQNDLMGTAEDFADQFLR q value: 0.00016177 Tr_novel:TRUE RefSeq_Novel:TRUE
Search result spec prec mz: 1123.9979 Actual spec prec mz: 1123.9979
Fragments matched per AA: 1.4 Proportion of top 20 peaks matched: 0.45
Intensity
250000
200000
150000
100000
50000
0
y1
y2
y4
y5
y6
y7
y8
y9
y10
y11
y12
b10*
b16
b11*
y10*
M/Z
500
1000
1500
B
Scatterplot of predicted elution time
Fitting R2: 0.674
Novel peptide residual Z score: −1.05
Number of peptides: 1656
Pred. hydrophobicity
60
40
20
10000
20000
30000
40000
Experimental scan number
C
Distributions of residuals from best−fit line
of predicted RT vs Expt. scan number
Line: Z score of novel peptide
Z: −1.05
count
400
300
200
100
0
−10
−5
0
5
10
Z scores of residuals from best linear fit
