# Supplementary material for: Splice-Junction-Based Mapping of Alternative Isoforms in the Human Proteome
Source: Cell Rep. Author manuscript; Available in PMC 2020 Jan 15. (PMC6961840; doi:10.1016/j.celrep.2019.11.026)
Supplement: 2 [file NIHMS1546469-supplement-2.zip › DF1/PXD009021/Liver/Liver_5_ACOX1_SHVDSASFNPELLTHILDGSPEK.pdf]

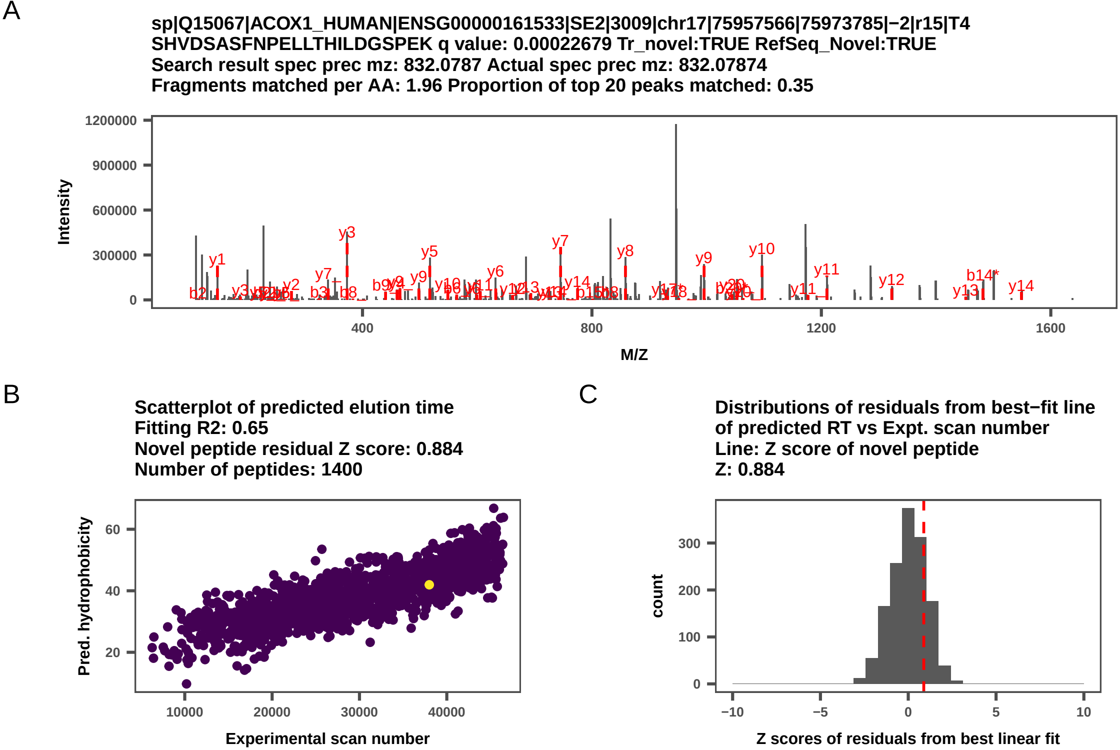
A
sp|Q15067|ACOX1_HUMAN|ENSG00000161533|SE2|3009|chr17|75957566|75973785|−2|r15|T4
SHVDSASFNPELLTHILDGSPEK q value: 0.00022679 Tr_novel:TRUE RefSeq_Novel:TRUE
Search result spec prec mz: 832.0787 Actual spec prec mz: 832.07874
Fragments matched per AA: 1.96 Proportion of top 20 peaks matched: 0.35
Intensity
1200000
900000
600000
300000
0
y1
y3
y5
y7
y8
y9
y10
y11
y12
y14
b14*
y6
y13
M/Z
400
800
1200
1600
B
Scatterplot of predicted elution time
Fitting R2: 0.65
Novel peptide residual Z score: 0.884
Number of peptides: 1400
Pred. hydrophobicity
60
40
20
10000
20000
30000
40000
Experimental scan number
C
Distributions of residuals from best−fit line
of predicted RT vs Expt. scan number
Line: Z score of novel peptide
Z: 0.884
count
300
200
100
0
−10
−5
0
5
10
Z scores of residuals from best linear fit
