# Supplementary material for: Splice-Junction-Based Mapping of Alternative Isoforms in the Human Proteome
Source: Cell Rep. Author manuscript; Available in PMC 2020 Jan 15. (PMC6961840; doi:10.1016/j.celrep.2019.11.026)
Supplement: 2 [file NIHMS1546469-supplement-2.zip › DF1/PXD009021/Liver/Liver_18_ASMTL_APDVVIGADTIVVEWER.pdf]

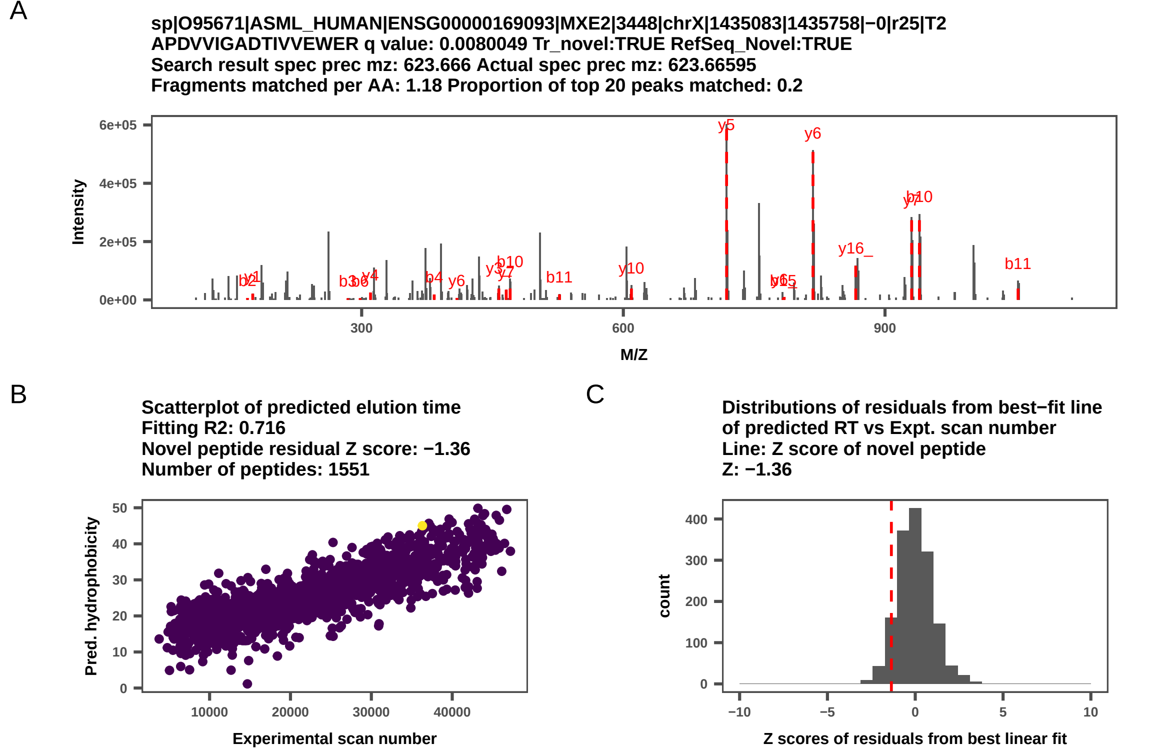
A
sp|O95671|ASML_HUMAN|ENSG00000169093|MXE2|3448|chrX|1435083|1435758|−0|r25|T2
APDVVIGADTIVVEWER q value: 0.0080049 Tr_novel:TRUE RefSeq_Novel:TRUE
Search result spec prec mz: 623.666 Actual spec prec mz: 623.66595
Fragments matched per AA: 1.18 Proportion of top 20 peaks matched: 0.2
Intensity
6e+05
4e+05
2e+05
0e+00
300
600
900
M/Z
y5
y6
y7
b10
y16_
b11
y10
b4
y6
b11
y4
y1
b2
B
Scatterplot of predicted elution time
Fitting R2: 0.716
Novel peptide residual Z score: −1.36
Number of peptides: 1551
Pred. hydrophobicity
50
40
30
20
10
0
10000
20000
30000
40000
Experimental scan number
C
Distributions of residuals from best−fit line
of predicted RT vs Expt. scan number
Line: Z score of novel peptide
Z: −1.36
count
400
300
200
100
0
−10
−5
0
5
10
Z scores of residuals from best linear fit
