# Supplementary material for: Splice-Junction-Based Mapping of Alternative Isoforms in the Human Proteome
Source: Cell Rep. Author manuscript; Available in PMC 2020 Jan 15. (PMC6961840; doi:10.1016/j.celrep.2019.11.026)
Supplement: 2 [file NIHMS1546469-supplement-2.zip › DF1/PXD009021/Liver/Liver_6_EEF1A1_DMSLLSLKADCAVLIVAAGVGEFEAGISK.pdf]

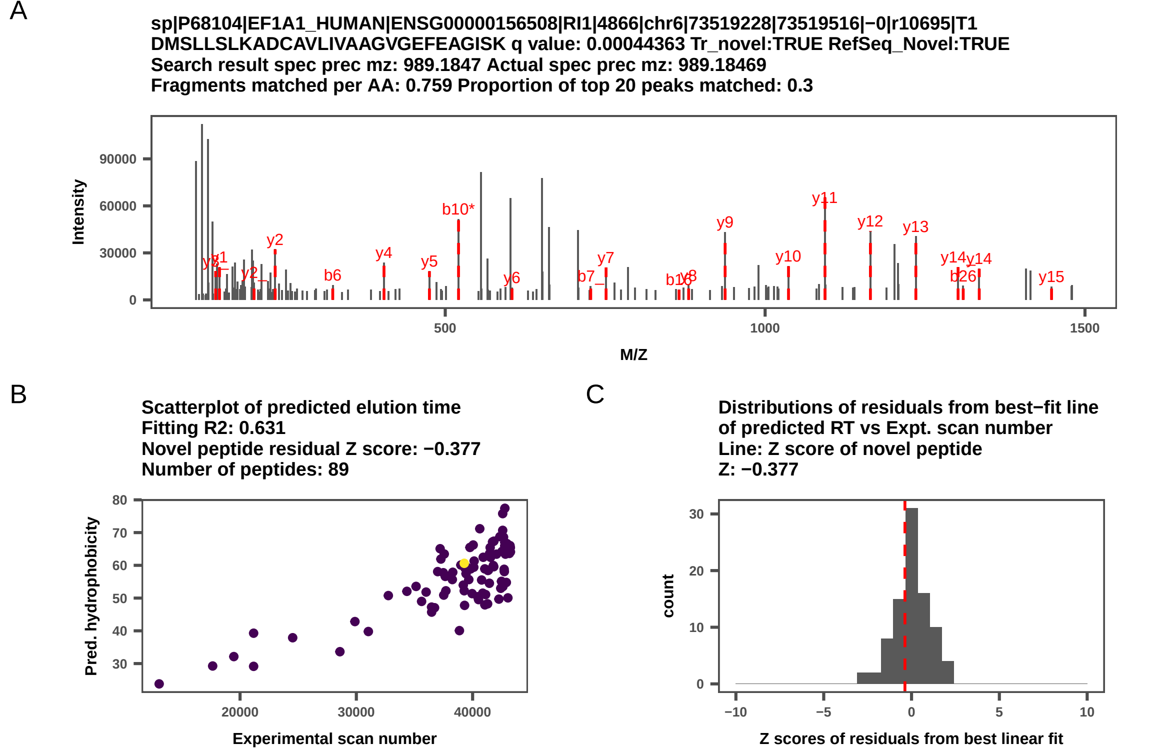
A
sp|P68104|EF1A1_HUMAN|ENSG00000156508|RI1|4866|chr6|73519228|73519516|−0|r10695|T1
DMSLLSLKADCAVLIVAAGVGEFEAGISK q value: 0.00044363 Tr_novel:TRUE RefSeq_Novel:TRUE
Search result spec prec mz: 989.1847 Actual spec prec mz: 989.18469
Fragments matched per AA: 0.759 Proportion of top 20 peaks matched: 0.3
Intensity
90000
60000
30000
0
y2
b10*
y4
y5
b6
y6
y7
b7
y9
y10
y11
y12
y13
y14
b26
y15
500
1000
1500
M/Z
B
Scatterplot of predicted elution time
Fitting R2: 0.631
Novel peptide residual Z score: −0.377
Number of peptides: 89
Pred. hydrophobicity
80
70
60
50
40
30
20000
30000
40000
Experimental scan number
C
Distributions of residuals from best−fit line
of predicted RT vs Expt. scan number
Line: Z score of novel peptide
Z: −0.377
count
30
20
10
0
−10
−5
0
5
10
Z scores of residuals from best linear fit
