# Supplementary material for: Splice-Junction-Based Mapping of Alternative Isoforms in the Human Proteome
Source: Cell Rep. Author manuscript; Available in PMC 2020 Jan 15. (PMC6961840; doi:10.1016/j.celrep.2019.11.026)
Supplement: 2 [file NIHMS1546469-supplement-2.zip › DF1/PXD009021/Liver/Liver_16_UBE2J2_SIAQTMSSEAQRPTTATQR.pdf]

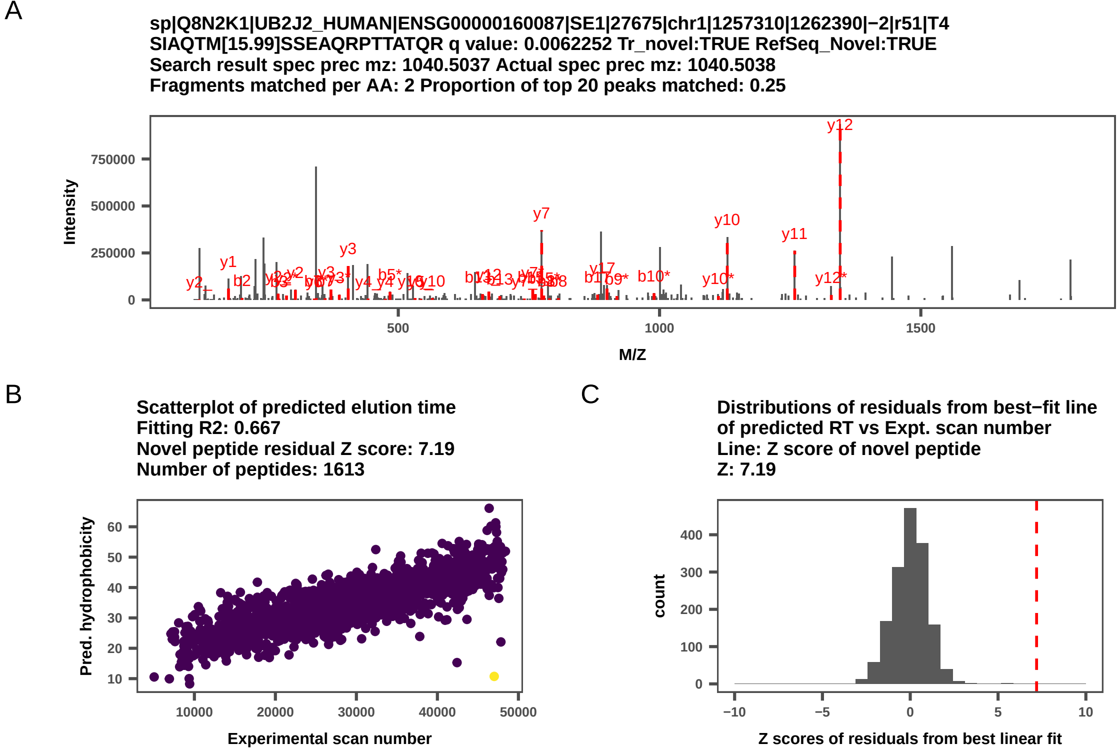
A
sp|Q8N2K1|UB2J2_HUMAN|ENSG00000160087|SE1|27675|chr1|1257310|1262390|−2|r51|T4
SIAQTM[15.99]SSEAQRPTTATQR q value: 0.0062252 Tr_novel:TRUE RefSeq_Novel:TRUE
Search result spec prec mz: 1040.5037 Actual spec prec mz: 1040.5038
Fragments matched per AA: 2 Proportion of top 20 peaks matched: 0.25
Intensity
750000
500000
250000
0
y12
y7
y10
y11
y3
y1
b2
y12*
y10*
b10*
y17
500
1000
1500
M/Z
B
Scatterplot of predicted elution time
Fitting R2: 0.667
Novel peptide residual Z score: 7.19
Number of peptides: 1613
Pred. hydrophobicity
60
50
40
30
20
10
10000
20000
30000
40000
50000
Experimental scan number
C
Distributions of residuals from best−fit line
of predicted RT vs Expt. scan number
Line: Z score of novel peptide
Z: 7.19
count
400
300
200
100
0
−10
−5
0
5
10
Z scores of residuals from best linear fit
