# Supplementary material for: Splice-Junction-Based Mapping of Alternative Isoforms in the Human Proteome
Source: Cell Rep. Author manuscript; Available in PMC 2020 Jan 15. (PMC6961840; doi:10.1016/j.celrep.2019.11.026)

A

sp|O95255|MRP6_HUMAN|ENSG00000091262|A5SS1|659|chr16|16219947|16221831|−0|r100|T1
MVAAIPGSLEPGNVLGFALIVLCTSSVAVALW q value: 0.0063863 Tr_novel:TRUE RefSeq_Novel:FALSE
Search result spec prec mz: 1086.2579 Actual spec prec mz: 1086.2579
Fragments matched per AA: 1 Proportion of top 20 peaks matched: 0.25

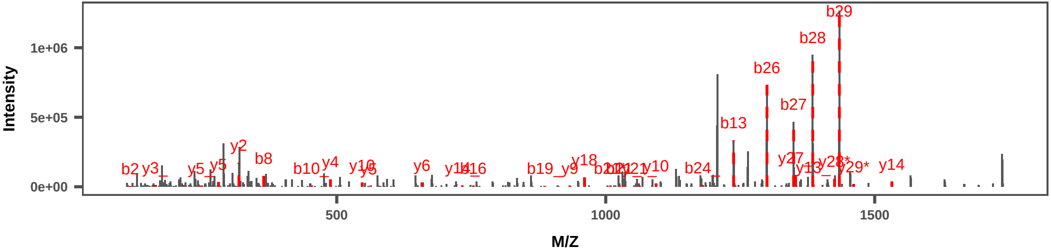

B

Scatterplot of predicted elution time
Fitting R2: 0.597
Novel peptide residual Z score: −5.9
Number of peptides: 1360

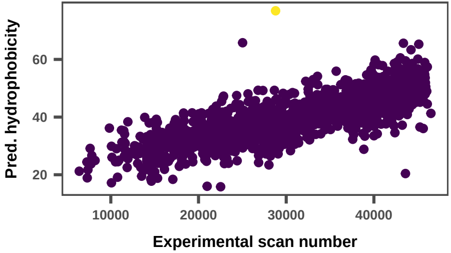

C

Distributions of residuals from best−fit line
of predicted RT vs Expt. scan number
Line: Z score of novel peptide
Z: −5.9

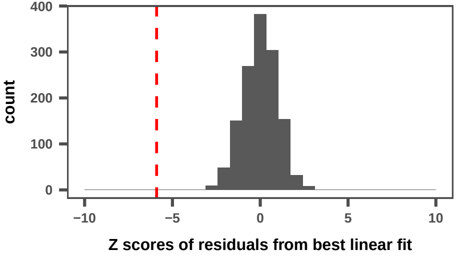

Supplement: 2 [file NIHMS1546469-supplement-2.zip › DF1/PXD009021/Liver/Liver_17_ABCC6_MVAAIPGSLEPGNVLGFALIVLCTSSVAVALW.pdf]
