# Supplementary material for: Splice-Junction-Based Mapping of Alternative Isoforms in the Human Proteome
Source: Cell Rep. Author manuscript; Available in PMC 2020 Jan 15. (PMC6961840; doi:10.1016/j.celrep.2019.11.026)
Supplement: 2 [file NIHMS1546469-supplement-2.zip › DF1/PXD009021/Liver/Liver_9_ERBB2_DPGLLLALLPPGAASTQVCTGTDMK.pdf]

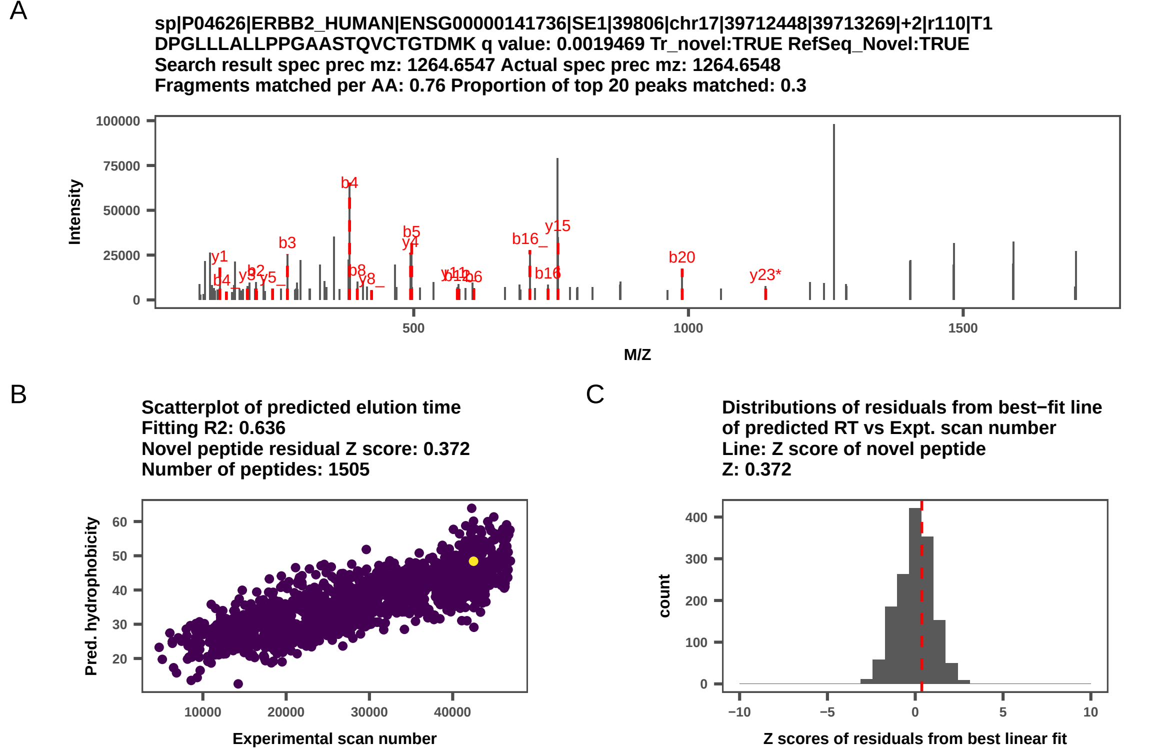
A
sp|P04626|ERBB2_HUMAN|ENSG00000141736|SE1|39806|chr17|39712448|39713269|+2|r110|T1
DPGLLLALLPPGAASTQVCTGTDMK q value: 0.0019469 Tr_novel:TRUE RefSeq_Novel:TRUE
Search result spec prec mz: 1264.6547 Actual spec prec mz: 1264.6548
Fragments matched per AA: 0.76 Proportion of top 20 peaks matched: 0.3
Intensity
100000
75000
50000
25000
0
b4
b5
y4
b3
y1
b2
y5_
b8
y8_
b16_
y15
b16
b20
y23*
500
1000
1500
M/Z
B
Scatterplot of predicted elution time
Fitting R2: 0.636
Novel peptide residual Z score: 0.372
Number of peptides: 1505
Pred. hydrophobicity
60
50
40
30
20
10000
20000
30000
40000
Experimental scan number
C
Distributions of residuals from best−fit line
of predicted RT vs Expt. scan number
Line: Z score of novel peptide
Z: 0.372
count
400
300
200
100
0
−10
−5
0
5
10
Z scores of residuals from best linear fit
