# Supplementary material for: Splice-Junction-Based Mapping of Alternative Isoforms in the Human Proteome
Source: Cell Rep. Author manuscript; Available in PMC 2020 Jan 15. (PMC6961840; doi:10.1016/j.celrep.2019.11.026)
Supplement: 2 [file NIHMS1546469-supplement-2.zip › DF1/PXD009021/Liver/Liver_12_KIAA1468_PTMSYYGSGGGVNPFLSDSDEDDDEVAATEER.pdf]

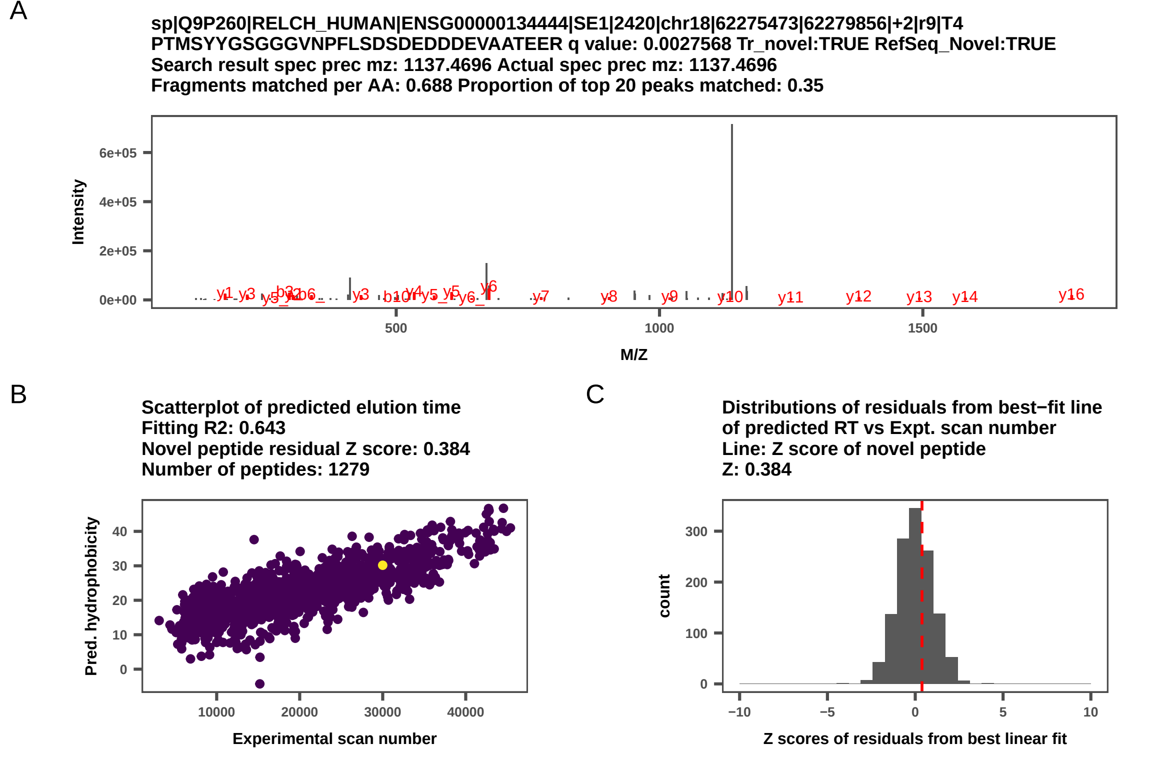
A
sp|Q9P260|RELCH_HUMAN|ENSG00000134444|SE1|2420|chr18|62275473|62279856|+2|r9|T4
PTMSYYGSGGGVNPFLSDSDEDDDEVAATEER q value: 0.0027568 Tr_novel:TRUE RefSeq_Novel:TRUE
Search result spec prec mz: 1137.4696 Actual spec prec mz: 1137.4696
Fragments matched per AA: 0.688 Proportion of top 20 peaks matched: 0.35
Intensity
6e+05
4e+05
2e+05
0e+00
y1
y3
y3
y4
y5
y6
y7
y8
y9
y10
y11
y12
y13
y14
y16
500
1000
1500
M/Z
B
Scatterplot of predicted elution time
Fitting R2: 0.643
Novel peptide residual Z score: 0.384
Number of peptides: 1279
Pred. hydrophobicity
40
30
20
10
0
10000
20000
30000
40000
Experimental scan number
C
Distributions of residuals from best−fit line
of predicted RT vs Expt. scan number
Line: Z score of novel peptide
Z: 0.384
count
300
200
100
0
−10
−5
0
5
10
Z scores of residuals from best linear fit
