# Supplementary material for: Splice-Junction-Based Mapping of Alternative Isoforms in the Human Proteome
Source: Cell Rep. Author manuscript; Available in PMC 2020 Jan 15. (PMC6961840; doi:10.1016/j.celrep.2019.11.026)
Supplement: 2 [file NIHMS1546469-supplement-2.zip › DF1/PXD009021/Liver/Liver_13_SYMPK_EPEAKGNLPPAPPSCSP.pdf]

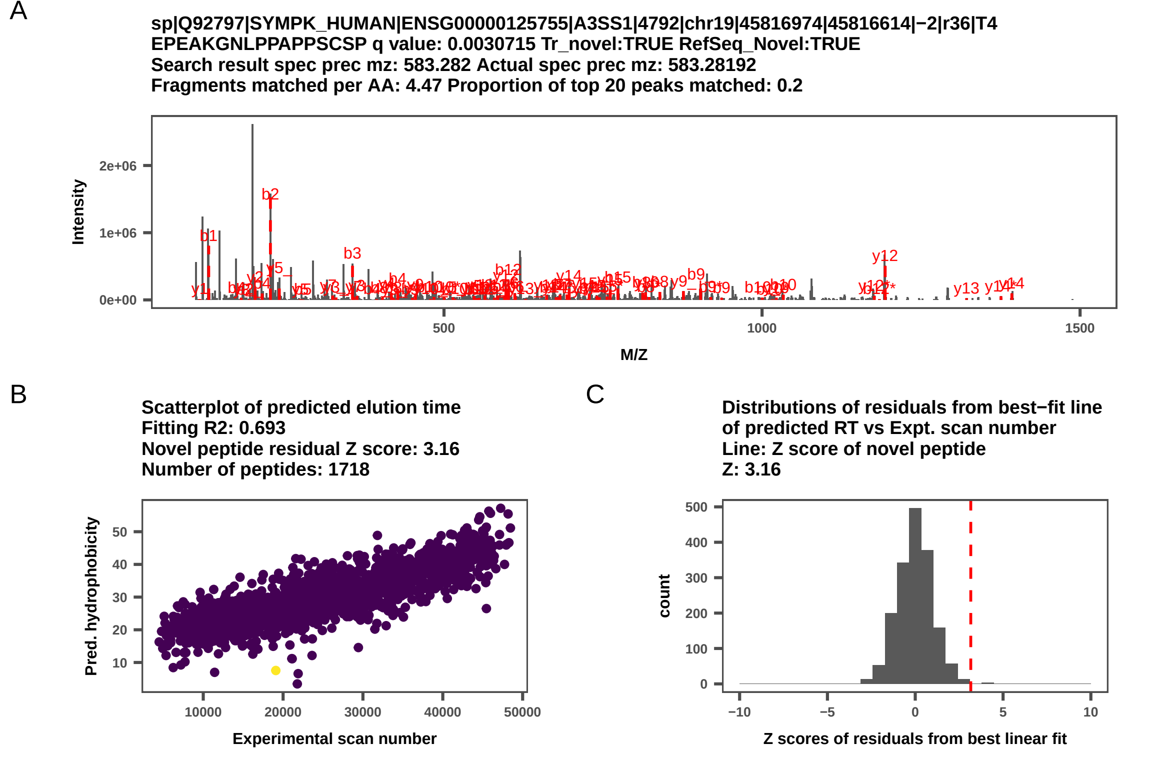

A
sp|Q92797|SYMPK_HUMAN|ENSG00000125755|A3SS1|4792|chr19|45816974|45816614|−2|r36|T4
EPEAKGNLPPAPPSCSP q value: 0.0030715 Tr_novel:TRUE RefSeq_Novel:TRUE
Search result spec prec mz: 583.282 Actual spec prec mz: 583.28192
Fragments matched per AA: 4.47 Proportion of top 20 peaks matched: 0.2
Intensity
2e+06
1e+06
0e+00
b1
b2
b3
y12
y13
500
1000
1500
M/Z
B
Scatterplot of predicted elution time
Fitting R2: 0.693
Novel peptide residual Z score: 3.16
Number of peptides: 1718
Pred. hydrophobicity
50
40
30
20
10
10000
20000
30000
40000
50000
Experimental scan number
C
Distributions of residuals from best−fit line
of predicted RT vs Expt. scan number
Line: Z score of novel peptide
Z: 3.16
count
500
400
300
200
100
0
−10
−5
0
5
10
Z scores of residuals from best linear fit
