# Supplementary material for: Splice-Junction-Based Mapping of Alternative Isoforms in the Human Proteome
Source: Cell Rep. Author manuscript; Available in PMC 2020 Jan 15. (PMC6961840; doi:10.1016/j.celrep.2019.11.026)
Supplement: 2 [file NIHMS1546469-supplement-2.zip › DF1/PXD006675/PulmonaryValve/PulmonaryValve_9_LONP1_EIFDIAFPDEQAEALAVEGPTWAPCPGRSSSVLWGAAR.pdf]

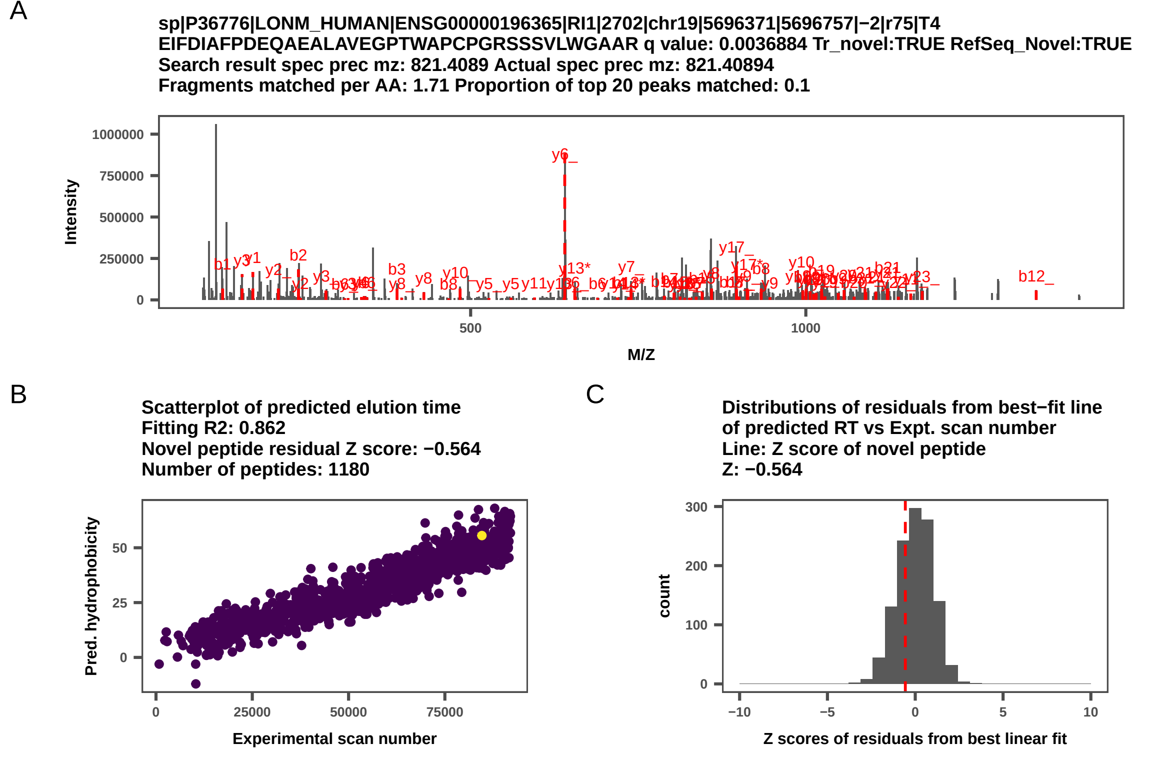
A
sp|P36776|LONM_HUMAN|ENSG00000196365|RI1|2702|chr19|5696371|5696757|−2|r75|T4
EIFDIAFPDEQAEALAVEGPTWAPCPGRSSSVLWGAAR q value: 0.0036884 Tr_novel:TRUE RefSeq_Novel:TRUE
Search result spec prec mz: 821.4089 Actual spec prec mz: 821.40894
Fragments matched per AA: 1.71 Proportion of top 20 peaks matched: 0.1
Intensity
1000000
750000
500000
250000
0
500
1000
M/Z
y6_
y17_
b12_
B
Scatterplot of predicted elution time
Fitting R2: 0.862
Novel peptide residual Z score: −0.564
Number of peptides: 1180
Pred. hydrophobicity
50
25
0
0
25000
50000
75000
Experimental scan number
C
Distributions of residuals from best−fit line
of predicted RT vs Expt. scan number
Line: Z score of novel peptide
Z: −0.564
count
300
200
100
0
−10
−5
0
5
10
Z scores of residuals from best linear fit
