# Supplementary material for: Splice-Junction-Based Mapping of Alternative Isoforms in the Human Proteome
Source: Cell Rep. Author manuscript; Available in PMC 2020 Jan 15. (PMC6961840; doi:10.1016/j.celrep.2019.11.026)
Supplement: 2 [file NIHMS1546469-supplement-2.zip › DF1/PXD006675/PulmonaryValve/PulmonaryValve_2_TNXB_VGPVSAVGVTAPGK.pdf]

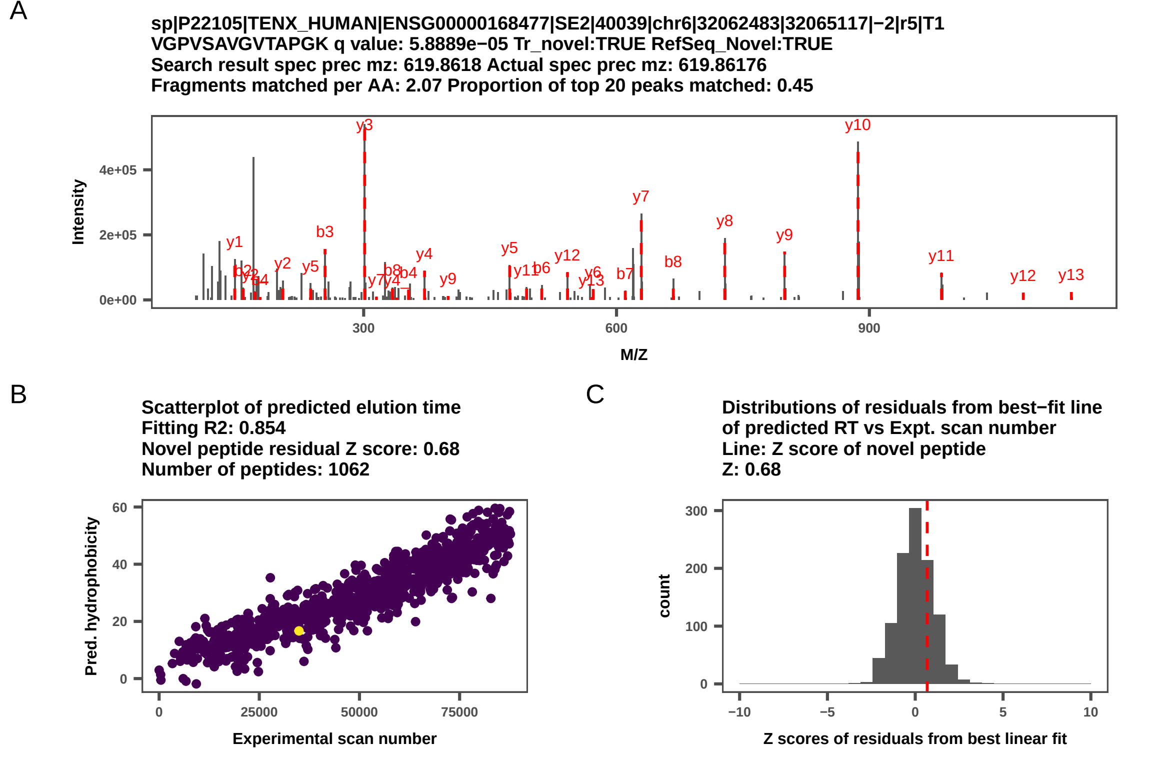
A
sp|P22105|TENX_HUMAN|ENSG00000168477|SE2|40039|chr6|32062483|32065117|−2|r5|T1
VGPVSAVGVTAPGK q value: 5.8889e−05 Tr_novel:TRUE RefSeq_Novel:TRUE
Search result spec prec mz: 619.8618 Actual spec prec mz: 619.86176
Fragments matched per AA: 2.07 Proportion of top 20 peaks matched: 0.45
Intensity
4e+05
2e+05
0e+00
300
600
900
M/Z
y1 b2 y2 b4 y2 y5 b3 y3 y7 y4 b8 b4 y4 y9 y5 y11 b6 y12 y6 y13 b7 y7 b8 y8 y9 y10 y11 y12 y13
B
Scatterplot of predicted elution time
Fitting R2: 0.854
Novel peptide residual Z score: 0.68
Number of peptides: 1062
Pred. hydrophobicity
60
40
20
0
0
25000
50000
75000
Experimental scan number
C
Distributions of residuals from best−fit line
of predicted RT vs Expt. scan number
Line: Z score of novel peptide
Z: 0.68
count
300
200
100
0
−10
−5
0
5
10
Z scores of residuals from best linear fit
