# Supplementary material for: Splice-Junction-Based Mapping of Alternative Isoforms in the Human Proteome
Source: Cell Rep. Author manuscript; Available in PMC 2020 Jan 15. (PMC6961840; doi:10.1016/j.celrep.2019.11.026)
Supplement: 2 [file NIHMS1546469-supplement-2.zip › DF1/PXD006675/PulmonaryValve/PulmonaryValve_1_DPP7_LPGALLPAASGPLQLR.pdf]

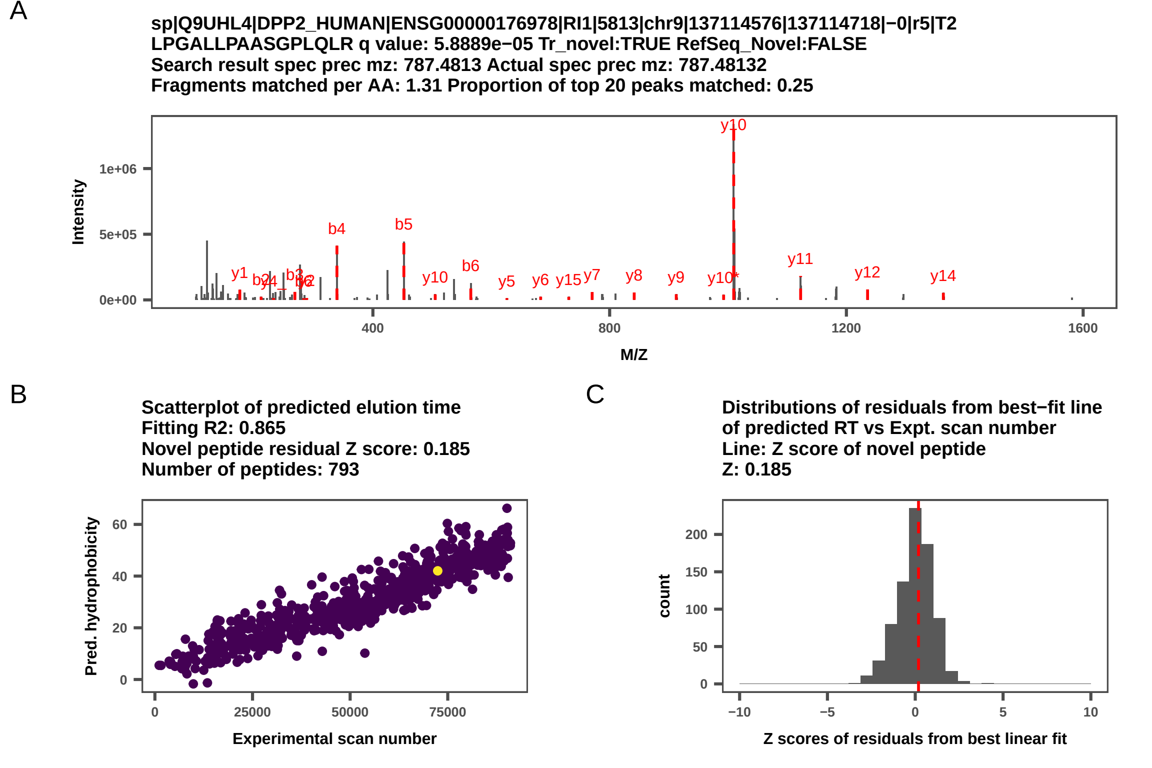
A
sp|Q9UHL4|DPP2_HUMAN|ENSG00000176978|RI1|5813|chr9|137114576|137114718|−0|r5|T2
LPGALLPAASGPLQLR q value: 5.8889e−05 Tr_novel:TRUE RefSeq_Novel:FALSE
Search result spec prec mz: 787.4813 Actual spec prec mz: 787.48132
Fragments matched per AA: 1.31 Proportion of top 20 peaks matched: 0.25
Intensity
1e+06
5e+05
0e+00
y10
b4
b5
b6
y1
y10
y5
y6
y15
y7
y8
y9
y10*
y11
y12
y14
400
800
1200
1600
M/Z
B
Scatterplot of predicted elution time
Fitting R2: 0.865
Novel peptide residual Z score: 0.185
Number of peptides: 793
Pred. hydrophobicity
60
40
20
0
0
25000
50000
75000
Experimental scan number
C
Distributions of residuals from best−fit line
of predicted RT vs Expt. scan number
Line: Z score of novel peptide
Z: 0.185
count
200
150
100
50
0
−10
−5
0
5
10
Z scores of residuals from best linear fit
