# Supplementary material for: Splice-Junction-Based Mapping of Alternative Isoforms in the Human Proteome
Source: Cell Rep. Author manuscript; Available in PMC 2020 Jan 15. (PMC6961840; doi:10.1016/j.celrep.2019.11.026)
Supplement: 2 [file NIHMS1546469-supplement-2.zip › DF1/PXD006675/PulmonaryValve/PulmonaryValve_7_ELN_AQLLPGALAAAK.pdf]

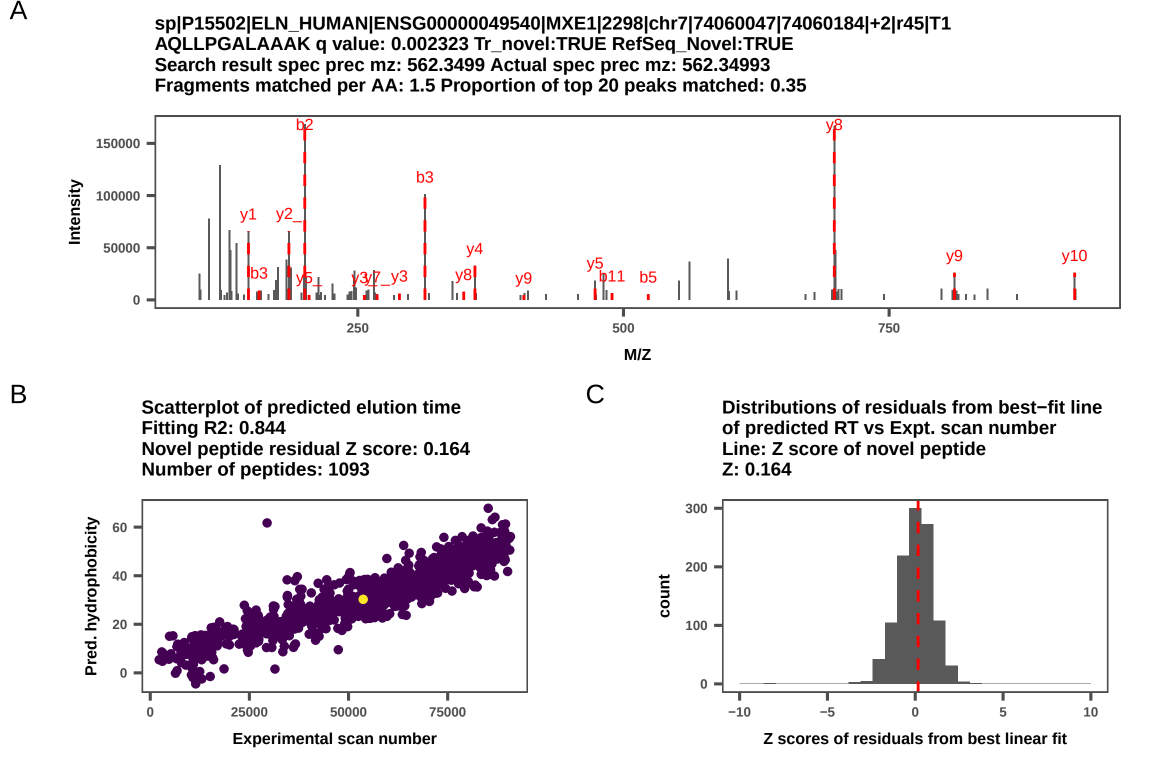
A
sp|P15502|ELN_HUMAN|ENSG00000049540|MXE1|2298|chr7|74060047|74060184|+2|r45|T1
AQLLPGALAAAK q value: 0.002323 Tr_novel:TRUE RefSeq_Novel:TRUE
Search result spec prec mz: 562.3499 Actual spec prec mz: 562.34993
Fragments matched per AA: 1.5 Proportion of top 20 peaks matched: 0.35
Intensity
150000
100000
50000
0
b2
y8
b3
y1
y2_
b3
y5_
y3
y7_
y3
y4
y8
y9
y5
b11
b5
y9
y10
250
500
750
M/Z
B
Scatterplot of predicted elution time
Fitting R2: 0.844
Novel peptide residual Z score: 0.164
Number of peptides: 1093
Pred. hydrophobicity
60
40
20
0
0
25000
50000
75000
Experimental scan number
C
Distributions of residuals from best−fit line
of predicted RT vs Expt. scan number
Line: Z score of novel peptide
Z: 0.164
count
300
200
100
0
−10
−5
0
5
10
Z scores of residuals from best linear fit
