# Supplementary material for: Splice-Junction-Based Mapping of Alternative Isoforms in the Human Proteome
Source: Cell Rep. Author manuscript; Available in PMC 2020 Jan 15. (PMC6961840; doi:10.1016/j.celrep.2019.11.026)
Supplement: 2 [file NIHMS1546469-supplement-2.zip › DF1/PXD006675/PulmonaryValve/PulmonaryValve_8_CCS_GSGQLRVQDVEVHLEDQMVLVHTTLPSQEVQALLEGTGR.pdf]

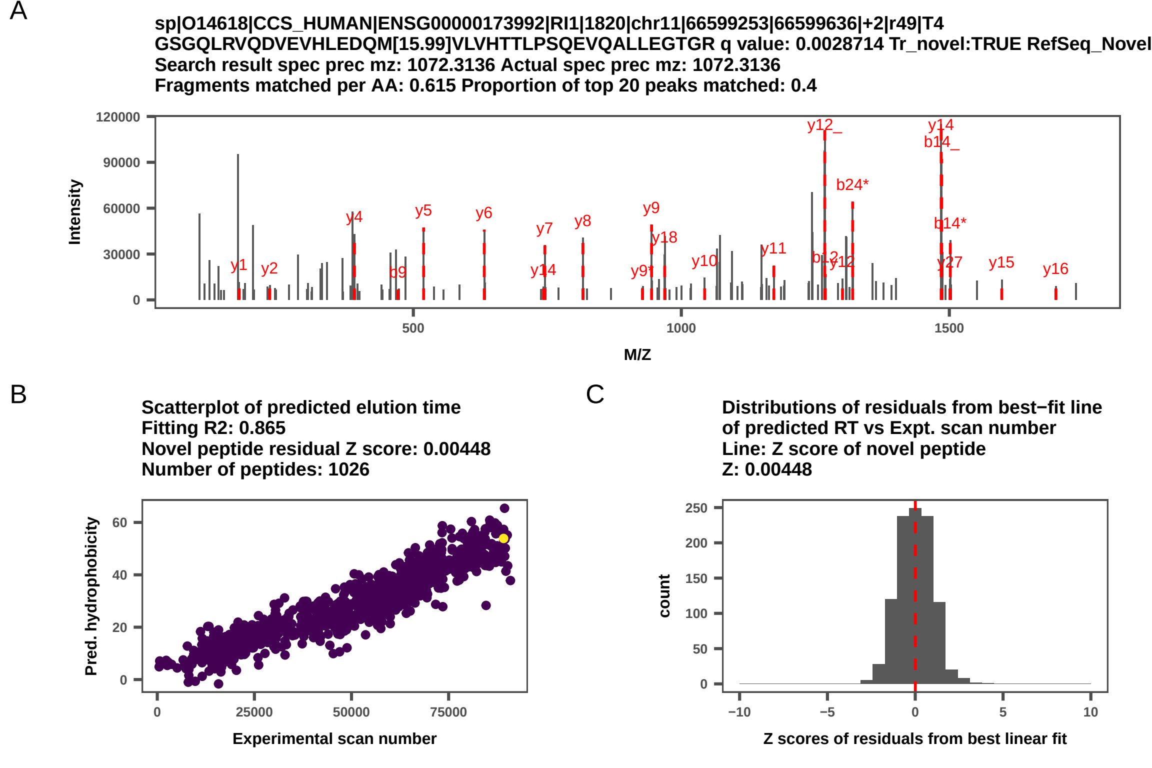

A
sp|O14618|CCS_HUMAN|ENSG00000173992|RI1|1820|chr11|66599253|66599636|+2|r49|T4
GSGQLRVQDVEVHLEDQM[15.99]VLVHTTLPSQEVQALLEGTGR q value: 0.0028714 Tr_novel:TRUE RefSeq_Novel
Search result spec prec mz: 1072.3136 Actual spec prec mz: 1072.3136
Fragments matched per AA: 0.615 Proportion of top 20 peaks matched: 0.4
Intensity
120000
90000
60000
30000
0
y1
y2
y4
b9
y5
y6
y7
y14
y8
y9
y9*
y18
y10
y11
y12_
b12
y12
b24*
y14
b14_
b14*
y27
y15
y16
500
1000
1500
M/Z
B
Scatterplot of predicted elution time
Fitting R2: 0.865
Novel peptide residual Z score: 0.00448
Number of peptides: 1026
Pred. hydrophobicity
60
40
20
0
0
25000
50000
75000
Experimental scan number
C
Distributions of residuals from best−fit line
of predicted RT vs Expt. scan number
Line: Z score of novel peptide
Z: 0.00448
count
250
200
150
100
50
0
−10
−5
0
5
10
Z scores of residuals from best linear fit
