# Supplementary material for: Splice-Junction-Based Mapping of Alternative Isoforms in the Human Proteome
Source: Cell Rep. Author manuscript; Available in PMC 2020 Jan 15. (PMC6961840; doi:10.1016/j.celrep.2019.11.026)
Supplement: 2 [file NIHMS1546469-supplement-2.zip › DF1/PXD006675/EndothelialCells/EndothelialCells_4_NPAS2_GQGTNPGPHPCLQATPLMSSSLAPHSDR.pdf]

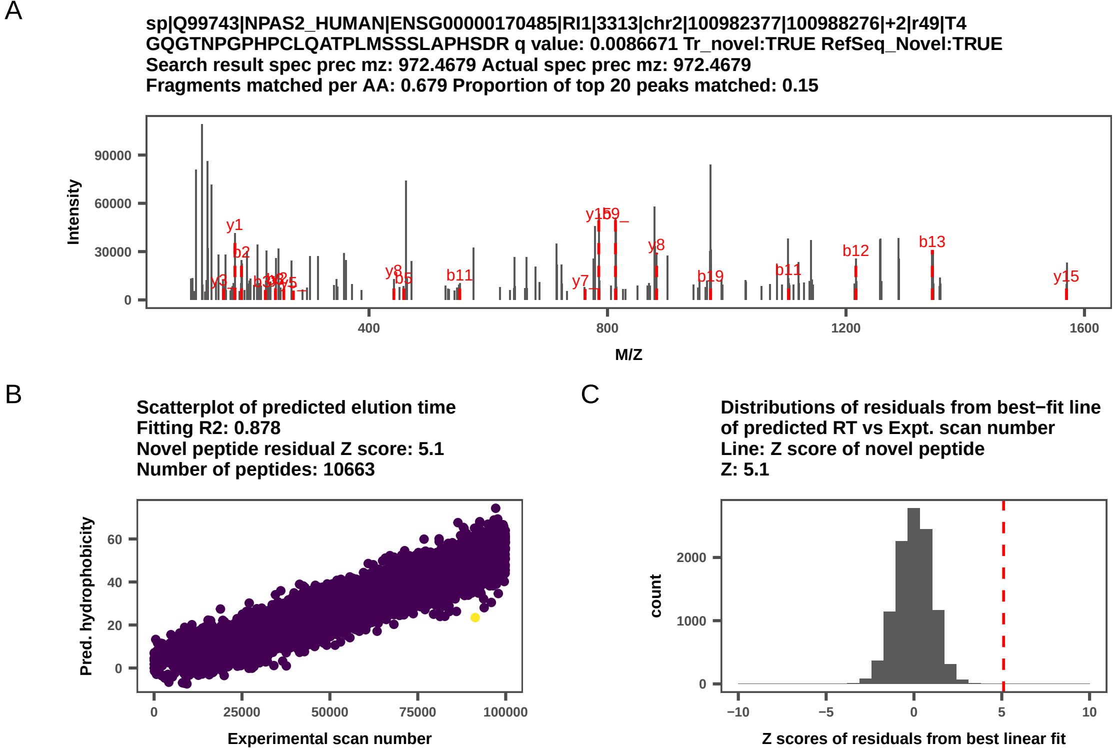
A
sp|Q99743|NPAS2_HUMAN|ENSG00000170485|RI1|3313|chr2|100982377|100988276|+2|r49|T4
GQGTNPGPHPCLQATPLMSSSLAPHSDR q value: 0.0086671 Tr_novel:TRUE RefSeq_Novel:TRUE
Search result spec prec mz: 972.4679 Actual spec prec mz: 972.4679
Fragments matched per AA: 0.679 Proportion of top 20 peaks matched: 0.15
Intensity
90000
60000
30000
0
400
800
1200
1600
M/Z
y1
b2
y3
y8
b5
b11
y7
y8
b19
b11
b12
b13
y15
B
Scatterplot of predicted elution time
Fitting R2: 0.878
Novel peptide residual Z score: 5.1
Number of peptides: 10663
Pred. hydrophobicity
60
40
20
0
0
25000
50000
75000
100000
Experimental scan number
C
Distributions of residuals from best−fit line
of predicted RT vs Expt. scan number
Line: Z score of novel peptide
Z: 5.1
count
2000
1000
0
−10
−5
0
5
10
Z scores of residuals from best linear fit
