# Supplementary material for: Splice-Junction-Based Mapping of Alternative Isoforms in the Human Proteome
Source: Cell Rep. Author manuscript; Available in PMC 2020 Jan 15. (PMC6961840; doi:10.1016/j.celrep.2019.11.026)
Supplement: 2 [file NIHMS1546469-supplement-2.zip › DF1/PXD006675/EndothelialCells/EndothelialCells_3_SRRM1_KVELSESEDK.pdf]

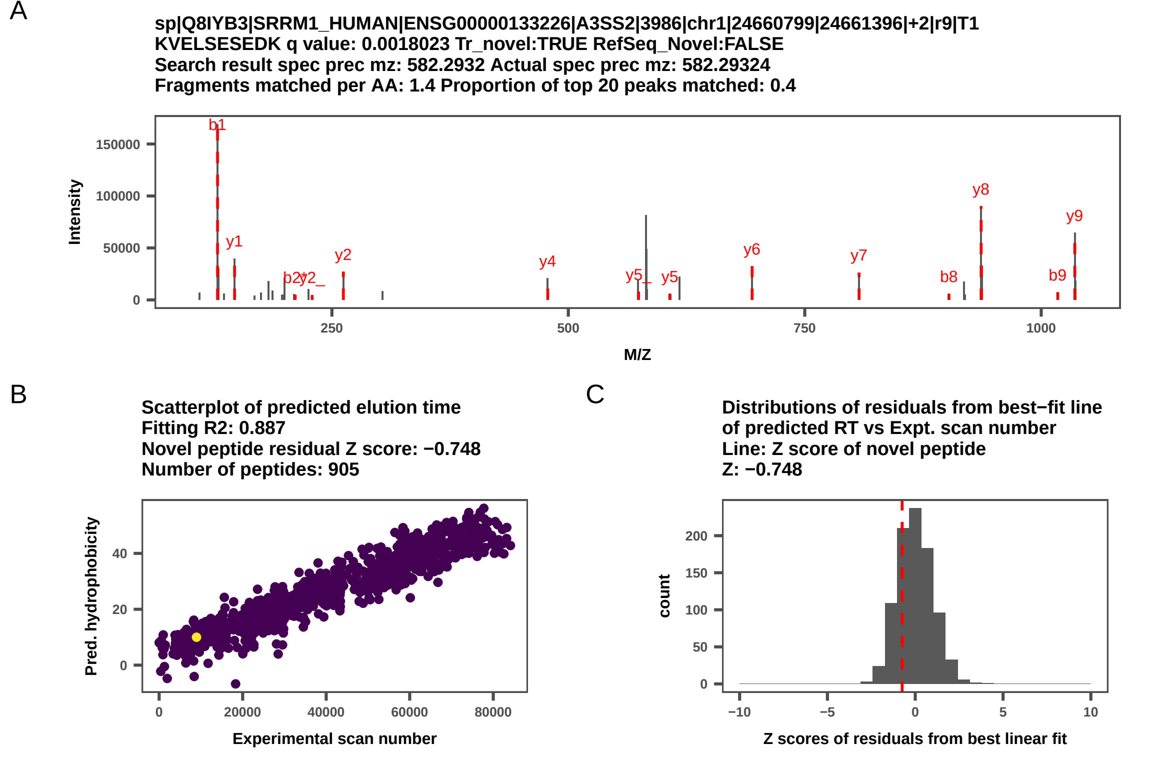
A
sp|Q8IYB3|SRRM1_HUMAN|ENSG00000133226|A3SS2|3986|chr1|24660799|24661396|+2|r9|T1
KVELSESEDK q value: 0.0018023 Tr_novel:TRUE RefSeq_Novel:FALSE
Search result spec prec mz: 582.2932 Actual spec prec mz: 582.29324
Fragments matched per AA: 1.4 Proportion of top 20 peaks matched: 0.4
Intensity
150000
100000
50000
0
b1
y1
b2
y2_
y2
y4
y5
y5_
y6
y7
b8
y8
b9
y9
250
500
750
1000
M/Z
B
Scatterplot of predicted elution time
Fitting R2: 0.887
Novel peptide residual Z score: −0.748
Number of peptides: 905
Pred. hydrophobicity
40
20
0
0
20000
40000
60000
80000
Experimental scan number
C
Distributions of residuals from best−fit line
of predicted RT vs Expt. scan number
Line: Z score of novel peptide
Z: −0.748
count
200
150
100
50
0
−10
−5
0
5
10
Z scores of residuals from best linear fit
