# Supplementary material for: Splice-Junction-Based Mapping of Alternative Isoforms in the Human Proteome
Source: Cell Rep. Author manuscript; Available in PMC 2020 Jan 15. (PMC6961840; doi:10.1016/j.celrep.2019.11.026)
Supplement: 2 [file NIHMS1546469-supplement-2.zip › DF1/PXD006675/LeftVentricle/LeftVentricle_31_RRNAD1_MPLGQALSVLETEDIFHR.pdf]

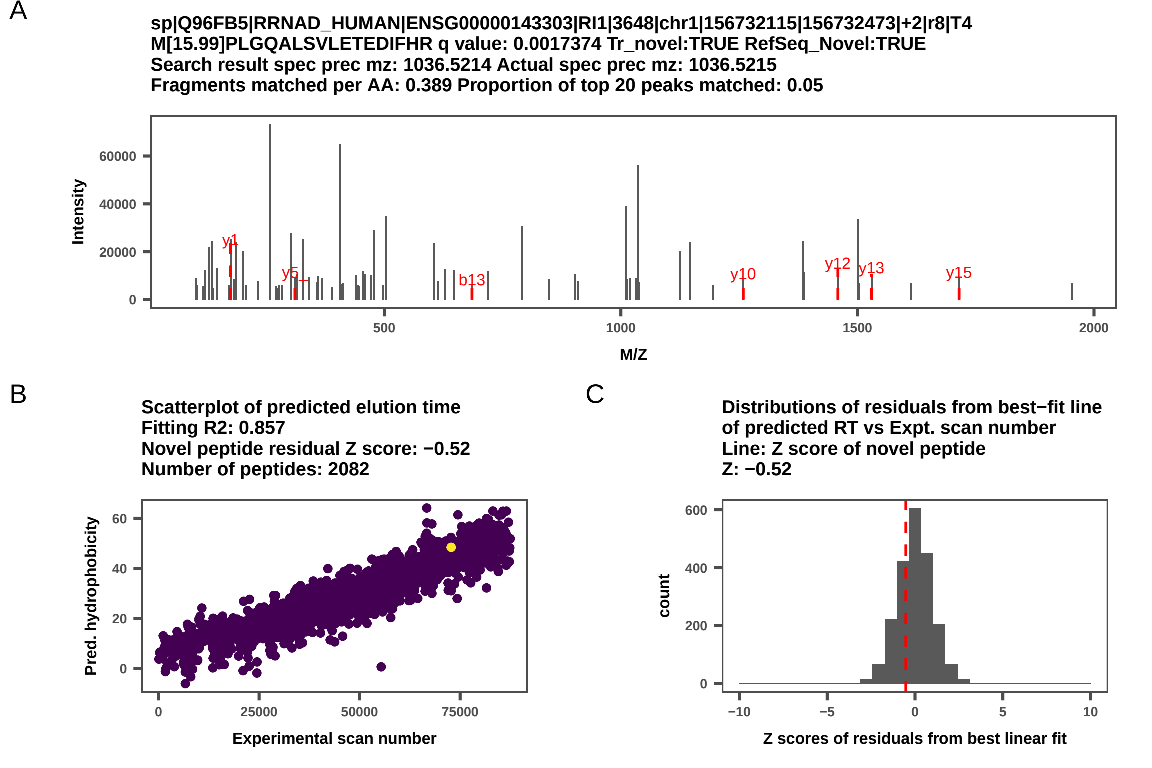
A
sp|Q96FB5|RRNAD_HUMAN|ENSG00000143303|RI1|3648|chr1|156732115|156732473|+2|r8|T4
M[15.99]PLGQALSVLETEDIFHR q value: 0.0017374 Tr_novel:TRUE RefSeq_Novel:TRUE
Search result spec prec mz: 1036.5214 Actual spec prec mz: 1036.5215
Fragments matched per AA: 0.389 Proportion of top 20 peaks matched: 0.05
Intensity
60000
40000
20000
0
y1
y5
b13
y10
y12
y13
y15
500
1000
1500
2000
M/Z
B
Scatterplot of predicted elution time
Fitting R2: 0.857
Novel peptide residual Z score: −0.52
Number of peptides: 2082
Pred. hydrophobicity
60
40
20
0
0
25000
50000
75000
Experimental scan number
C
Distributions of residuals from best−fit line
of predicted RT vs Expt. scan number
Line: Z score of novel peptide
Z: −0.52
count
600
400
200
0
−10
−5
0
5
10
Z scores of residuals from best linear fit
