# Supplementary material for: Splice-Junction-Based Mapping of Alternative Isoforms in the Human Proteome
Source: Cell Rep. Author manuscript; Available in PMC 2020 Jan 15. (PMC6961840; doi:10.1016/j.celrep.2019.11.026)
Supplement: 2 [file NIHMS1546469-supplement-2.zip › DF1/PXD006675/LeftVentricle/LeftVentricle_30_CYBA_KPSEEEAAVAAGGPPGGPQVNPIPVTDEVDR.pdf]

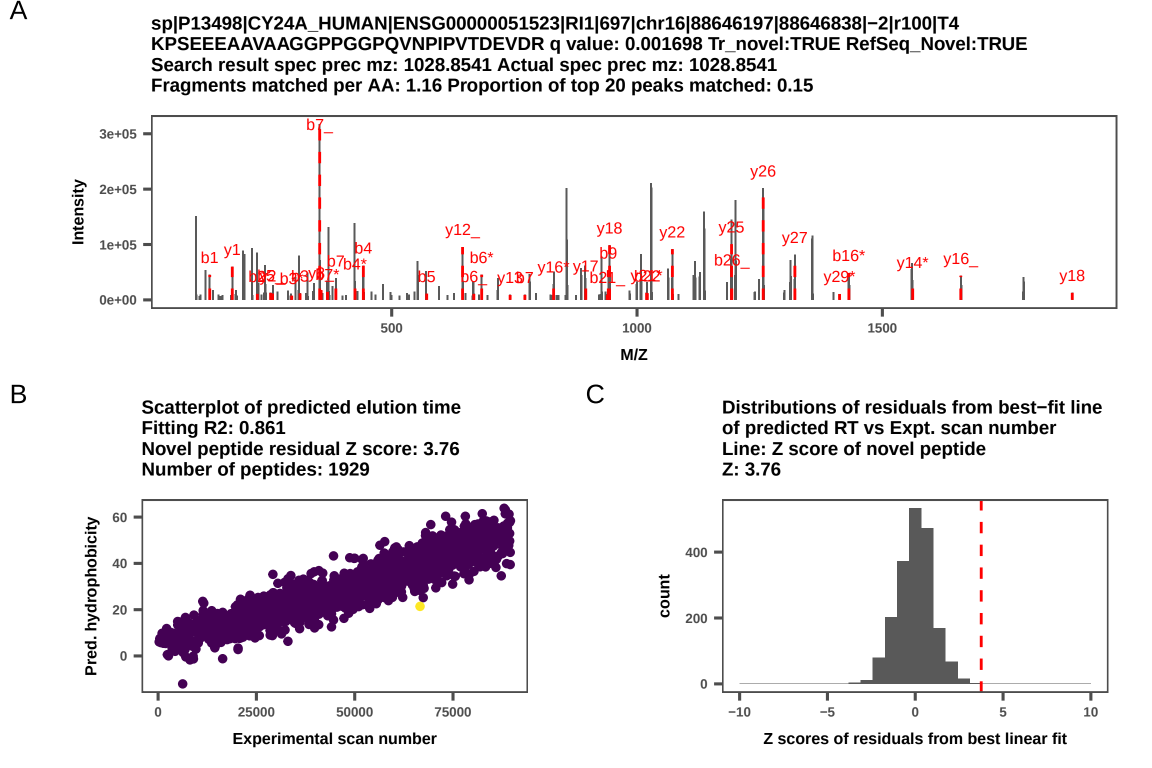
A
sp|P13498|CY24A_HUMAN|ENSG00000051523|RI1|697|chr16|88646197|88646838|−2|r100|T4
KPSEEEAAVAAGGPPGGPQVNPIPVTDEVDR q value: 0.001698 Tr_novel:TRUE RefSeq_Novel:TRUE
Search result spec prec mz: 1028.8541 Actual spec prec mz: 1028.8541
Fragments matched per AA: 1.16 Proportion of top 20 peaks matched: 0.15
Intensity
3e+05
2e+05
1e+05
0e+00
b7_
y26
y12_
y18
y22
y25
y27
b4
b1
y1
b6*
b9
b26_
b16*
y14*
y16_
y18
b5
y29*
y17
y16*
500
1000
1500
M/Z
B
Scatterplot of predicted elution time
Fitting R2: 0.861
Novel peptide residual Z score: 3.76
Number of peptides: 1929
Pred. hydrophobicity
60
40
20
0
0
25000
50000
75000
Experimental scan number
C
Distributions of residuals from best−fit line
of predicted RT vs Expt. scan number
Line: Z score of novel peptide
Z: 3.76
count
400
200
0
−10
−5
0
5
10
Z scores of residuals from best linear fit
