# Supplementary material for: Splice-Junction-Based Mapping of Alternative Isoforms in the Human Proteome
Source: Cell Rep. Author manuscript; Available in PMC 2020 Jan 15. (PMC6961840; doi:10.1016/j.celrep.2019.11.026)
Supplement: 2 [file NIHMS1546469-supplement-2.zip › DF1/PXD006675/LeftVentricle/LeftVentricle_41_TTN_APHVEFLRPLTD.pdf]

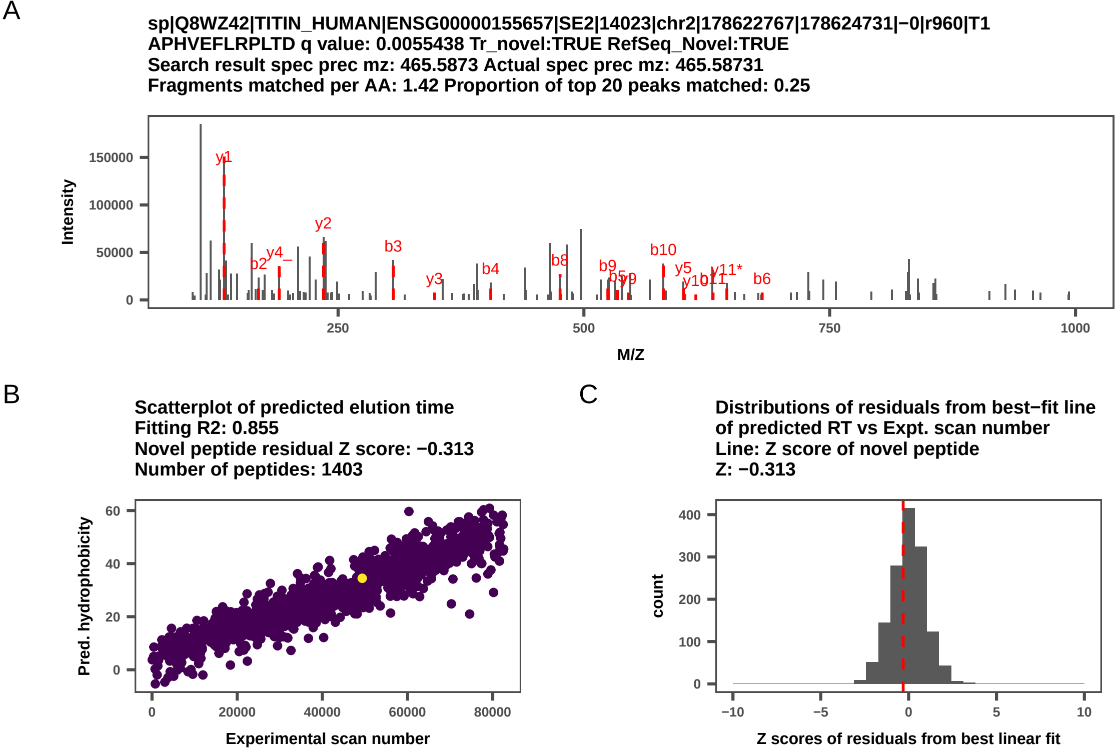
A
sp|Q8WZ42|TITIN_HUMAN|ENSG00000155657|SE2|14023|chr2|178622767|178624731|−0|r960|T1
APHVEFLRPLTD q value: 0.0055438 Tr_novel:TRUE RefSeq_Novel:TRUE
Search result spec prec mz: 465.5873 Actual spec prec mz: 465.58731
Fragments matched per AA: 1.42 Proportion of top 20 peaks matched: 0.25
Intensity
150000
100000
50000
0
y1
y2
b2
y4_
b3
y3
b4
b8
b9
b5
y9
b10
y5
y10
b11
y11*
b6
250
500
750
1000
M/Z
B
Scatterplot of predicted elution time
Fitting R2: 0.855
Novel peptide residual Z score: −0.313
Number of peptides: 1403
Pred. hydrophobicity
60
40
20
0
0
20000
40000
60000
80000
Experimental scan number
C
Distributions of residuals from best−fit line
of predicted RT vs Expt. scan number
Line: Z score of novel peptide
Z: −0.313
count
400
300
200
100
0
−10
−5
0
5
10
Z scores of residuals from best linear fit
