# Supplementary material for: Splice-Junction-Based Mapping of Alternative Isoforms in the Human Proteome
Source: Cell Rep. Author manuscript; Available in PMC 2020 Jan 15. (PMC6961840; doi:10.1016/j.celrep.2019.11.026)
Supplement: 2 [file NIHMS1546469-supplement-2.zip › DF1/PXD006675/LeftVentricle/LeftVentricle_23_TNS1_PHNPADILLHPTGEEDEGK.pdf]

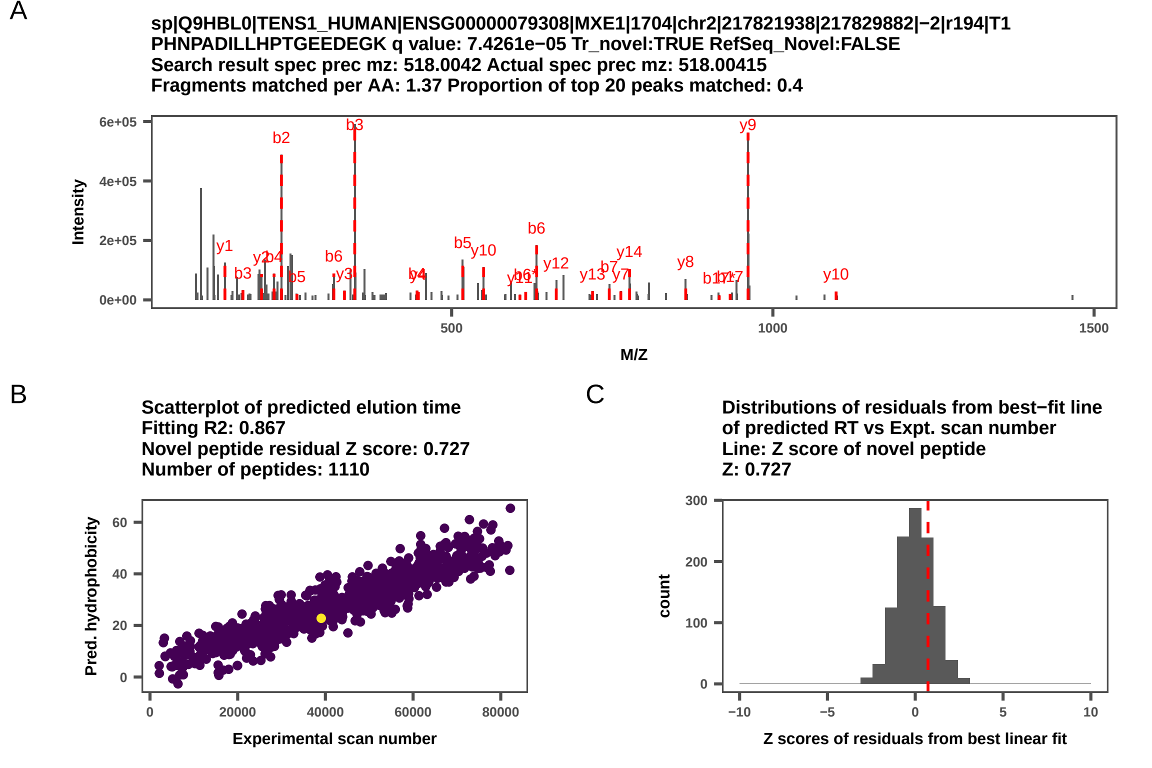
A
sp|Q9HBL0|TENS1_HUMAN|ENSG00000079308|MXE1|1704|chr2|217821938|217829882|−2|r194|T1
PHNPADILLHPTGEEDEGK q value: 7.4261e−05 Tr_novel:TRUE RefSeq_Novel:FALSE
Search result spec prec mz: 518.0042 Actual spec prec mz: 518.00415
Fragments matched per AA: 1.37 Proportion of top 20 peaks matched: 0.4
Intensity
M/Z
6e+05
4e+05
2e+05
0e+00
500
1000
1500
b2
b3
y9
b6
y10
B
Scatterplot of predicted elution time
Fitting R2: 0.867
Novel peptide residual Z score: 0.727
Number of peptides: 1110
Pred. hydrophobicity
Experimental scan number
0
20000
40000
60000
80000
20
40
60
C
Distributions of residuals from best−fit line
of predicted RT vs Expt. scan number
Line: Z score of novel peptide
Z: 0.727
count
Z scores of residuals from best linear fit
300
200
100
−10
−5
5
10
