# Supplementary material for: Splice-Junction-Based Mapping of Alternative Isoforms in the Human Proteome
Source: Cell Rep. Author manuscript; Available in PMC 2020 Jan 15. (PMC6961840; doi:10.1016/j.celrep.2019.11.026)
Supplement: 2 [file NIHMS1546469-supplement-2.zip › DF1/PXD006675/LeftVentricle/LeftVentricle_37_MAP4_KKPCSETSQIEGSPTEFLEEK.pdf]

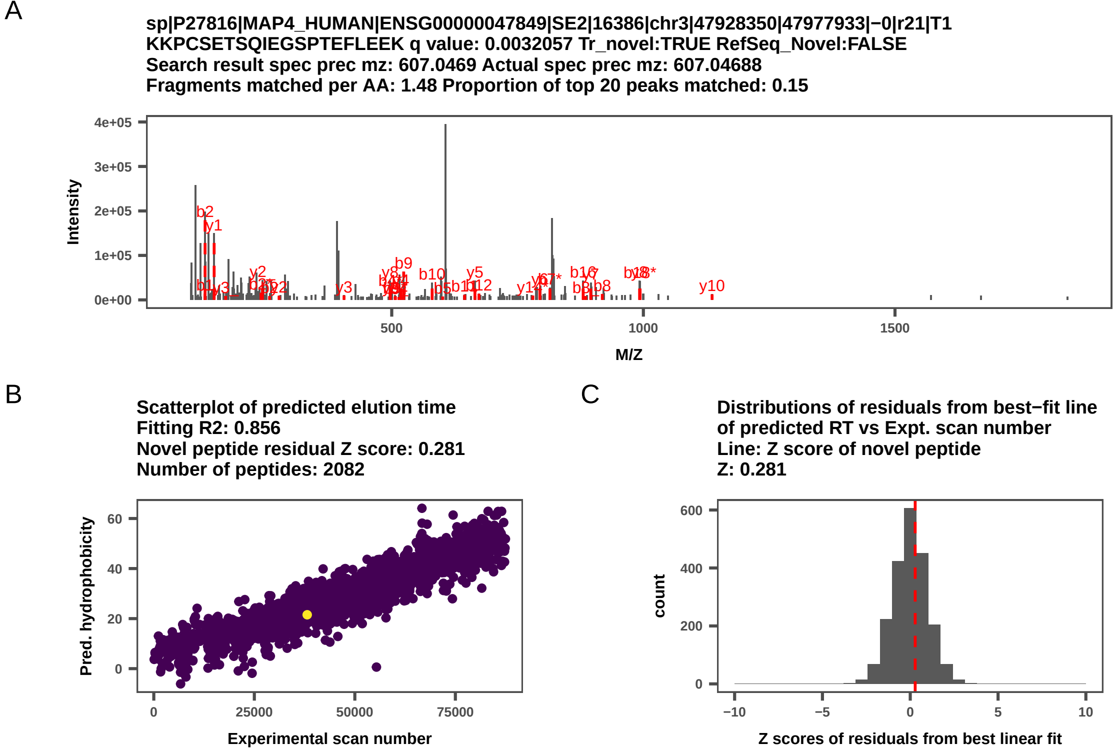
A
sp|P27816|MAP4_HUMAN|ENSG00000047849|SE2|16386|chr3|47928350|47977933|−0|r21|T1
KKPCSETSQIEGSPTEFLEEK q value: 0.0032057 Tr_novel:TRUE RefSeq_Novel:FALSE
Search result spec prec mz: 607.0469 Actual spec prec mz: 607.04688
Fragments matched per AA: 1.48 Proportion of top 20 peaks matched: 0.15
Intensity
4e+05
3e+05
2e+05
1e+05
0e+00
b2
y1
y2
b9
b10
y5
y10
M/Z
500
1000
1500
B
Scatterplot of predicted elution time
Fitting R2: 0.856
Novel peptide residual Z score: 0.281
Number of peptides: 2082
Pred. hydrophobicity
60
40
20
0
0
25000
50000
75000
Experimental scan number
C
Distributions of residuals from best−fit line
of predicted RT vs Expt. scan number
Line: Z score of novel peptide
Z: 0.281
count
600
400
200
0
−10
−5
0
5
10
Z scores of residuals from best linear fit
