# Supplementary material for: Splice-Junction-Based Mapping of Alternative Isoforms in the Human Proteome
Source: Cell Rep. Author manuscript; Available in PMC 2020 Jan 15. (PMC6961840; doi:10.1016/j.celrep.2019.11.026)
Supplement: 2 [file NIHMS1546469-supplement-2.zip › DF1/PXD006675/LeftVentricle/LeftVentricle_55_TOGARAM1_NLVQQDFLLQR.pdf]

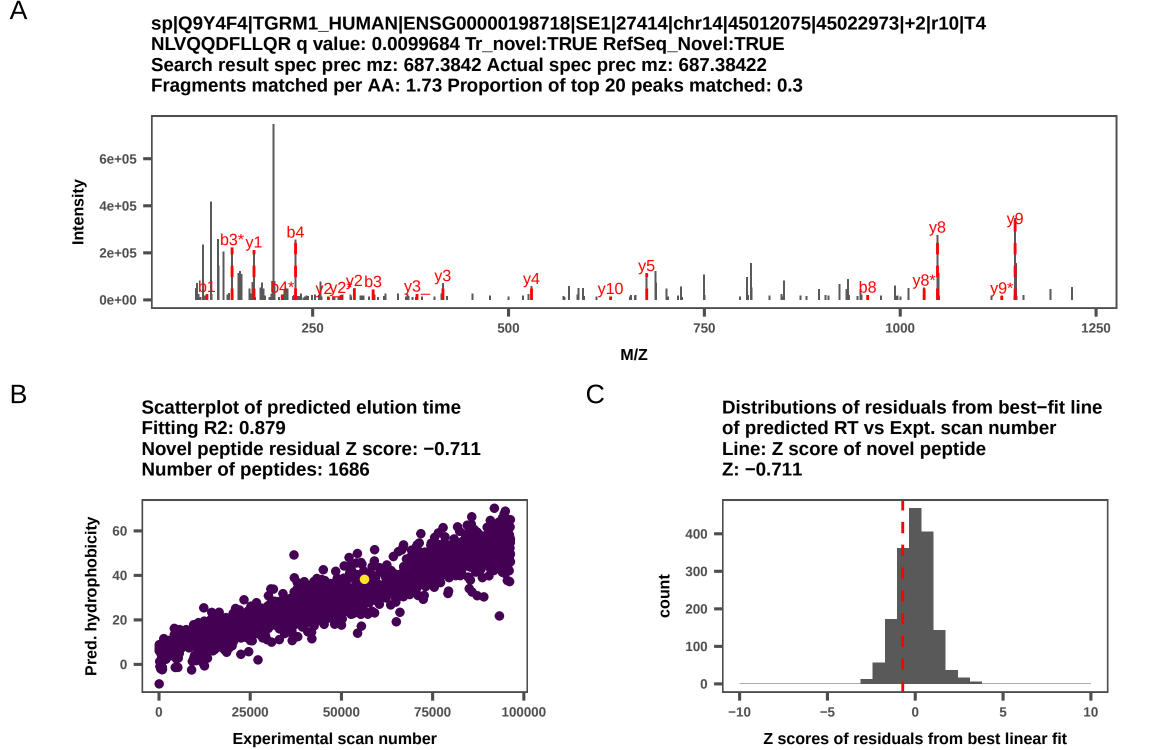
A
sp|Q9Y4F4|TGRM1_HUMAN|ENSG00000198718|SE1|27414|chr14|45012075|45022973|+2|r10|T4
NLVQQDFLLQR q value: 0.0099684 Tr_novel:TRUE RefSeq_Novel:TRUE
Search result spec prec mz: 687.3842 Actual spec prec mz: 687.38422
Fragments matched per AA: 1.73 Proportion of top 20 peaks matched: 0.3
Intensity
6e+05
4e+05
2e+05
0e+00
b1
b3*
y1
b4
b4*
y2
y2*
y2
b3
y3
y3
y4
y10
y5
b8
y8*
y8
y9*
y9
250
500
750
1000
1250
M/Z
B
Scatterplot of predicted elution time
Fitting R2: 0.879
Novel peptide residual Z score: −0.711
Number of peptides: 1686
Pred. hydrophobicity
60
40
20
0
0
25000
50000
75000
100000
Experimental scan number
C
Distributions of residuals from best−fit line
of predicted RT vs Expt. scan number
Line: Z score of novel peptide
Z: −0.711
count
400
300
200
100
0
−10
−5
0
5
10
Z scores of residuals from best linear fit
