# Supplementary material for: Splice-Junction-Based Mapping of Alternative Isoforms in the Human Proteome
Source: Cell Rep. Author manuscript; Available in PMC 2020 Jan 15. (PMC6961840; doi:10.1016/j.celrep.2019.11.026)
Supplement: 2 [file NIHMS1546469-supplement-2.zip › DF1/PXD006675/LeftVentricle/LeftVentricle_20_RYR2_VLDIANVLFHLEQVEHPQR.pdf]

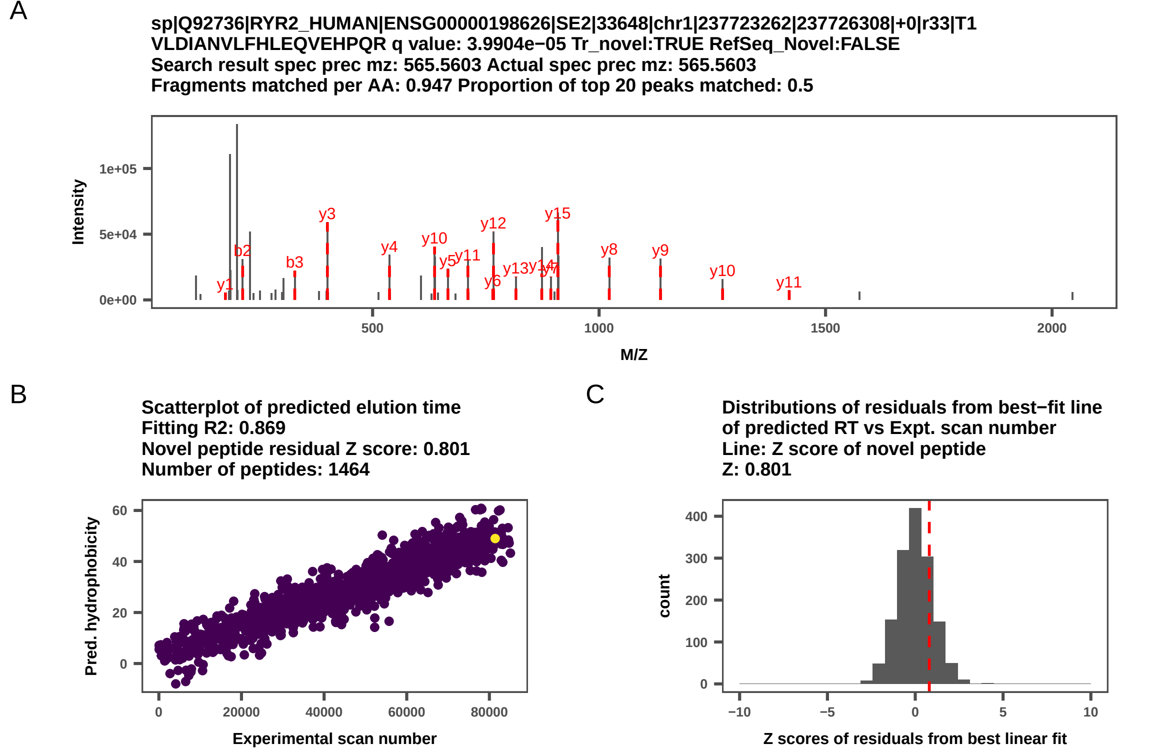
A
sp|Q92736|RYR2_HUMAN|ENSG00000198626|SE2|33648|chr1|237723262|237726308|+0|r33|T1
VLDIANVLFHLEQVEHPQR q value: 3.9904e−05 Tr_novel:TRUE RefSeq_Novel:FALSE
Search result spec prec mz: 565.5603 Actual spec prec mz: 565.5603
Fragments matched per AA: 0.947 Proportion of top 20 peaks matched: 0.5
Intensity
1e+05
5e+04
0e+00
y1
b2
b3
y3
y4
y10
y5
y11
y12
y6
y13
y14
y7
y15
y8
y9
y10
y11
500
1000
1500
2000
M/Z
B
Scatterplot of predicted elution time
Fitting R2: 0.869
Novel peptide residual Z score: 0.801
Number of peptides: 1464
Pred. hydrophobicity
60
40
20
0
0
20000
40000
60000
80000
Experimental scan number
C
Distributions of residuals from best−fit line
of predicted RT vs Expt. scan number
Line: Z score of novel peptide
Z: 0.801
count
400
300
200
100
0
−10
−5
0
5
10
Z scores of residuals from best linear fit
