# Supplementary material for: Splice-Junction-Based Mapping of Alternative Isoforms in the Human Proteome
Source: Cell Rep. Author manuscript; Available in PMC 2020 Jan 15. (PMC6961840; doi:10.1016/j.celrep.2019.11.026)
Supplement: 2 [file NIHMS1546469-supplement-2.zip › DF1/PXD006675/LeftVentricle/LeftVentricle_28_WDR47_NELEFTMQEAVQCLHALEEYCPSKDDYSK.pdf]

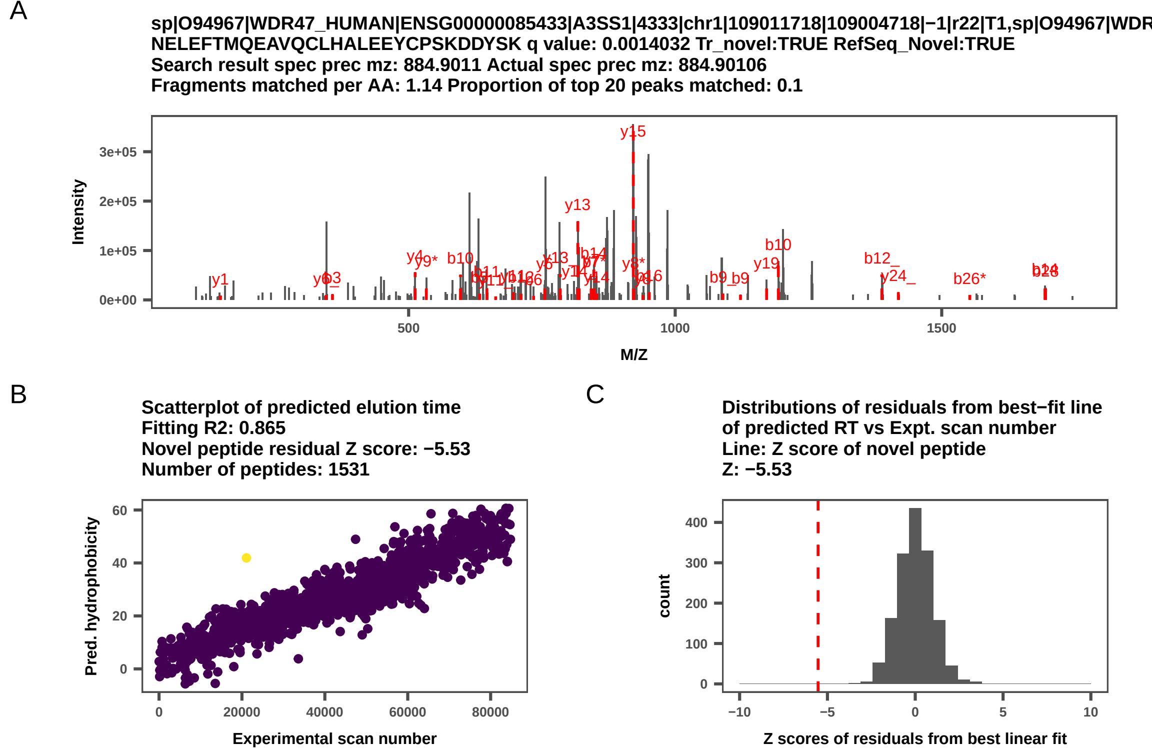
A
sp|O94967|WDR47_HUMAN|ENSG00000085433|A3SS1|4333|chr1|109011718|109004718|−1|r22|T1,sp|O94967|WDR
NELEFTMQEAVQCLHALEEYCPSKDDYSK q value: 0.0014032 Tr_novel:TRUE RefSeq_Novel:TRUE
Search result spec prec mz: 884.9011 Actual spec prec mz: 884.90106
Fragments matched per AA: 1.14 Proportion of top 20 peaks matched: 0.1
Intensity
3e+05
2e+05
1e+05
0e+00
y15
y13
b10
y19
b12_
y24_
b26*
y1
M/Z
500
1000
1500
B
Scatterplot of predicted elution time
Fitting R2: 0.865
Novel peptide residual Z score: −5.53
Number of peptides: 1531
Pred. hydrophobicity
60
40
20
0
0
20000
40000
60000
80000
Experimental scan number
C
Distributions of residuals from best−fit line
of predicted RT vs Expt. scan number
Line: Z score of novel peptide
Z: −5.53
count
400
300
200
100
0
−10
−5
0
5
10
Z scores of residuals from best linear fit
