# Supplementary material for: Splice-Junction-Based Mapping of Alternative Isoforms in the Human Proteome
Source: Cell Rep. Author manuscript; Available in PMC 2020 Jan 15. (PMC6961840; doi:10.1016/j.celrep.2019.11.026)
Supplement: 2 [file NIHMS1546469-supplement-2.zip › DF1/PXD006675/LeftVentricle/LeftVentricle_36_SMTN_LFLLIQEDGQMAVAAPDEGALR.pdf]

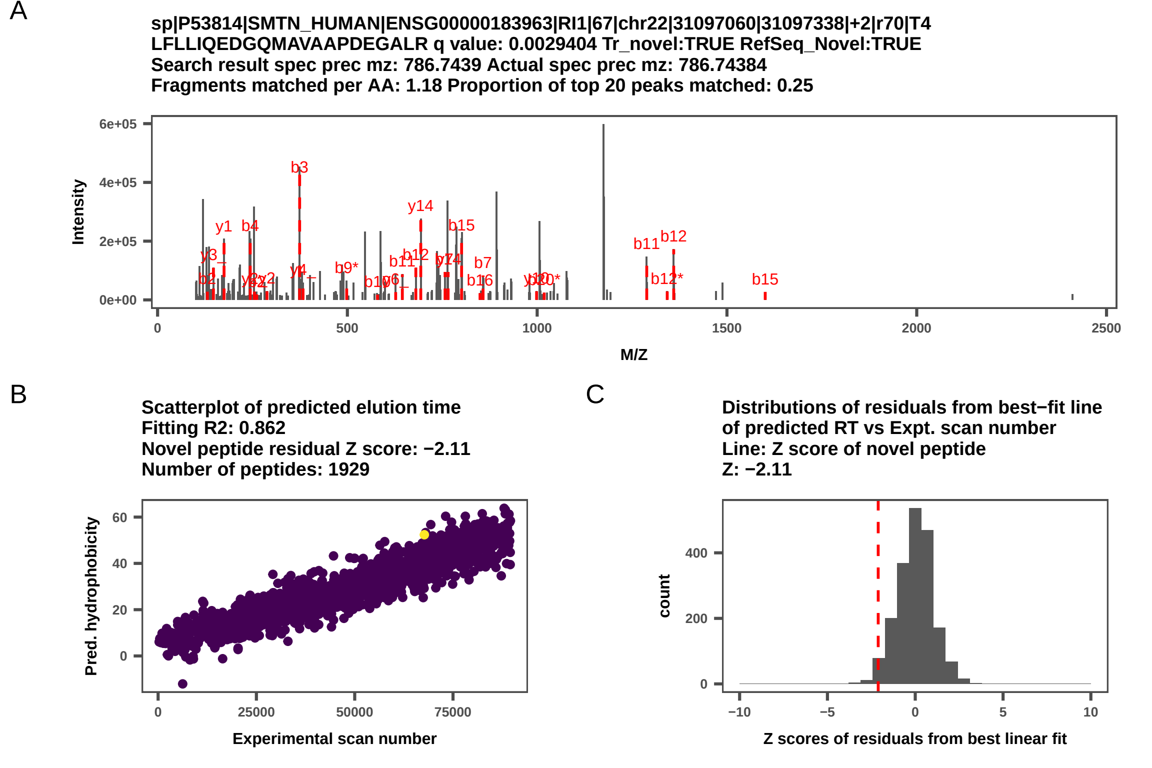
A
sp|P53814|SMTN_HUMAN|ENSG00000183963|RI1|67|chr22|31097060|31097338|+2|r70|T4
LFLLIQEDGQMAVAAPDEGALR q value: 0.0029404 Tr_novel:TRUE RefSeq_Novel:TRUE
Search result spec prec mz: 786.7439 Actual spec prec mz: 786.74384
Fragments matched per AA: 1.18 Proportion of top 20 peaks matched: 0.25
Intensity
6e+05
4e+05
2e+05
0e+00
b3
y14
y1
b4
b15
b11
b12
y3
b9*
b7
b2
b16
b12*
b15
M/Z
0
500
1000
1500
2000
2500
B
Scatterplot of predicted elution time
Fitting R2: 0.862
Novel peptide residual Z score: −2.11
Number of peptides: 1929
Pred. hydrophobicity
60
40
20
0
0
25000
50000
75000
Experimental scan number
C
Distributions of residuals from best−fit line
of predicted RT vs Expt. scan number
Line: Z score of novel peptide
Z: −2.11
count
400
200
0
−10
−5
0
5
10
Z scores of residuals from best linear fit
