# Supplementary material for: Splice-Junction-Based Mapping of Alternative Isoforms in the Human Proteome
Source: Cell Rep. Author manuscript; Available in PMC 2020 Jan 15. (PMC6961840; doi:10.1016/j.celrep.2019.11.026)
Supplement: 2 [file NIHMS1546469-supplement-2.zip › DF1/PXD006675/LeftVentricle/LeftVentricle_6_TTN_TPVQEEVIEVKVPEVPK.pdf]

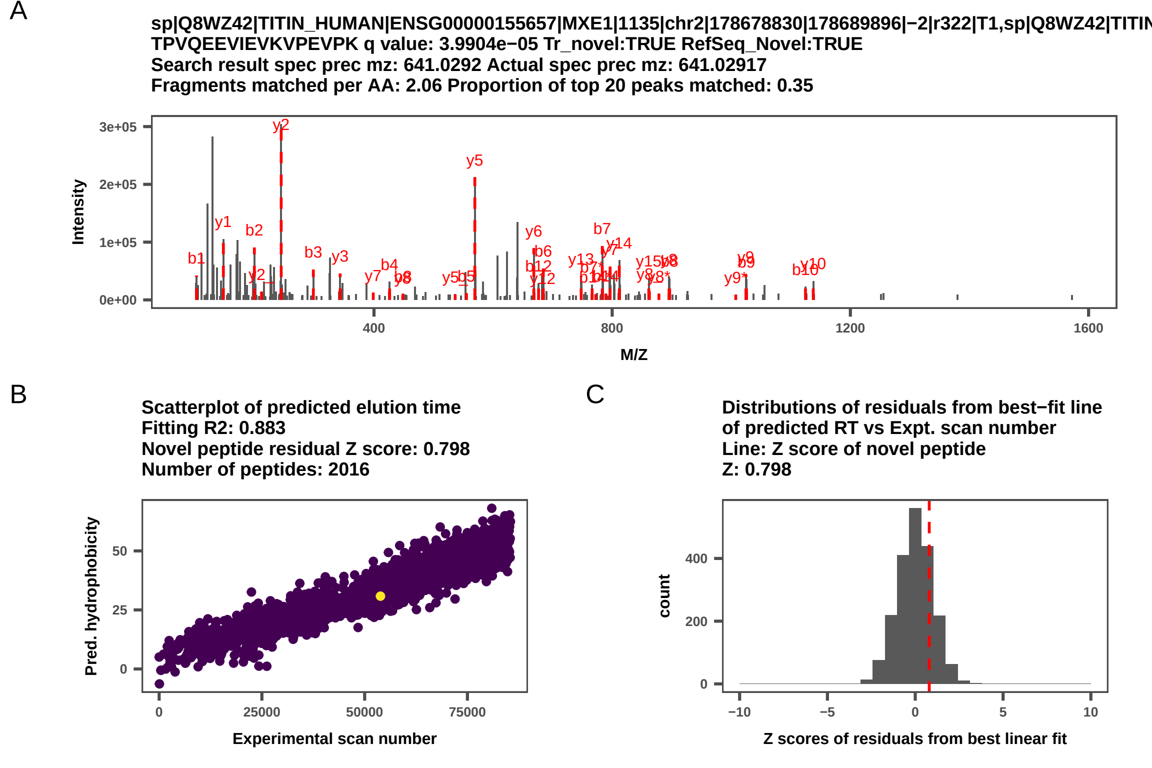

A
sp|Q8WZ42|TITIN_HUMAN|ENSG00000155657|MXE1|1135|chr2|178678830|178689896|−2|r322|T1,sp|Q8WZ42|TITIN
TPVQEEVIEVKVPEVPK q value: 3.9904e−05 Tr_novel:TRUE RefSeq_Novel:TRUE
Search result spec prec mz: 641.0292 Actual spec prec mz: 641.02917
Fragments matched per AA: 2.06 Proportion of top 20 peaks matched: 0.35
Intensity
3e+05
2e+05
1e+05
0e+00
y2
y5
y1
b2
b1
b3
y3
b4
y7
y6
b7
y14
b6
b10
y10
M/Z
400
800
1200
1600
B
Scatterplot of predicted elution time
Fitting R2: 0.883
Novel peptide residual Z score: 0.798
Number of peptides: 2016
Pred. hydrophobicity
50
25
0
0
25000
50000
75000
Experimental scan number
C
Distributions of residuals from best−fit line
of predicted RT vs Expt. scan number
Line: Z score of novel peptide
Z: 0.798
count
400
200
0
−10
−5
0
5
10
Z scores of residuals from best linear fit
