# Supplementary material for: Splice-Junction-Based Mapping of Alternative Isoforms in the Human Proteome
Source: Cell Rep. Author manuscript; Available in PMC 2020 Jan 15. (PMC6961840; doi:10.1016/j.celrep.2019.11.026)
Supplement: 2 [file NIHMS1546469-supplement-2.zip › DF1/PXD006675/LeftVentricle/LeftVentricle_17_TTN_RVEAEPAEEVTIMEEK.pdf]

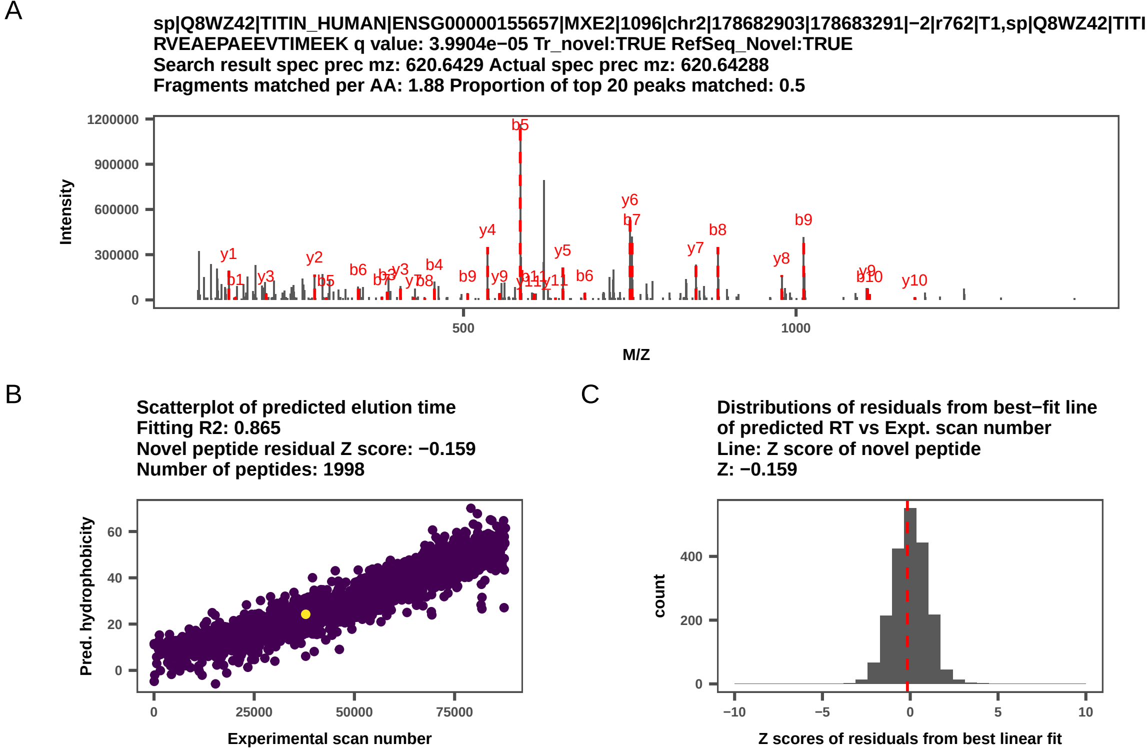
A
sp|Q8WZ42|TITIN_HUMAN|ENSG00000155657|MXE2|1096|chr2|178682903|178683291|−2|r762|T1,sp|Q8WZ42|TITI
RVEAEPAEEVTIMEEK q value: 3.9904e−05 Tr_novel:TRUE RefSeq_Novel:TRUE
Search result spec prec mz: 620.6429 Actual spec prec mz: 620.64288
Fragments matched per AA: 1.88 Proportion of top 20 peaks matched: 0.5
Intensity
1200000
900000
600000
300000
0
500
1000
M/Z
b5
y6
b7
y4
b9
b8
y7
y5
y8
y1
y2
b6
y3
b4
b1
y3
b5
b9
y9
b6
y9
b10
y10
B
Scatterplot of predicted elution time
Fitting R2: 0.865
Novel peptide residual Z score: −0.159
Number of peptides: 1998
Pred. hydrophobicity
60
40
20
0
0
25000
50000
75000
Experimental scan number
C
Distributions of residuals from best−fit line
of predicted RT vs Expt. scan number
Line: Z score of novel peptide
Z: −0.159
count
400
200
0
−10
−5
0
5
10
Z scores of residuals from best linear fit
