# Supplementary material for: Splice-Junction-Based Mapping of Alternative Isoforms in the Human Proteome
Source: Cell Rep. Author manuscript; Available in PMC 2020 Jan 15. (PMC6961840; doi:10.1016/j.celrep.2019.11.026)
Supplement: 2 [file NIHMS1546469-supplement-2.zip › DF1/PXD006675/LeftVentricle/LeftVentricle_7_PPP1R1A_VEHHLGQQQQGEEPEGAAESTGTQESRPPGIPDTEVESR.pdf]

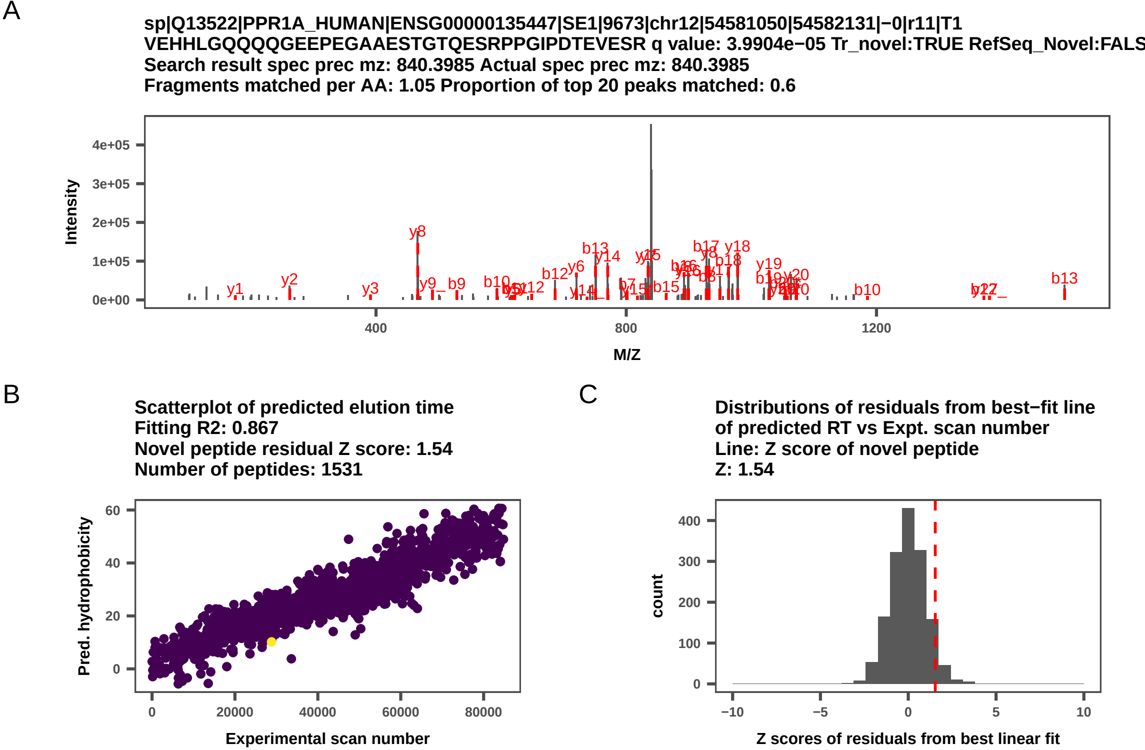
A
sp|Q13522|PPR1A_HUMAN|ENSG00000135447|SE1|9673|chr12|54581050|54582131|−0|r11|T1
VEHHLGQQQQGEEPEGAAESTGTQESRPPGIPDTEVESR q value: 3.9904e−05 Tr_novel:TRUE RefSeq_Novel:FALS
Search result spec prec mz: 840.3985 Actual spec prec mz: 840.3985
Fragments matched per AA: 1.05 Proportion of top 20 peaks matched: 0.6
Intensity
4e+05
3e+05
2e+05
1e+05
0e+00
M/Z
400
800
1200
y1
y2
y3
y8
y9
b9
b10
b12
y6
b13
y14
b15
b17
y18
y19
y20
b10
b13
B
Scatterplot of predicted elution time
Fitting R2: 0.867
Novel peptide residual Z score: 1.54
Number of peptides: 1531
Pred. hydrophobicity
60
40
20
0
0
20000
40000
60000
80000
Experimental scan number
C
Distributions of residuals from best−fit line
of predicted RT vs Expt. scan number
Line: Z score of novel peptide
Z: 1.54
count
400
300
200
100
0
−10
−5
0
5
10
Z scores of residuals from best linear fit
