# Supplementary material for: Splice-Junction-Based Mapping of Alternative Isoforms in the Human Proteome
Source: Cell Rep. Author manuscript; Available in PMC 2020 Jan 15. (PMC6961840; doi:10.1016/j.celrep.2019.11.026)
Supplement: 2 [file NIHMS1546469-supplement-2.zip › DF1/PXD006675/LeftVentricle/LeftVentricle_11_MYBPC3_RRTDSHEDTGILDFSSLLK.pdf]

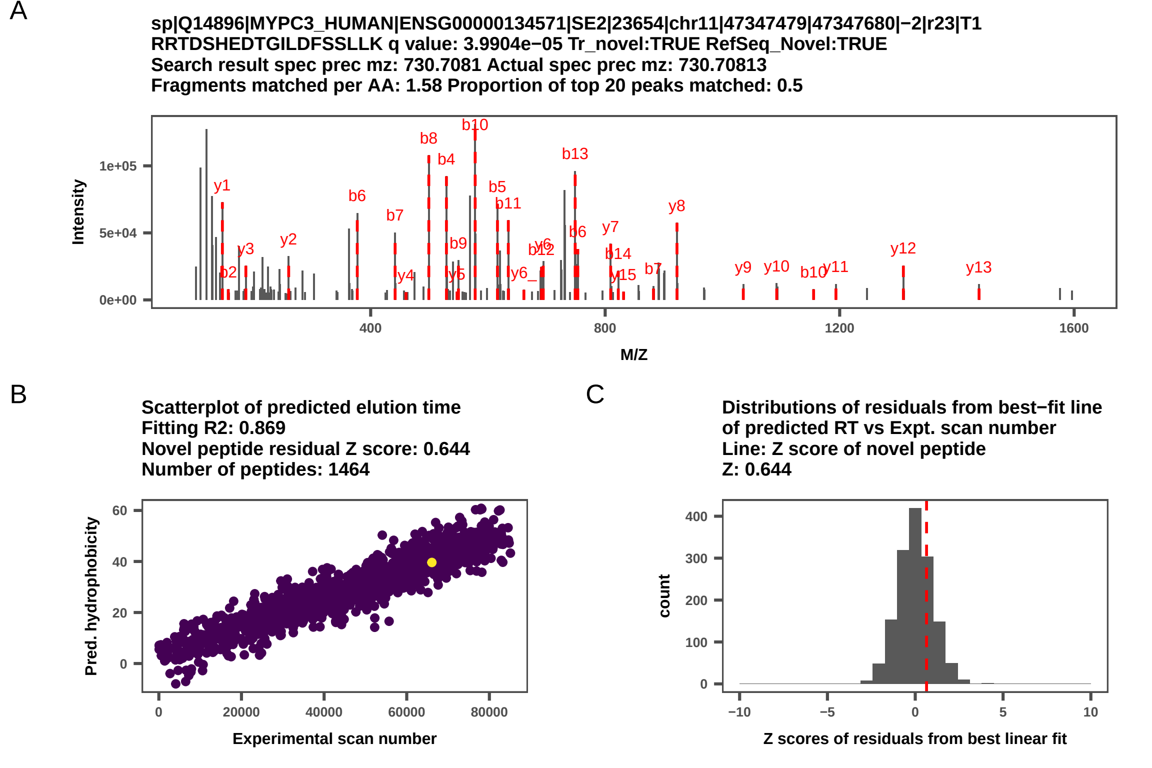
A
sp|Q14896|MYPC3_HUMAN|ENSG00000134571|SE2|23654|chr11|47347479|47347680|−2|r23|T1
RRTDSHEDTGILDFSSLLK q value: 3.9904e−05 Tr_novel:TRUE RefSeq_Novel:TRUE
Search result spec prec mz: 730.7081 Actual spec prec mz: 730.70813
Fragments matched per AA: 1.58 Proportion of top 20 peaks matched: 0.5
Intensity
1e+05
5e+04
0e+00
M/Z
400
800
1200
1600
y1 y2 y3 y4 y5 y6 y7 y8 y9 y10 y11 y12 y13 y15
b2 b4 b5 b6 b7 b8 b9 b10 b11 b12 b13 b14
B
Scatterplot of predicted elution time
Fitting R2: 0.869
Novel peptide residual Z score: 0.644
Number of peptides: 1464
Pred. hydrophobicity
0
20
40
60
Experimental scan number
0
20000
40000
60000
80000
C
Distributions of residuals from best−fit line
of predicted RT vs Expt. scan number
Line: Z score of novel peptide
Z: 0.644
count
0
100
200
300
400
Z scores of residuals from best linear fit
−10
−5
0
5
10
