# Supplementary material for: Splice-Junction-Based Mapping of Alternative Isoforms in the Human Proteome
Source: Cell Rep. Author manuscript; Available in PMC 2020 Jan 15. (PMC6961840; doi:10.1016/j.celrep.2019.11.026)
Supplement: 2 [file NIHMS1546469-supplement-2.zip › DF1/PXD006675/LeftVentricle/LeftVentricle_47_TPM3_AISEELDHALNDMTSG.pdf]

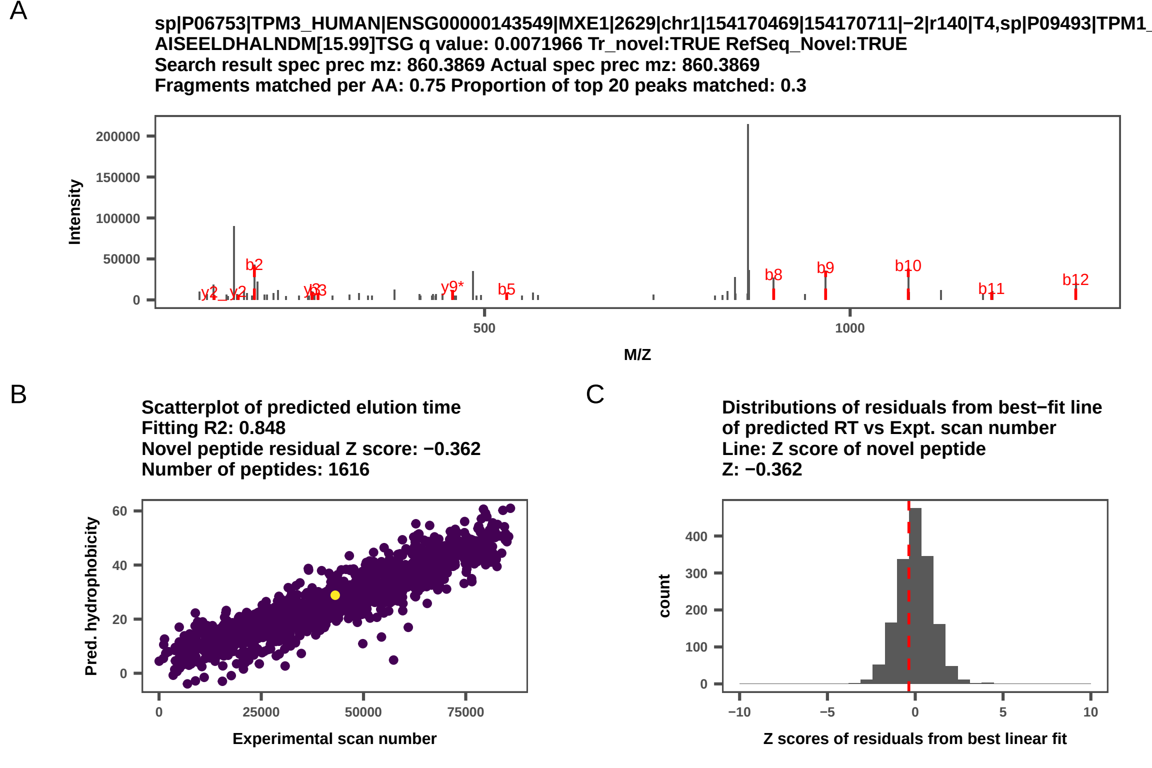
A
sp|P06753|TPM3_HUMAN|ENSG00000143549|MXE1|2629|chr1|154170469|154170711|−2|r140|T4,sp|P09493|TPM1_
AISEELDHALNDM[15.99]TSG q value: 0.0071966 Tr_novel:TRUE RefSeq_Novel:TRUE
Search result spec prec mz: 860.3869 Actual spec prec mz: 860.3869
Fragments matched per AA: 0.75 Proportion of top 20 peaks matched: 0.3
Intensity
200000
150000
100000
50000
0
y2_y2
b2
y3
b3
y9*
b5
b8
b9
b10
b11
b12
500
1000
M/Z
B
Scatterplot of predicted elution time
Fitting R2: 0.848
Novel peptide residual Z score: −0.362
Number of peptides: 1616
Pred. hydrophobicity
60
40
20
0
0
25000
50000
75000
Experimental scan number
C
Distributions of residuals from best−fit line
of predicted RT vs Expt. scan number
Line: Z score of novel peptide
Z: −0.362
count
400
300
200
100
0
−10
−5
0
5
10
Z scores of residuals from best linear fit
