# Supplementary material for: Splice-Junction-Based Mapping of Alternative Isoforms in the Human Proteome
Source: Cell Rep. Author manuscript; Available in PMC 2020 Jan 15. (PMC6961840; doi:10.1016/j.celrep.2019.11.026)
Supplement: 2 [file NIHMS1546469-supplement-2.zip › DF1/PXD006675/LeftVentricle/LeftVentricle_12_PDLIM5_ANLSSSTGNVEDSFEGFR.pdf]

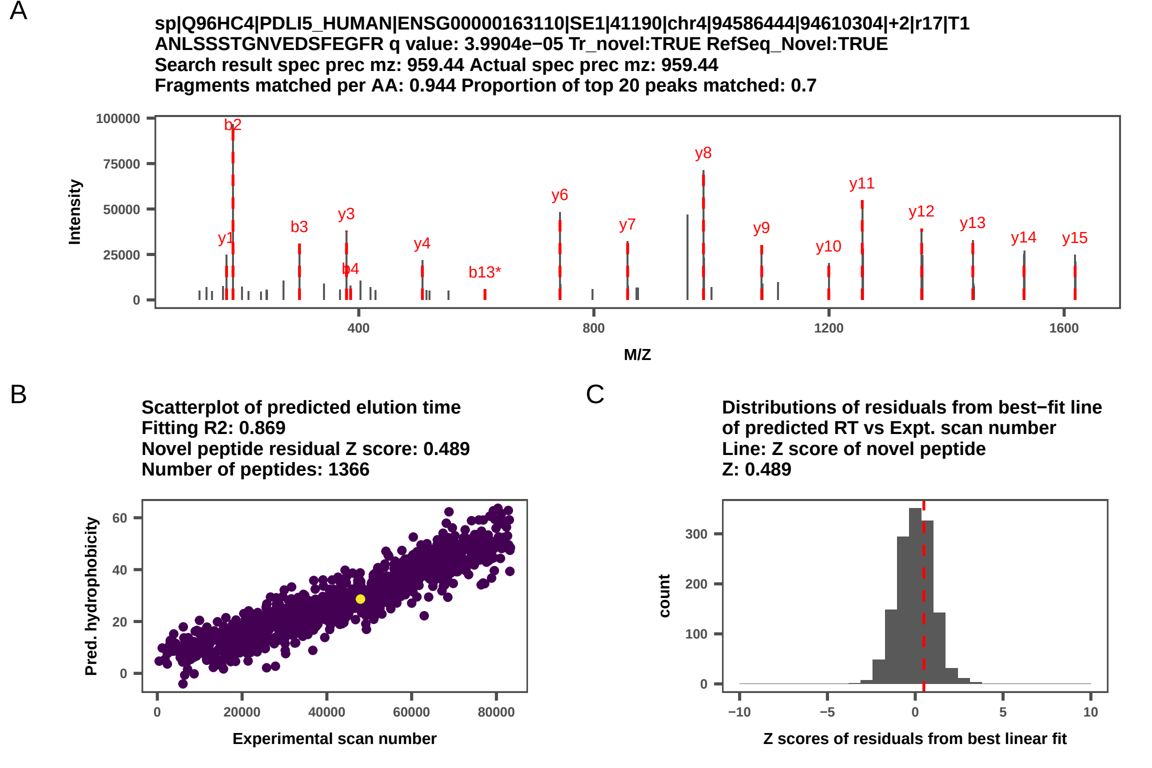
A
sp|Q96HC4|PDLI5_HUMAN|ENSG00000163110|SE1|41190|chr4|94586444|94610304|+2|r17|T1
ANLSSSTGNVEDSFEGFR q value: 3.9904e−05 Tr_novel:TRUE RefSeq_Novel:TRUE
Search result spec prec mz: 959.44 Actual spec prec mz: 959.44
Fragments matched per AA: 0.944 Proportion of top 20 peaks matched: 0.7
Intensity
M/Z
b2
y1
b3
y3
b4
y4
b13*
y6
y7
y8
y9
y10
y11
y12
y13
y14
y15
B
Scatterplot of predicted elution time
Fitting R2: 0.869
Novel peptide residual Z score: 0.489
Number of peptides: 1366
Pred. hydrophobicity
Experimental scan number
C
Distributions of residuals from best−fit line
of predicted RT vs Expt. scan number
Line: Z score of novel peptide
Z: 0.489
count
Z scores of residuals from best linear fit
