# Supplementary material for: Splice-Junction-Based Mapping of Alternative Isoforms in the Human Proteome
Source: Cell Rep. Author manuscript; Available in PMC 2020 Jan 15. (PMC6961840; doi:10.1016/j.celrep.2019.11.026)
Supplement: 2 [file NIHMS1546469-supplement-2.zip › DF1/PXD006675/LeftVentricle/LeftVentricle_48_ADCK1_VSWTSMALAASGIYFYSNK.pdf]

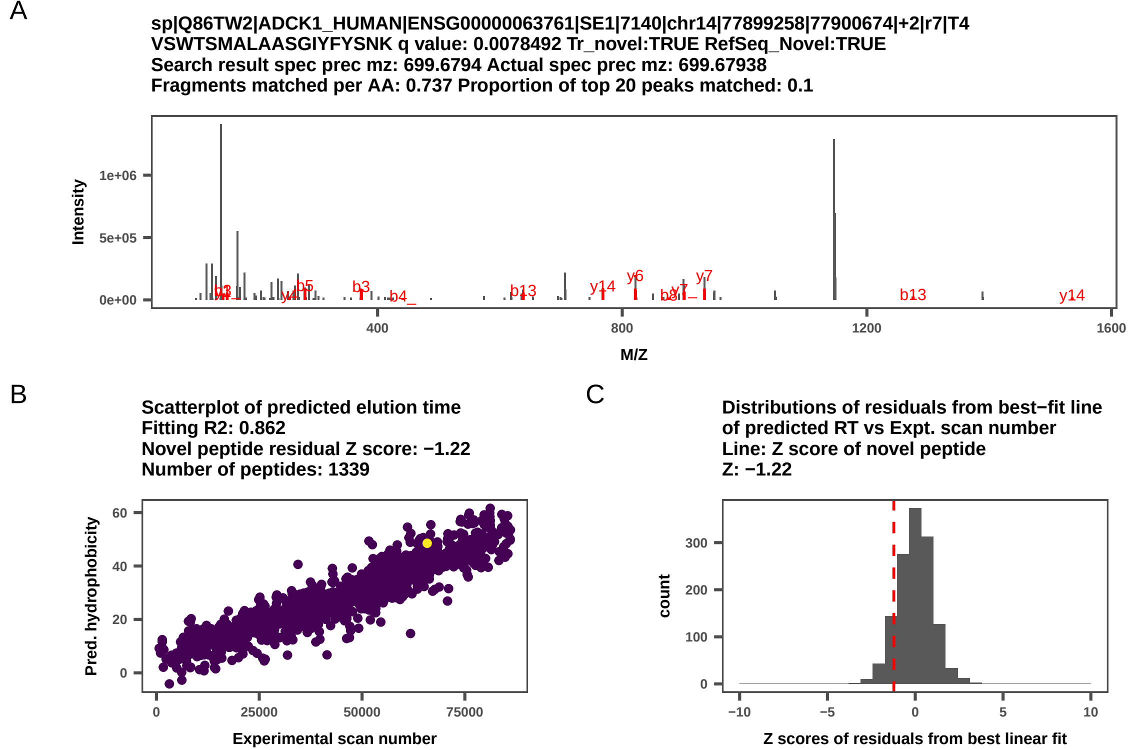

A
sp|Q86TW2|ADCK1_HUMAN|ENSG00000063761|SE1|7140|chr14|77899258|77900674|+2|r7|T4
VSWTSMALAASGIYFYSNK q value: 0.0078492 Tr_novel:TRUE RefSeq_Novel:TRUE
Search result spec prec mz: 699.6794 Actual spec prec mz: 699.67938
Fragments matched per AA: 0.737 Proportion of top 20 peaks matched: 0.1
Intensity
1e+06
5e+05
0e+00
b5
b3
b4_
b13
y14
y6
y7
b13
y14
400
800
1200
1600
M/Z
B
Scatterplot of predicted elution time
Fitting R2: 0.862
Novel peptide residual Z score: −1.22
Number of peptides: 1339
Pred. hydrophobicity
60
40
20
0
25000
50000
75000
Experimental scan number
C
Distributions of residuals from best−fit line
of predicted RT vs Expt. scan number
Line: Z score of novel peptide
Z: −1.22
count
300
200
100
0
−10
−5
0
5
10
Z scores of residuals from best linear fit
