# Supplementary material for: Splice-Junction-Based Mapping of Alternative Isoforms in the Human Proteome
Source: Cell Rep. Author manuscript; Available in PMC 2020 Jan 15. (PMC6961840; doi:10.1016/j.celrep.2019.11.026)
Supplement: 2 [file NIHMS1546469-supplement-2.zip › DF1/PXD006675/LeftVentricle/LeftVentricle_40_LDB3_QPGGTQPQPPGPWGFR.pdf]

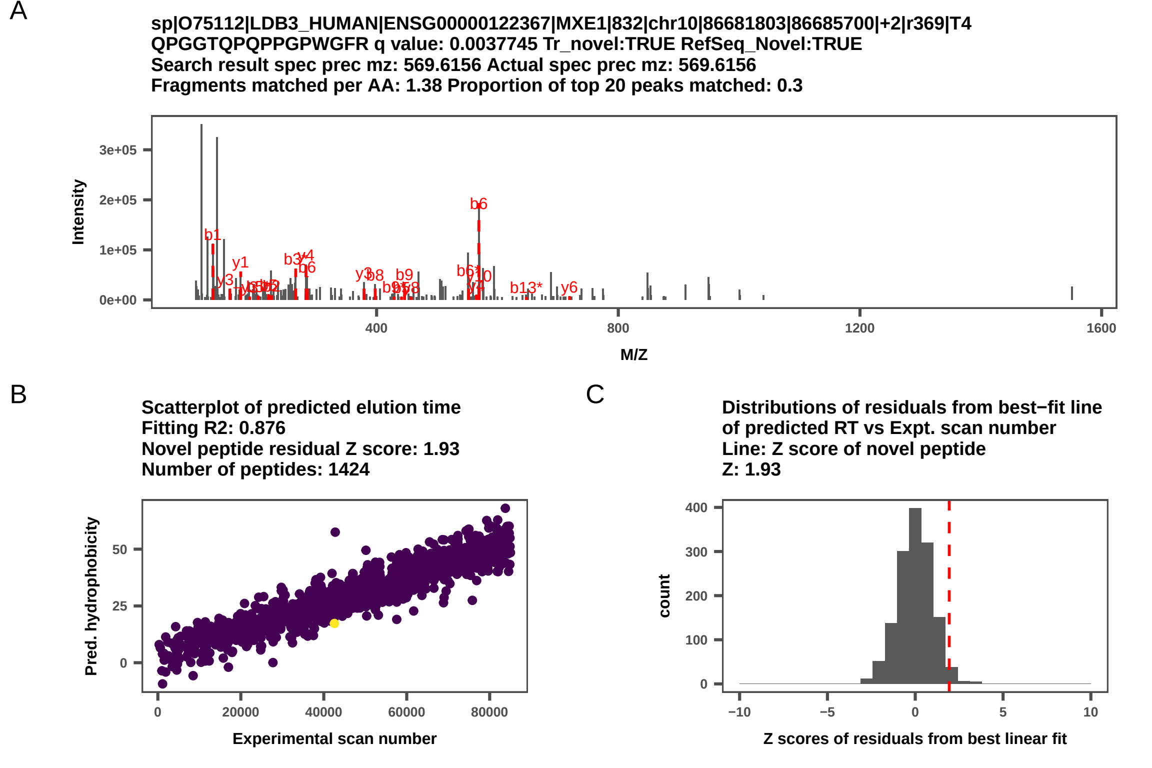

A
sp|O75112|LDB3_HUMAN|ENSG00000122367|MXE1|832|chr10|86681803|86685700|+2|r369|T4
QPGGTQPQPPGPWGFR q value: 0.0037745 Tr_novel:TRUE RefSeq_Novel:TRUE
Search result spec prec mz: 569.6156 Actual spec prec mz: 569.6156
Fragments matched per AA: 1.38 Proportion of top 20 peaks matched: 0.3
Intensity
3e+05
2e+05
1e+05
0e+00
b1
y1
y3
b3*
y4
b6
y3
b8
b9
b6
b6*
y10
b13*
y6
400
800
1200
1600
M/Z
B
Scatterplot of predicted elution time
Fitting R2: 0.876
Novel peptide residual Z score: 1.93
Number of peptides: 1424
Pred. hydrophobicity
50
25
0
0
20000
40000
60000
80000
Experimental scan number
C
Distributions of residuals from best−fit line
of predicted RT vs Expt. scan number
Line: Z score of novel peptide
Z: 1.93
count
400
300
200
100
0
−10
−5
0
5
10
Z scores of residuals from best linear fit
