# Supplementary material for: Splice-Junction-Based Mapping of Alternative Isoforms in the Human Proteome
Source: Cell Rep. Author manuscript; Available in PMC 2020 Jan 15. (PMC6961840; doi:10.1016/j.celrep.2019.11.026)

A

sp|P06396|GELS_HUMAN|ENSG00000148180|SE1|44726|chr9|−1|121300126|+0|r4|T1
AELAMEKLFCCFPNSMVVEHPEFLK q value: 0.0015994 Tr_novel:TRUE RefSeq_Novel:TRUE
Search result spec prec mz: 1009.4852 Actual spec prec mz: 1009.4851
Fragments matched per AA: 1.08 Proportion of top 20 peaks matched: 0.25

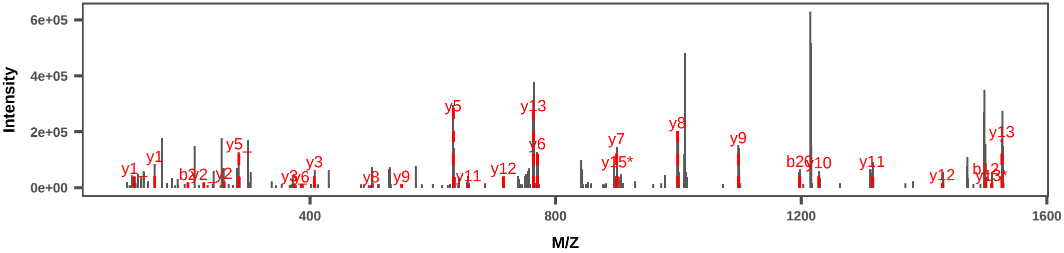

B

Scatterplot of predicted elution time
Fitting R2: 0.869
Novel peptide residual Z score: −3.77
Number of peptides: 1871

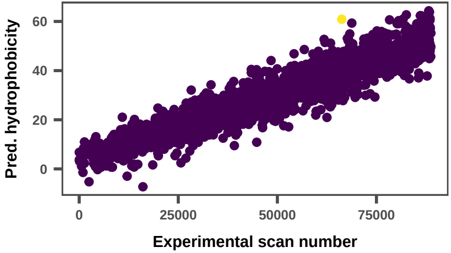

C

Distributions of residuals from best−fit line
of predicted RT vs Expt. scan number
Line: Z score of novel peptide
Z: −3.77

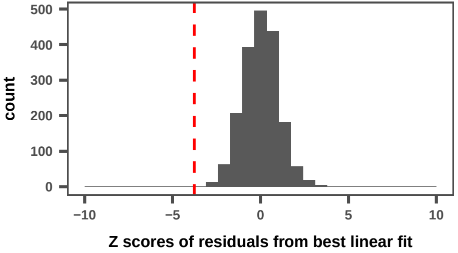

Supplement: 2 [file NIHMS1546469-supplement-2.zip › DF1/PXD006675/LeftVentricle/LeftVentricle_29_GSN_AELAMEKLFCCFPNSMVVEHPEFLK.pdf]
