# Supplementary material for: Splice-Junction-Based Mapping of Alternative Isoforms in the Human Proteome
Source: Cell Rep. Author manuscript; Available in PMC 2020 Jan 15. (PMC6961840; doi:10.1016/j.celrep.2019.11.026)
Supplement: 2 [file NIHMS1546469-supplement-2.zip › DF1/PXD006675/LeftVentricle/LeftVentricle_32_SYPL1_ETSLHSPSNTSAPHSQGGIPPPTGL.pdf]

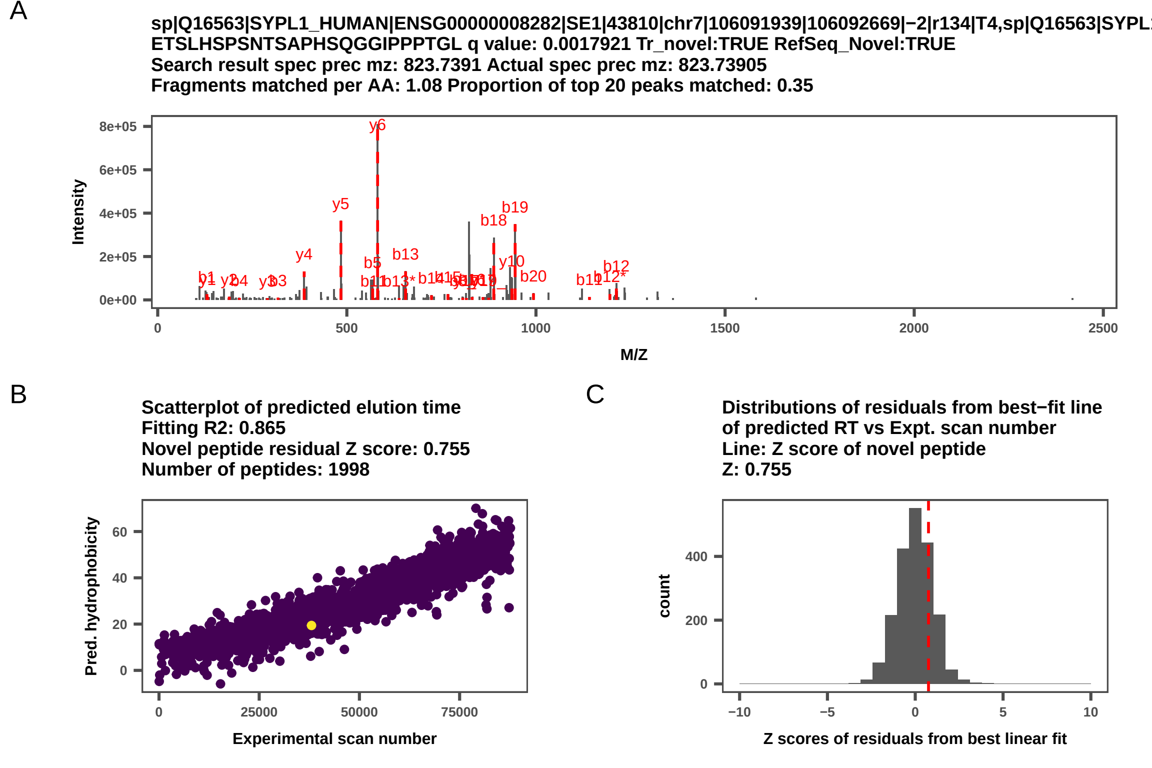
A
sp|Q16563|SYPL1_HUMAN|ENSG00000008282|SE1|43810|chr7|106091939|106092669|−2|r134|T4,sp|Q16563|SYPL
ETSLHSPSNTSAPHSQGGIPPPTGL q value: 0.0017921 Tr_novel:TRUE RefSeq_Novel:TRUE
Search result spec prec mz: 823.7391 Actual spec prec mz: 823.73905
Fragments matched per AA: 1.08 Proportion of top 20 peaks matched: 0.35
Intensity
8e+05
6e+05
4e+05
2e+05
0e+00
y6
y5
y4
b19
b18
b13
b5
y10
b20
b12
b12*
b1
y1
b4
b3
b11
b13*
M/Z
0
500
1000
1500
2000
2500
B
Scatterplot of predicted elution time
Fitting R2: 0.865
Novel peptide residual Z score: 0.755
Number of peptides: 1998
Pred. hydrophobicity
60
40
20
0
0
25000
50000
75000
Experimental scan number
C
Distributions of residuals from best−fit line
of predicted RT vs Expt. scan number
Line: Z score of novel peptide
Z: 0.755
count
400
200
0
−10
−5
0
5
10
Z scores of residuals from best linear fit
