# Supplementary material for: Splice-Junction-Based Mapping of Alternative Isoforms in the Human Proteome
Source: Cell Rep. Author manuscript; Available in PMC 2020 Jan 15. (PMC6961840; doi:10.1016/j.celrep.2019.11.026)
Supplement: 2 [file NIHMS1546469-supplement-2.zip › DF1/PXD006675/LeftVentricle/LeftVentricle_54_POLR3GL_LEGLQFCSPR.pdf]

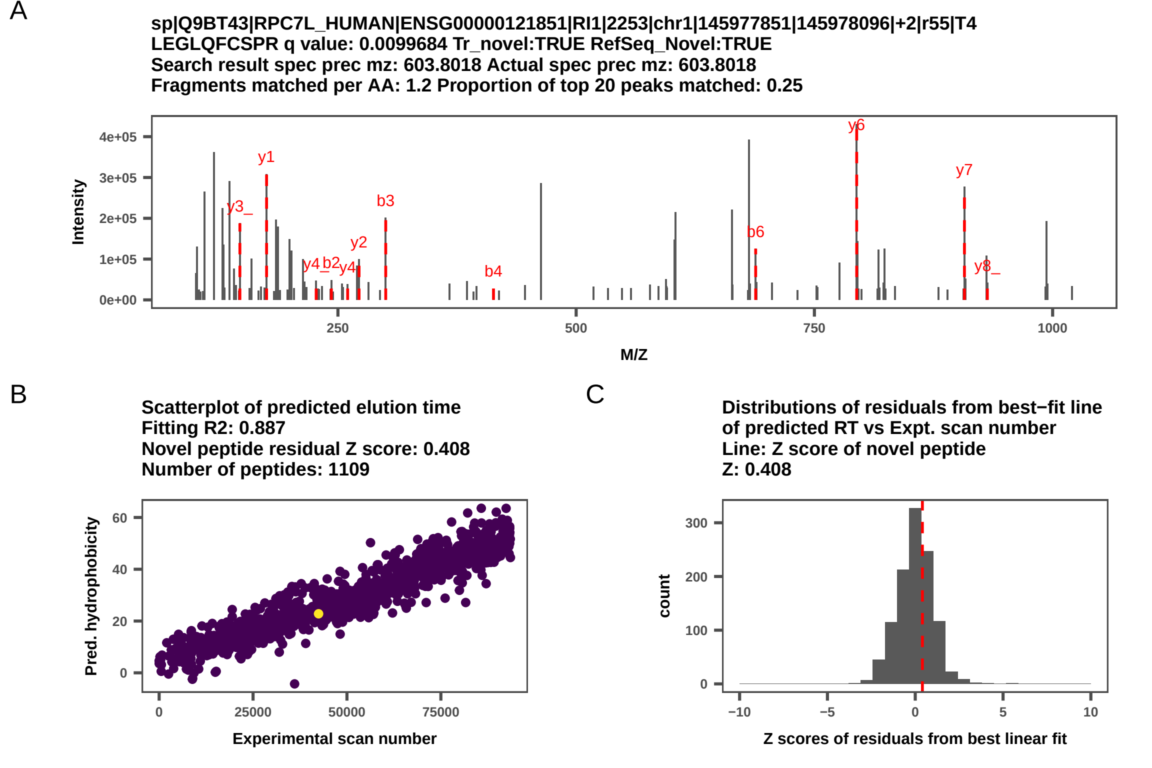
A
sp|Q9BT43|RPC7L_HUMAN|ENSG00000121851|RI1|2253|chr1|145977851|145978096|+2|r55|T4
LEGLQFCSPR q value: 0.0099684 Tr_novel:TRUE RefSeq_Novel:TRUE
Search result spec prec mz: 603.8018 Actual spec prec mz: 603.8018
Fragments matched per AA: 1.2 Proportion of top 20 peaks matched: 0.25
Intensity
4e+05
3e+05
2e+05
1e+05
0e+00
y1
y3_
b3
y2
y4
b2_
y4
b4
y6
y7
b6
y8_
250
500
750
1000
M/Z
B
Scatterplot of predicted elution time
Fitting R2: 0.887
Novel peptide residual Z score: 0.408
Number of peptides: 1109
Pred. hydrophobicity
60
40
20
0
0
25000
50000
75000
Experimental scan number
C
Distributions of residuals from best−fit line
of predicted RT vs Expt. scan number
Line: Z score of novel peptide
Z: 0.408
count
300
200
100
0
−10
−5
0
5
10
Z scores of residuals from best linear fit
