# Supplementary material for: Splice-Junction-Based Mapping of Alternative Isoforms in the Human Proteome
Source: Cell Rep. Author manuscript; Available in PMC 2020 Jan 15. (PMC6961840; doi:10.1016/j.celrep.2019.11.026)
Supplement: 2 [file NIHMS1546469-supplement-2.zip › DF1/PXD006675/LeftVentricle/LeftVentricle_33_CBFA2T2_VTAIDTNGQPAPAAAGTAALR.pdf]

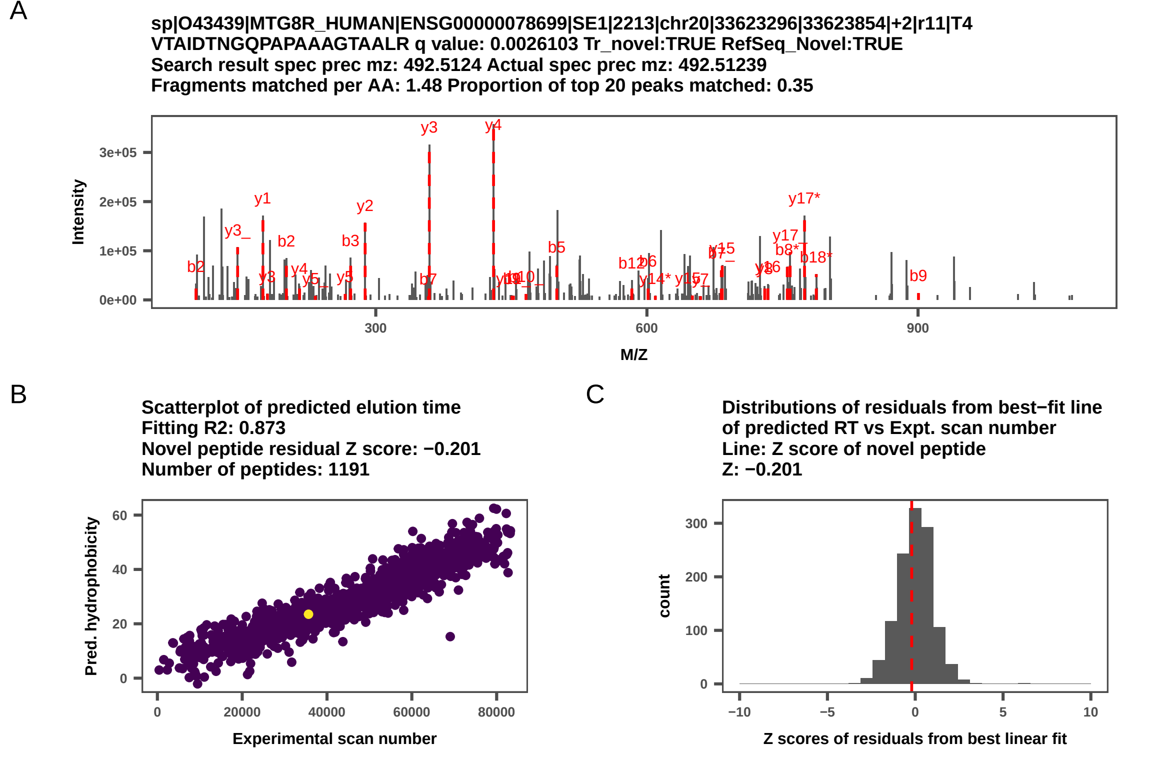
A
sp|O43439|MTG8R_HUMAN|ENSG00000078699|SE1|2213|chr20|33623296|33623854|+2|r11|T4
VTAIDTNGQPAPAAAGTAALR q value: 0.0026103 Tr_novel:TRUE RefSeq_Novel:TRUE
Search result spec prec mz: 492.5124 Actual spec prec mz: 492.51239
Fragments matched per AA: 1.48 Proportion of top 20 peaks matched: 0.35
Intensity
3e+05
2e+05
1e+05
0e+00
y3
y4
y1
y2
y17*
y3_
b2
b3
b5
y17
b18*
b9
b7
y14*
M/Z
300
600
900
B
Scatterplot of predicted elution time
Fitting R2: 0.873
Novel peptide residual Z score: −0.201
Number of peptides: 1191
Pred. hydrophobicity
60
40
20
0
0
20000
40000
60000
80000
Experimental scan number
C
Distributions of residuals from best−fit line
of predicted RT vs Expt. scan number
Line: Z score of novel peptide
Z: −0.201
count
300
200
100
0
−10
−5
0
5
10
Z scores of residuals from best linear fit
