# Supplementary material for: Splice-Junction-Based Mapping of Alternative Isoforms in the Human Proteome
Source: Cell Rep. Author manuscript; Available in PMC 2020 Jan 15. (PMC6961840; doi:10.1016/j.celrep.2019.11.026)
Supplement: 2 [file NIHMS1546469-supplement-2.zip › DF1/PXD006675/LeftVentricle/LeftVentricle_52_ABHD4_IPQGWLSSWLPTWRPTSMSQLK.pdf]

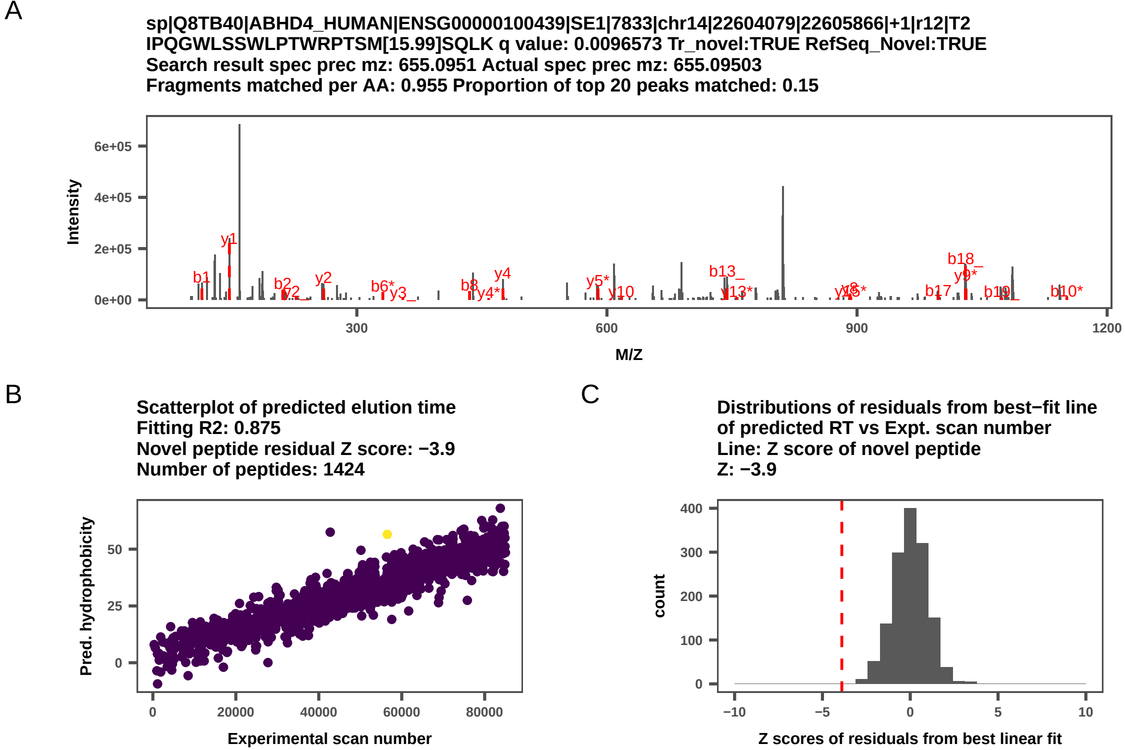
A
sp|Q8TB40|ABHD4_HUMAN|ENSG00000100439|SE1|7833|chr14|22604079|22605866|+1|r12|T2
IPQGWLSSWLPTWRPTSM[15.99]SQLK q value: 0.0096573 Tr_novel:TRUE RefSeq_Novel:TRUE
Search result spec prec mz: 655.0951 Actual spec prec mz: 655.09503
Fragments matched per AA: 0.955 Proportion of top 20 peaks matched: 0.15
Intensity
6e+05
4e+05
2e+05
0e+00
y1
b1
b2
y2
b6*
y3_
b8
y4*
y4
y5*
y10
b13_
y13*
b18_
y9*
b17
b19
b10*
300
600
900
1200
M/Z
B
Scatterplot of predicted elution time
Fitting R2: 0.875
Novel peptide residual Z score: −3.9
Number of peptides: 1424
Pred. hydrophobicity
50
25
0
0
20000
40000
60000
80000
Experimental scan number
C
Distributions of residuals from best−fit line
of predicted RT vs Expt. scan number
Line: Z score of novel peptide
Z: −3.9
count
400
300
200
100
0
−10
−5
0
5
10
Z scores of residuals from best linear fit
