# Supplementary material for: Splice-Junction-Based Mapping of Alternative Isoforms in the Human Proteome
Source: Cell Rep. Author manuscript; Available in PMC 2020 Jan 15. (PMC6961840; doi:10.1016/j.celrep.2019.11.026)
Supplement: 2 [file NIHMS1546469-supplement-2.zip › DF1/PXD006675/LeftVentricle/LeftVentricle_19_TTN_ASNEYGSVSCTATLTVTEPPR.pdf]

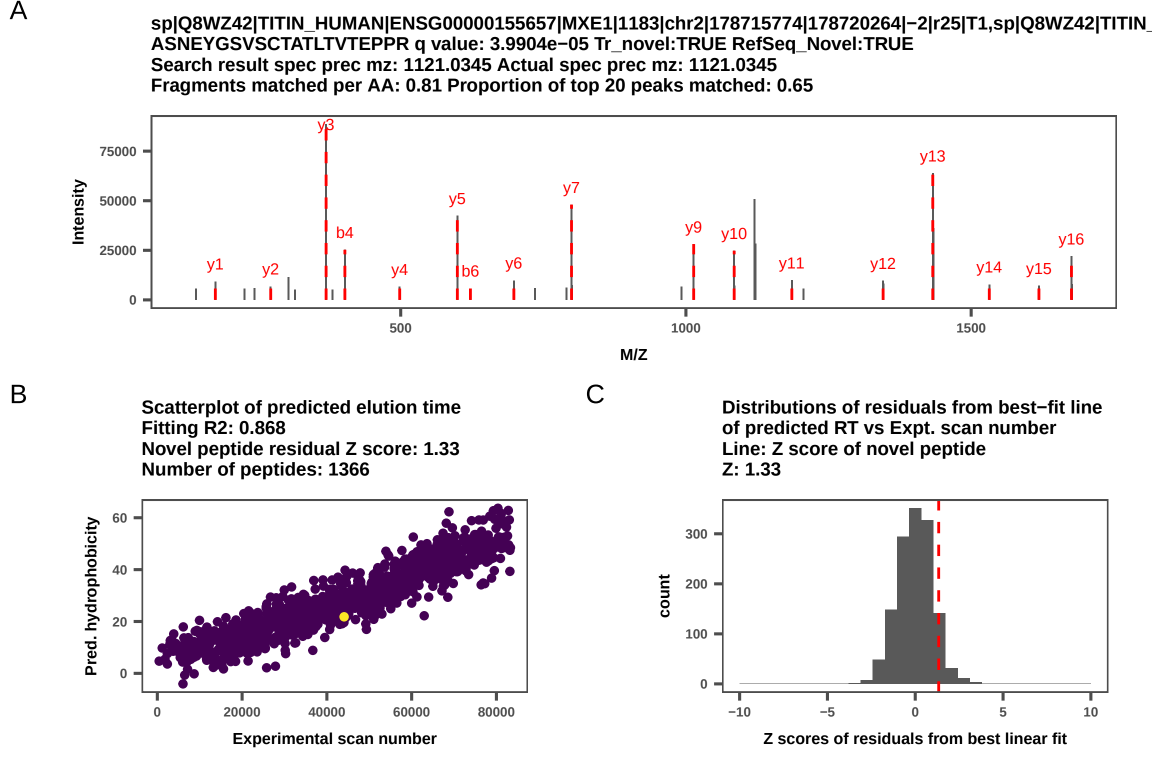

A
sp|Q8WZ42|TITIN_HUMAN|ENSG00000155657|MXE1|1183|chr2|178715774|178720264|−2|r25|T1,sp|Q8WZ42|TITIN_
ASNEYGSVSCTATLTVTEPPR q value: 3.9904e−05 Tr_novel:TRUE RefSeq_Novel:TRUE
Search result spec prec mz: 1121.0345 Actual spec prec mz: 1121.0345
Fragments matched per AA: 0.81 Proportion of top 20 peaks matched: 0.65
Intensity
75000
50000
25000
0
y3
y13
y7
y5
b4
y9
y10
y16
y1
y2
y4
b6
y6
y11
y12
y14
y15
500
1000
1500
M/Z
B
Scatterplot of predicted elution time
Fitting R2: 0.868
Novel peptide residual Z score: 1.33
Number of peptides: 1366
Pred. hydrophobicity
60
40
20
0
0
20000
40000
60000
80000
Experimental scan number
C
Distributions of residuals from best−fit line
of predicted RT vs Expt. scan number
Line: Z score of novel peptide
Z: 1.33
count
300
200
100
0
−10
−5
0
5
10
Z scores of residuals from best linear fit
