# Supplementary material for: Splice-Junction-Based Mapping of Alternative Isoforms in the Human Proteome
Source: Cell Rep. Author manuscript; Available in PMC 2020 Jan 15. (PMC6961840; doi:10.1016/j.celrep.2019.11.026)
Supplement: 2 [file NIHMS1546469-supplement-2.zip › DF1/PXD006675/LeftVentricle/LeftVentricle_34_ARIH2_FSCLSLLSSWDYRSLLHGSGLSTPYTR.pdf]

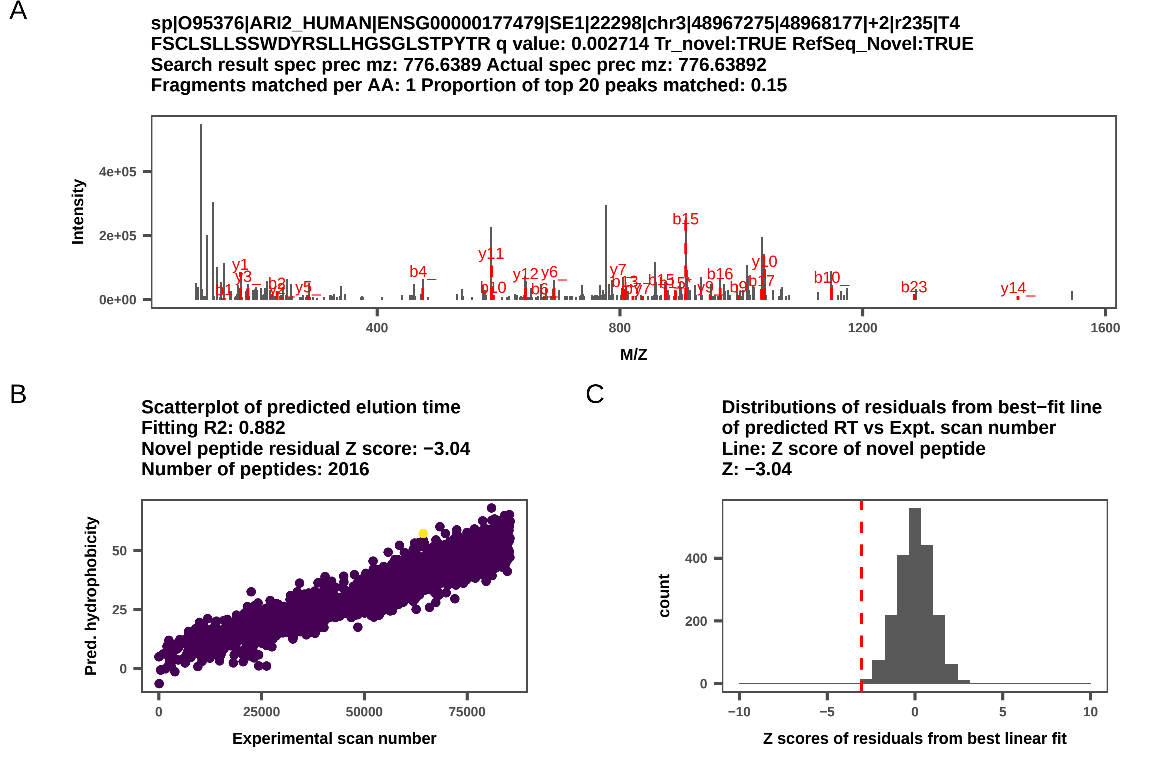
A
sp|O95376|ARI2_HUMAN|ENSG00000177479|SE1|22298|chr3|48967275|48968177|+2|r235|T4
FSCLSLLSSWDYRSLLHGSGLSTPYTR q value: 0.002714 Tr_novel:TRUE RefSeq_Novel:TRUE
Search result spec prec mz: 776.6389 Actual spec prec mz: 776.63892
Fragments matched per AA: 1 Proportion of top 20 peaks matched: 0.15
Intensity
4e+05
2e+05
0e+00
400
800
1200
1600
M/Z
B
Scatterplot of predicted elution time
Fitting R2: 0.882
Novel peptide residual Z score: −3.04
Number of peptides: 2016
Pred. hydrophobicity
50
25
0
0
25000
50000
75000
Experimental scan number
C
Distributions of residuals from best−fit line
of predicted RT vs Expt. scan number
Line: Z score of novel peptide
Z: −3.04
count
400
200
0
−10
−5
0
5
10
Z scores of residuals from best linear fit
