# Supplementary material for: Splice-Junction-Based Mapping of Alternative Isoforms in the Human Proteome
Source: Cell Rep. Author manuscript; Available in PMC 2020 Jan 15. (PMC6961840; doi:10.1016/j.celrep.2019.11.026)
Supplement: 2 [file NIHMS1546469-supplement-2.zip › DF1/PXD006675/LeftVentricle/LeftVentricle_44_TPM3_AISEELDHALNDMTSGS.pdf]

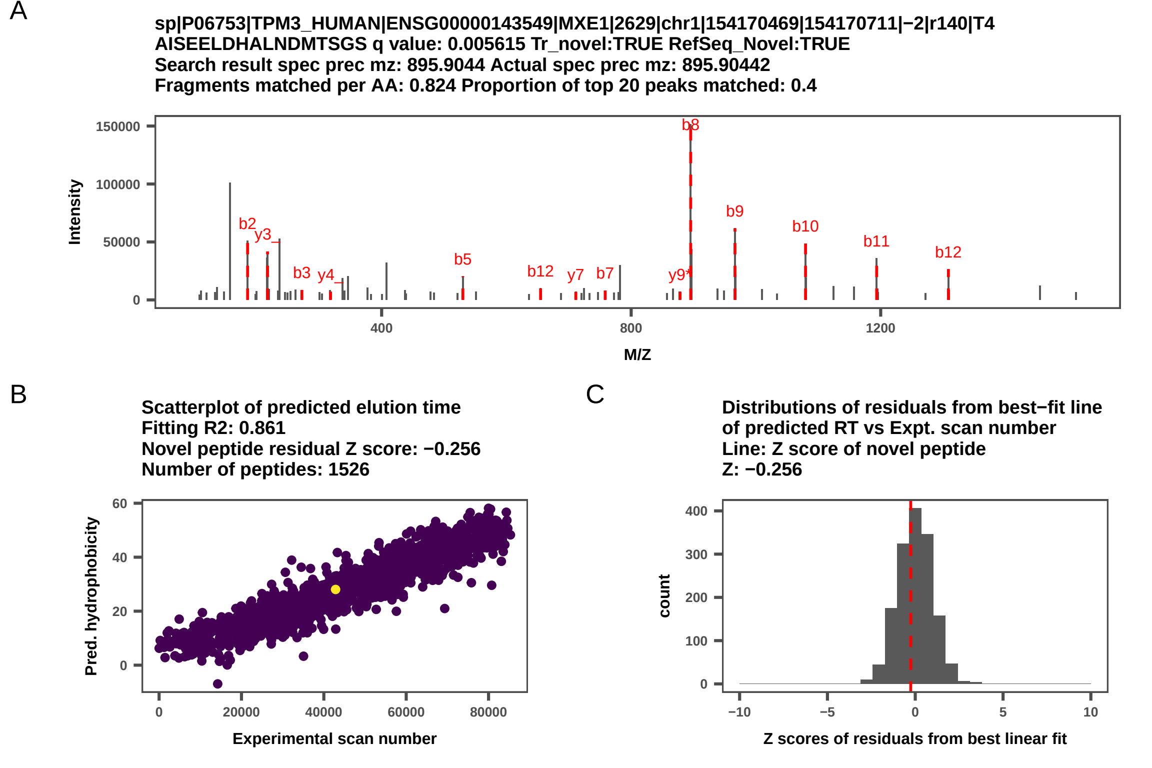
A
sp|P06753|TPM3_HUMAN|ENSG00000143549|MXE1|2629|chr1|154170469|154170711|−2|r140|T4
AISEELDHALNDMTSGS q value: 0.005615 Tr_novel:TRUE RefSeq_Novel:TRUE
Search result spec prec mz: 895.9044 Actual spec prec mz: 895.90442
Fragments matched per AA: 0.824 Proportion of top 20 peaks matched: 0.4
Intensity
150000
100000
50000
0
b8
b2
y3
b3
y4
b5
b12
y7
b7
y9*
b9
b10
b11
b12
400
800
1200
M/Z
B
Scatterplot of predicted elution time
Fitting R2: 0.861
Novel peptide residual Z score: −0.256
Number of peptides: 1526
Pred. hydrophobicity
60
40
20
0
0
20000
40000
60000
80000
Experimental scan number
C
Distributions of residuals from best−fit line
of predicted RT vs Expt. scan number
Line: Z score of novel peptide
Z: −0.256
count
400
300
200
100
0
−10
−5
0
5
10
Z scores of residuals from best linear fit
