# Supplementary material for: Splice-Junction-Based Mapping of Alternative Isoforms in the Human Proteome
Source: Cell Rep. Author manuscript; Available in PMC 2020 Jan 15. (PMC6961840; doi:10.1016/j.celrep.2019.11.026)
Supplement: 2 [file NIHMS1546469-supplement-2.zip › DF1/PXD006675/LeftVentricle/LeftVentricle_42_LUC7L2_TVVLMMSFLEMLDQLMGTSR.pdf]

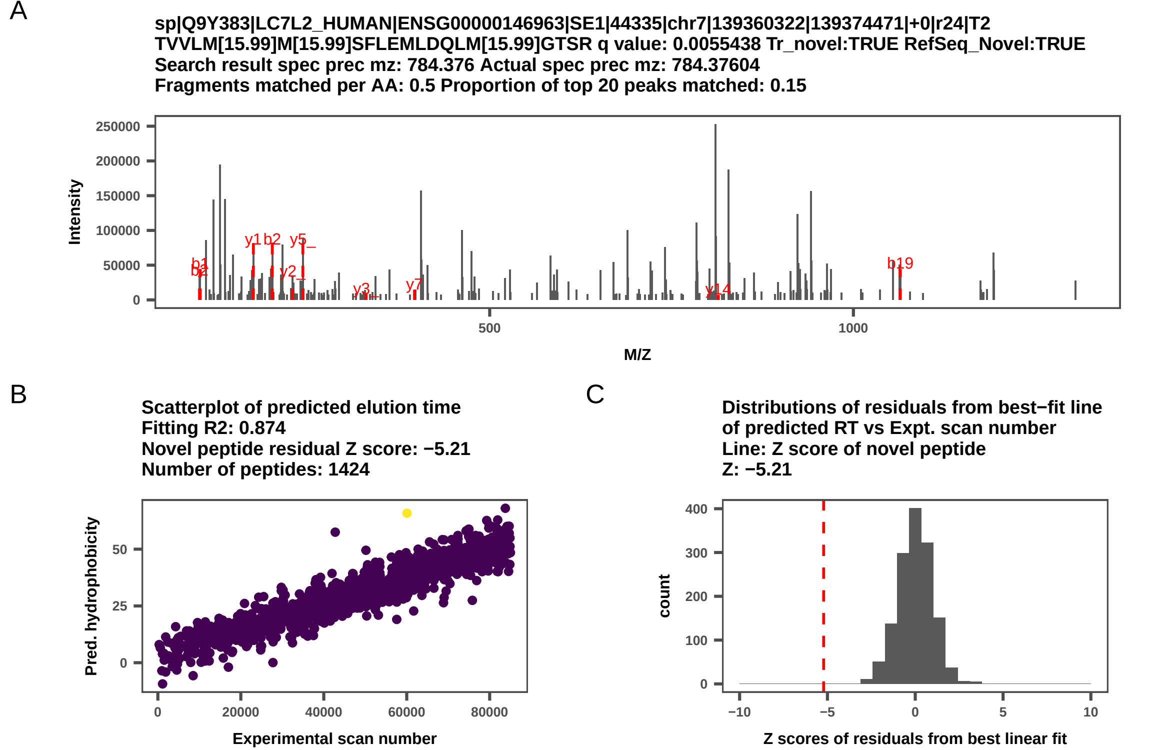
A
sp|Q9Y383|LC7L2_HUMAN|ENSG00000146963|SE1|44335|chr7|139360322|139374471|+0|r24|T2
TVVLM[15.99]M[15.99]SFLEMLDQLM[15.99]GTSR q value: 0.0055438 Tr_novel:TRUE RefSeq_Novel:TRUE
Search result spec prec mz: 784.376 Actual spec prec mz: 784.37604
Fragments matched per AA: 0.5 Proportion of top 20 peaks matched: 0.15
Intensity
250000
200000
150000
100000
50000
0
b1
b2
y1
b2
y5
y2
y3
y7
y14
b19
500
1000
M/Z
B
Scatterplot of predicted elution time
Fitting R2: 0.874
Novel peptide residual Z score: −5.21
Number of peptides: 1424
Pred. hydrophobicity
50
25
0
0
20000
40000
60000
80000
Experimental scan number
C
Distributions of residuals from best−fit line
of predicted RT vs Expt. scan number
Line: Z score of novel peptide
Z: −5.21
count
400
300
200
100
0
−10
−5
0
5
10
Z scores of residuals from best linear fit
