# Supplementary material for: Splice-Junction-Based Mapping of Alternative Isoforms in the Human Proteome
Source: Cell Rep. Author manuscript; Available in PMC 2020 Jan 15. (PMC6961840; doi:10.1016/j.celrep.2019.11.026)

A

sp|P09493|TPM1_HUMAN|ENSG00000140416|SE1|6193|chr15|63060939|63061273|+2|r8781|T4
AISEELDHALNDMTSGP q value: 0.0055792 Tr_novel:TRUE RefSeq_Novel:TRUE
Search result spec prec mz: 900.9095 Actual spec prec mz: 900.90958
Fragments matched per AA: 1.12 Proportion of top 20 peaks matched: 0.45

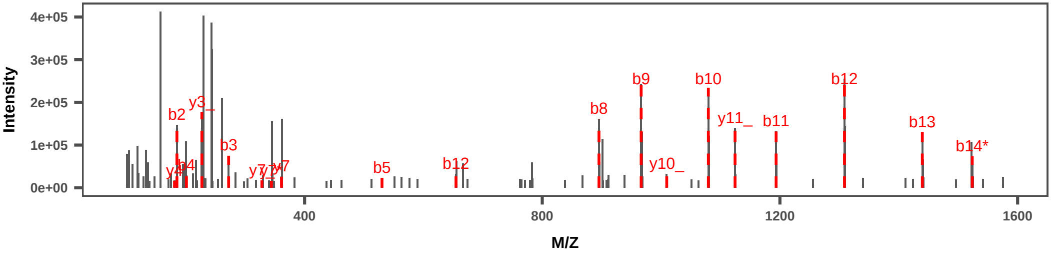

B

Scatterplot of predicted elution time
Fitting R2: 0.871
Novel peptide residual Z score: 1.25
Number of peptides: 1533

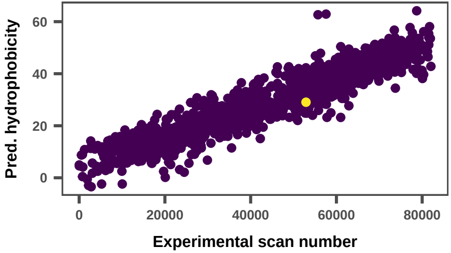

C

Distributions of residuals from best−fit line
of predicted RT vs Expt. scan number
Line: Z score of novel peptide
Z: 1.25

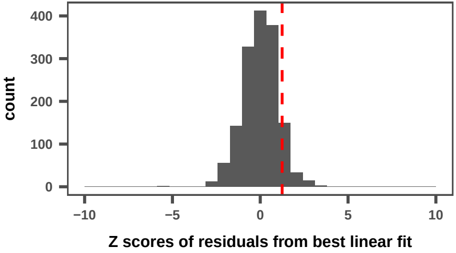

Supplement: 2 [file NIHMS1546469-supplement-2.zip › DF1/PXD006675/LeftVentricle/LeftVentricle_43_TPM1_AISEELDHALNDMTSGP.pdf]
