# Supplementary material for: Splice-Junction-Based Mapping of Alternative Isoforms in the Human Proteome
Source: Cell Rep. Author manuscript; Available in PMC 2020 Jan 15. (PMC6961840; doi:10.1016/j.celrep.2019.11.026)
Supplement: 2 [file NIHMS1546469-supplement-2.zip › DF1/PXD006675/LeftVentricle/LeftVentricle_51_DEF8_QSLFQEPGPDVEGHPVPASPWALAGDAEALPE.pdf]

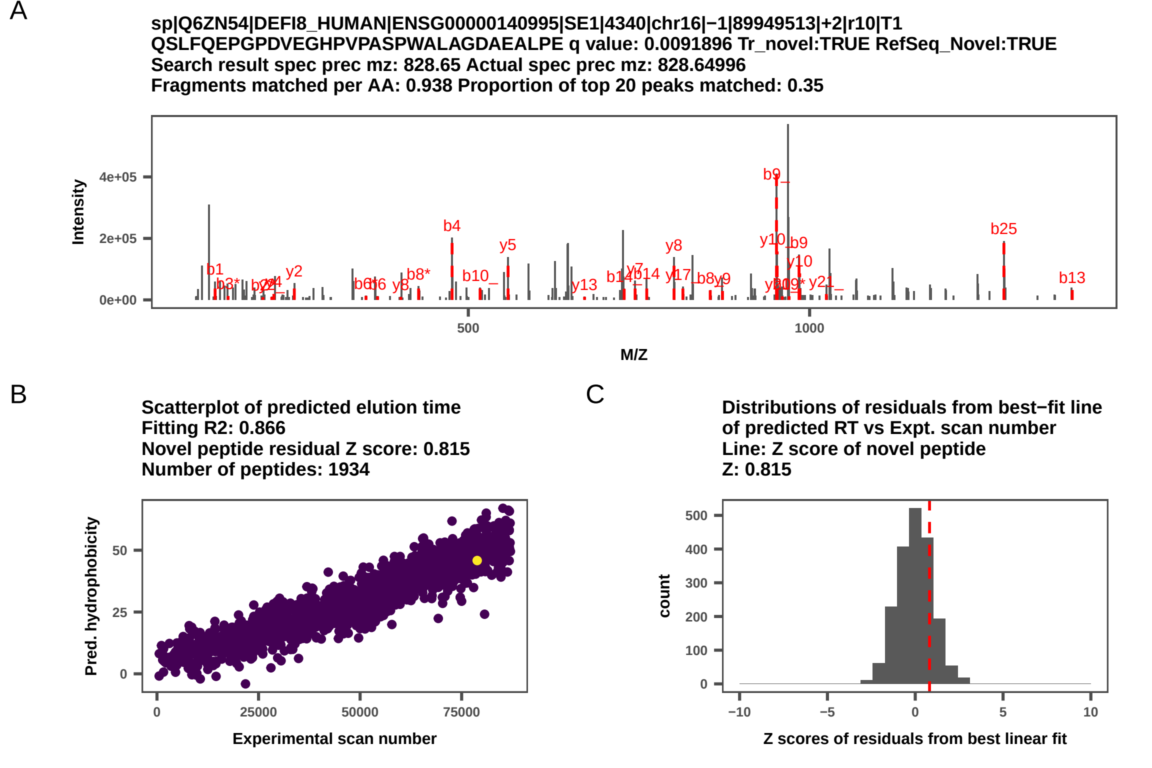
A
sp|Q6ZN54|DEFI8_HUMAN|ENSG00000140995|SE1|4340|chr16|−1|89949513|+2|r10|T1
QSLFQEPGPDVEGHPVPASPWALAGDAEALPE q value: 0.0091896 Tr_novel:TRUE RefSeq_Novel:TRUE
Search result spec prec mz: 828.65 Actual spec prec mz: 828.64996
Fragments matched per AA: 0.938 Proportion of top 20 peaks matched: 0.35
Intensity
4e+05
2e+05
0e+00
b1
b4
b10_
y2
y5
y13
y8
b9_
y10
b9
b25
b13
500
1000
M/Z
B
Scatterplot of predicted elution time
Fitting R2: 0.866
Novel peptide residual Z score: 0.815
Number of peptides: 1934
Pred. hydrophobicity
50
25
0
0
25000
50000
75000
Experimental scan number
C
Distributions of residuals from best−fit line
of predicted RT vs Expt. scan number
Line: Z score of novel peptide
Z: 0.815
count
500
400
300
200
100
0
−10
−5
0
5
10
Z scores of residuals from best linear fit
