# Supplementary material for: Splice-Junction-Based Mapping of Alternative Isoforms in the Human Proteome
Source: Cell Rep. Author manuscript; Available in PMC 2020 Jan 15. (PMC6961840; doi:10.1016/j.celrep.2019.11.026)
Supplement: 2 [file NIHMS1546469-supplement-2.zip › DF1/PXD006675/LeftVentricle/LeftVentricle_46_GDI2_SLGTGLTECILSGIMSVNGK.pdf]

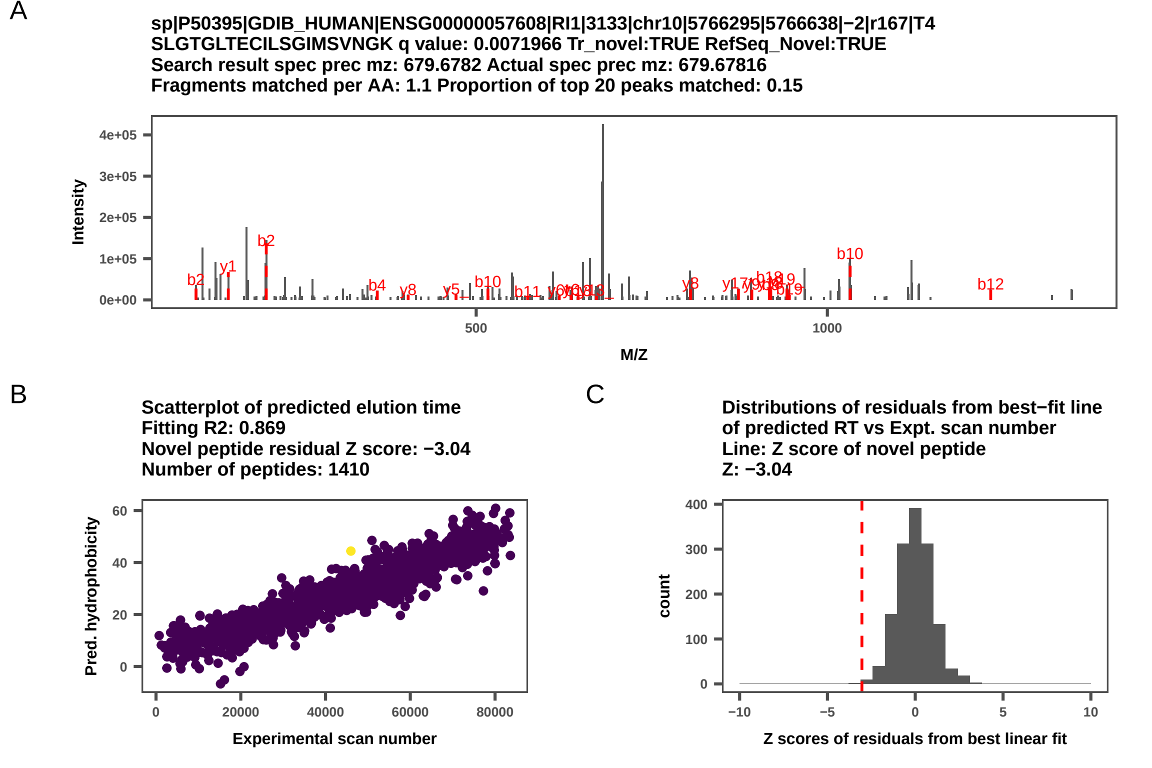

A
sp|P50395|GDIB_HUMAN|ENSG00000057608|RI1|3133|chr10|5766295|5766638|−2|r167|T4
SLGTGLTECILSGIMSVNGK q value: 0.0071966 Tr_novel:TRUE RefSeq_Novel:TRUE
Search result spec prec mz: 679.6782 Actual spec prec mz: 679.67816
Fragments matched per AA: 1.1 Proportion of top 20 peaks matched: 0.15
Intensity
4e+05
3e+05
2e+05
1e+05
0e+00
b2
y1
b2
b4
y8
y5
b10
b11
y8
b10
b12
500
1000
M/Z
B
Scatterplot of predicted elution time
Fitting R2: 0.869
Novel peptide residual Z score: −3.04
Number of peptides: 1410
Pred. hydrophobicity
60
40
20
0
0
20000
40000
60000
80000
Experimental scan number
C
Distributions of residuals from best−fit line
of predicted RT vs Expt. scan number
Line: Z score of novel peptide
Z: −3.04
count
400
300
200
100
0
−10
−5
0
5
10
Z scores of residuals from best linear fit
