# Supplementary material for: Splice-Junction-Based Mapping of Alternative Isoforms in the Human Proteome
Source: Cell Rep. Author manuscript; Available in PMC 2020 Jan 15. (PMC6961840; doi:10.1016/j.celrep.2019.11.026)
Supplement: 2 [file NIHMS1546469-supplement-2.zip › DF1/PXD006675/LeftVentricle/LeftVentricle_4_PPP1R1A_ELQMMVEHHLGQQQQGEEPEGAAESTGTQESRPPGIPDTEVESR.pdf]

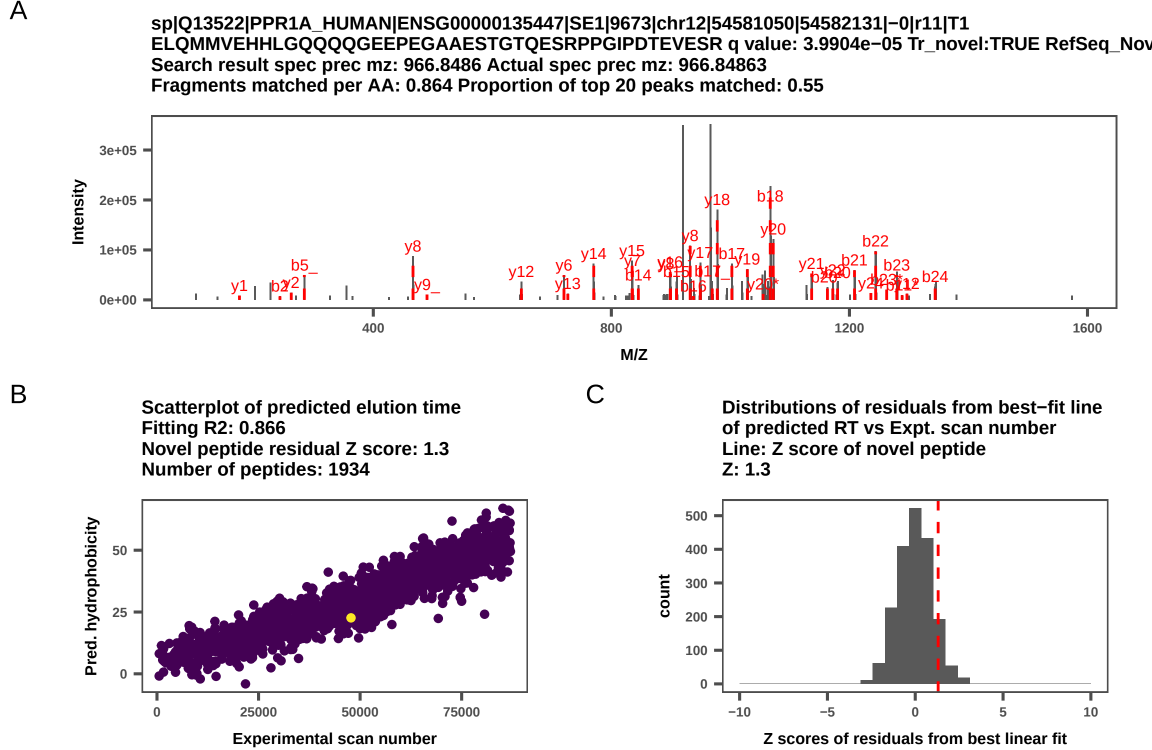
A
sp|Q13522|PPR1A_HUMAN|ENSG00000135447|SE1|9673|chr12|54581050|54582131|−0|r11|T1
ELQMMVEHHLGQQQQGEEPEGAAESTGTQESRPPGIPDTEVESR q value: 3.9904e−05 Tr_novel:TRUE RefSeq_Nov
Search result spec prec mz: 966.8486 Actual spec prec mz: 966.84863
Fragments matched per AA: 0.864 Proportion of top 20 peaks matched: 0.55
Intensity
3e+05
2e+05
1e+05
0e+00
M/Z
400
800
1200
1600
y1
b2
y2
b5_
y8
y9_
y12
y6
y13
y14
y15
y7
b14
y8
y18
b18
y20
y17
b17
y19
y21
b21
b22
b23
b24
B
Scatterplot of predicted elution time
Fitting R2: 0.866
Novel peptide residual Z score: 1.3
Number of peptides: 1934
Pred. hydrophobicity
0
25
50
Experimental scan number
0
25000
50000
75000
C
Distributions of residuals from best−fit line
of predicted RT vs Expt. scan number
Line: Z score of novel peptide
Z: 1.3
count
0
100
200
300
400
500
Z scores of residuals from best linear fit
−10
−5
0
5
10
