# Supplementary material for: Splice-Junction-Based Mapping of Alternative Isoforms in the Human Proteome
Source: Cell Rep. Author manuscript; Available in PMC 2020 Jan 15. (PMC6961840; doi:10.1016/j.celrep.2019.11.026)
Supplement: 2 [file NIHMS1546469-supplement-2.zip › DF1/PXD006675/LeftVentricle/LeftVentricle_8_SVIL_RPELCTSHSETPTVDDEEK.pdf]

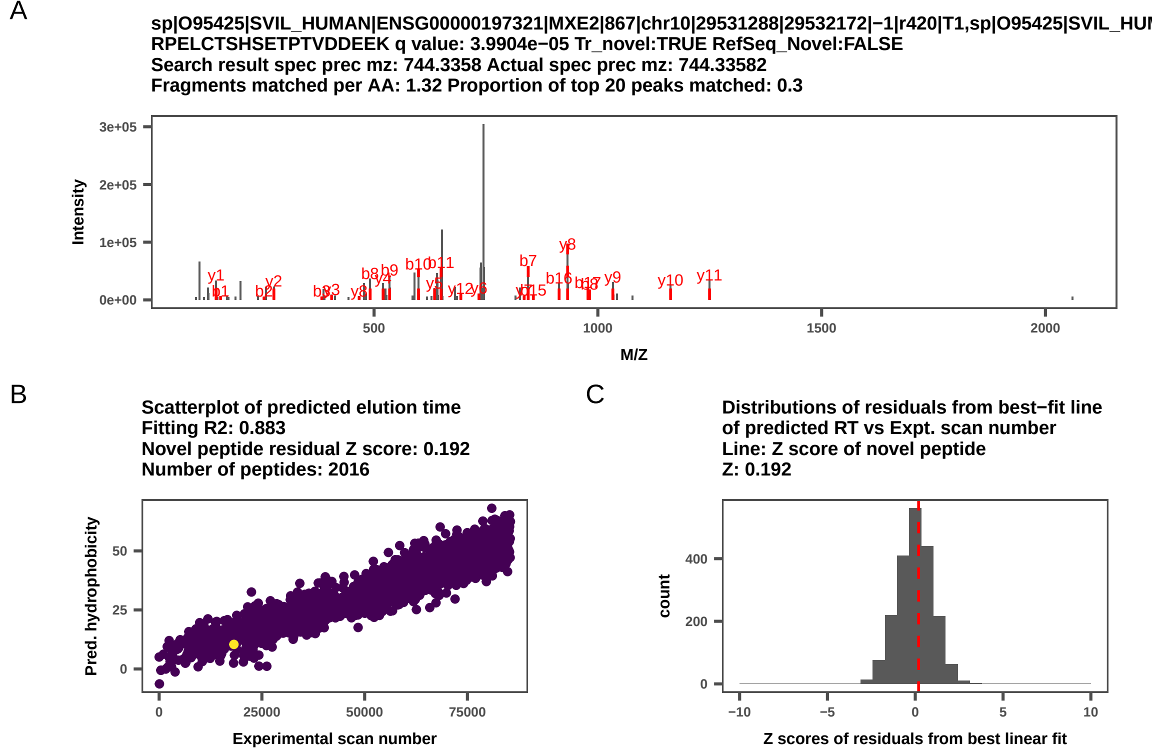
A
sp|O95425|SVIL_HUMAN|ENSG00000197321|MXE2|867|chr10|29531288|29532172|−1|r420|T1,sp|O95425|SVIL_HUM
RPELCTSHSETPTVDDEEK q value: 3.9904e−05 Tr_novel:TRUE RefSeq_Novel:FALSE
Search result spec prec mz: 744.3358 Actual spec prec mz: 744.33582
Fragments matched per AA: 1.32 Proportion of top 20 peaks matched: 0.3
Intensity
3e+05
2e+05
1e+05
0e+00
y1
b1
y2
b2
b8
b9
y4
b10
b11
y5
y12
b7
y8
b16
y9
y10
y11
500
1000
1500
2000
M/Z
B
Scatterplot of predicted elution time
Fitting R2: 0.883
Novel peptide residual Z score: 0.192
Number of peptides: 2016
Pred. hydrophobicity
50
25
0
25000
50000
75000
Experimental scan number
C
Distributions of residuals from best−fit line
of predicted RT vs Expt. scan number
Line: Z score of novel peptide
Z: 0.192
count
400
200
0
−10
−5
5
10
Z scores of residuals from best linear fit
