# Supplementary material for: Splice-Junction-Based Mapping of Alternative Isoforms in the Human Proteome
Source: Cell Rep. Author manuscript; Available in PMC 2020 Jan 15. (PMC6961840; doi:10.1016/j.celrep.2019.11.026)
Supplement: 2 [file NIHMS1546469-supplement-2.zip › DF1/PXD006675/LeftVentricle/LeftVentricle_39_MAP4_DSYVPLELAK.pdf]

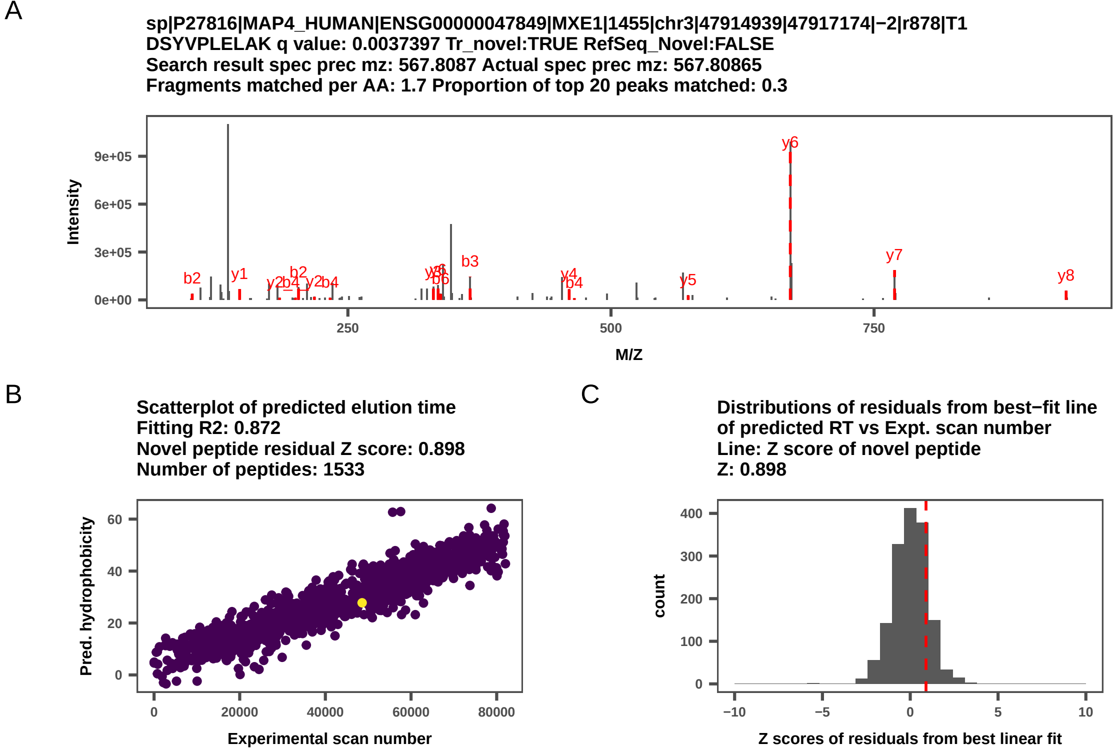
A
sp|P27816|MAP4_HUMAN|ENSG00000047849|MXE1|1455|chr3|47914939|47917174|−2|r878|T1
DSYVPLELAK q value: 0.0037397 Tr_novel:TRUE RefSeq_Novel:FALSE
Search result spec prec mz: 567.8087 Actual spec prec mz: 567.80865
Fragments matched per AA: 1.7 Proportion of top 20 peaks matched: 0.3
Intensity
9e+05
6e+05
3e+05
0e+00
b2
y1
b2
y6
b3
y4
b4
y5
y7
y8
250
500
750
M/Z
B
Scatterplot of predicted elution time
Fitting R2: 0.872
Novel peptide residual Z score: 0.898
Number of peptides: 1533
Pred. hydrophobicity
60
40
20
0
0
20000
40000
60000
80000
Experimental scan number
C
Distributions of residuals from best−fit line
of predicted RT vs Expt. scan number
Line: Z score of novel peptide
Z: 0.898
count
400
300
200
100
0
−10
−5
0
5
10
Z scores of residuals from best linear fit
