# Supplementary material for: Splice-Junction-Based Mapping of Alternative Isoforms in the Human Proteome
Source: Cell Rep. Author manuscript; Available in PMC 2020 Jan 15. (PMC6961840; doi:10.1016/j.celrep.2019.11.026)
Supplement: 2 [file NIHMS1546469-supplement-2.zip › DF1/PXD006675/LeftVentricle/LeftVentricle_27_TTN_SVPEPPPPKPVEEVEVPTVTK.pdf]

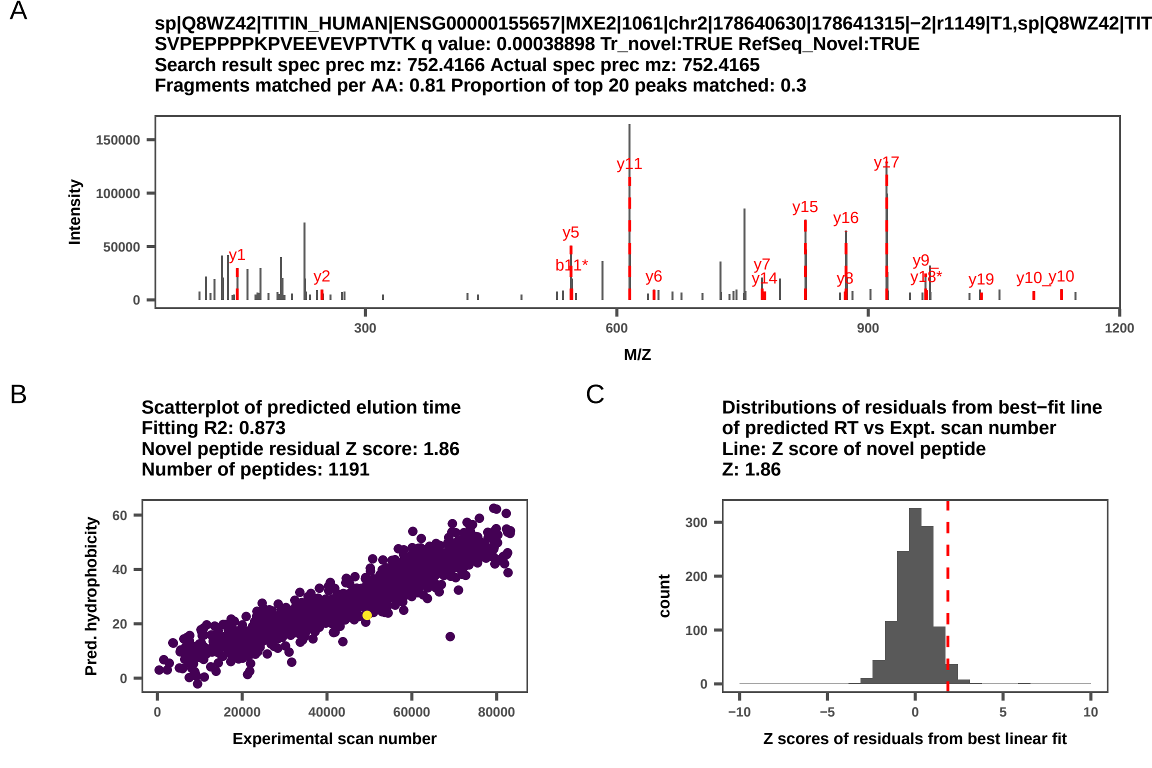
A
sp|Q8WZ42|TITIN_HUMAN|ENSG00000155657|MXE2|1061|chr2|178640630|178641315|−2|r1149|T1,sp|Q8WZ42|TIT
SVPEPPPPKPVEEVEVPTVTK q value: 0.00038898 Tr_novel:TRUE RefSeq_Novel:TRUE
Search result spec prec mz: 752.4166 Actual spec prec mz: 752.4165
Fragments matched per AA: 0.81 Proportion of top 20 peaks matched: 0.3
Intensity
150000
100000
50000
0
y1
y2
y5
b11*
y11
y6
y7
y14
y15
y8
y16
y17
y9
y18*
y19
y10_y10
300
600
900
1200
M/Z
B
Scatterplot of predicted elution time
Fitting R2: 0.873
Novel peptide residual Z score: 1.86
Number of peptides: 1191
Pred. hydrophobicity
60
40
20
0
0
20000
40000
60000
80000
Experimental scan number
C
Distributions of residuals from best−fit line
of predicted RT vs Expt. scan number
Line: Z score of novel peptide
Z: 1.86
count
300
200
100
0
−10
−5
0
5
10
Z scores of residuals from best linear fit
