# Supplementary material for: Splice-Junction-Based Mapping of Alternative Isoforms in the Human Proteome
Source: Cell Rep. Author manuscript; Available in PMC 2020 Jan 15. (PMC6961840; doi:10.1016/j.celrep.2019.11.026)
Supplement: 2 [file NIHMS1546469-supplement-2.zip › DF1/PXD006675/LeftVentricle/LeftVentricle_1_MYBPC3_RTDSHEDTGILDFSSLLK.pdf]

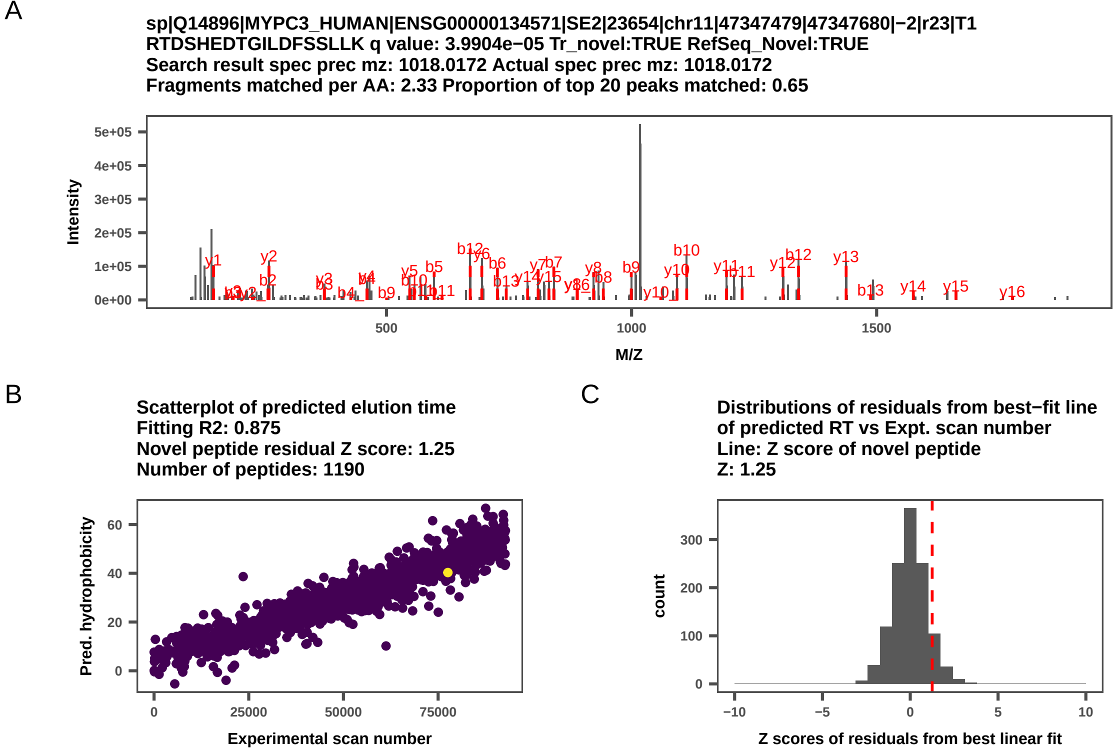
A
sp|Q14896|MYPC3_HUMAN|ENSG00000134571|SE2|23654|chr11|47347479|47347680|−2|r23|T1
RTDSHEDTGILDFSSLLK q value: 3.9904e−05 Tr_novel:TRUE RefSeq_Novel:TRUE
Search result spec prec mz: 1018.0172 Actual spec prec mz: 1018.0172
Fragments matched per AA: 2.33 Proportion of top 20 peaks matched: 0.65
Intensity
M/Z
B
Scatterplot of predicted elution time
Fitting R2: 0.875
Novel peptide residual Z score: 1.25
Number of peptides: 1190
Pred. hydrophobicity
Experimental scan number
C
Distributions of residuals from best−fit line
of predicted RT vs Expt. scan number
Line: Z score of novel peptide
Z: 1.25
count
Z scores of residuals from best linear fit
