# Supplementary material for: Splice-Junction-Based Mapping of Alternative Isoforms in the Human Proteome
Source: Cell Rep. Author manuscript; Available in PMC 2020 Jan 15. (PMC6961840; doi:10.1016/j.celrep.2019.11.026)
Supplement: 2 [file NIHMS1546469-supplement-2.zip › DF1/PXD006675/LeftVentricle/LeftVentricle_5_TNS1_TPLSALGLKPHNPADILLHPTGEEDEGK.pdf]

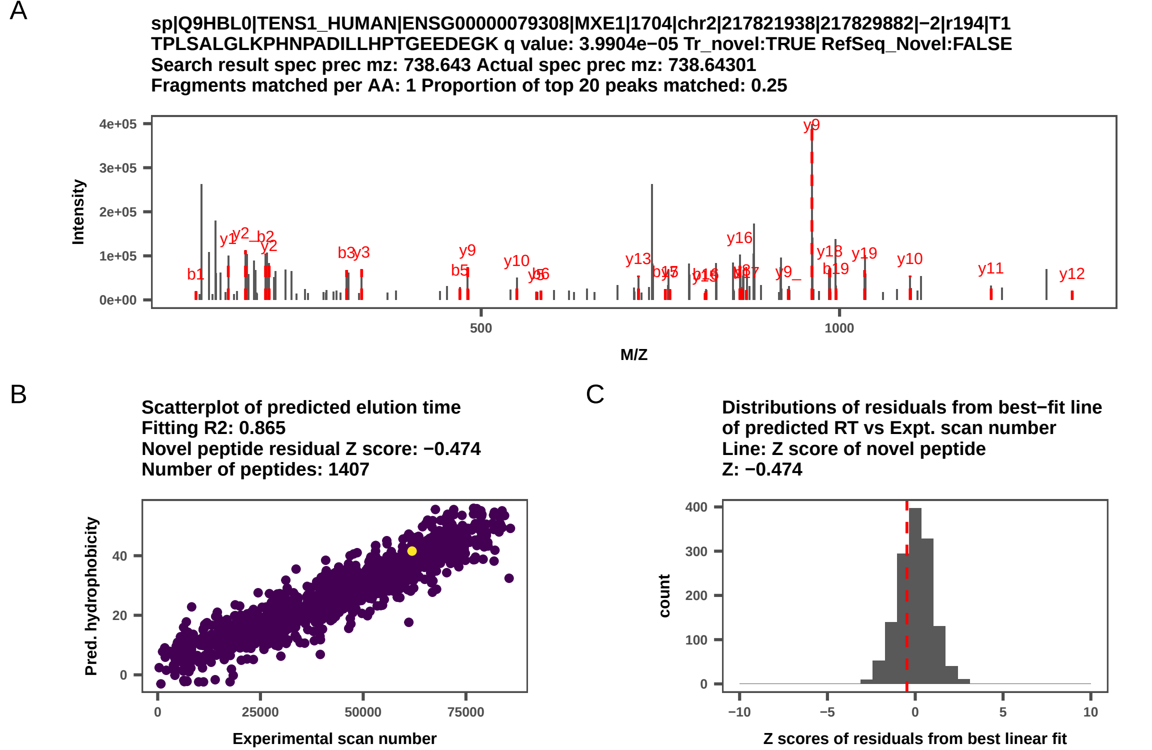
A
sp|Q9HBL0|TENS1_HUMAN|ENSG00000079308|MXE1|1704|chr2|217821938|217829882|−2|r194|T1
TPLSALGLKPHNPADILLHPTGEEDEGK q value: 3.9904e−05 Tr_novel:TRUE RefSeq_Novel:FALSE
Search result spec prec mz: 738.643 Actual spec prec mz: 738.64301
Fragments matched per AA: 1 Proportion of top 20 peaks matched: 0.25
Intensity
4e+05
3e+05
2e+05
1e+05
0e+00
b1
y1
y2
b2
y2
b3
y3
b5
y9
y10
y13
y16
y9
y18
b19
y19
y10
y11
y12
500
1000
M/Z
B
Scatterplot of predicted elution time
Fitting R2: 0.865
Novel peptide residual Z score: −0.474
Number of peptides: 1407
Pred. hydrophobicity
40
20
0
0
25000
50000
75000
Experimental scan number
C
Distributions of residuals from best−fit line
of predicted RT vs Expt. scan number
Line: Z score of novel peptide
Z: −0.474
count
400
300
200
100
0
−10
−5
0
5
10
Z scores of residuals from best linear fit
