# Supplementary material for: Splice-Junction-Based Mapping of Alternative Isoforms in the Human Proteome
Source: Cell Rep. Author manuscript; Available in PMC 2020 Jan 15. (PMC6961840; doi:10.1016/j.celrep.2019.11.026)
Supplement: 2 [file NIHMS1546469-supplement-2.zip › DF1/PXD006675/LeftVentricle/LeftVentricle_26_TPM3_AISEELDHALNDMTSG.pdf]

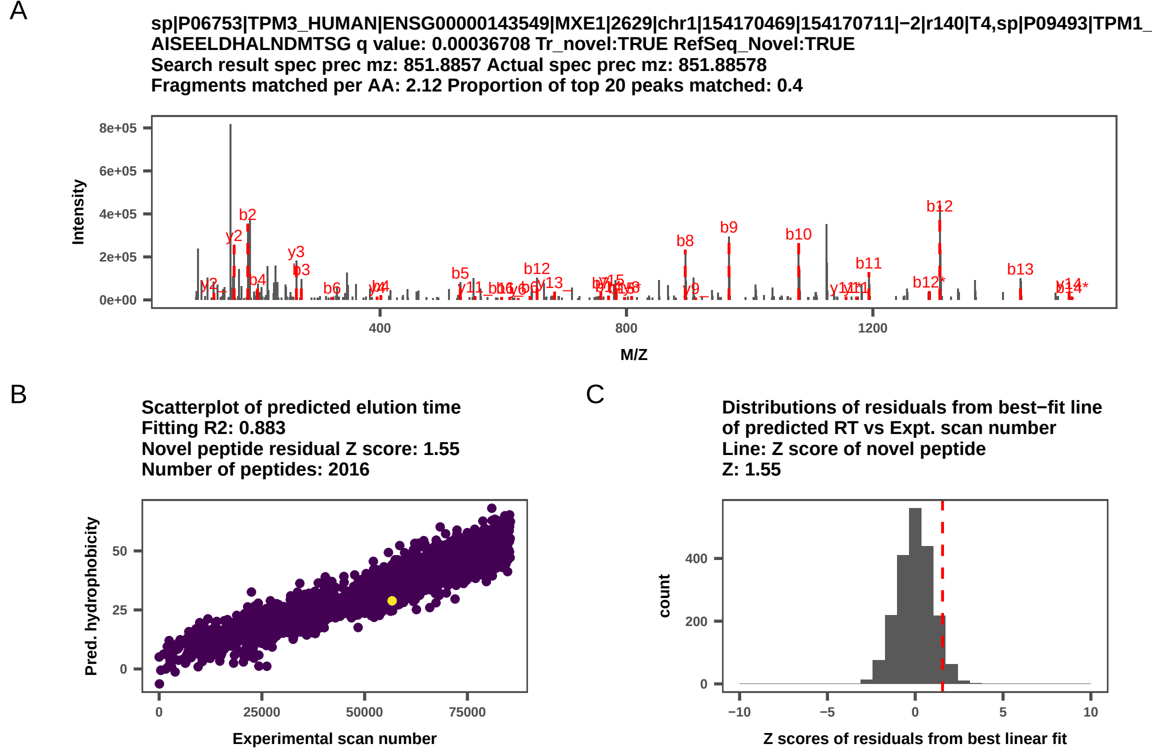
A
sp|P06753|TPM3_HUMAN|ENSG00000143549|MXE1|2629|chr1|154170469|154170711|−2|r140|T4,sp|P09493|TPM1_
AISEELDHALNDMTSG q value: 0.00036708 Tr_novel:TRUE RefSeq_Novel:TRUE
Search result spec prec mz: 851.8857 Actual spec prec mz: 851.88578
Fragments matched per AA: 2.12 Proportion of top 20 peaks matched: 0.4
Intensity
8e+05
6e+05
4e+05
2e+05
0e+00
b2
y2
y3
b3
b4
b5
b6
b8
b9
b10
b11
b12
b12*
b13
400
800
1200
M/Z
B
Scatterplot of predicted elution time
Fitting R2: 0.883
Novel peptide residual Z score: 1.55
Number of peptides: 2016
Pred. hydrophobicity
50
25
0
0
25000
50000
75000
Experimental scan number
C
Distributions of residuals from best−fit line
of predicted RT vs Expt. scan number
Line: Z score of novel peptide
Z: 1.55
count
400
200
0
−10
−5
0
5
10
Z scores of residuals from best linear fit
