# Supplementary material for: Splice-Junction-Based Mapping of Alternative Isoforms in the Human Proteome
Source: Cell Rep. Author manuscript; Available in PMC 2020 Jan 15. (PMC6961840; doi:10.1016/j.celrep.2019.11.026)
Supplement: 2 [file NIHMS1546469-supplement-2.zip › DF1/PXD006675/LeftVentricle/LeftVentricle_38_LDB3_VVVNSPAK.pdf]

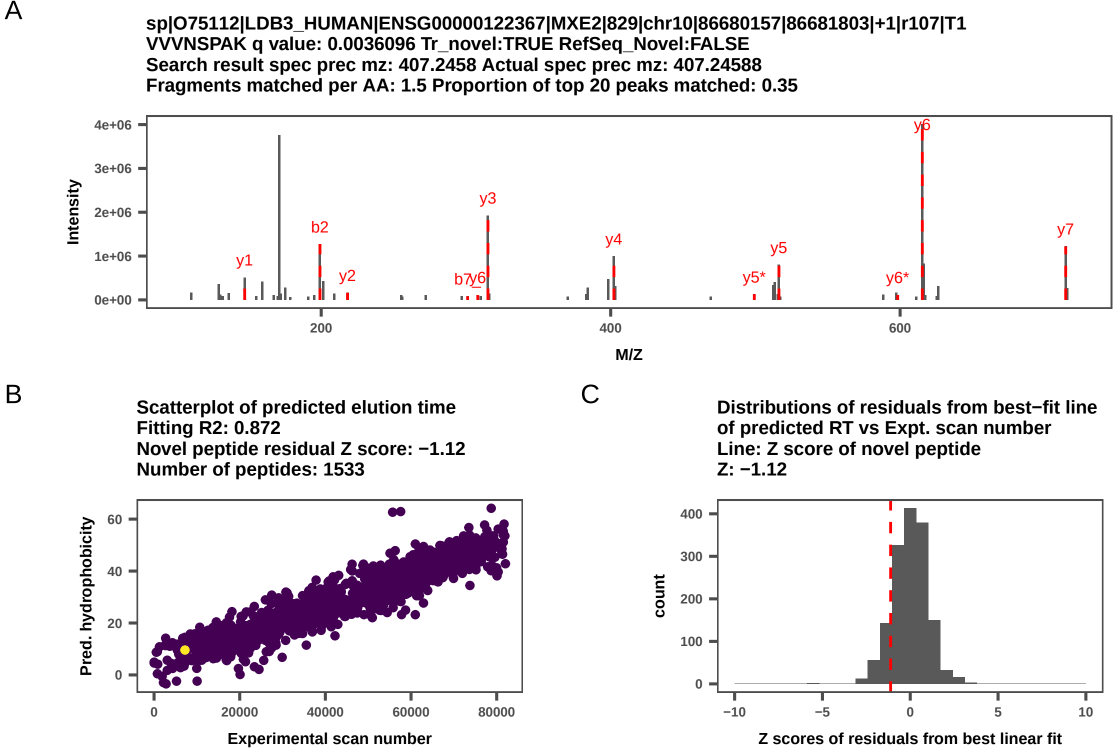
A
sp|O75112|LDB3_HUMAN|ENSG00000122367|MXE2|829|chr10|86680157|86681803|+1|r107|T1
VVVNSPAK q value: 0.0036096 Tr_novel:TRUE RefSeq_Novel:FALSE
Search result spec prec mz: 407.2458 Actual spec prec mz: 407.24588
Fragments matched per AA: 1.5 Proportion of top 20 peaks matched: 0.35
Intensity
4e+06
3e+06
2e+06
1e+06
0e+00
y1
b2
y2
b7_y6
y3
y4
y5*
y5
y6*
y6
y7
200
400
600
M/Z
B
Scatterplot of predicted elution time
Fitting R2: 0.872
Novel peptide residual Z score: −1.12
Number of peptides: 1533
Pred. hydrophobicity
60
40
20
0
0
20000
40000
60000
80000
Experimental scan number
C
Distributions of residuals from best−fit line
of predicted RT vs Expt. scan number
Line: Z score of novel peptide
Z: −1.12
count
400
300
200
100
0
−10
−5
0
5
10
Z scores of residuals from best linear fit
