# Supplementary material for: Splice-Junction-Based Mapping of Alternative Isoforms in the Human Proteome
Source: Cell Rep. Author manuscript; Available in PMC 2020 Jan 15. (PMC6961840; doi:10.1016/j.celrep.2019.11.026)
Supplement: 2 [file NIHMS1546469-supplement-2.zip › DF1/PXD006675/AtrialSeptum/AtrialSeptum_19_TTN_AESPPPEVPGGEK.pdf]

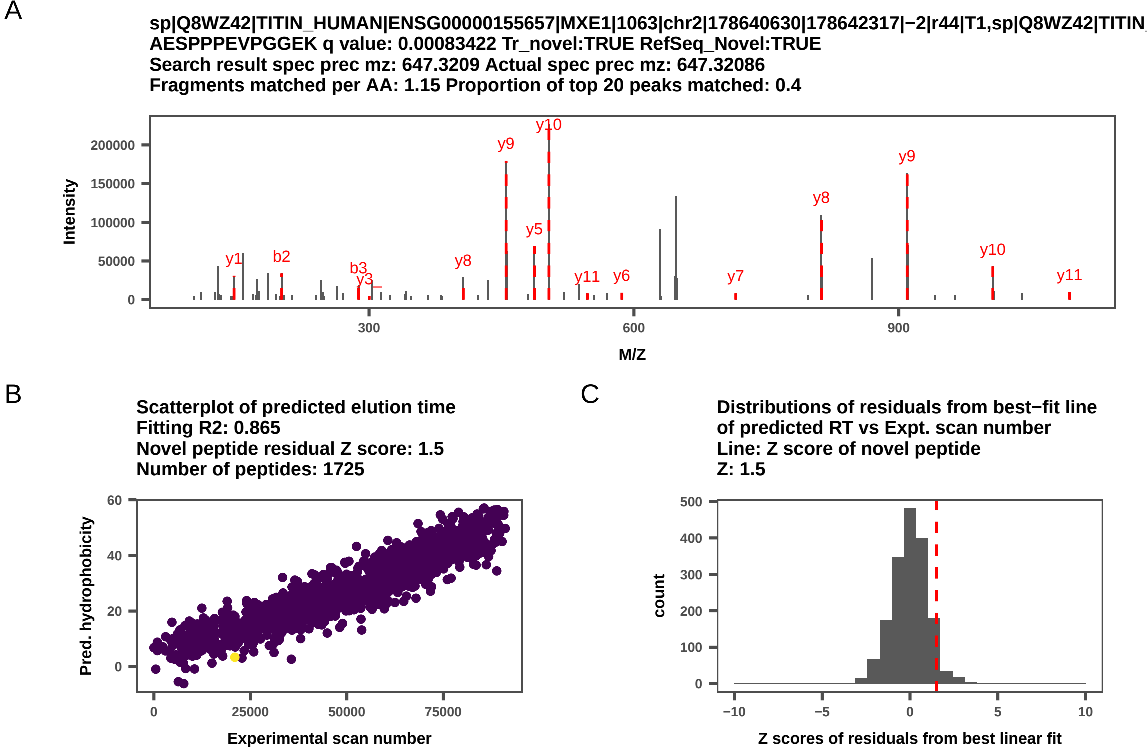
A
sp|Q8WZ42|TITIN_HUMAN|ENSG00000155657|MXE1|1063|chr2|178640630|178642317|−2|r44|T1,sp|Q8WZ42|TITIN_
AESPPPEVPGGEK q value: 0.00083422 Tr_novel:TRUE RefSeq_Novel:TRUE
Search result spec prec mz: 647.3209 Actual spec prec mz: 647.32086
Fragments matched per AA: 1.15 Proportion of top 20 peaks matched: 0.4
Intensity
M/Z
y1
b2
b3
y3
y8
y9
y5
y10
y11
y6
y7
y8
y9
y10
y11
B
Scatterplot of predicted elution time
Fitting R2: 0.865
Novel peptide residual Z score: 1.5
Number of peptides: 1725
Pred. hydrophobicity
Experimental scan number
C
Distributions of residuals from best−fit line
of predicted RT vs Expt. scan number
Line: Z score of novel peptide
Z: 1.5
count
Z scores of residuals from best linear fit
