# Supplementary material for: Splice-Junction-Based Mapping of Alternative Isoforms in the Human Proteome
Source: Cell Rep. Author manuscript; Available in PMC 2020 Jan 15. (PMC6961840; doi:10.1016/j.celrep.2019.11.026)
Supplement: 2 [file NIHMS1546469-supplement-2.zip › DF1/PXD006675/AtrialSeptum/AtrialSeptum_35_ADCK1_VSWTSMALAASGIYFYSNK.pdf]

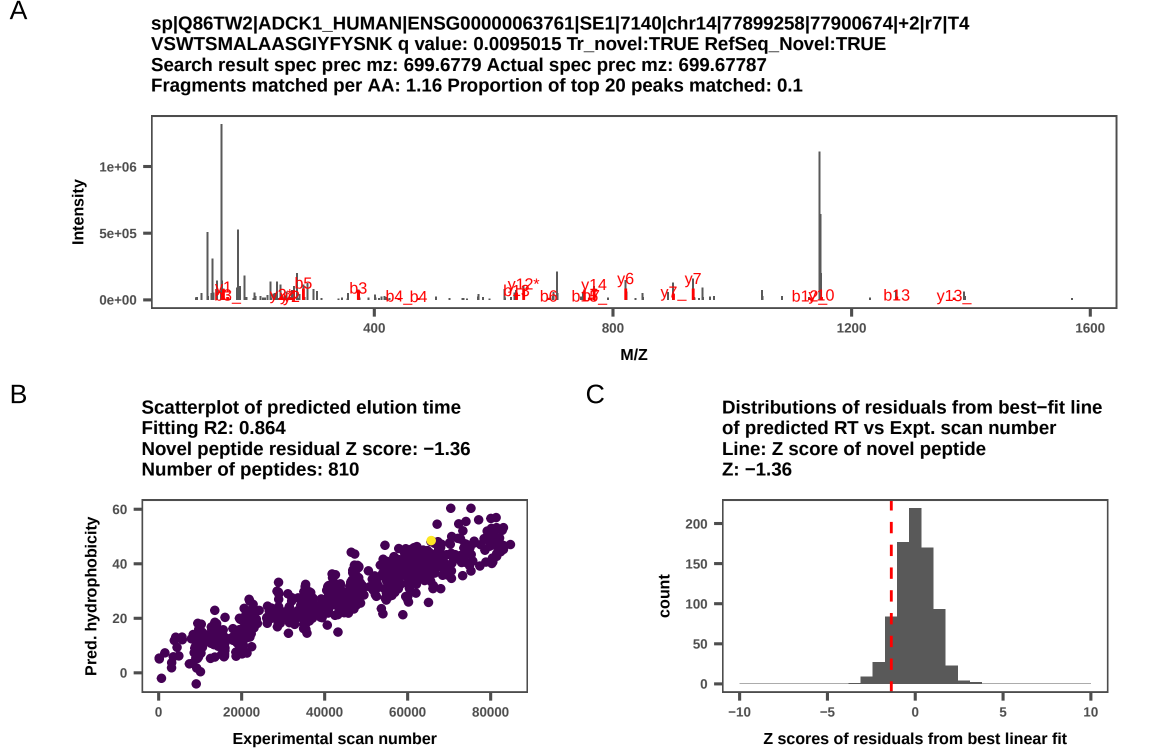
A
sp|Q86TW2|ADCK1_HUMAN|ENSG00000063761|SE1|7140|chr14|77899258|77900674|+2|r7|T4
VSWTSMALAASGIYFYSNK q value: 0.0095015 Tr_novel:TRUE RefSeq_Novel:TRUE
Search result spec prec mz: 699.6779 Actual spec prec mz: 699.67787
Fragments matched per AA: 1.16 Proportion of top 20 peaks matched: 0.1
Intensity
1e+06
5e+05
0e+00
b5
b3
b4_b4
y12*
y14
y6
y7
b6
b13
y13_
M/Z
400
800
1200
1600
B
Scatterplot of predicted elution time
Fitting R2: 0.864
Novel peptide residual Z score: −1.36
Number of peptides: 810
Pred. hydrophobicity
60
40
20
0
0
20000
40000
60000
80000
Experimental scan number
C
Distributions of residuals from best−fit line
of predicted RT vs Expt. scan number
Line: Z score of novel peptide
Z: −1.36
count
200
150
100
50
0
−10
−5
0
5
10
Z scores of residuals from best linear fit
