# Supplementary material for: Splice-Junction-Based Mapping of Alternative Isoforms in the Human Proteome
Source: Cell Rep. Author manuscript; Available in PMC 2020 Jan 15. (PMC6961840; doi:10.1016/j.celrep.2019.11.026)
Supplement: 2 [file NIHMS1546469-supplement-2.zip › DF1/PXD006675/AtrialSeptum/AtrialSeptum_5_TTN_SNCTVSVHVSEPPR.pdf]

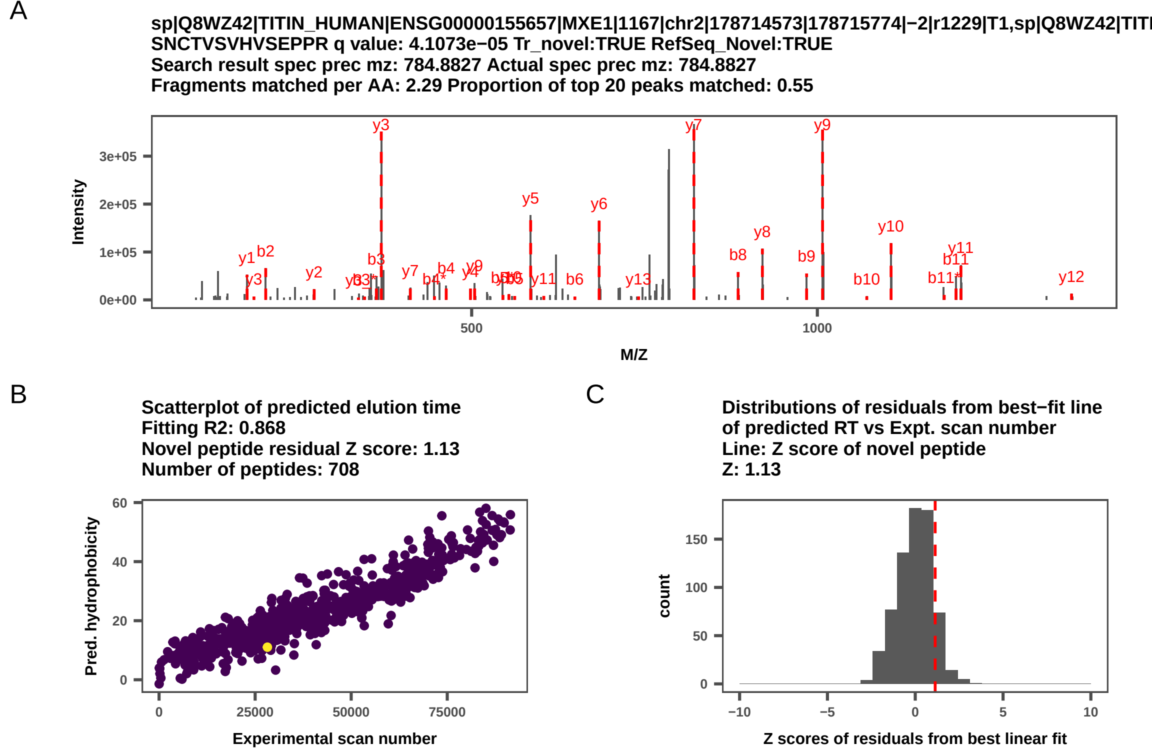
A
sp|Q8WZ42|TITIN_HUMAN|ENSG00000155657|MXE1|1167|chr2|178714573|178715774|−2|r1229|T1,sp|Q8WZ42|TITI
SNCTVSVHVSEPPR q value: 4.1073e−05 Tr_novel:TRUE RefSeq_Novel:TRUE
Search result spec prec mz: 784.8827 Actual spec prec mz: 784.8827
Fragments matched per AA: 2.29 Proportion of top 20 peaks matched: 0.55
Intensity
3e+05
2e+05
1e+05
0e+00
y3
y7
y9
y5
y6
y8
y10
y11
b11
b8
b9
b10
b11*
y12
y13
b6
y11
y1
b2
y3
y2
b3
y7
b4
b4*
y9
y4
M/Z
500
1000
B
Scatterplot of predicted elution time
Fitting R2: 0.868
Novel peptide residual Z score: 1.13
Number of peptides: 708
Pred. hydrophobicity
60
40
20
0
0
25000
50000
75000
Experimental scan number
C
Distributions of residuals from best−fit line
of predicted RT vs Expt. scan number
Line: Z score of novel peptide
Z: 1.13
count
150
100
50
0
−10
−5
0
5
10
Z scores of residuals from best linear fit
