# Supplementary material for: Splice-Junction-Based Mapping of Alternative Isoforms in the Human Proteome
Source: Cell Rep. Author manuscript; Available in PMC 2020 Jan 15. (PMC6961840; doi:10.1016/j.celrep.2019.11.026)
Supplement: 2 [file NIHMS1546469-supplement-2.zip › DF1/PXD006675/AtrialSeptum/AtrialSeptum_36_MAP3K3_IASNIMNDLVALQMNR.pdf]

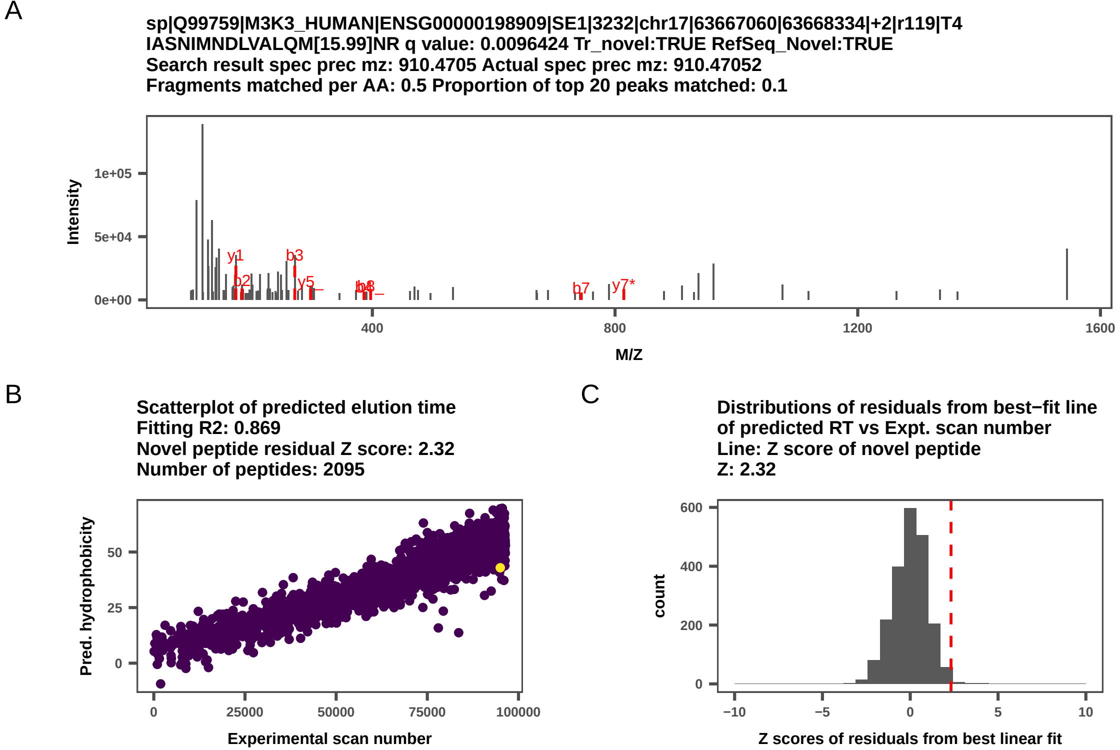
A
sp|Q99759|M3K3_HUMAN|ENSG00000198909|SE1|3232|chr17|63667060|63668334|+2|r119|T4
IASNIMNDLVALQM[15.99]NR q value: 0.0096424 Tr_novel:TRUE RefSeq_Novel:TRUE
Search result spec prec mz: 910.4705 Actual spec prec mz: 910.47052
Fragments matched per AA: 0.5 Proportion of top 20 peaks matched: 0.1
Intensity
1e+05
5e+04
0e+00
y1
b3
b2
y5
b8
b7
y7*
400
800
1200
1600
M/Z
B
Scatterplot of predicted elution time
Fitting R2: 0.869
Novel peptide residual Z score: 2.32
Number of peptides: 2095
Pred. hydrophobicity
50
25
0
0
25000
50000
75000
100000
Experimental scan number
C
Distributions of residuals from best−fit line
of predicted RT vs Expt. scan number
Line: Z score of novel peptide
Z: 2.32
count
600
400
200
0
−10
−5
0
5
10
Z scores of residuals from best linear fit
