# Supplementary material for: Splice-Junction-Based Mapping of Alternative Isoforms in the Human Proteome
Source: Cell Rep. Author manuscript; Available in PMC 2020 Jan 15. (PMC6961840; doi:10.1016/j.celrep.2019.11.026)
Supplement: 2 [file NIHMS1546469-supplement-2.zip › DF1/PXD006675/AtrialSeptum/AtrialSeptum_17_NRAP_LNALHLSDFR.pdf]

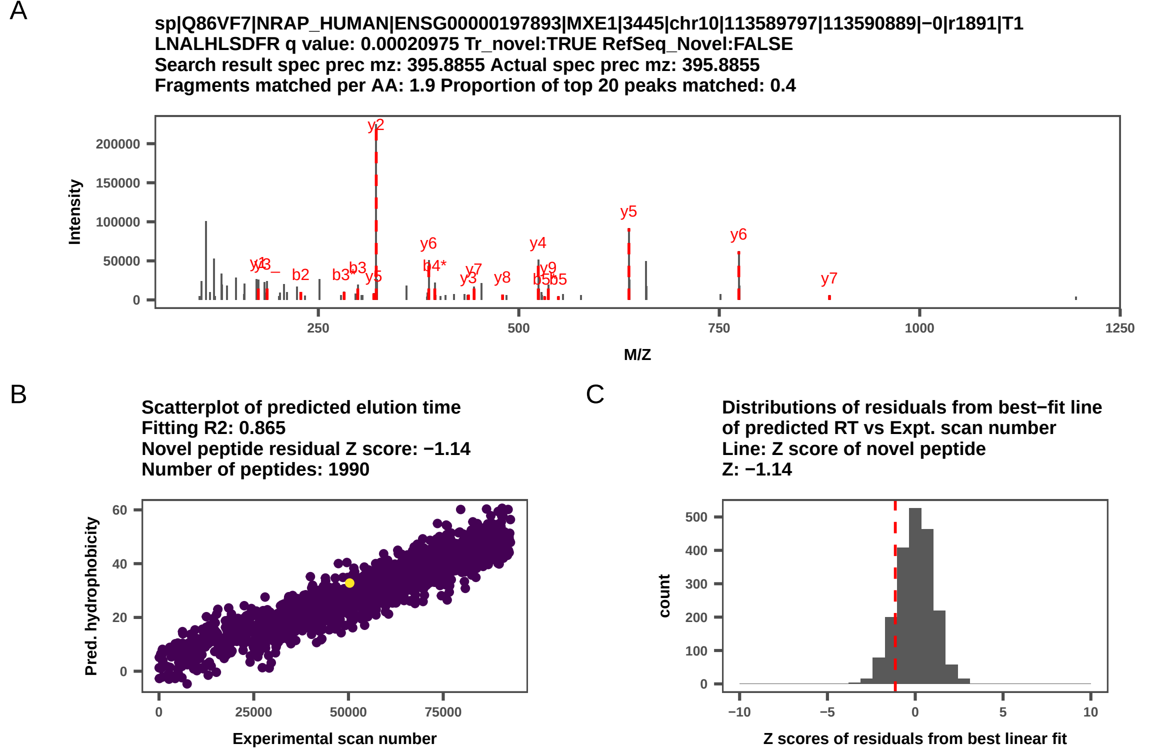
A
sp|Q86VF7|NRAP_HUMAN|ENSG00000197893|MXE1|3445|chr10|113589797|113590889|−0|r1891|T1
LNALHLSDFR q value: 0.00020975 Tr_novel:TRUE RefSeq_Novel:FALSE
Search result spec prec mz: 395.8855 Actual spec prec mz: 395.8855
Fragments matched per AA: 1.9 Proportion of top 20 peaks matched: 0.4
Intensity
200000
150000
100000
50000
0
y2
y5
y6
y4
y6
y7
b2
b3
b3*
b4*
y5
y7
y3
y8
y9
b5
b5
250
500
750
1000
1250
M/Z
B
Scatterplot of predicted elution time
Fitting R2: 0.865
Novel peptide residual Z score: −1.14
Number of peptides: 1990
Pred. hydrophobicity
60
40
20
0
0
25000
50000
75000
Experimental scan number
C
Distributions of residuals from best−fit line
of predicted RT vs Expt. scan number
Line: Z score of novel peptide
Z: −1.14
count
500
400
300
200
100
0
−10
−5
0
5
10
Z scores of residuals from best linear fit
