# Supplementary material for: Splice-Junction-Based Mapping of Alternative Isoforms in the Human Proteome
Source: Cell Rep. Author manuscript; Available in PMC 2020 Jan 15. (PMC6961840; doi:10.1016/j.celrep.2019.11.026)
Supplement: 2 [file NIHMS1546469-supplement-2.zip › DF1/PXD006675/AtrialSeptum/AtrialSeptum_26_ABCG1_TVGWVGTAMNASSYSAEMTEPK.pdf]

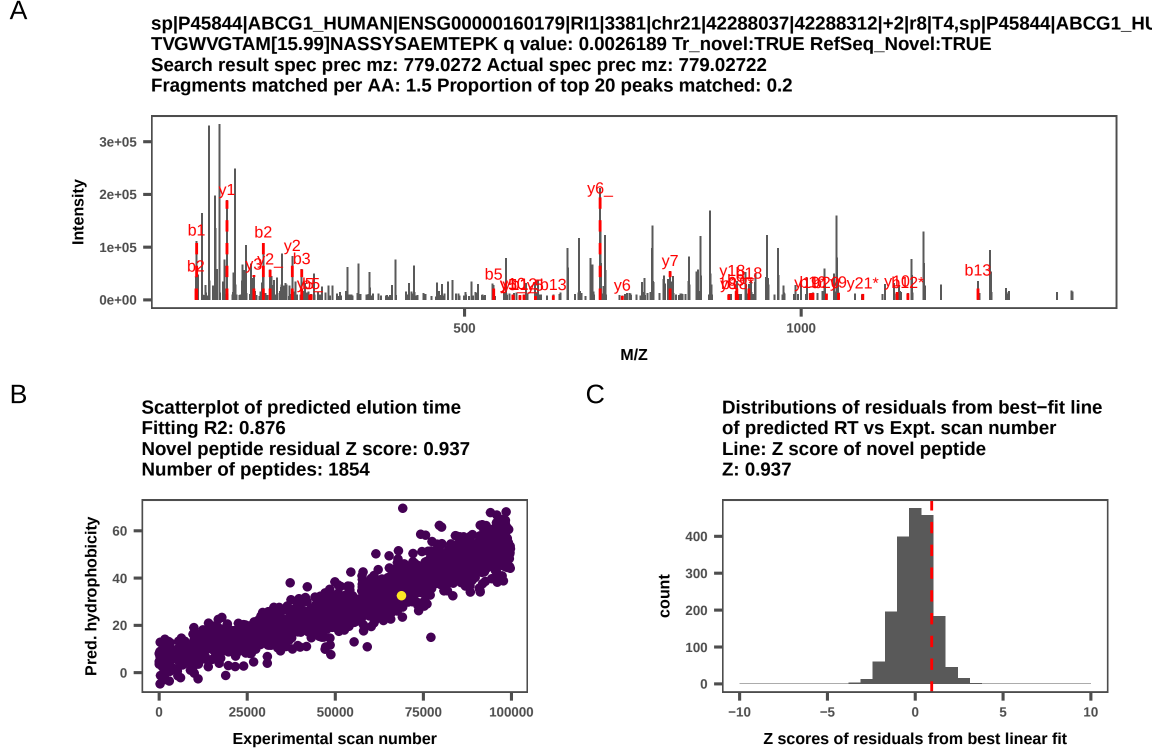
A
sp|P45844|ABCG1_HUMAN|ENSG00000160179|RI1|3381|chr21|42288037|42288312|+2|r8|T4,sp|P45844|ABCG1_HU
TVGWVGTAM[15.99]NASSYSAEMTEPK q value: 0.0026189 Tr_novel:TRUE RefSeq_Novel:TRUE
Search result spec prec mz: 779.0272 Actual spec prec mz: 779.02722
Fragments matched per AA: 1.5 Proportion of top 20 peaks matched: 0.2
Intensity
3e+05
2e+05
1e+05
0e+00
b1
b2
y1
y3
y2_
y2
b3
b5
b13
y6_
y6
y7
b13
y21*
500
1000
M/Z
B
Scatterplot of predicted elution time
Fitting R2: 0.876
Novel peptide residual Z score: 0.937
Number of peptides: 1854
Pred. hydrophobicity
60
40
20
0
0
25000
50000
75000
100000
Experimental scan number
C
Distributions of residuals from best−fit line
of predicted RT vs Expt. scan number
Line: Z score of novel peptide
Z: 0.937
count
400
300
200
100
0
−10
−5
0
5
10
Z scores of residuals from best linear fit
