# Supplementary material for: Splice-Junction-Based Mapping of Alternative Isoforms in the Human Proteome
Source: Cell Rep. Author manuscript; Available in PMC 2020 Jan 15. (PMC6961840; doi:10.1016/j.celrep.2019.11.026)
Supplement: 3 [file NIHMS1546469-supplement-3.zip › DF2/PXD000561/Prostate-61-Q8WWI1-SWASPVYTEADGTFSR.pdf]

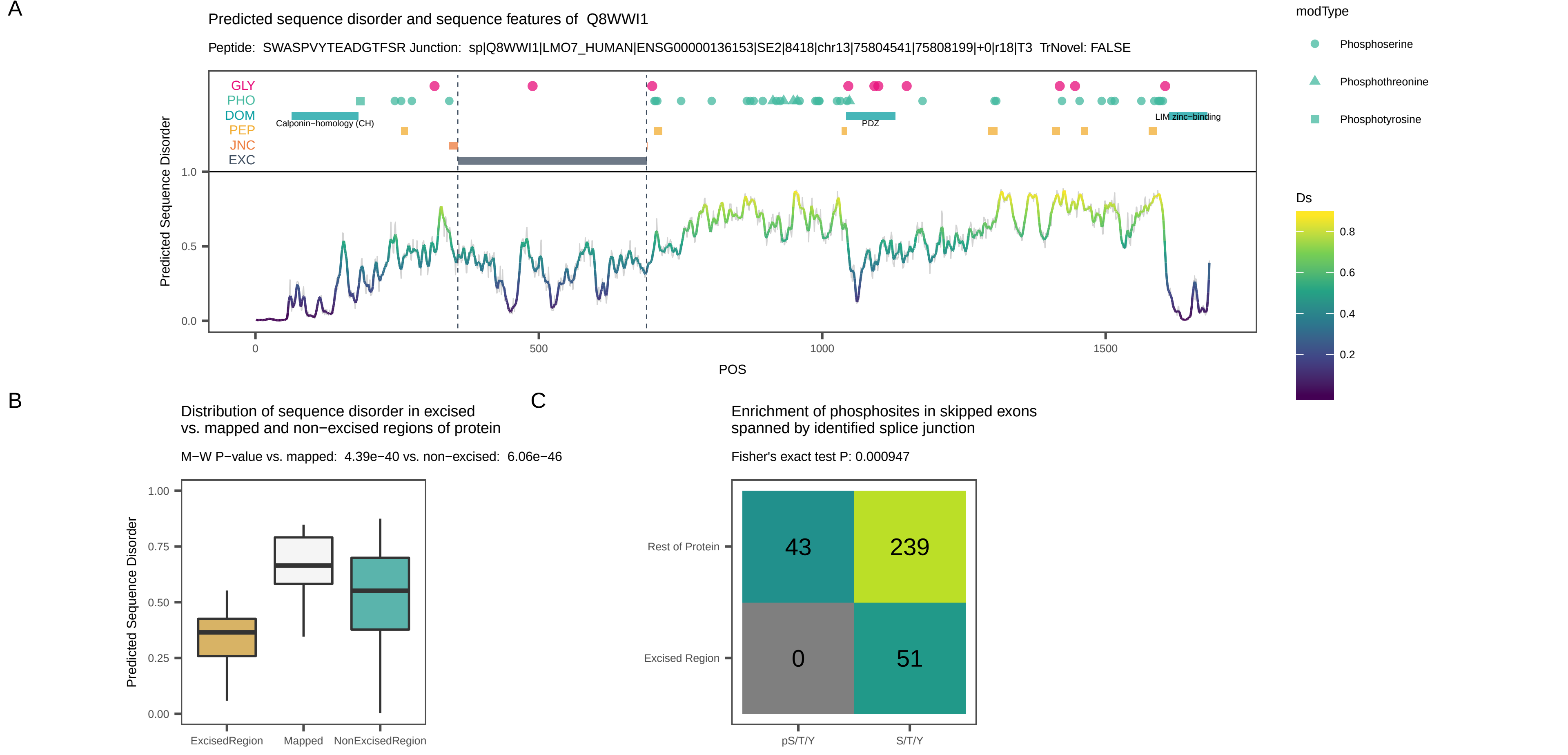
A
Predicted sequence disorder and sequence features of Q8WWI1
Peptide: SWASPVYTEADGTFSR Junction: sp|Q8WWI1|LMO7_HUMAN|ENSG00000136153|SE2|8418|chr13|75804541|75808199|+0|r18|T3 TrNovel: FALSE
GLY
PHO
DOM
PEP
JNC
EXC
Calponin−homology (CH)
PDZ
LIM zinc−binding
Predicted Sequence Disorder
POS
modType
Phosphoserine
Phosphothreonine
Phosphotyrosine
Ds
B
Distribution of sequence disorder in excised vs. mapped and non−excised regions of protein
M−W P−value vs. mapped: 4.39e−40 vs. non−excised: 6.06e−46
Predicted Sequence Disorder
ExcisedRegion
Mapped
NonExcisedRegion
C
Enrichment of phosphosites in skipped exons spanned by identified splice junction
Fisher's exact test P: 0.000947
Rest of Protein
Excised Region
43
239
0
51
pS/T/Y
S/T/Y
