# Supplementary material for: Splice-Junction-Based Mapping of Alternative Isoforms in the Human Proteome
Source: Cell Rep. Author manuscript; Available in PMC 2020 Jan 15. (PMC6961840; doi:10.1016/j.celrep.2019.11.026)
Supplement: 3 [file NIHMS1546469-supplement-3.zip › DF2/PXD000561/Prostate-58-Q9BV36-ASSESQGLGAGVR.pdf]

A

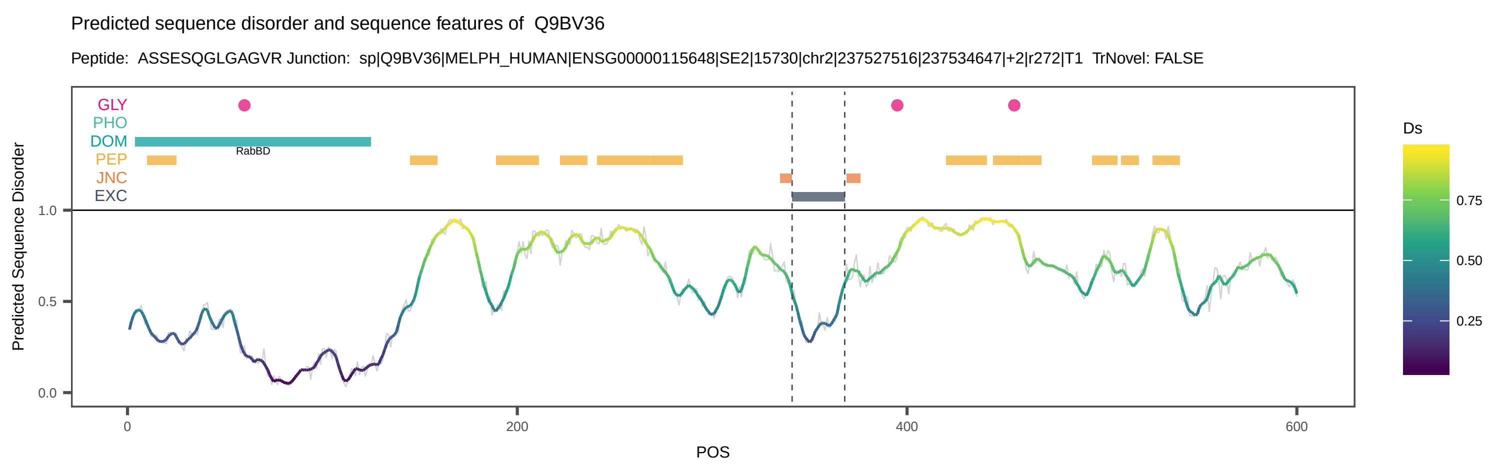

Predicted sequence disorder and sequence features of Q9BV36
Peptide: ASSESQGLGAGVR Junction: sp|Q9BV36|MELPH_HUMAN|ENSG00000115648|SE2|15730|chr2|237527516|237534647|+2|r272|T1 TrNovel: FALSE
GLY
PHO
DOM
PEP
JNC
EXC
RabBD
Predicted Sequence Disorder
1.0
0.5
0.0
0
200
400
600
POS
Ds
0.75
0.50
0.25

B

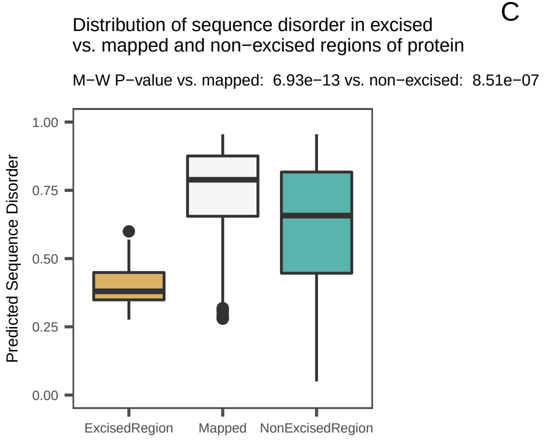

Distribution of sequence disorder in excised
vs. mapped and non−excised regions of protein
M−W P−value vs. mapped: 6.93e−13 vs. non−excised: 8.51e−07
Predicted Sequence Disorder
1.00
0.75
0.50
0.25
0.00
ExcisedRegion
Mapped
NonExcisedRegion

C
