# Supplementary material for: Splice-Junction-Based Mapping of Alternative Isoforms in the Human Proteome
Source: Cell Rep. Author manuscript; Available in PMC 2020 Jan 15. (PMC6961840; doi:10.1016/j.celrep.2019.11.026)
Supplement: 3 [file NIHMS1546469-supplement-3.zip › DF2/PXD000561/Testis-114-Q96P26-LSNQGSQESSLR.pdf]

A

Predicted sequence disorder and sequence features of Q96P26

Peptide: LSNQGSQESSLR Junction: sp|Q96P26|5NT1B_HUMAN|ENSG00000185013|SE2|30935|chr2|18586391|18587173|−0|r33|T1 TrNovel: FALSE

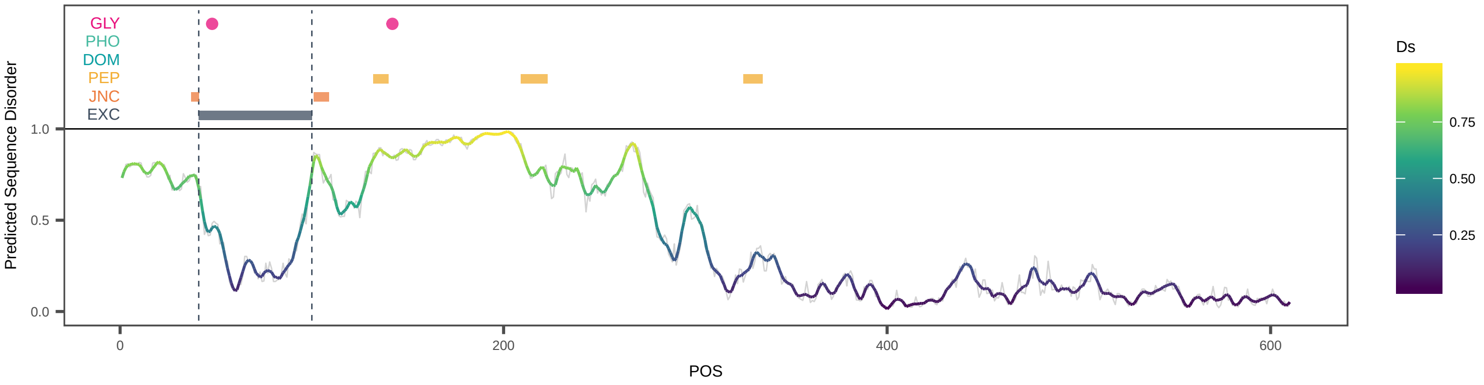

B

Distribution of sequence disorder in excised vs. mapped and non−excised regions of protein

M−W P−value vs. mapped: 1.39e−07 vs. non−excised: 0.513

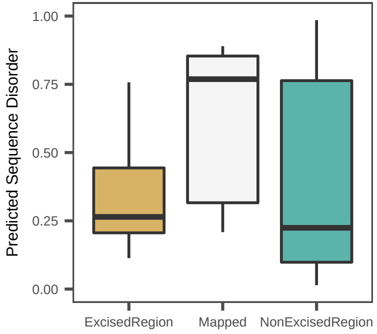

C
