# Supplementary material for: Splice-Junction-Based Mapping of Alternative Isoforms in the Human Proteome
Source: Cell Rep. Author manuscript; Available in PMC 2020 Jan 15. (PMC6961840; doi:10.1016/j.celrep.2019.11.026)
Supplement: 3 [file NIHMS1546469-supplement-3.zip › DF2/PXD000561/Ovary-39-Q8TAM2-TALDLAALSTEHSQYK.pdf]

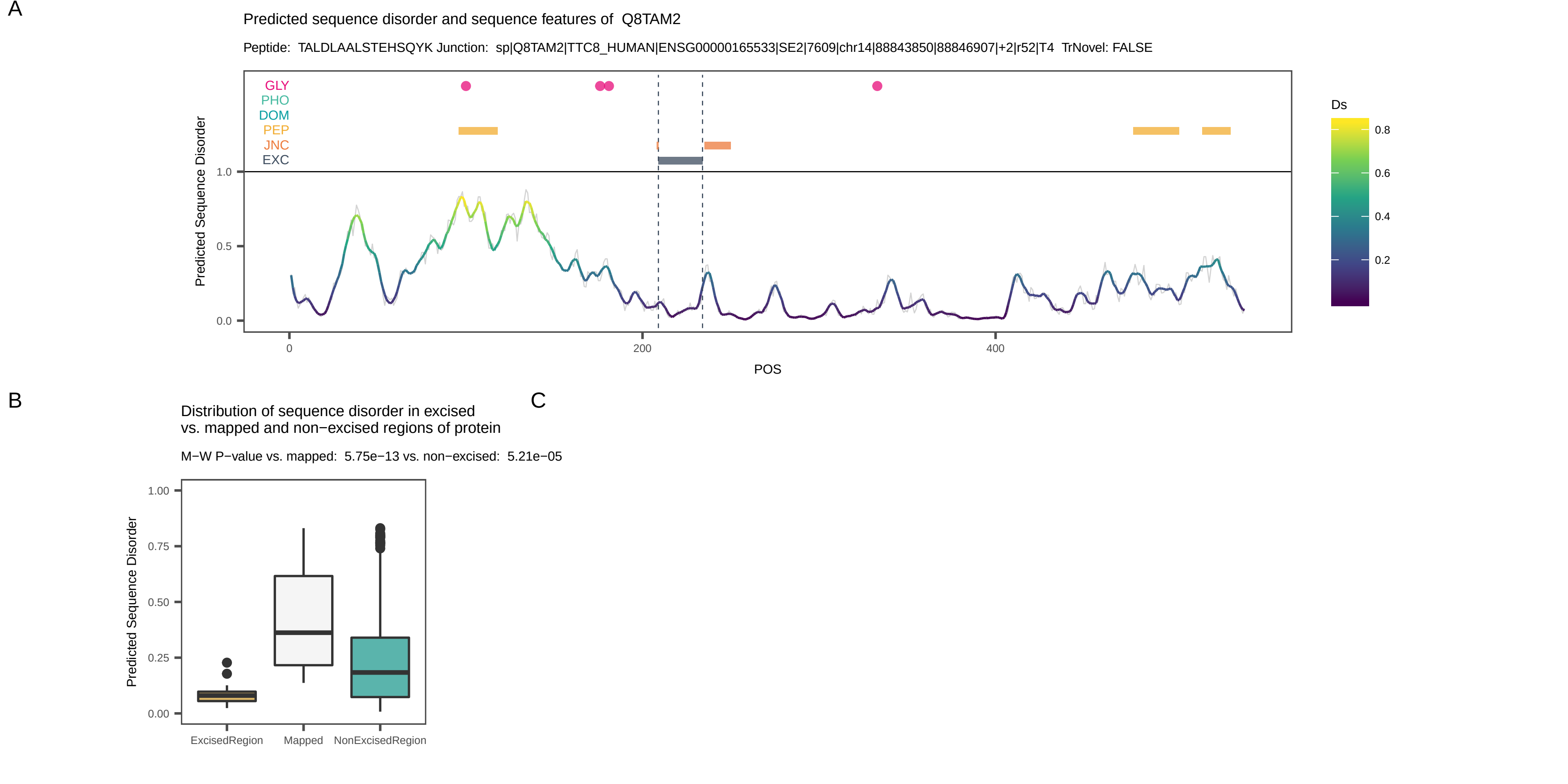
A
Predicted sequence disorder and sequence features of Q8TAM2
Peptide: TALDLAALSTEHSQYK Junction: sp|Q8TAM2|TTC8_HUMAN|ENSG00000165533|SE2|7609|chr14|88843850|88846907|+2|r52|T4 TrNovel: FALSE
GLY
PHO
DOM
PEP
JNC
EXC
Predicted Sequence Disorder
1.0
0.5
0.0
0
200
400
POS
Ds
0.8
0.6
0.4
0.2
B
Distribution of sequence disorder in excised vs. mapped and non−excised regions of protein
M−W P−value vs. mapped: 5.75e−13 vs. non−excised: 5.21e−05
Predicted Sequence Disorder
1.00
0.75
0.50
0.25
0.00
ExcisedRegion
Mapped
NonExcisedRegion
C
