# Supplementary material for: Splice-Junction-Based Mapping of Alternative Isoforms in the Human Proteome
Source: Cell Rep. Author manuscript; Available in PMC 2020 Jan 15. (PMC6961840; doi:10.1016/j.celrep.2019.11.026)
Supplement: 3 [file NIHMS1546469-supplement-3.zip › DF2/PXD000561/Testis-74-P28370-LDGQTPHEEREEAIEAFNAPNSSK.pdf]

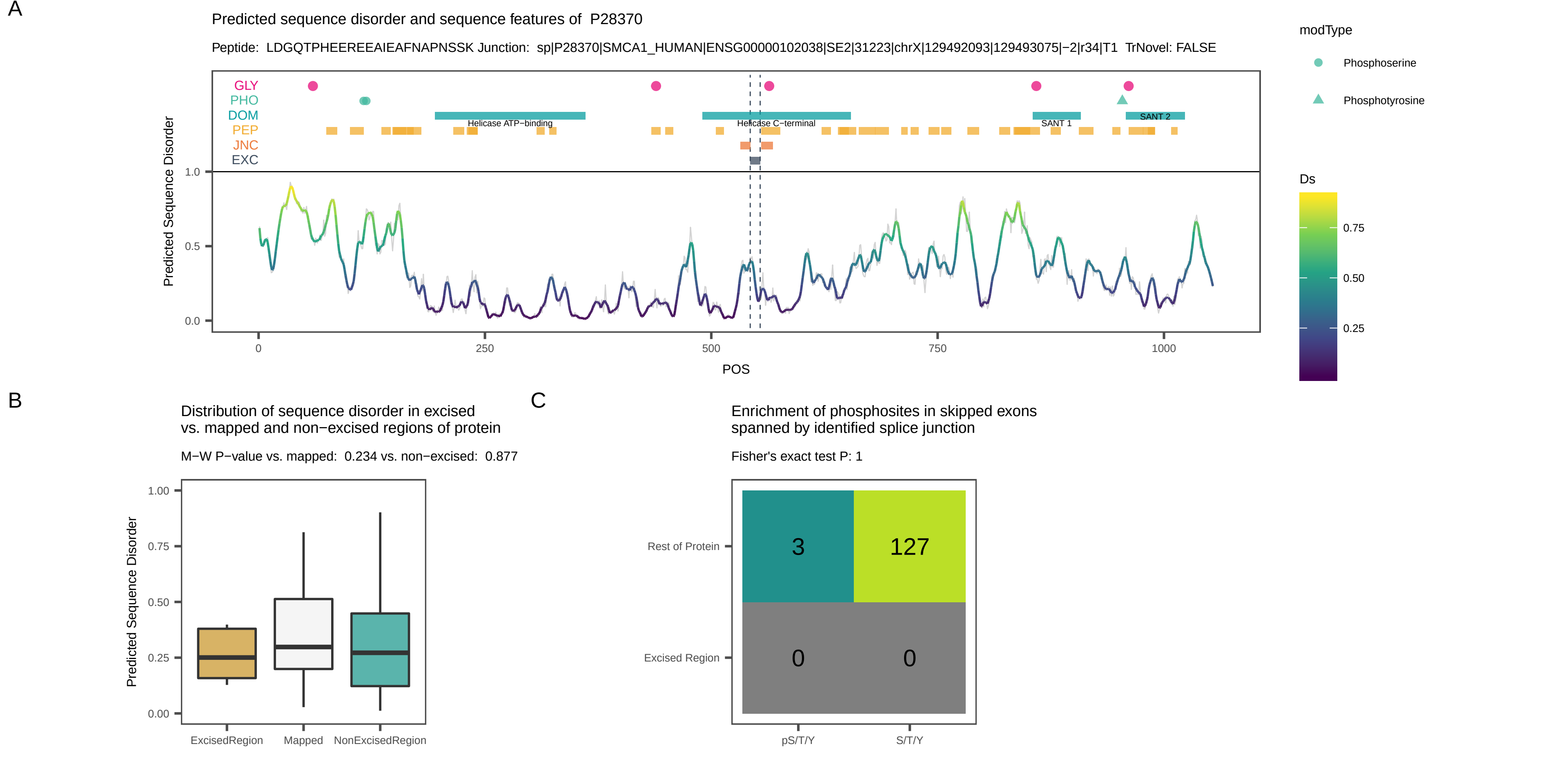

A
Predicted sequence disorder and sequence features of P28370
Peptide: LDGQTPHEEREEAIEAFNAPNSSK Junction: sp|P28370|SMCA1_HUMAN|ENSG00000102038|SE2|31223|chrX|129492093|129493075|−2|r34|T1 TrNovel: FALSE
GLY
PHO
DOM
PEP
JNC
EXC
Helicase ATP−binding
Helicase C−terminal
SANT 1
SANT 2
Predicted Sequence Disorder
1.0
0.5
0.0
0
250
500
750
1000
POS
modType
Phosphoserine
Phosphotyrosine
Ds
0.75
0.50
0.25
B
Distribution of sequence disorder in excised
vs. mapped and non−excised regions of protein
M−W P−value vs. mapped: 0.234 vs. non−excised: 0.877
Predicted Sequence Disorder
1.00
0.75
0.50
0.25
0.00
ExcisedRegion
Mapped
NonExcisedRegion
C
Enrichment of phosphosites in skipped exons
spanned by identified splice junction
Fisher's exact test P: 1
Rest of Protein
Excised Region
3
127
0
0
pS/T/Y
S/T/Y
