# Supplementary material for: Splice-Junction-Based Mapping of Alternative Isoforms in the Human Proteome
Source: Cell Rep. Author manuscript; Available in PMC 2020 Jan 15. (PMC6961840; doi:10.1016/j.celrep.2019.11.026)
Supplement: 3 [file NIHMS1546469-supplement-3.zip › DF2/PXD000561/Ovary-24-O60271-SHTSLKDELSDVSQGGSK.pdf]

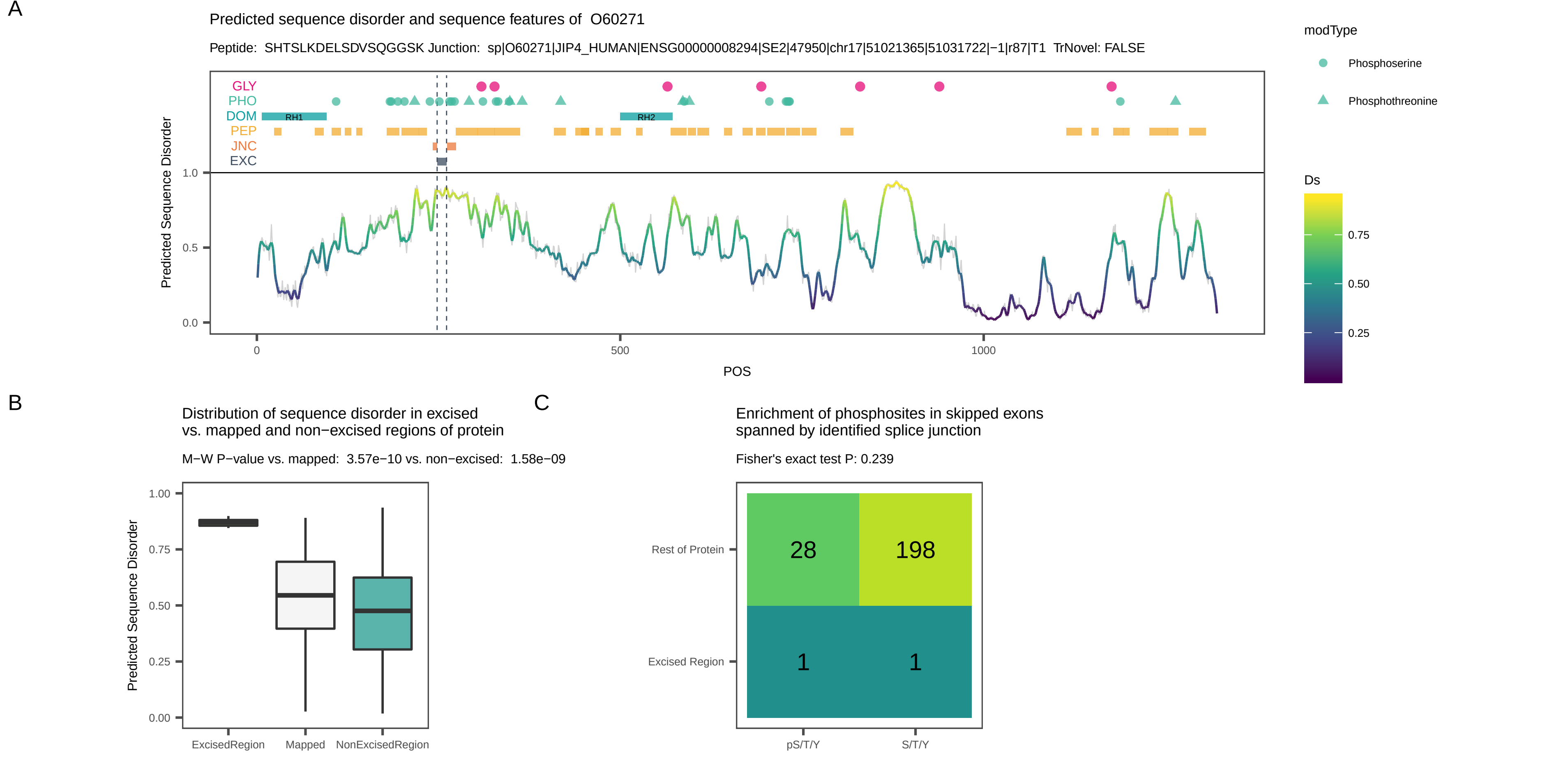
A
Predicted sequence disorder and sequence features of O60271
Peptide: SHTSLKDELSDVSQGGSK Junction: sp|O60271|JIP4_HUMAN|ENSG00000008294|SE2|47950|chr17|51021365|51031722|−1|r87|T1 TrNovel: FALSE
GLY
PHO
DOM
PEP
JNC
EXC
RH1
RH2
Predicted Sequence Disorder
1.0
0.5
0.0
0
500
1000
POS
modType
Phosphoserine
Phosphothreonine
Ds
0.75
0.50
0.25
B
Distribution of sequence disorder in excised
vs. mapped and non−excised regions of protein
M−W P−value vs. mapped: 3.57e−10 vs. non−excised: 1.58e−09
Predicted Sequence Disorder
1.00
0.75
0.50
0.25
0.00
ExcisedRegion
Mapped
NonExcisedRegion
C
Enrichment of phosphosites in skipped exons
spanned by identified splice junction
Fisher's exact test P: 0.239
Rest of Protein
Excised Region
28
198
1
1
pS/T/Y
S/T/Y
