# Supplementary material for: Splice-Junction-Based Mapping of Alternative Isoforms in the Human Proteome
Source: Cell Rep. Author manuscript; Available in PMC 2020 Jan 15. (PMC6961840; doi:10.1016/j.celrep.2019.11.026)
Supplement: 3 [file NIHMS1546469-supplement-3.zip › DF2/PXD000561/Lung-43-P08133-EDAQEIADTPSGDKTSLETR.pdf]

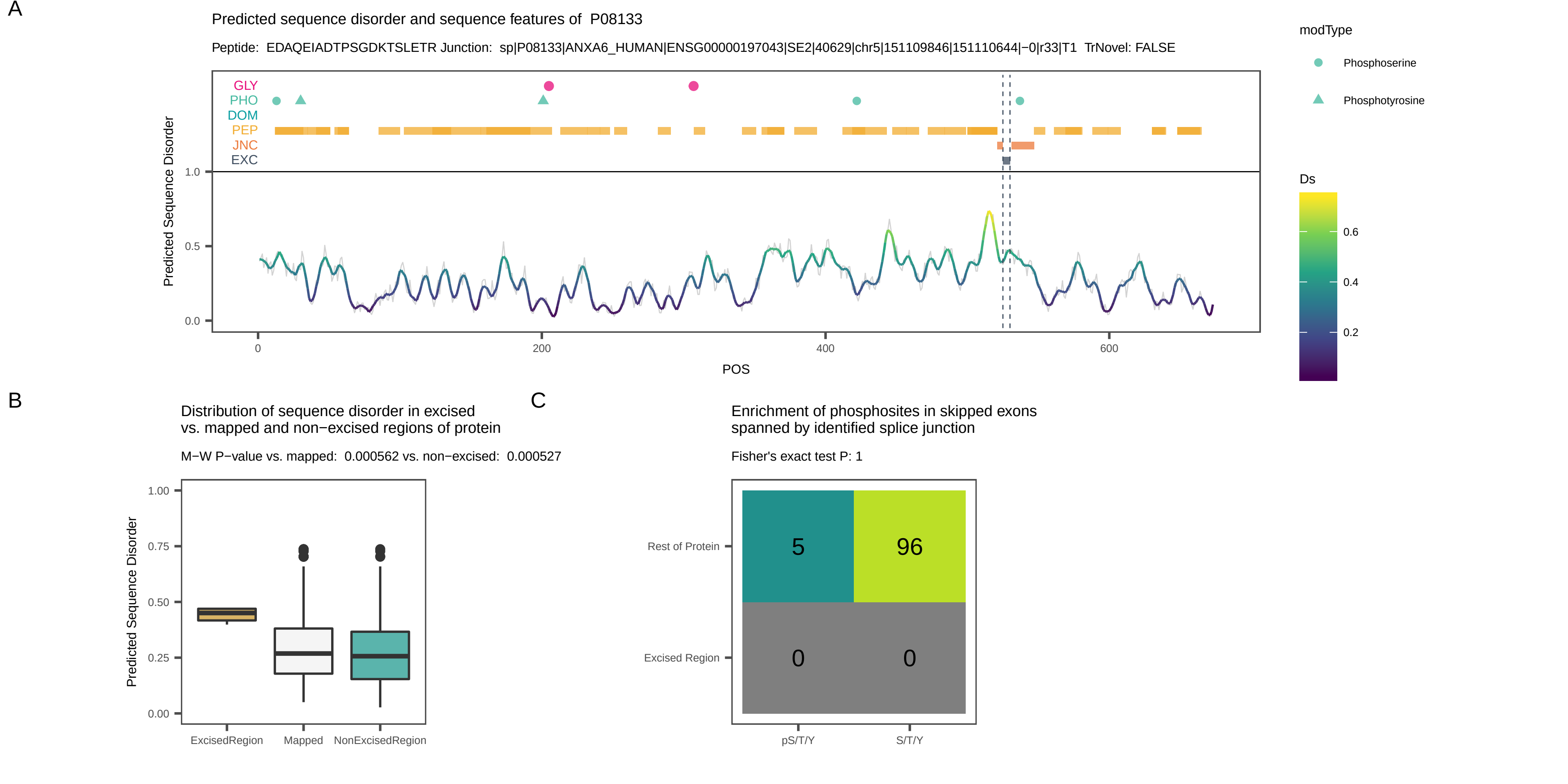

A
Predicted sequence disorder and sequence features of P08133
Peptide: EDAQEIADTPSGDKTSLETR Junction: sp|P08133|ANXA6_HUMAN|ENSG00000197043|SE2|40629|chr5|151109846|151110644|−0|r33|T1 TrNovel: FALSE
GLY
PHO
DOM
PEP
JNC
EXC
Predicted Sequence Disorder
1.0
0.5
0.0
0
200
400
600
POS
modType
Phosphoserine
Phosphotyrosine
Ds
0.6
0.4
0.2
B
Distribution of sequence disorder in excised
vs. mapped and non−excised regions of protein
M−W P−value vs. mapped: 0.000562 vs. non−excised: 0.000527
Predicted Sequence Disorder
1.00
0.75
0.50
0.25
0.00
ExcisedRegion
Mapped
NonExcisedRegion
C
Enrichment of phosphosites in skipped exons
spanned by identified splice junction
Fisher's exact test P: 1
Rest of Protein
Excised Region
5
96
0
0
pS/T/Y
S/T/Y
