# Supplementary material for: Splice-Junction-Based Mapping of Alternative Isoforms in the Human Proteome
Source: Cell Rep. Author manuscript; Available in PMC 2020 Jan 15. (PMC6961840; doi:10.1016/j.celrep.2019.11.026)
Supplement: 3 [file NIHMS1546469-supplement-3.zip › DF2/PXD000561/Heart-186-Q8WXS3-SAAAPDSGPEAGGLHSGMLEDGLPSNGVPR.pdf]

A

Predicted sequence disorder and sequence features of Q8WXS3

Peptide: SAAAPDSGPEAGGLHSGMLEDGLPSNGVPR Junction: sp|Q8WXS3|BAALC_HUMAN|ENSG00000164929|SE2|14886|chr8|103141057|103200762|+0|r9|T1 TrNovel: FALSE

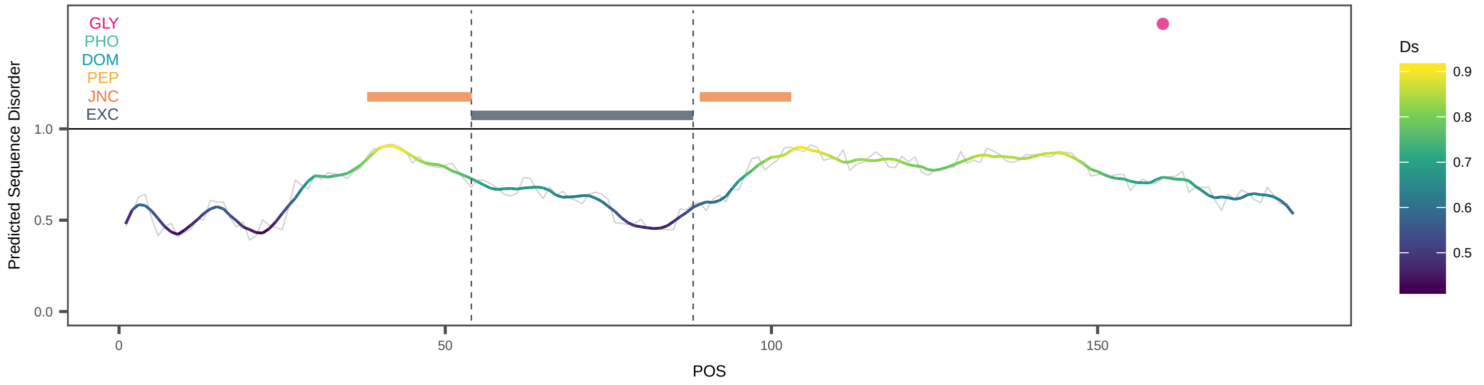

B

Distribution of sequence disorder in excised
vs. mapped and non−excised regions of protein

M−W P−value vs. mapped: NA vs. non−excised: 4.19e−07

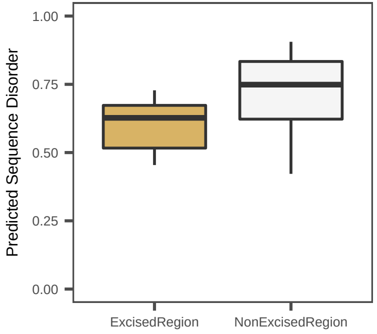

C
