# Supplementary material for: Splice-Junction-Based Mapping of Alternative Isoforms in the Human Proteome
Source: Cell Rep. Author manuscript; Available in PMC 2020 Jan 15. (PMC6961840; doi:10.1016/j.celrep.2019.11.026)
Supplement: 3 [file NIHMS1546469-supplement-3.zip › DF2/PXD000561/AdrenalGland-14-P49321-TEDESLVENNDNIDETEGSEEDDK.pdf]

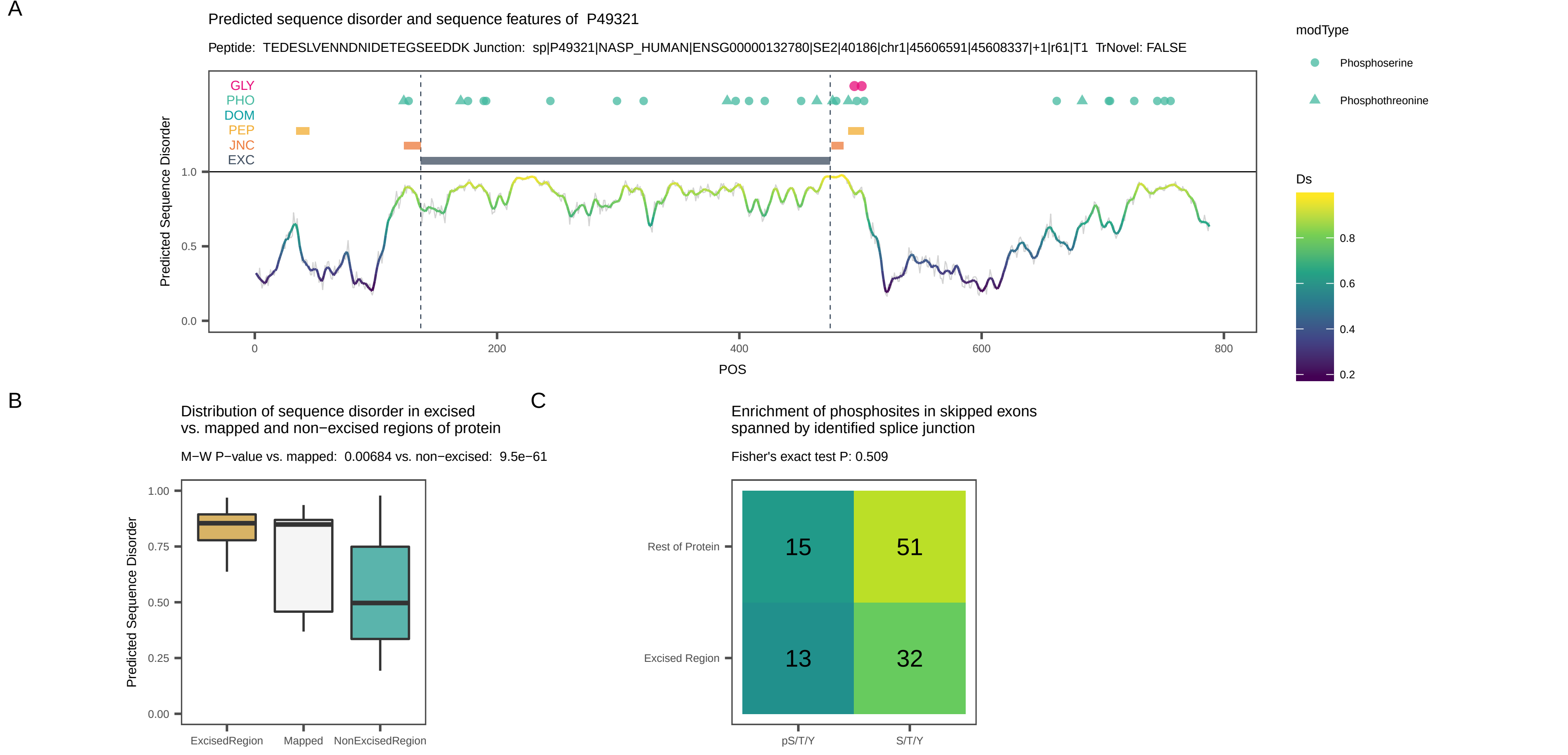
A
Predicted sequence disorder and sequence features of P49321
Peptide: TEDESLVENNDNIDETEGSEEDDK Junction: sp|P49321|NASP_HUMAN|ENSG00000132780|SE2|40186|chr1|45606591|45608337|+1|r61|T1 TrNovel: FALSE
GLY
PHO
DOM
PEP
JNC
EXC
Predicted Sequence Disorder
1.0
0.5
0.0
0
200
400
600
800
POS
modType
Phosphoserine
Phosphothreonine
Ds
0.8
0.6
0.4
0.2
B
Distribution of sequence disorder in excised
vs. mapped and non−excised regions of protein
M−W P−value vs. mapped: 0.00684 vs. non−excised: 9.5e−61
Predicted Sequence Disorder
1.00
0.75
0.50
0.25
0.00
ExcisedRegion
Mapped
NonExcisedRegion
C
Enrichment of phosphosites in skipped exons
spanned by identified splice junction
Fisher's exact test P: 0.509
Rest of Protein
Excised Region
15
51
13
32
pS/T/Y
S/T/Y
