# Supplementary material for: Splice-Junction-Based Mapping of Alternative Isoforms in the Human Proteome
Source: Cell Rep. Author manuscript; Available in PMC 2020 Jan 15. (PMC6961840; doi:10.1016/j.celrep.2019.11.026)
Supplement: 3 [file NIHMS1546469-supplement-3.zip › DF2/PXD000561/Testis-113-Q9ULL0-KNLQVIVEPKEEEPNLPLVSEEEK.pdf]

A

Predicted sequence disorder and sequence features of Q9ULL0

Peptide: KNLQVIVEPKEEEPNLPLVSEEEK Junction: sp|Q9ULL0|K1210_HUMAN|ENSG00000250423|SE1|60658|chrX|119093775|119096691|−0|r33|T1 TrNovel: TRUE

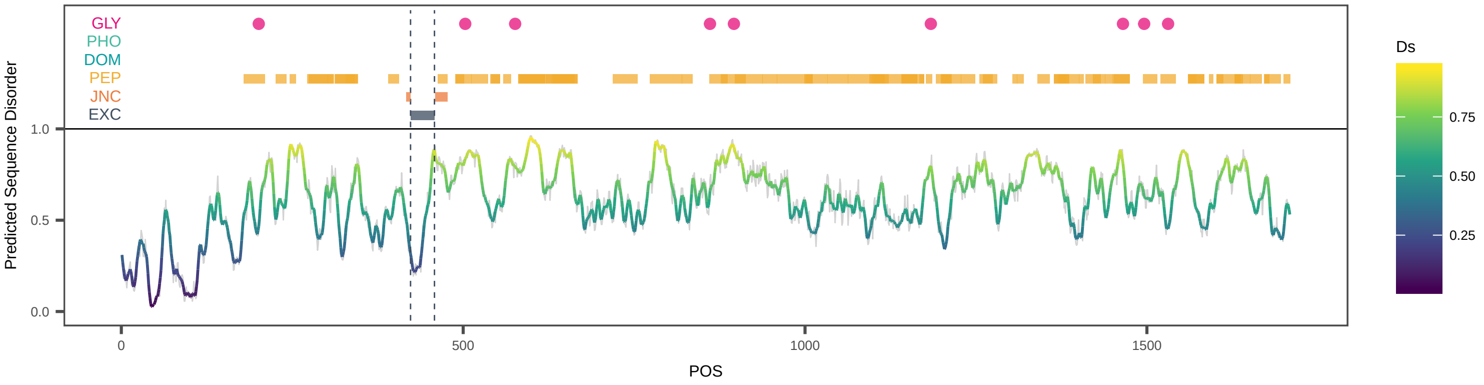

B

Distribution of sequence disorder in excised vs. mapped and non−excised regions of protein

M−W P−value vs. mapped: 3.55e−08 vs. non−excised: 8.23e−06

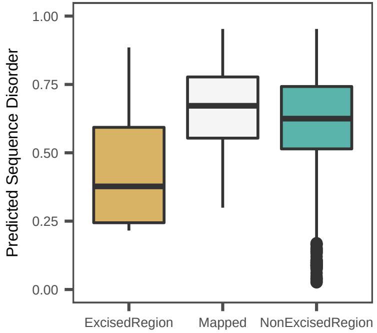

C
