# Supplementary material for: Splice-Junction-Based Mapping of Alternative Isoforms in the Human Proteome
Source: Cell Rep. Author manuscript; Available in PMC 2020 Jan 15. (PMC6961840; doi:10.1016/j.celrep.2019.11.026)
Supplement: 3 [file NIHMS1546469-supplement-3.zip › DF2/PXD000561/Prostate-80-P49407-GGDVAVELPFTLMHPK.pdf]

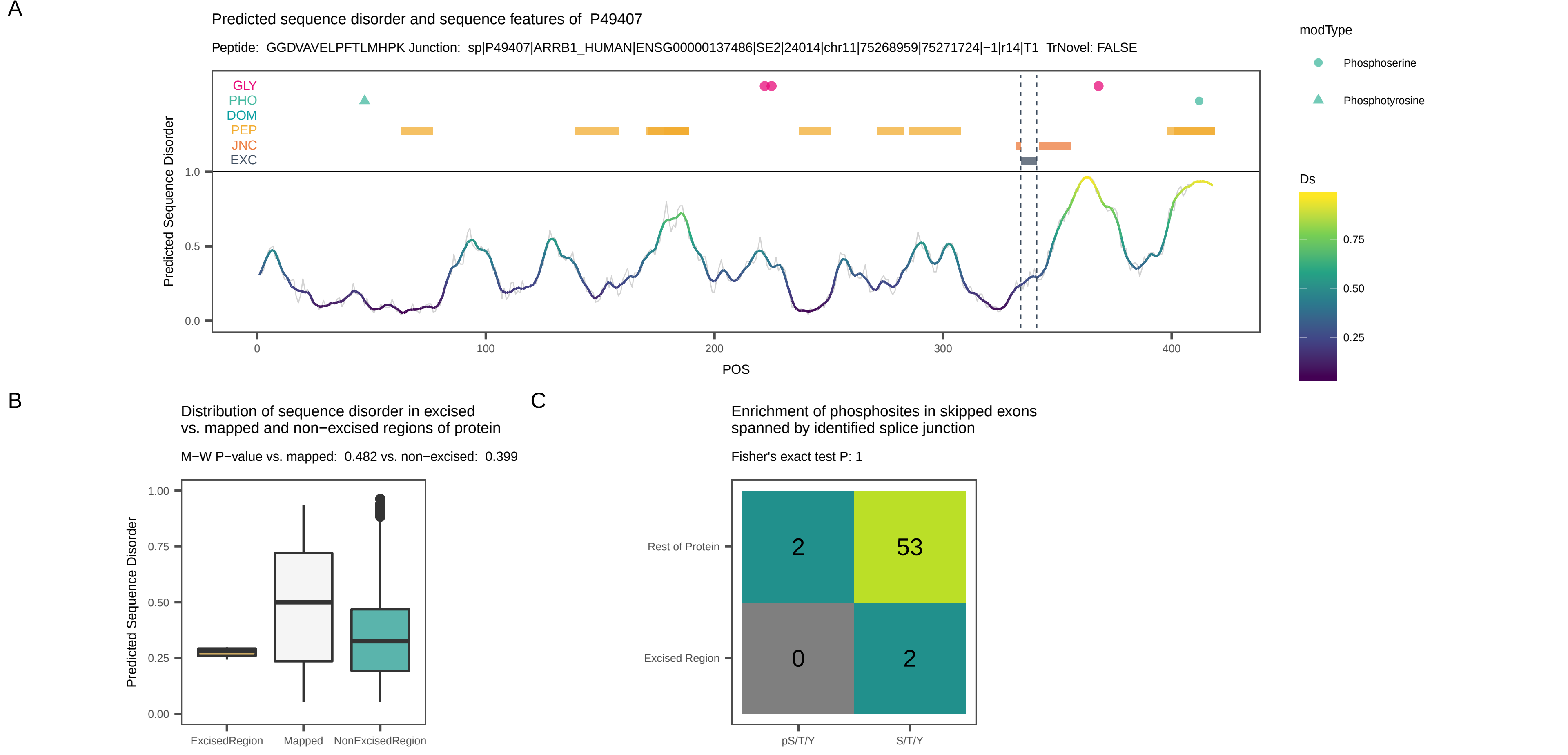
A
Predicted sequence disorder and sequence features of P49407
Peptide: GGDVAVELPFTLMHPK Junction: sp|P49407|ARRB1_HUMAN|ENSG00000137486|SE2|24014|chr11|75268959|75271724|−1|r14|T1 TrNovel: FALSE
GLY
PHO
DOM
PEP
JNC
EXC
Predicted Sequence Disorder
1.0
0.5
0.0
0
100
200
300
400
POS
modType
Phosphoserine
Phosphotyrosine
Ds
0.75
0.50
0.25
B
Distribution of sequence disorder in excised
vs. mapped and non−excised regions of protein
M−W P−value vs. mapped: 0.482 vs. non−excised: 0.399
Predicted Sequence Disorder
1.00
0.75
0.50
0.25
0.00
ExcisedRegion
Mapped
NonExcisedRegion
C
Enrichment of phosphosites in skipped exons
spanned by identified splice junction
Fisher's exact test P: 1
Rest of Protein
Excised Region
2
53
0
2
pS/T/Y
S/T/Y
