# Supplementary material for: Splice-Junction-Based Mapping of Alternative Isoforms in the Human Proteome
Source: Cell Rep. Author manuscript; Available in PMC 2020 Jan 15. (PMC6961840; doi:10.1016/j.celrep.2019.11.026)
Supplement: 3 [file NIHMS1546469-supplement-3.zip › DF2/PXD000561/Pancreas-30-O14936-TQSSSCEIYVR.pdf]

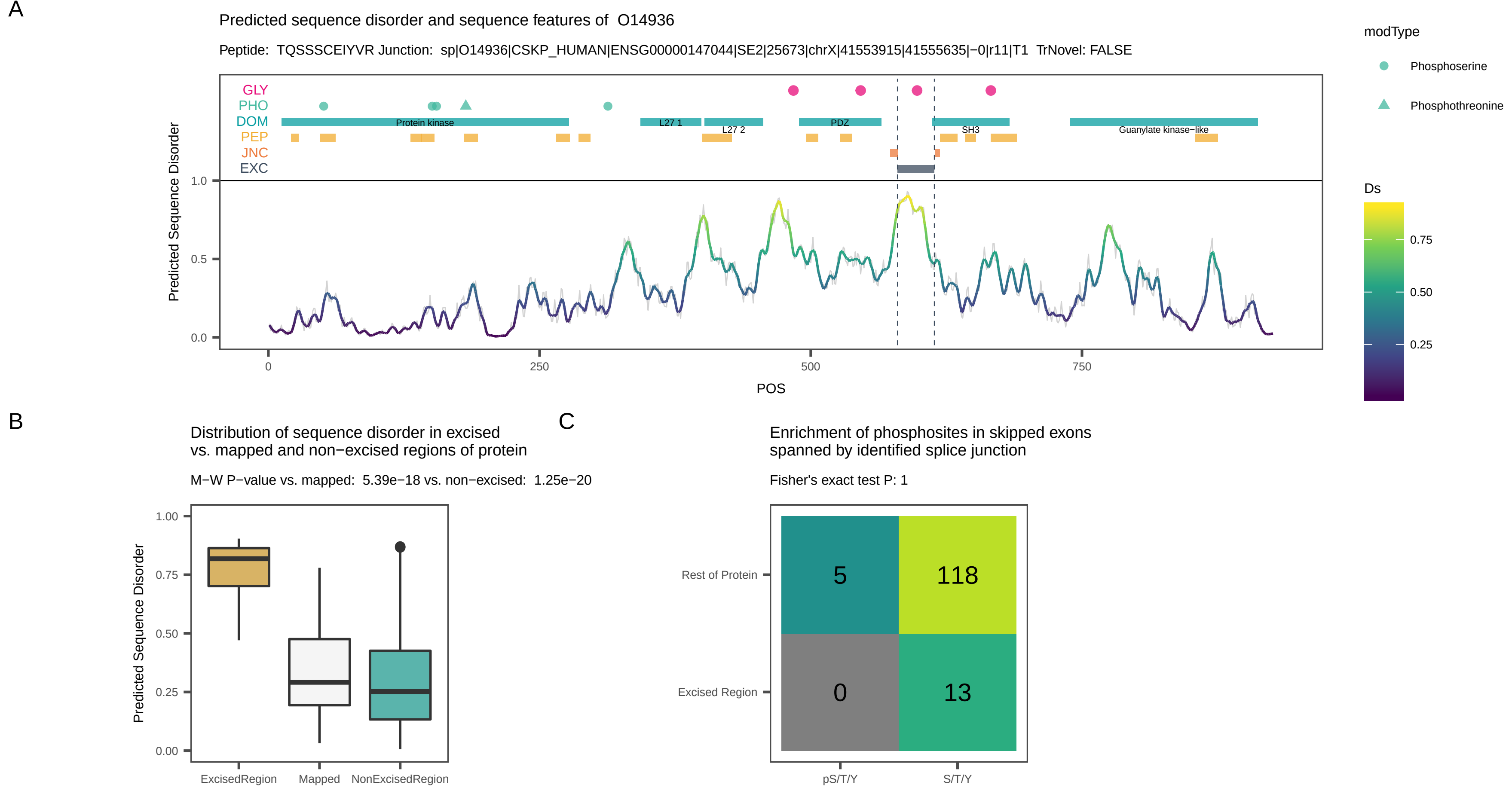
A
Predicted sequence disorder and sequence features of O14936
Peptide: TQSSSCEIYVR Junction: sp|O14936|CSKP_HUMAN|ENSG00000147044|SE2|25673|chrX|41553915|41555635|−0|r11|T1 TrNovel: FALSE
GLY
PHO
DOM
PEP
JNC
EXC
Protein kinase
L27 1
L27 2
PDZ
SH3
Guanylate kinase−like
Predicted Sequence Disorder
1.0
0.5
0.0
0
250
500
750
POS
modType
Phosphoserine
Phosphothreonine
Ds
0.75
0.50
0.25
B
Distribution of sequence disorder in excised
vs. mapped and non−excised regions of protein
M−W P−value vs. mapped: 5.39e−18 vs. non−excised: 1.25e−20
Predicted Sequence Disorder
1.00
0.75
0.50
0.25
0.00
ExcisedRegion
Mapped
NonExcisedRegion
C
Enrichment of phosphosites in skipped exons
spanned by identified splice junction
Fisher's exact test P: 1
Rest of Protein
Excised Region
5
118
0
13
pS/T/Y
S/T/Y
