# Supplementary material for: Splice-Junction-Based Mapping of Alternative Isoforms in the Human Proteome
Source: Cell Rep. Author manuscript; Available in PMC 2020 Jan 15. (PMC6961840; doi:10.1016/j.celrep.2019.11.026)
Supplement: 3 [file NIHMS1546469-supplement-3.zip › DF2/PXD000561/Heart-156-Q53H47-QENLPVGAWPPGAAPAPFQTMK.pdf]

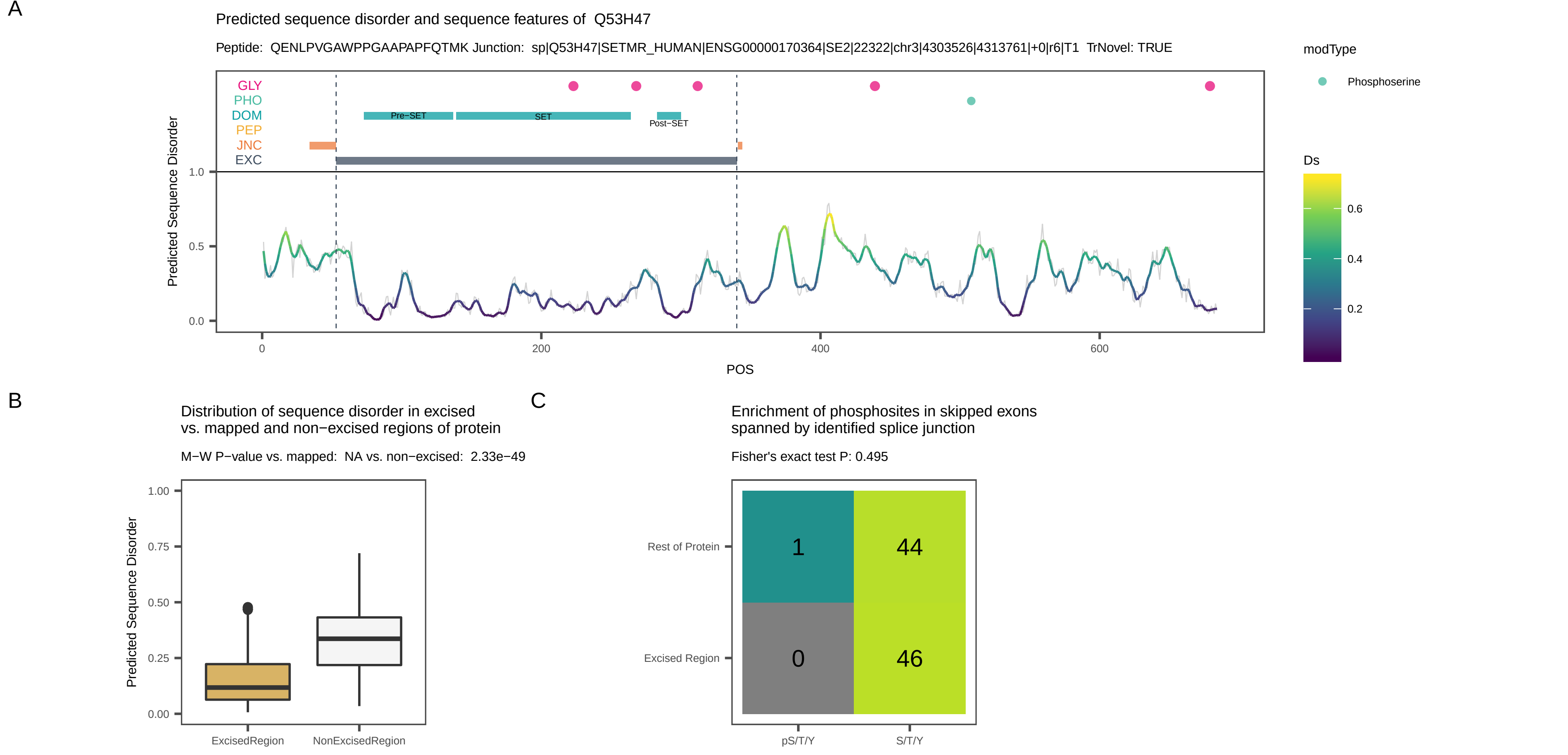
A
Predicted sequence disorder and sequence features of Q53H47
Peptide: QENLPVGAWPPGAAPAPFQTMK Junction: sp|Q53H47|SETMR_HUMAN|ENSG00000170364|SE2|22322|chr3|4303526|4313761|+0|r6|T1 TrNovel: TRUE
GLY
PHO
DOM
PEP
JNC
EXC
Pre−SET
SET
Post−SET
Predicted Sequence Disorder
1.0
0.5
0.0
0
200
400
600
POS
modType
Phosphoserine
Ds
0.6
0.4
0.2
B
Distribution of sequence disorder in excised
vs. mapped and non−excised regions of protein
M−W P−value vs. mapped: NA vs. non−excised: 2.33e−49
Predicted Sequence Disorder
1.00
0.75
0.50
0.25
0.00
ExcisedRegion
NonExcisedRegion
C
Enrichment of phosphosites in skipped exons
spanned by identified splice junction
Fisher's exact test P: 0.495
Rest of Protein
Excised Region
1
44
0
46
pS/T/Y
S/T/Y
