# Supplementary material for: Splice-Junction-Based Mapping of Alternative Isoforms in the Human Proteome
Source: Cell Rep. Author manuscript; Available in PMC 2020 Jan 15. (PMC6961840; doi:10.1016/j.celrep.2019.11.026)
Supplement: 3 [file NIHMS1546469-supplement-3.zip › DF2/PXD000561/AdrenalGland-52-Q15257-VSEAIEK.pdf]

A

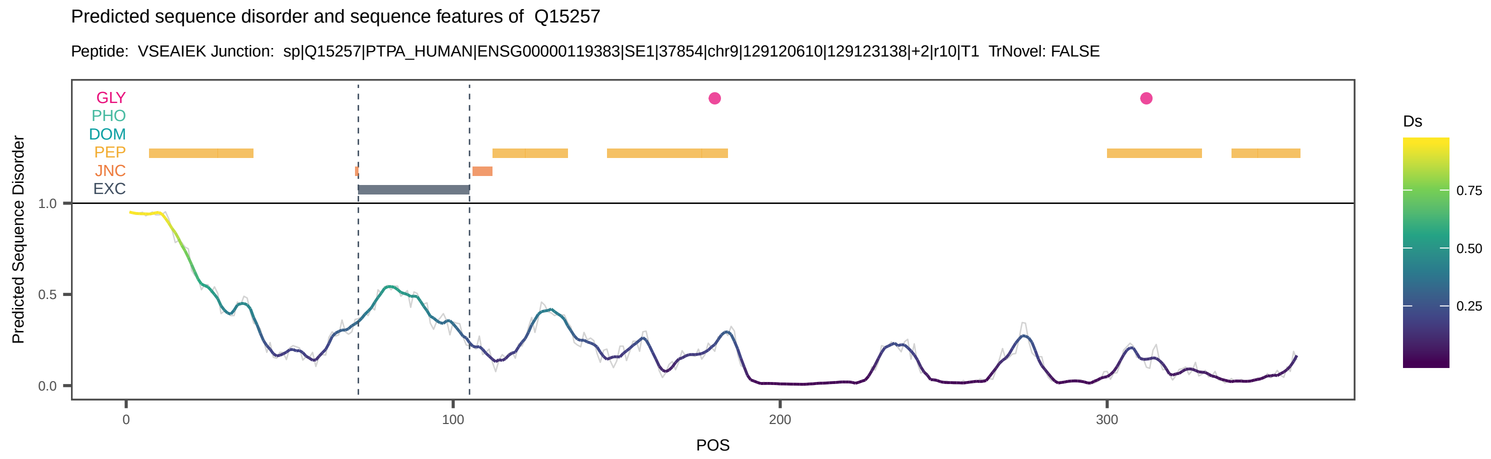
Predicted sequence disorder and sequence features of Q15257
Peptide: VSEAIEK Junction: sp|Q15257|PTPA_HUMAN|ENSG00000119383|SE1|37854|chr9|129120610|129123138|+2|r10|T1 TrNovel: FALSE
GLY
PHO
DOM
PEP
JNC
EXC
Predicted Sequence Disorder
1.0
0.5
0.0
0
100
200
300
POS
Ds
0.75
0.50
0.25

B

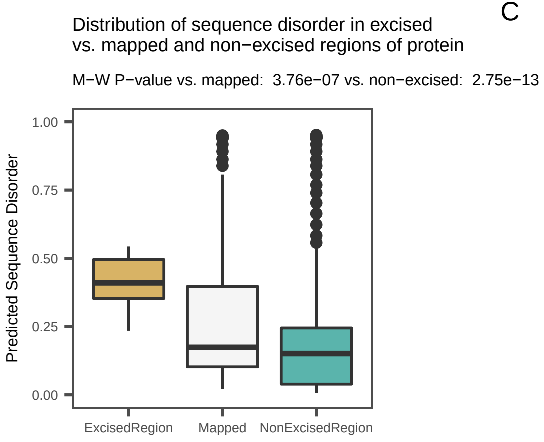
Distribution of sequence disorder in excised vs. mapped and non−excised regions of protein
M−W P−value vs. mapped: 3.76e−07 vs. non−excised: 2.75e−13
Predicted Sequence Disorder
1.00
0.75
0.50
0.25
0.00
ExcisedRegion
Mapped
NonExcisedRegion

C
